# Chemoselective Late-Stage Functionalization of Peptides via Photocatalytic C2-Alkylation of Tryptophan

**DOI:** 10.1021/acs.orglett.3c01795

**Published:** 2023-07-18

**Authors:** Joanna C. Lee, James D. Cuthbertson, Nicholas J. Mitchell

**Affiliations:** † School of Chemistry, University of Nottingham, University Park, Nottingham NG7 2RD, United Kingdom; ‡ School of Chemistry, GlaxoSmithKline Carbon Neutral Laboratories for Sustainable Chemistry, University of Nottingham, Jubilee Campus, Nottingham NG7 2TU, United Kingdom

## Abstract

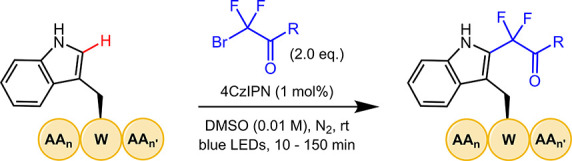

Across eukaryotic proteomes, tryptophan is the least abundant of the 20 canonical amino acids, which makes it an ideal chemical handle for the late-stage functionalization of peptide and protein scaffolds with minimal production of undesired isoforms. Herein, we report the photocatalytic C2-alkylation of tryptophan using bromodifluoroacetate/acetamide-derived radical precursors. This rapid visible-light-mediated reaction is additive-free, operationally simple, and tolerates diverse functionality. We demonstrate the late-stage modification of a variety of complex peptides, including examples of biological significance.

Chemistry that targets the canonical amino acids enables the late-stage functionalization of peptides and the site-selective modification of proteins.^1−3^ Such techniques facilitate the installation of groups to modulate the activity and/or stability of polypeptide tools and therapeutics.^4^ An extensive array of reactions has been developed to target the canonical amino acids.^5^ The majority of these chemoselective methods focus on the amino acids lysine (Lys)^1^ or cysteine (Cys)^6,7^ due to the inherent nucleophilicity of their side chain functional groups. While reactions selective for alternative amino acids have been developed, many of these are less effective on (or cannot be applied to) complex peptides and proteins.^5^

Key considerations when developing methods that target the canonical residues include the inherent reactivity of the side chain and relative proteinogenic abundance of the residue. A broad reactivity profile allows for the application of milder and more varied methods of functionalization, thereby facilitating the development of chemoselective chemistry that is likely to be effective on large and complex peptides and proteins. Techniques that target residues with higher natural abundance, such as Lys, often produce multiple isoforms, which can be challenging to separate from the desired product in larger polypeptide systems. Because of the inherent reactivity of its electron-rich indole side chain and its low natural abundance, tryptophan (Trp) is an ideal proteinogenic chemical handle. This residue accounts for just 1.1% of eukaryotic proteomes,^8^ the lowest of the 20 canonical amino acids. Thus, the sequence of any given target protein is likely to have no more than one Trp residue. If the protein target does not contain this amino acid, then a non-native Trp mutant can be recombinantly expressed.

Previous efforts to target this residue draw on the wealth of indole chemistry reported in the literature. A significant amount of work has focused on transition metal (TM)-catalyzed C–H activation of Trp, including rhodium,^9−11^ manganese,^12,13^ and ruthenium^14,15^ C2-alkylation; palladium-catalyzed C2-arylation^16−19^ and peptide olefination;^20^ gold-catalyzed C2-ethynylation;^21^ and palladium/cobalt-catalyzed macrocyclization.^22−24^ Metal-free strategies toward C2-sulfenylation,^25^ trifluoromethylation,^26,27^ and arylation^28^ have also been explored, as well as the modification of Trp using persistent radical traps.^29^ Over the past decade, photocatalytic C–H functionalization has come to the forefront in this field.^30,31^ Many of these methods have achieved greater success when applied to the functionalization of complex peptides and proteins compared with transition-metal-catalyzed approaches. Examples include alkylation at the C2^32^ and β-positions of Trp.^33^ More recently, electron donor–acceptor (EDA) pyridinium salts have been applied to great effect to target Trp in both peptides and proteins.^34−36^

In 2014, Cho et al. reported a method for the photocatalytic difluoroalkylation of aromatic and heteroaromatic compounds, which included two examples of indole C2-alkylation (Figure 1A).^37^ Considering the success of photocatalytic methods in this field and the advantages of utilizing Trp as a chemical handle, we reasoned that photocatalytic C2-alkylation could potentially be leveraged as an effective peptide modification strategy. During the course of our studies, Paixão and co-workers reported a similar photocatalytic method for Trp C2-alkylation using α-bromo carbonyl compounds (Figure 1B).^38^ However, despite the utility of Paixão et al.’s method, their chemistry relies on a precious metal photocatalyst and requires extended reaction times (24 h), as well as an additive (2,6-lutidine). In order to broaden the scope and extend the applications of Trp C2-alkylation as a bioconjugation strategy, we sought to develop a method that overcomes these limitations. Herein, we report the development of a mild and operationally simple strategy using electron-deficient radicals^39^ for the late-stage alkylation of Trp-containing biomolecules that achieves these goals (Figure 1C). The reaction tolerates diverse functionalities and can be applied to complex peptides. Furthermore, the incorporation of a difluoroalkyl moiety enables the modulation of key physicochemical properties that impact the pharmacokinetics of the molecule and allows the progression and conversion of the reaction to be accurately quantified by ^19^F NMR spectroscopy.

**Figure 1:**
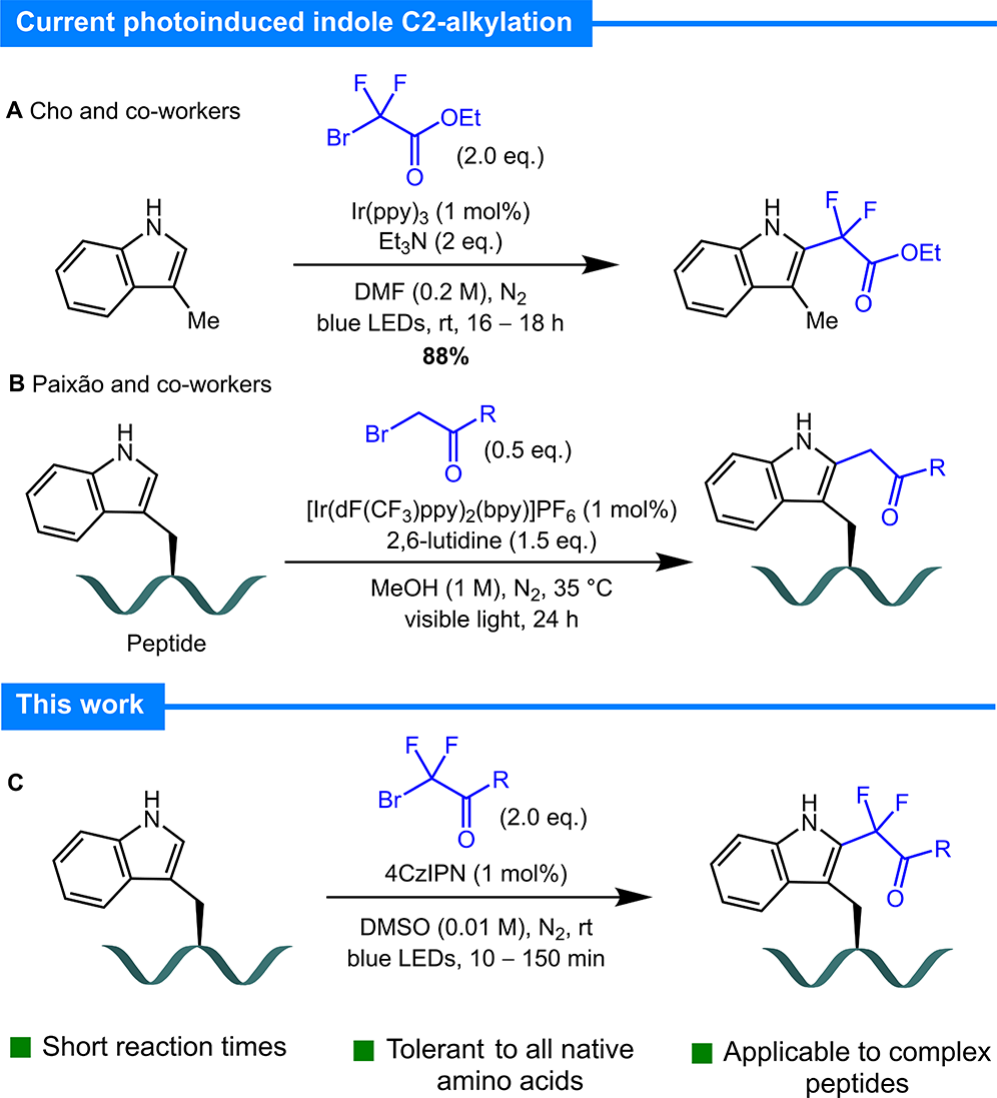
Selected photocatalytic methods for C2-alkylation of indole/tryptophan.

We initially chose to explore the radical functionalization of amino acid Ac-Trp-OEt (**1**) using ethyl bromodifluoroacetate (**2a**) as the radical precursor; irradiation was achieved using a PhotoRedOx Box (Hepatochem) equipped with a 34 mW cm^–2^ LED 450 nm bulb. Reactions run under the conditions reported by the Cho group {0.25 M in DMF, 1 mol % *fac*-[Ir(ppy)_3_], 2 equiv of TEA, blue LEDs^37^} afforded 30% yield by ^19^F NMR over a 17 h period. Following optimization, we identified suitable conditions using the organic photocatalyst 4-CzIPN [1,2,3,5-tetrakis(carbazol-9-yl)-4,6-dicyanobenzene 2,4,5,6-tetrakis(9*H*-carbazol-9-yl) isophthalonitrile]^40^ in DMSO (Table 1, entry 1). Pleasingly, under these conditions, near-complete consumption of starting amino acid **1** was observed (via analytical HPLC) within 10 min. The product was obtained in 71% yield, as determined by ^19^F NMR spectroscopy. Interestingly, stirring was found to have no impact on the reaction and, in some early studies, even resulted in reduced yields because of the formation of a greater proportion of side products; thus, all further reactions were carried out without agitation. Running the reaction under an atmosphere of air gave similar results to an inert atmosphere, highlighting the robustness of the chemistry (Table 1, entry 2). However, to avoid undesired oxidation of residues, such as Cys and methionine (Met), in more complex starting materials, an inert atmosphere was preferred. The reaction was observed to proceed similarly when run in DMF (Table 1, entry 3); however, running the reaction in MeCN severely affected the conversion, with a significant proportion of starting material **1** left unreacted after 10 min (Table 1, entry 4). The reaction tolerates an organic/aqueous solvent mixture up to 10% aqueous (Table 1, entry 5). However, increasing the amount of water further (50% aqueous DMSO) resulted in minimal product formation (Table 1, entry 6). Increasing the concentration of the reaction from 0.01 to 0.25 M reduced the yield to 8% after 10 min and 45% after 60 min with approximately 1 equiv. of **2a** left unreacted (Table 1, entry 7). Switching to an iridium(III) photocatalyst [Ir(ppy)_3_] decreased the yield of the reaction (Table 1, entry 8). The use of inexpensive, lower intensity blue LED strips in place of the PhotoRedOx Box resulted in a lower yield and an increase in reaction time (Table 1, entry 9). Leaving the reaction for longer durations (60 min) resulted in a lower yield because of the formation of undesired side products (overalkylation of the indole ring, Table 1, entry 10). Scale-up of the reaction from 60 μmol of amino acid **1** to 0.2 mmol had a negligible effect on the yield of product **3a** (Table 1, entry 11). Following isolation and purification by flash chromatography, the desired product was obtained in a yield that was comparable with the yield determined spectroscopically. Finally, it was confirmed that the reaction does not proceed in the absence of photocatalyst or light (Table 1, entry 12).

**Table 1:**
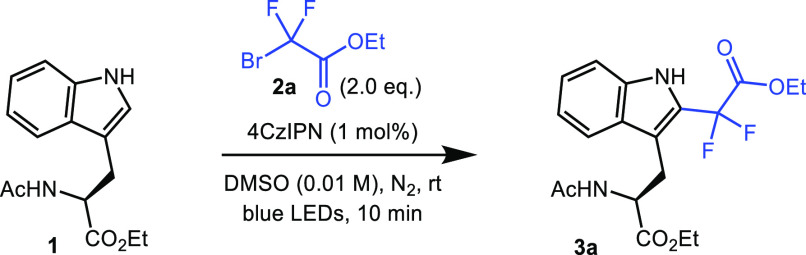
Optimization Studies and Control Reactions^a^

entry	deviation from standard conditions	% yield^b^
1	none	71
2	aerobic	61
3	DMF	61
4	MeCN	23
5	DMSO:H_2_O (9:1)	71
6	DMSO:H_2_O (1:1)	2
7	DMSO (0.25 M)	45^c^
8	Ir(ppy)_3_	56
9	blue LED strip -60 min	53
10	60 min	45
11	0.2 mmol “scale-up”	65 [64^d^]
12	no PC/no light	0

a Reactions performed under a nitrogen atmosphere on a 60.0 μmol scale.

b Yield of product **3a** determined by ^19^F NMR spectroscopy using α,α,α-trifluorotoluene as the internal standard.

c 60 min, 120 μmol scale.

d Isolated yield.

With optimized conditions in hand, we sought to explore the scope of the alkylating agent **2**. Several bromodifluoroacetates (**2b**–**e**) and bromodifluoroacetamides (**2f**–**l**) carrying a range of sterically and electronically different groups, including aliphatic, aromatic, cyclic, and heterocyclic moieties, were prepared [Figure 2, see the Supporting Information (SI) for experimental details]. Radical precursors bearing functionality applicable within the context of chemical biology, such as a PEG chain, an alkyne, a biotin reporter tag, and a sugar moiety, were included in this series. Applying the standard conditions developed for bromide **2a**, we repeated the reaction using these compounds to alkylate amino acid **1** (Figure 2A). The *“esterseries”* of radical precursors (**2a**–**d**) gave the product in yields ranging from 60 to 79% (**3a**–**d**), as determined by ^19^F NMR spectroscopy. Reaction monitoring via analytical HPLC indicated that the reactions were very close to completion within 10–15 min for this series. Past this time point range, further undesired overalkylation of the indole was observed, which resulted in lower yields (Figure 2B). Notably, the reaction using radical precursor **2e** bearing an alkyne group gave a lower yield (21%), even after extended reaction times, which was attributed to undesired radical addition to the alkyne. The “*amide series*” of radical precursors gave yields from 39 to 65% as determined by ^19^F NMR spectroscopy (**3f**–**k**). Reaction monitoring indicated that the reactions were complete within 15–150 min. The reactions with benzyl derivatives **2b** and **2h** were repeated on a larger scale (0.2 mmol) to enable isolation and full characterization of the products. Both **3b** and **3h** were isolated in the same yields as those determined by ^19^F NMR spectroscopy for the smaller-scale reactions.

**Figure 2:**
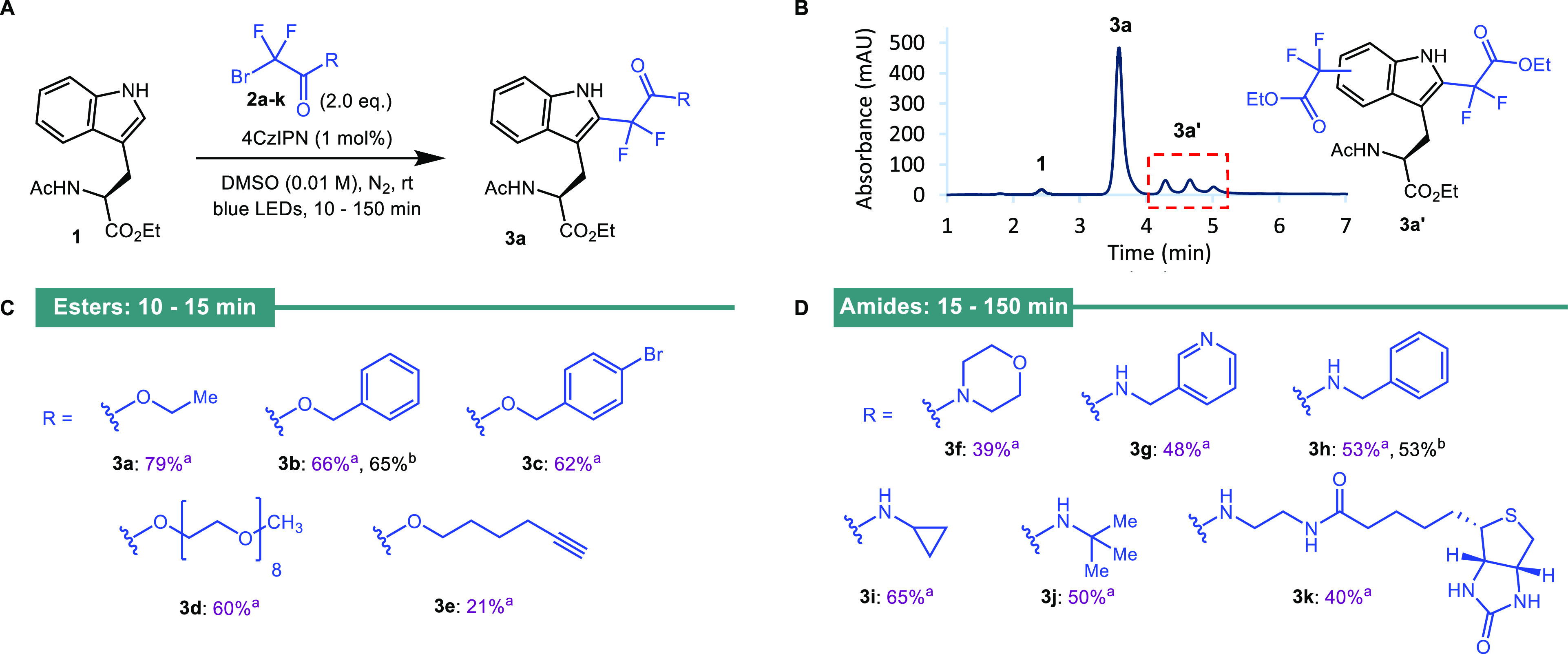
Photochemical alkylation of Ac-Trp-OEt **1**. (A) Reactions performed on a 20.0 μmol scale. ^a^Yields were determined by ^19^F NMR spectroscopy using α,α,α-trifluorotoluene as the internal standard. ^b^Isolated yield was on a 200 μmol scale. (B) Analytical HPLC chromatogram of the crude reaction mixture for the photochemical alkylation of Ac-Trp-OEt **1** using probe **2a** (40–80% gradient over 5 min). (C) Ester series of products produced using standard conditions. (D) Amide series of products produced using standard conditions.

Having investigated how the steric and electronic properties of alkylating agent **2** govern reactivity, we progressed on to an assessment of our strategy on complex peptides. The sequence Ac-WHISKEY-NH_2_**4** was synthesized via Fmoc-SPPS (solid-phase peptide synthesis). Carrying the majority of proteinogenic chemical functionality displayed across the proteome, this model enables a thorough interrogation of the tolerance and chemoselectivity of our reaction (Figure 3). The standard conditions optimized for Ac-Trp-OEt (**1**) were applied to peptide **4** (5 μmol) using radical precursor **2a**. HPLC analysis revealed that the reaction was complete within 10 min; product **5a** was obtained in 55% yield, as determined by quantitative ^19^F NMR spectroscopy. Following purification by preparative RP-HPLC, the product was isolated in 43% yield. Characterization of the product by ^1^H NMR spectroscopy confirmed selective functionalization of Trp, as evidenced by the disappearance of the C2 proton signal.

**Figure 3:**
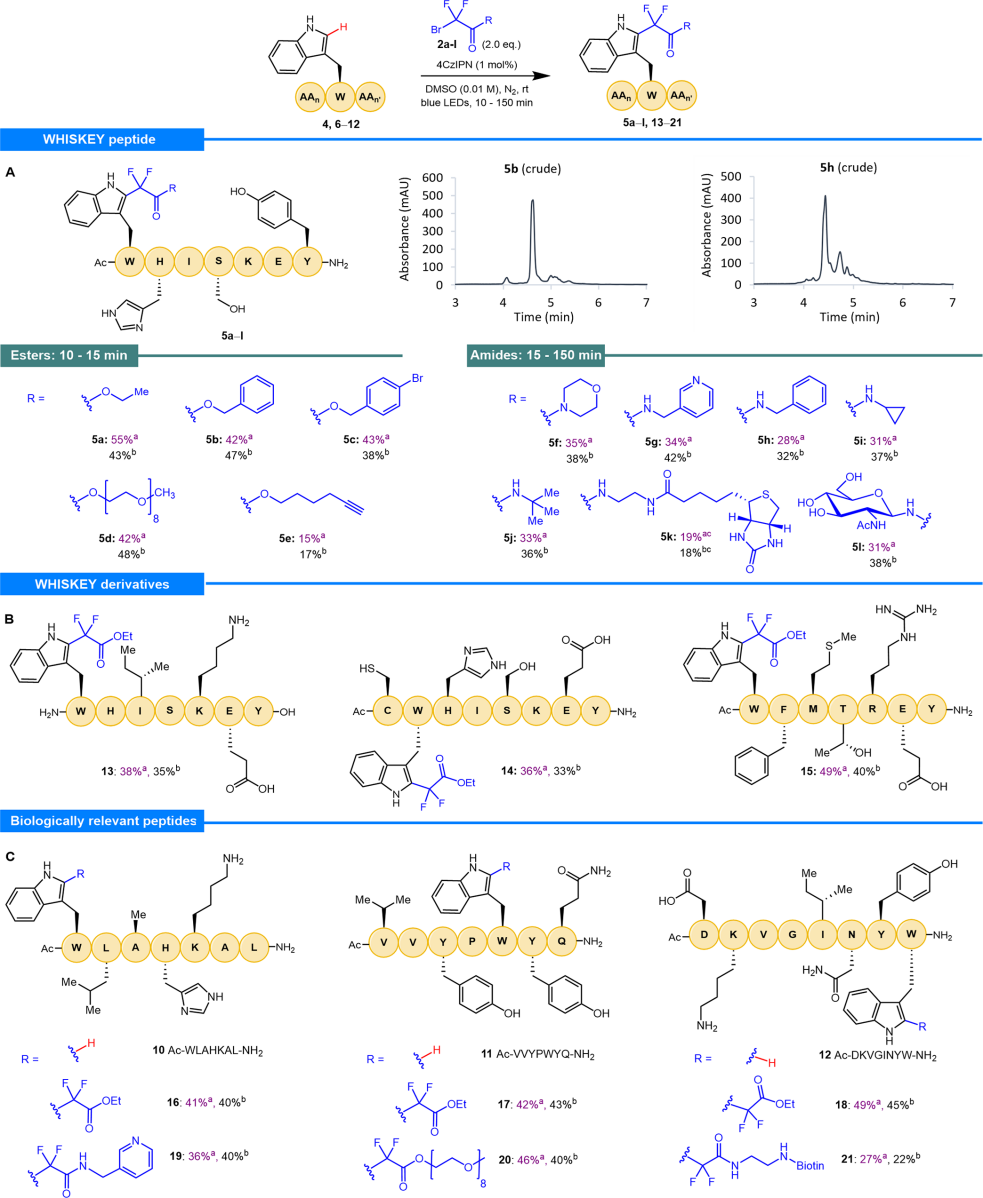
(A–C) Photochemical chemoselective alkylation of Trp-containing peptides (**4, 6–12**); reactions performed on a 5.00 μmol scale. ^a^Yields determined by ^19^F NMR spectroscopy using α,α,α-trifluorotoluene as the internal standard. ^b^Isolated yields. ^c^4CzIPN (5 mol %), bromodifluoroacetamide **2k** (1.5 equiv).

The reaction was repeated with the remaining ester-derived radical precursors (**2b**–**d**) affording yields in the range 42–43% (**5b**–**d**) with comparable isolated yields. The hexyne radical precursor (**2e**) again gave a lower yield (**5e**) with a comparable isolated yield (17%). The *amide series* (**2f**–**l**), including the *N*-acetylglucosamine (Glc*N*Ac) radical precursor acetamide, **2l**, employed to explore C2-glycosylation, afforded yields in the range 19–35% (**5f**–**l**), with the isolated yields mirroring the yields determined spectroscopically. Analysis of the crude reaction mixtures for the *amide series* showed the generation of a greater proportion of alkylated side-products compared with the *ester series* compounds (Figure 3A). However, by monitoring the reaction (HPLC) and controlling the end point, overalkylation of the indole could be minimized. While these yields are lower than those acquired for the functionalization of Ac-Trp-OEt (**1**), they do demonstrate rapid and chemoselective alkylation of Trp on a complex polypeptide. Importantly, when the substrate Ac-HISKEY-NH_2_**6** (with the Trp residue omitted) was exposed to the reaction conditions, no undesired modification of the alternative amino acids was observed.

Encouraged by these results, we prepared model peptides **7**–**9** to probe the reaction in the presence of a free N-terminus and carboxy C-terminus (**7**; H-WHISKEY-OH), a Cys residue (**8**; Ac-CWHISKEY-NH_2_), and additional canonical amino acids with untested reactive functionality (**9**; Ac-WFMTREY-NH_2_). The reaction of these models with radical precursor **2a** was undertaken, and yields similar to those observed for peptide **4** were confirmed (**13**–**15**; Figure 3B). In all cases these translated into comparable isolated yields. In addition to these models, peptides **10**–**12** were synthesized as biologically relevant examples (Figure 3C). Peptide **10** is a dipeptidyl peptidase-4 (DPP-4) inhibitor, and peptides **11** and **12** are angiotensin converting enzyme (ACE) inhibitors. Furthermore, the lone Trp residue in these models is located at different positions in each sequence: N-terminus (**10**), internal (**11**), and C-terminus (**12**), thereby enabling assessment of the effect that the positioning of the Trp residue has on conversion to the product. The reaction of radical precursor **2a** with these sequences under standard conditions gave yields comparable with those of reactions using model peptide **4** (**16**–**18**; Figure 3C). These results suggest that the positioning and local steric and electronic environment surrounding the Trp residue have a negligible effect on the formation of the desired product. Alkylation of the biologically relevant peptides **10**–**12** with radical precursor **2g**, and the more chemically diverse peptides **2d** and **2k**, respectively, was also demonstrated on these models (**19**–**21**; Figure 3C). Pleasingly, the yields were broadly consistent with those achieved using model peptide **4**. The consistency of the yields across the peptide models illustrates the tolerance of this reaction to all proteinogenic functionality.

In conclusion, we have developed a rapid, functional-group-tolerant, and operationally simple photocatalytic difluoroalkylation reaction that enables the chemoselective functionalization of Trp residues in complex peptides. For the majority of the radical precursors explored, the reactions are complete within minutes, regardless of the position of the target residue. The precursors can be readily synthesized using simple, established chemistry, which enables diverse functionality to be introduced into valuable peptide targets at a late stage.

## Data Availability

The data underlying this study are available in the published article and its Supporting Information.

## Supporting Information Available

Experimental details and full characterization data of all compounds (PDF)
