# Supplementary material for: Chemoselective Late-Stage Functionalization of Peptides via Photocatalytic C2-Alkylation of Tryptophan
Source: Org Lett. 2023 Jul 18;25(29):5459–64. doi: 10.1021/acs.orglett.3c01795 (PMC10391624; doi:10.1021/acs.orglett.3c01795)

Chemoselective Late-Stage Functionalization of Peptides via Photocatalytic C2-Alkylation of Tryptophan

Joanna C. Lee,[a,b] James D. Cuthbertson*[a,b], Nicholas J. Mitchell*[a]

[a] *School of Chemistry, University of Nottingham, University Park, Nottingham NG7 2RD, U.K.*

[b] *GlaxoSmithKline Carbon Neutral Laboratories for Sustainable Chemistry, University of Nottingham, Jubilee Campus, Triumph Road, Nottingham, NG7 2TU, U.K.*

*Email: nicholas.mitchell@nottingham.ac.uk

*Email: james.cuthbertson@nottingham.ac.uk

Supplementary Information

Contents

1. General information S3
2. Optimization studies with Ac-Trp-OEt S7
3. Synthesis of radical precursors S11
  3.1 Bromodifluoroacetates S12
  3.2 Bromodifluoroacetamides S14
4. Peptide synthesis S18
5. Photochemical alkylation of Ac-Trp-OEt S26
6. Photochemical alkylation of Trp-containing peptides S41
7. References S66
8. NMR spectra S67
  Radical precursors S68
  Photochemical alkylation of Ac-Trp-OEt S91
  Peptide substrates S103
  Photochemical alkylation of Trp-containing peptides S107

1. General information

^{1}H, ^{13}C and ^{19}F NMR spectra were recorded on a Bruker DPX 400, Bruker AV400, Bruker AV(III)400, Bruker AV(III)400HD or Bruker AV(III)500 spectrometer. Chemical shifts (δ) are reported in parts per million (ppm); coupling constants (J) are quoted in Hertz (Hz) and are reported to the nearest 0.1 Hz. ^{1}H and ^{13}C chemical shifts are reported relative to tetramethylsilane, and are referenced to the appropriate residual solvent peaks: $CDCl_3$ (δ 7.26 ppm [^{1}H]/ δ 77.16 ppm [^{13}C]), CD_3OD (δ 3.31 [^{1}H]/ δ 49.00 [^{13}C]), DMSO-d_6 (δ 2.50 [^{1}H]/ δ 39.52 [^{13}C]. ^{1}H NMR spectral data are reported as follows; chemical shift, multiplicity, number of protons and coupling constant. The following abbreviations (and combinations of) are used to label multiplicities: s (singlet), d (doublet), t (triplet), q (quartet), dd (doublet of doublets), ddd (doublet of doublet of doublets), and m (multiplet). HSQC, DEPT, TOCSY, and COSY experiments were used to assist structural assignments.

Analytical RP-HPLC: Performed on a Thermo Ultimate 3000 μHPLC system equipped with PDA eλ detector (λ = 210 – 400 nm). Amino acids and peptides were analysed using a Waters SunFire 5 μm, 2.1 × 150 mm column (C-18) at a flow rate of 0.6 mL•min^{-1}. The mobile phase was composed of 0.1% trifluoroacetic acid in H_2O (Solvent A) and 0.1% trifluoroacetic acid in acetonitrile (Solvent B). The analysis of the chromatograms was conducted using Chromeleon 7 software.

Preparative RP-HPLC: Performed using a Waters 1525 binary pump HPLC equipped with a dual wavelength UV detector set to 210 nm and 280 nm. Peptides were purified on a Waters SunFire 5 μm (C-18) preparative column, 19 × 150 mm, operating at a flow rate of 6 mL•min^{-1} using a mobile phase of 0.1% trifluoroacetic acid in H_2O (Solvent A) and 0.1% trifluoroacetic acid in MeCN (Solvent B) using the gradient specified for each specific compound. Semi-preparative reverse-phase HPLC was performed using the same HPLC and solvent system. The column used was a Waters SunFire 5 μm (C-18) semi-preparative column, 10 × 250 mm, operating at a flow rate of 5 mL•min^{-1} using the gradient specified for each specific compound.

High-resolution mass spectra were recorded on a Bruker MicroTOF II spectrometer (ESI) and Bruker Ultraflex III (MALDI). Electron ionisation (EI) mass spectra were recorded on a JEOL AccuTOF GCX with the MS ion source conditions: temperature (270 °C), ionisation energy (70 eV) and mass calibration by PFTBA. Infrared spectra (IR) were recorded on a Bruker Alpha platinum-ATR with diamond window. Melting points were measured on a Gallenkamp melting point apparatus.

Analytical TLC was carried out on aluminium-backed silica gel plates (Merck/EMD Millipore, 60 Å pore size, pre-coated with 254 nm-responsive fluorescent dye), and spots were visualised with UV

irradiation (254 and 366 nm). Flash column chromatography was accomplished using silica gel 60 Å (40-63 µm particle size), used as purchased from Sigma-Aldrich, eluting with the solvents listed.

Materials

Starting materials and reagents were obtained from commercial sources and were used without further purification unless stated otherwise. Reagents that were not commercially available were synthesised as outlined for each specific compound.

Automated Solid-Phase Peptide Synthesis (SPPS)

Automated Fmoc-SPPS was carried out on a Biotage Initiator+ Alstra microwave peptide synthesizer. General synthetic procedures for Fmoc-deprotection and capping were carried out in accordance with the manufacturer's specifications. Standardized amino acid couplings were performed for 15 min at 50 $^{\circ}$C under microwave irradiation in the presence of amino acid (0.5 M in DMF, 4.0 equiv.), Oxyma Pure (0.5 M in DMF, 4.0 equiv.) and diisopropylcarbodiimide (0.5 M in DMF, 4.0 equiv.).

Cleavage: For peptides containing Cys/Met, a mixture of TFA, thioanisole, triisopropylsilane (TIPS) and water (85:5:5:5 v/v/v/v) was added to the resin. After 3 h, the resin was washed with TFA (3 × 2 mL). For all other peptides, a mixture of TFA, triisopropylsilane (TIPS) and water (90:5:5 v/v/v) was added to the resin. After 3 h, the resin was washed with TFA (3 × 2 mL).

Work-up: The combined solutions were concentrated under a stream of nitrogen to < 5 mL. Diethyl ether (40 mL) was added to precipitate the peptide and the suspension centrifuged. The pellet was then dissolved in water containing 0.1% TFA, filtered and purified by preparative HPLC and analysed by LC-MS and ESI mass spectrometry.

Photoredox reaction setups

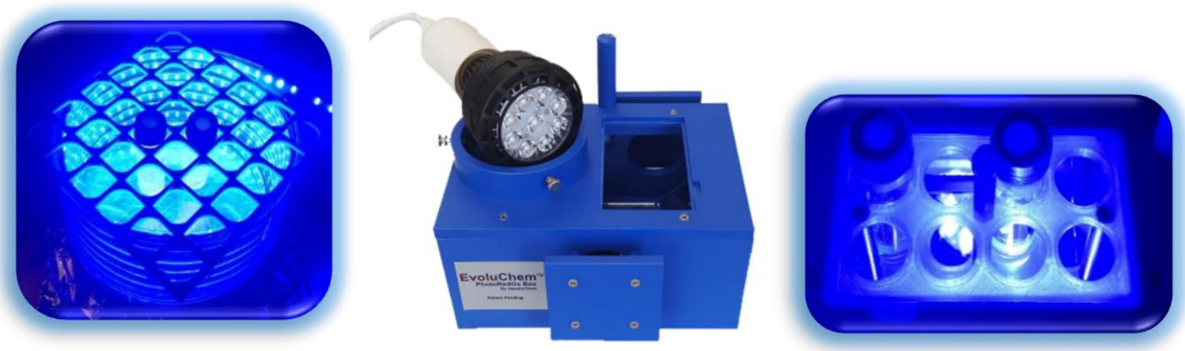

A *Standard setup:* The photoredox setup used standard blue LED strip lights wrapped inside a Pyrex dish. Reaction vessels were chosen based on the reaction volume, using either 8 mL or 20 mL screw top vials. The temperature inside the dish was within the range of 23 - 43 °C.

B *PhotoRedOx box:* The EvoluChem™ PhotoRedOx Box (Hepatochem, HCK1006-01-016) was used with 8 mL and 20 mL vials and EvoluChem™ LED spotlights (HCK1012-01-002, 18 W, 34 mW/cm^2, 450 nm (blue) and 6200 K (white)).

The reactions were set up inside a standard fume hood, then transferred to the specified photoredox reaction setup.

Stock solutions

Where necessary stock solutions of known concentration were made by dissolving a quantity of the substrate/reagent/catalyst in anhydrous DMSO.

Quantitative NMR spectroscopy analysis of reaction mixtures

After irradiating the reaction mixture with blue LEDs for the specified amount of time, α,α,α-trifluorotoluene (0.333 eq.) was added, then a sample (600 µL) was taken for analysis by ^{19}F NMR spectroscopy. The delay and number of scans for this experiment were set to 15 s and 32 scans respectively. Inversion recovery experiments showed that the t0 was 2.5 s. The integral of the α,α,α-trifluorotoluene (TFT) singlet was set to 1, and due to the 1:1 relationship between the standard and product doublets, integration of a product doublet represented the quantitative NMR spectroscopic yield.

Figure S1 - Representative ^{19}F NMR Spectrum of an unpurified reaction mixture (amino acid substrate)

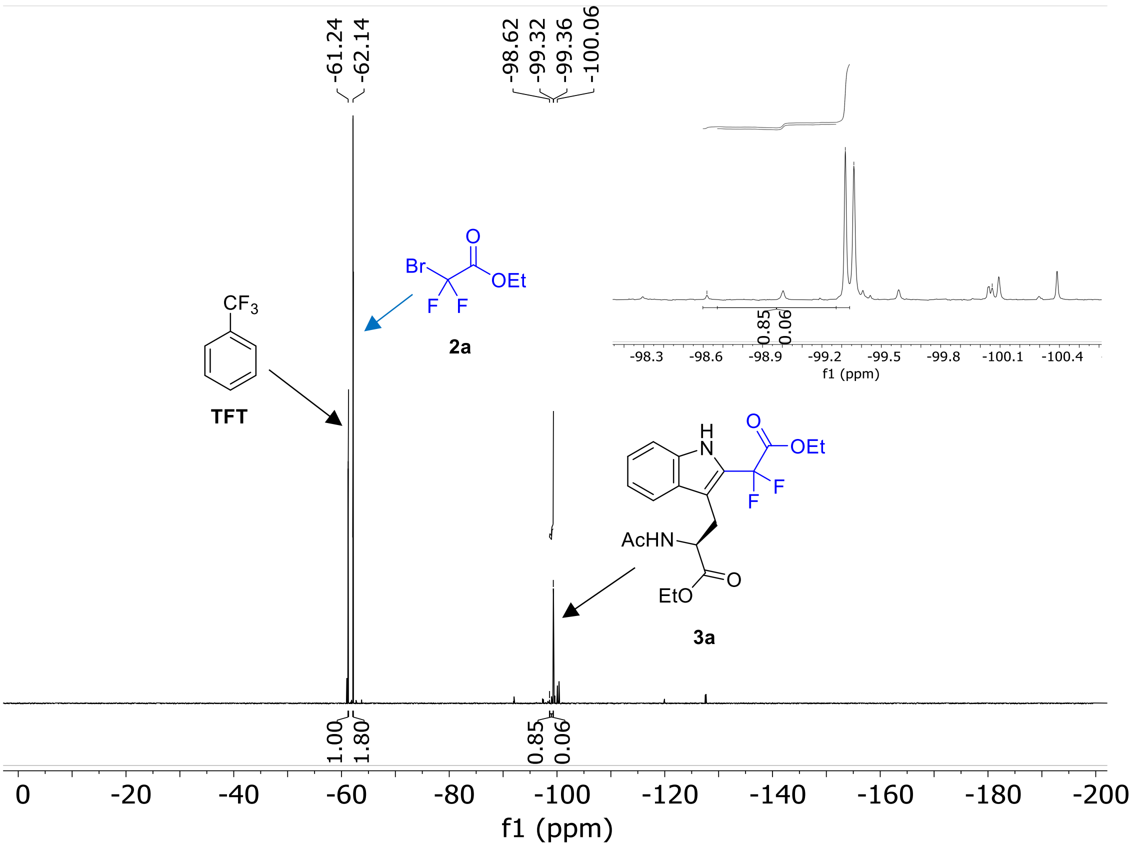

Figure S2 - Representative ^{19}F NMR Spectrum of an unpurified reaction mixture (peptide substrate)

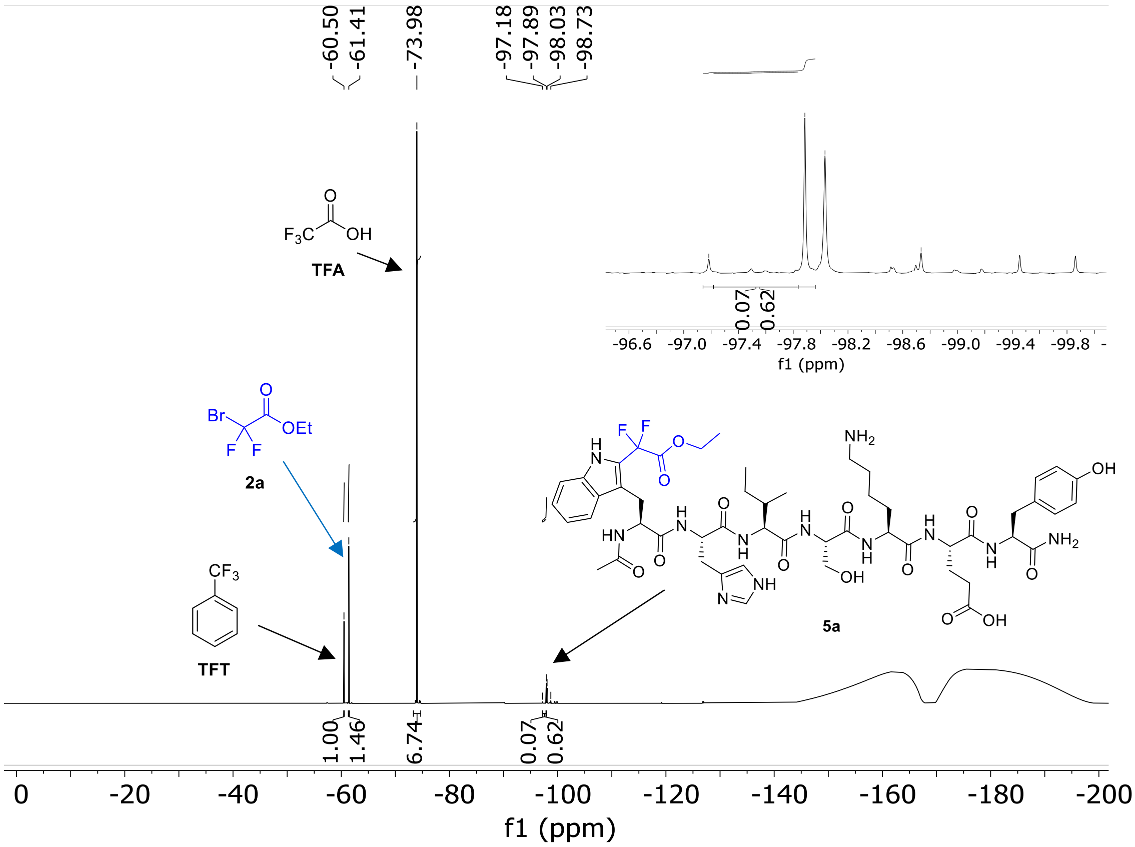

2. Optimization studies with Ac-Trp-OEt

N-acetyl-L-tryptophan ethyl ester (1)

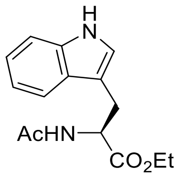

To a stirred suspension of L-tryptophan ethyl ester hydrochloride (1.27 g, 4.73 mmol, 1.00 eq.) in DCM (10 mL, 0.5 M) were added DIPEA (1.80 mL, 10.0 mmol, 2.00 eq.) and acetic anhydride (0.470 mL, 5.00 mmol, 1.00 eq.) under an argon atmosphere and stirred for 3 h. The solution was diluted with water (10 mL) and extracted with DCM (3 × 10 mL). The combined organic extracts were washed with 1 M aq. HCl (10 mL), sat. aq. $NaHCO_3$ (10 mL) and brine (10 mL), dried over $MgSO_4$ and concentrated *in vacuo*. The crude residue was purified by recrystallisation from ethyl acetate and petroleum ether to give the title compound **1** as an off-white crystalline solid (1.13 g, 4.12 mmol, 87%). Characterization data were in accordance with those previously reported.[1]

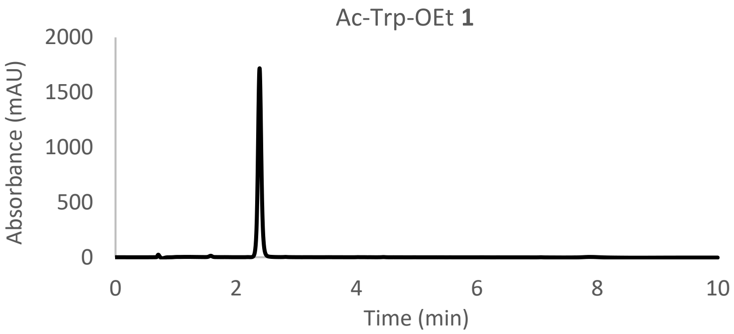

Figure S3. Pure analytical HPLC chromatogram for Ac-Trp-OEt **1**. Rt = 2.39 min on analytical gradient 40-80% B over 5 min, 0.6 mL min^{-1}, 280 nm.

Optimization studies with Ac-Trp-OEt 1

A solution of Ac-Trp-OEt **1** (16.5 mg, 60.0 µmol, 1.00 eq.), photocatalyst, base and ethyl bromodifluoroacetate **2a** in solvent was purged with N_2 (5 min) and irradiated with blue LEDs (PhotoRedOx box, 18 W) for 10 min at room temperature. α,α,α-trifluorotoluene (20 µL, 1.0 M in DMSO, 20.0 µmol, 0.333 eq.) was added to the reaction mixture before quantitative NMR spectroscopy, analytical HPLC and MS analysis was carried out.

Table S1 – Optimization studies – solvent screen

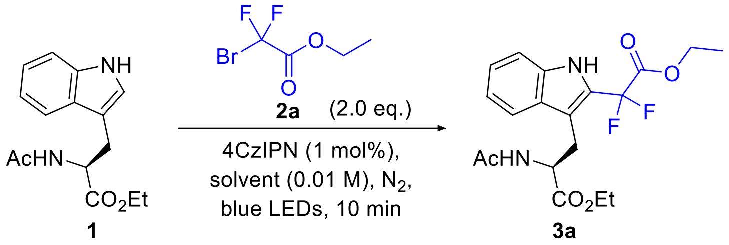

Entry	Solvent	% Yield[a]
1	DMSO	71
2	DMF	61
3	MeCN	22
4	9:1 DMSO:H_2O	71
5	1:1 DMSO:H_2O	2
6	DMSO (0.25 M)	45[b]

Reactions performed under a nitrogen atmosphere on a 60.0 µmol scale. [a]Yield of **3a** determined by ^{19}F NMR spectroscopy using α,α,α-trifluorotoluene as the internal standard. [b]60 min, 120 µmol scale

Table S2 – Optimization studies – base screen

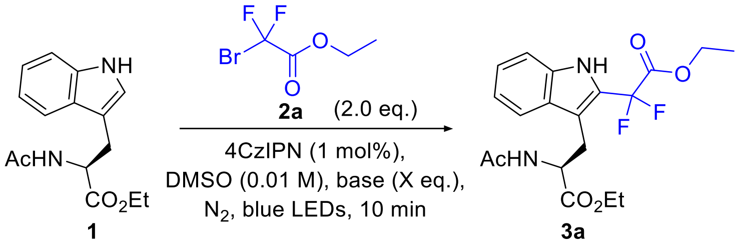

Entry	Base (X eq.)	% Yield[a]
1	-	68
2	TEA (2.0)	6
3	TEA (1.0)	12
4	K_2CO_3 (1.0)	62
5	K_3PO_4 (1.0)	66
6	NaOH (1.0)	65

Reactions performed under a nitrogen atmosphere on a 60.0 µmol scale. [a]Yield of **3a** determined by ^{19}F NMR spectroscopy using α,α,α-trifluorotoluene as the internal standard.

Table S3 – Optimization studies – photocatalyst screen

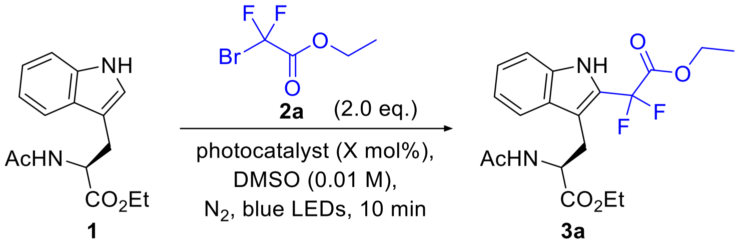

Entry	Photocatalyst (X mol%)	% Yield[a]
1	4CzIPN (1.0)	70
2	4CzIPN (0.5)	62
3	4CzIPN (2.0)	79
4	4CzIPN (3.0)	78
5	*fac*-Ir(ppy)$_3$ (1.0)	56
6	3CzClIPN (1.0)	44
7	Ru(bpy)$_3$·6H$_2$O (1.0)	0
8	Eosin y (1.0)	0
9	-	0

Reactions performed under a nitrogen atmosphere on a 60.0 µmol scale. [a]Yield of **3a** determined by ^{19}F NMR spectroscopy using α,α,α-trifluorotoluene as the internal standard.

Table S4 – Optimization studies – time and ethyl bromodifluoroacetate **2a** equivalent screen

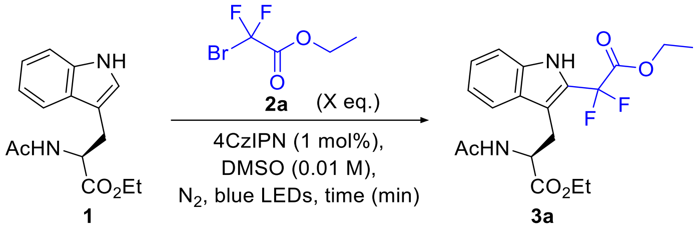

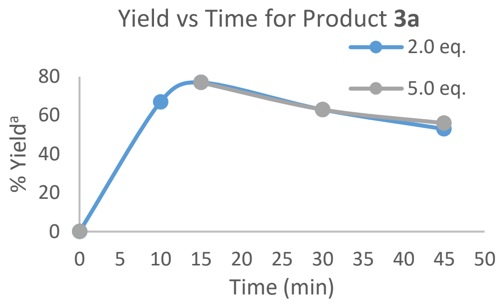

Entry	2a eq.	Time (min)	% Yield[a]
1	1.0	10	70
2	2.0	10	68
3	3.0	10	75
4	5.0	10	77[b]
5	2.0	15	77[b]
6	2.0	30	63
7	2.0	45	53
8	5.0	15	73
9	5.0	30	63
10	5.0	45	56

Reactions performed under a nitrogen atmosphere on a 60.0 μmol scale. [a]Yield of **3a** determined by ^{19}F NMR spectroscopy using α,α,α-trifluorotoluene as the internal standard. [b]average of 2 experiments.

3. Synthesis of radical precursors

General Procedure A: Synthesis of bromodifluoroacetates and bromodifluoroacetamides

Using a modification of the procedure reported by Jin and co-workers.[2] Under an inert atmosphere (N_2) a solution of alcohol or amine (1.00 mmol, 1.00 eq.) and triethylamine (279 µL, 2.00 mmol, 2.00 eq.) in anhydrous DCM (3 mL, 0.333 M) was cooled to 0 °C. A solution of bromodifluoroacetyl chloride (188 µL, 2.00 mmol, 2.00 eq.) in anhydrous DCM (1 mL, 2.00 M) was added dropwise. The reaction was allowed to warm to room temperature and stirring was continued for 16 - 18 h. The reaction mixture was diluted with water (5 mL) and washed with sat. aq. NH_4Cl (2 × 10 mL), sat. aq. $NaHCO_3$ (2 × 10 mL) and brine (10 mL), dried ($MgSO_4$) and concentrated *in vacuo*. The resulting residue was purified by flash column chromatography.

General Procedure B: Synthesis of bromodifluoroacetamides[4]

Prepared according to the procedure reported by Ohshima and co-workers.[3] To a glass vial, equipped with a stirrer bar, lanthanum trifluoromethanesulfonate (26.4 mg, 45.0 µmol, 5 mol%) was added then the vial was sealed with a silicone septum screw-cap and purged with N_2 (5 min). Ethyl bromodifluoroacetate **2a** (139 µL, 1.08 mmol, 1.20 eq.) and amine (0.900 mmol, 1.00 eq.) were added, then the mixture was stirred at rt for 17-20 h. The reaction was monitored by TLC analysis and once complete consumption of the starting material was observed, the crude mixture was directly purified by flash column chromatography.

3.1 Bromodifluoroacetates

Benzyl 2-bromo-2,2-difluoroacetate (2b)

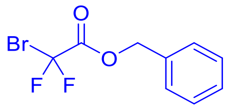

Prepared according to general procedure A using benzyl alcohol (104 µL, 1.00 mmol, 1.00 eq.), triethylamine (279 µL, 2.00 mmol, 2.00 eq.) and bromodifluoroacetyl chloride (188 µL, 2.00 mmol, 2.00 eq.). The crude material was purified by flash column chromatography (SiO_2, pentane/EtOAc, 9:1) to give the title compound **2b** (152 mg, 0.573 mmol, 57%) as a colourless oil. Characterization data were in accordance with those previously reported.[4]

4-Bromobenzyl 2-bromo-2,2-difluoroacetate (2c)

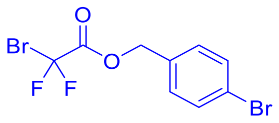

Prepared according to general procedure A using 4-bromo benzyl alcohol (187 µL, 1.00 mmol, 1.00 eq.), triethylamine (279 µL, 2.00 mmol, 2.00 eq.) and bromodifluoroacetyl chloride (188 µL, 2.00 mmol, 2.00 eq.). The crude material was purified by flash column chromatography (SiO_2, pentane/EtOAc, 9:1) to give the title compound **2c** (167 mg, 0.485 mmol, 49%) as a colourless oil; R_f = 0.93 (pentane/EtOAc, 9:1), υ_{max}/cm^{-1} (ATR) 2964, 2928, 2895, 2854, 1773, 1595, 1490; ^{1}H NMR (400 MHz, $CDCl_3$) δ 7.61 – 7.49 (m, 2H), 7.32 – 7.25 (m, 2H), 5.31 (s, 2H); ^{13}C {^{1}H} NMR (101 MHz, $CDCl_3$) δ 159.5 (t, J = 31.6 Hz), 132.5, 132.2, 130.3, 123.6, 108.7 (t, J = 314 Hz), 69.0; ^{19}F NMR (376 MHz, $CDCl_3$) δ −60.8 (s, 2F); HRMS (EIMS, 70 eV) m/z: $[M]^+$ Calcd for $C_9H_6{}^{79}Br_2F_2O_2$ 341.8684; Found: 341.8697 (3.7 ppm error).

2,5,8,11,14,17,20,23-Octaoxapentacosan-25-yl 2-bromo-2,2-difluoroacetate (2d)

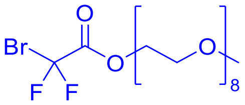

Prepared according to general procedure A using 2,5,8,11,14,17,20,23-octaoxapentacosan-25-ol (353 µL, 1.00 mmol, 1.00 eq.), triethylamine (279 µL, 2.00 mmol, 2.00 eq.) and bromodifluoroacetyl chloride (188 µL, 2.00 mmol, 2.00 eq.). The crude material was purified by flash column chromatography (SiO_2, DCM/MeOH, 95:5) to give the title compound **2d** (237 mg, 0.439 mmol, 44%) as a yellow oil; R_f = 0.46 (DCM/MeOH, 95:5), υ_{max}/cm^{-1} (ATR) 2913, 2870, 1774, 1740, 1579, 1452; ^1H NMR (400 MHz, $CDCl_3$) δ 4.53 – 4.45 (m, 2H), 3.81 – 3.77 (m, 2H), 3.69 – 3.60 (m, 26H), 3.57 – 3.51 (m, 2H), 3.37 (s, 3H); ^{13}C {^1H} NMR (101 MHz, $CDCl_3$) δ 159.7 (t, *J* = 31.3 Hz), 108.8 (t, *J* = 314 Hz), 72.1, 71.0, 70.8, 70.7, 70.7, 70.7, 68.4, 67.3, 59.2; ^{19}F NMR (376 MHz, $CDCl_3$) δ −60.7 (s, 2F); HRMS (ESI) m/z: $[M + Na]^+$ Calcd for $C_{19}H_{35}{}^{79}BrF_2NaO_{10}$ 563.1274; Found 563.1267 (1.2 ppm error).

Hex-5-yn-1-yl 2-bromo-2,2-difluoroacetate (2e)

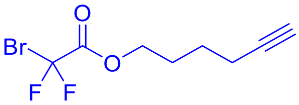

Prepared according to general procedure A using 5-hexyn-1-ol (110 µL, 1.00 mmol, 1.00 eq.), triethylamine (279 µL, 2.00 mmol, 2.00 eq.) and bromodifluoroacetyl chloride (188 µL, 2.00 mmol, 2.00 eq.). The crude material was purified by flash column chromatography (SiO_2, pentane/EtOAc, 9:1) to give the title compound **2e** (158 mg, 0.620 mmol, 62%) as a pale yellow oil; R_f = 0.87 (pentane/EtOAc, 9:1), υ_{max}/cm^{-1} (ATR) 3305, 2939, 2870, 2117, 1773; ^1H NMR (400 MHz, $CDCl_3$) δ 4.39 (t, 2H, *J* = 6.4 Hz), 2.27 (td, 2H, *J* = 6.9, 2.6 Hz), 1.98 (t, 1H, *J* = 2.6 Hz), 1.96 – 1.84 (m, 2H), 1.71 – 1.61 (m, 2H); ^{13}C {^1H} NMR (101 MHz, $CDCl_3$) δ 160.1 (t, *J* = 31.3 Hz), 109.2 (t, *J* = 314 Hz), 83.8, 69.5, 68.3, 27.6, 24.9, 18.4; ^{19}F NMR (376 MHz, $CDCl_3$) δ −60.7 (s, 2F); HRMS (ESI) m/z: [carboxylate fragment]$^-$ Cacld for $^{79}BrCF_2CO_2$ 172.9055; Found 172.9069 (8.1 ppm error).

3.2 Bromodifluoroacetamides

2-Bromo-2,2-difluoro-1-morpholinoethan-1-one (2f)

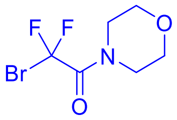

Prepared according to general procedure B using morpholine (78.7 µL, 0.900 mmol, 1.00 eq.), $BrCF_2CO_2Et$ **2a** (139 µL, 1.08 mmol, 1.20 eq.) and $La(OTf)_3$ (26.4 mg, 45.0 µmol, 5 mol%). The crude material was purified by flash column chromatography (SiO_2, pentane/EtOAc, 4:1) to give the title compound **2f** (129 mg, 0.526 mmol, 59%) as a colourless liquid. Characterization data were in accordance with those previously reported.[5]

2-Bromo-2,2-difluoro-*N*-(pyridin-3-ylmethyl)acetamide (2g)

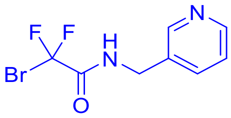

Prepared according to general procedure B using 3-picolylamine (91.6 µL, 0.900 mmol, 1.00 eq.), $BrCF_2CO_2Et$ **2a** (139 µL, 1.08 mmol, 1.20 eq.) and $La(OTf)_3$ (26.4 mg, 45.0 µmol, 5 mol%). The crude material was purified by flash column chromatography (SiO_2, EtOAc) to give the title compound **2g** (80.2 mg, 0.303 mmol, 34%) as a colourless crystalline solid; R_f = 0.34 (EtOAc); mp 73-75 °C; υ_{max}/cm^{-1} (ATR) 3169, 2923, 2851, 2817, 1701, 1559; ^1H NMR (400 MHz, $CDCl_3$) δ 8.50 (dd, 1H, *J* = 4.9, 2.1 Hz), 8.45 (d, 1H, *J* = 2.1 Hz), 7.66 (ddd, 1H, *J* = 7.9, 2.1 Hz), 7.58 (s, 1H), 7.30 (dd, 1H, *J* = 7.9, 4.9 Hz), 4.53 (d, 2H, *J* = 6.0 Hz); ^{13}C NMR {^1H} (101 MHz, $CDCl_3$) δ 160.5 (t, *J* = 27.9 Hz), 149.4, 149.1, 136.0, 132.4, 124.1, 111.7 (t, *J* = 316 Hz), 41.6; ^{19}F NMR (377 MHz, $CDCl_3$) δ −60.6 (s, 2F); HRMS (ESI) m/z: $[M + H]^+$ Calcd for $C_8H_8{}^{79}BrF_2N_2O$ 264.9783; Found 264.9785 (0.8 ppm error).

2-Bromo-*N*-benzyl-2,2-difluoroacetamide (2h)

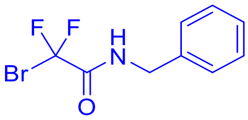

Prepared according to general procedure B using benzylamine (98.3 µL, 0.900 mmol, 1.00 eq.), $BrCF_2CO_2Et$ **2a** (139 µL, 1.08 mmol, 1.20 eq.) and $La(OTf)_3$ (26.4 mg, 45.0 µmol, 5 mol%). The crude material was purified by flash column chromatography (SiO_2, pentane/EtOAc, 9:1) to give the title compound **2h** (199 mg, 0.75 mmol, 84%) as a colourless crystalline solid. Characterization data were in accordance with those previously reported.[6]

2-Bromo-*N*-cyclopropyl-2,2-difluoroacetamide (2i)

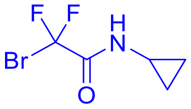

Prepared according to general procedure B using cyclopropylamine (187 µL, 2.70 mmol, 1.00 eq.), $BrCF_2CO_2Et$ **2a** (417 µL, 3.24 mmol, 1.20 eq.) and $La(OTf)_3$ (79.2 mg, 135 µmol, 5 mol%). The crude material was purified by flash column chromatography (SiO_2, pentane/EtOAc, 9:1) to give the title compound **2i** (469 mg, 2.19 mmol, 81%) as a colourless crystalline solid. Characterization data were in accordance with those previously reported.[5]

2-Bromo-*N*-(*tert*-butyl)-2,2-difluoroacetamide (2j)

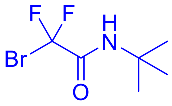

Prepared according to general procedure B using *tert*-butylamine (94.6 µL, 0.900 mmol, 1.00 eq.), $BrCF_2CO_2Et$ **2a** (139 µL, 1.08 mmol, 1.20 eq.) and $La(OTf)_3$ (26.4 mg, 45.0 µmol, 5 mol%). The crude material was purified by flash column chromatography (SiO_2, pentane/EtOAc, 9:1) to give the title compound **2j** (111 mg, 0.483 mmol, 54%) as a colourless crystalline solid. Characterization data were in accordance with those previously reported.[5]

Methyl 5-((3a*S*,4*S*,6a*R*)-2-oxohexahydro-1*H*-thieno[3,4-d]imidazol-4-yl)pentanoate (Biotin-OMe, S1)

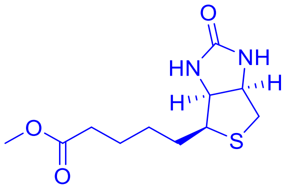

Biotin-OMe **S1** was prepared according to previous reports.[7] To methanol (10 mL) cooled to 0 °C was added acetyl chloride (1.46 mL, 20.5 mmol, 5.01 eq.) dropwise. This was stirred for 5 min before a suspension of biotin (1.0 g, 4.09 mmol, 1.00 eq.) in methanol (10 mL) was added dropwise at 0 °C. The resulting solution was stirred for 1 hour whilst warming to room temperature to give a colourless solution. The reaction mixture was concentrated *in vacuo*, then redissolved in 2% methanol in DCM (50 mL). This solution was extracted with sat. aq. $NaHCO_3$ (3 x 10 mL), water (10 mL) and brine (10 mL), dried over $MgSO_4$ and concentrated *in vacuo* to give the title compound **S1** (594 mg, 2.30 mmol, 56% yield) as a colourless solid. Characterization data were in accordance with those previously reported.[7]

N-(2-aminoethyl)-5-((3aS,4S,6aR)-2-oxohexahydro-1H-thieno[3,4-d]imidazol-4-yl)pentanamide (Biotin-NH_2, S2)

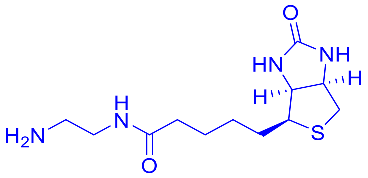

Biotin-NH_2 **S2** was prepared according to previous reports.[7] To Biotin-OMe **S1** (588 mg, 2.28 mmol, 1.00 eq.) in methanol (25 mL) was added ethylenediamine (3.80 mL, 57.0 mmol, 25.0 eq.). The resulting precipitate was stirred at 60 °C (heating mantle) for 16 h. The reaction mixture was concentrated *in vacuo*, with excess ethylenediamine being co-evaporated with toluene (3 x 20 mL). The resulting solid was washed with ethyl acetate (3 x 25 mL) and dried in vacuo to give the title compound **S2** (636 mg, 2.22 mmol, 97% yield) as an off-white solid. Characterization data were in accordance with those previously reported.[7]

N-(2-(2-bromo-2,2-difluoroacetamido)ethyl)-5-((3aS,4S,6aR)-2-oxohexahydro-1H-thieno[3,4-d]imidazol-4-yl)pentanamide (2k)

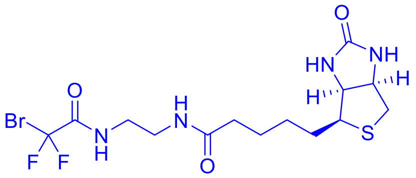

Prepared according to general procedure A using Biotin-NH_2 **S2** (623 mg, 2.18 mmol, 1.00 eq.), bromodifluoroacetyl chloride (410 µL, 4.35 mmol, 2.00 eq.) and triethylamine (606 µL, 4.35 mmol, 2.00 eq.). The crude material was purified by preparative HPLC (2 – 95% B ov. 30 min) to give the title compound **2k** (504 mg, 1.14 mmol, 52%) as a colourless solid; mp = 175-177 °C; υ_{max}/cm^{-1} (ATR) 3289, 3080, 2932, 2863, 1694, 1649, 1550; ^1H NMR (400 MHz, DMSO-d_6) δ 9.16 (t, 1H, *J* = 5.3 Hz), 7.90 (t, 1H, *J* = 5.5 Hz), 6.41 (s, 1H), 6.35 (s, 1H), 4.30 (dd, 1H, *J* = 7.8, 5.0 Hz), 4.16 – 4.08 (m, 1H), 3.24 – 3.13 (m, 4H), 3.13 – 3.02 (m, 1H), 2.82 (dd, 1H, *J* = 12.4, 5.0 Hz), 2.57 (d, 1H, *J* = 12.4 Hz), 2.05 (t, 2H, *J* = 7.4 Hz), 1.70 – 1.21 (m, 6H); ^{13}C NMR {^1H} (101 MHz, DMSO-d_6) δ 173.0, 163.2, 160.2 (t, *J* = 27.1 Hz), 112.4 (t, *J* = 315.1 Hz), 61.5, 59.7, 55.9, 40.3, 37.9, 35.7, 28.7, 28.5, 25.6; ^{19}F NMR (376 MHz, DMSO-d_6) δ −60.0 (s, 2F), −74.9 (s, TFA); HRMS (ESI) m/z: $[M + H]^+$ Calcd for $C_{14}H_{22}{}^{79}BrF_2N_4O_3S$ 443.0564; Found 443.0567 (0.7 ppm error).

***N*-((2*R*,3*R*,4*R*,5*S*,6*R*)-3-acetamido-4,5-dihydroxy-6-(hydroxymethyl)tetrahydro-2*H*-pyran-2-yl)-2-bromo-2,2-difluoroacetamide (2l)**

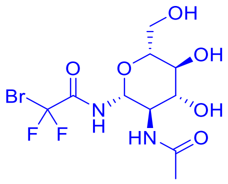

Prepared according to general procedure A using 2-acetamido-2-deoxy-beta-D-glucopyranosylamine (220 mg, 1.00 mmol, 1.00 eq.), triethylamine (140 µL, 1.00 mmol, 1.00 eq.) and bromodifluoroacetyl chloride (188 µL, 2.00 mmol, 2.00 eq.). The crude material was purified by preparative HPLC (2 – 95% B ov. 30 min then 2 – 70% B ov. 30 min) to give the title compound **2l** (23.6 mg, 0.063 mmol, 6%) as a colourless solid; mp = 208 - 210 °C (decomposed); υ_{max}/cm^{-1} (ATR) 3361, 3281, 3080, 2953, 2928, 2873, 2852, 1213, 1659, 1539; ^{1}H NMR (400 MHz, DMSO-d_6) δ 9.50 (d, 1H, *J* = 8.4 Hz), 7.90 (d, 1H, *J* = 8.5 Hz), 5.09 – 5.03 (m, 2H), 4.86 (t, 1H, *J* = 9.0 Hz), 4.71 – 4.49 (m, 1H), 3.73 – 3.59 (m, 2H), 3.49 – 3.35 (m, 2H), 3.23 – 3.14 (m, 1H), 3.13 – 3.04 (m, 1H), 1.80 (s, 3H); ^{13}C NMR {^{1}H} (126 MHz, DMSO-d_6) δ 170.2, 159.7 (t, *J* = 27.9 Hz), 111.3 (t, *J* = 314.3 Hz), 79.8, 79.3, 74.0, 70.3, 60.8, 54.4, 22.8; ^{19}F NMR (376 MHz, DMSO-d_6) δ −61.1 (d, 1F, *J* = 158.0 Hz), −61.5 (d, 1F, *J* = 158.0 Hz); HRMS (ESI) m/z: $[M + Na]^+$ Calcd for $C_{10}H_{15}{}^{79}BrF_2N_2O_6Na$ 398.9979; Found 398.9975 (1.00 ppm error).

4. Peptide synthesis

Ac-WHISKEY-NH_2 (4)

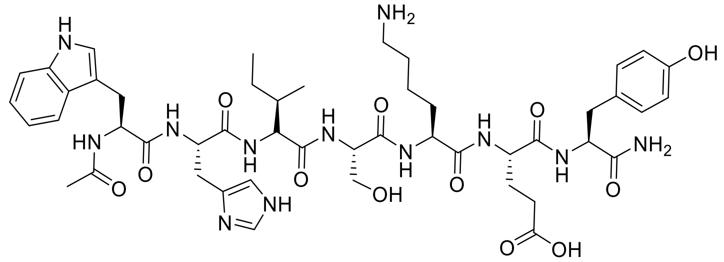

Prepared via SPPS using rink amide resin (250 µmol). The crude peptide was cleaved from the resin using mixture of TFA, TIPS and water (90:5:5 v/v/v) and purified by preparative RP-HPLC (2-95% B over 30 min) to give the title compound **4** (50.0 mg, 49.9 µmol, 20%) as a colourless powder; ^{1}H NMR (400 MHz, CD_3OD) δ 8.70 (d, 1H, *J* = 1.4 Hz), 7.51 (d, 1H, *J* = 7.9 Hz), 7.32 (d, 1H, *J* = 8.1 Hz), 7.22 (d, 1H, *J* = 1.4 Hz), 7.14 (s, 1H), 7.08 (dd, 1H, *J* = 8.1, 7.6 Hz), 7.03 – 6.94 (m, 3H), 6.67 (d, 2H, *J* = 8.4 Hz), 4.65 (dd, 1H, *J* = 7.2, 5.5 Hz), 4.56 (dd, 1H, *J* = 8.2, 6.1 Hz), 4.51 – 4.42 (m, 2H), 4.42 – 4.29 (m, 2H), 4.23 (d, 1H, *J* = 7.4 Hz), 3.88 (dd, 1H, *J* = 10.9, 5.5 Hz), 3.79 (dd, 1H, *J* = 10.9, 5.9 Hz), 3.30 – 2.95 (m, 5H), 2.91 – 2.81 (m, 2H), 2.77 – 2.67 (m, 1H), 2.40 – 2.22 (m, 2H), 2.07 – 1.95 (m, 1H), 1.94 (s, 3H), 1.92 – 1.82 (m, 3H), 1.79 – 1.67 (m, 1H), 1.67 – 1.57 (m, 2H), 1.57 – 1.49 (m, 1H), 1.49 – 1.36 (m, 2H), 1.26 – 1.12 (m, 1H), 0.96 (d, 3H, J = 6.8 Hz), 0.91 (t, 3H, *J* = 7.4 Hz); HRMS (ESI) m/z: $[M + H]^+$ Calcd for $C_{48}H_{67}N_{12}O_{12}$ 1003.4996; Found 1003.5022 (2.6 ppm error).

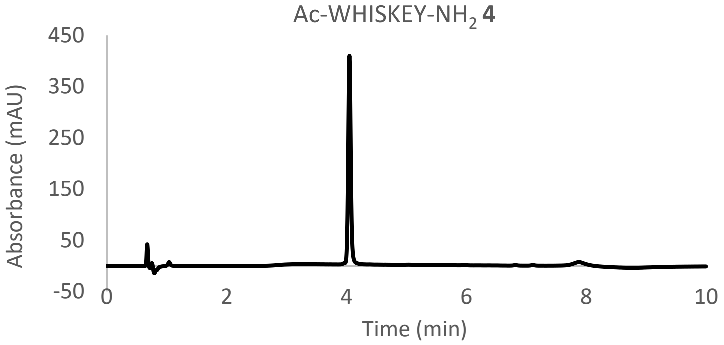

Figure S4. Analytical HPLC chromatogram for purified Ac-WHISKEY-NH_2 **4** Rt = 4.04 min on analytical gradient 2-95% B over 5 min, 0.6 mL min^{-1}, 280 nm.

Ac-HISKEY-NH$_2$ (6)

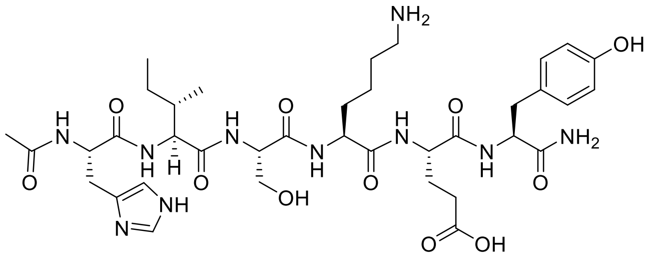

Prepared via SPPS using rink amide resin (250 µmol). The crude peptide was cleaved from the resin using mixture of TFA, TIPS and water (90:5:5 v/v/v) and purified by preparative RP-HPLC (2-70% B over 30 min) to give the title compound **6** (72.4 mg, 88.6 µmol, 44%) as a colourless powder; ^{1}H NMR (400 MHz, CD_3OD) δ 8.77 (d, 1H, *J* = 1.4 Hz), 7.33 (d, 1H, *J* = 1.4 Hz), 7.07 (d, 2H, *J* = 8.5 Hz), 6.69 (d, 2H, *J* = 8.6 Hz), 4.73 (dd, 1H, *J* = 7.5, 6.9 Hz), 4.51 (dd, 1H, *J* = 8.5, 5.6 Hz), 4.45 (dd, 1H, *J* = 6.1, 5.7 Hz) 4.33 – 4.25 (m, 2H), 4.22 (d, 1H, *J* = 7.4 Hz), 3.89 (dd, 1H, *J* = 10.9, 5.7 Hz), 3.79 (dd, 1H, *J* = 10.9, 6.1 Hz), 3.24 (dd, 1H, *J* = 15.3, 6.7 Hz), 3.13 – 3.02 (m, 2H), 2.92 (dd, *J* = 8.5, 7.5 Hz, 2H), 2.86 (dd, 1H, *J* = 14.0, 8.6 Hz), 2.41 – 2.23 (m, 2H), 2.07 – 1.99 (m, 1H), 1.98 (s, 3H), 1.94 – 1.80 (m, 3H), 1.80 – 1.63 (m, 3H), 1.62 – 1.40 (m, 3H), 1.28 – 1.12 (m, 1H), 0.95 (d, 3H, *J* = 6.8 Hz), 0.91 (t, 3H, *J* = 7.4 Hz); HRMS (ESI) m/z: [M + H]$^+$ Calcd for $C_{37}H_{57}N_{10}O_{11}$ 817.4208; Found 817.4168 (4.9 ppm error).

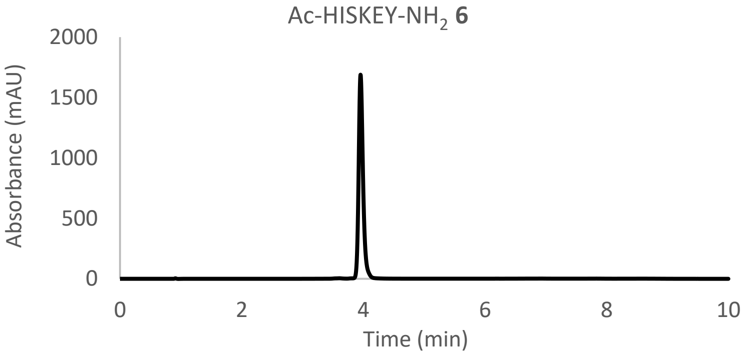

Figure S5. Analytical HPLC chromatogram for purified Ac-HISKEY-NH$_2$ **6** Rt = 3.95 min on analytical gradient 2-70% B over 5 min, 0.6 mL min^{-1}, 280 nm.

H-WHISKEY-OH (7)

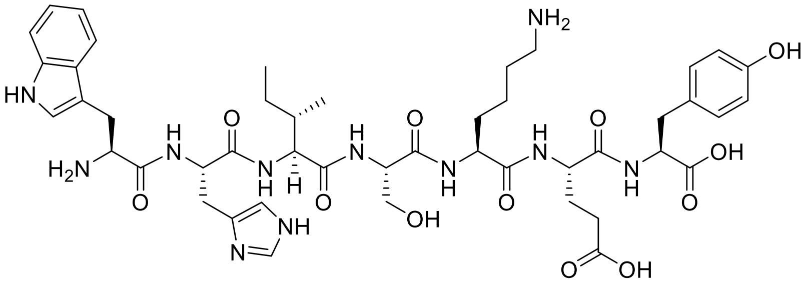

Prepared via SPPS using 2-CTC resin (250 µmol). The crude peptide was cleaved from the resin using mixture of TFA, TIPS and water (90:5:5 v/v/v) and purified by preparative RP-HPLC (10-80% B over 30 min) to give the title compound **7** (74.6 mg, 77.5 µmol, 31%) as a colourless powder; ^{1}H NMR (400 MHz, CD_3OD) δ 8.60 (s, 1H), 7.63 (d, 1H, *J* = 7.9 Hz), 7.38 (d, 1H, *J* = 8.1 Hz), 7.27 (s, 1H), 7.20 (s, 1H), 7.14 (dd, 1H, *J* = 8.1, 7.3 Hz), 7.10 – 7.01 (m, 3H), 6.77 – 6.65 (m, 2H), 4.75 (dd, 1H, *J* = 7.4, 6.5 Hz), 4.54 – 4.44 (m, 2H), 4.34 – 4.26 (m, 2H), 4.25 – 4.17 (m, 2H), 3.88 (dd, 1H, *J* = 10.9, 5.6 Hz), 3.81 (dd, 1H, *J* = 10.9, 6.0 Hz), 3.41 (dd, 1H, *J* = 15.0, 5.6 Hz), 3.29 – 2.99 (m, 4H), 2.94 – 2.81 (m, 3H), 2.42 – 2.24 (m, 2H), 2.10 – 1.97 (m, 1H), 1.96 – 1.81 (m, 3H), 1.80 – 1.61 (m, 3H), 1.61 – 1.51 (m, 1H), 1.51 – 1.38 (m, 2H), 1.31 – 1.14 (m, 1H), 0.96 (d, 3H, *J* = 6.9 Hz), 0.93 (t, 3H, *J* = 7.5 Hz); HRMS (ESI) m/z: $[M + H]^+$ Calcd for $C_{46}H_{64}N_{11}O_{12}$ 962.4736; Found 962.4805 (7.2 ppm error).

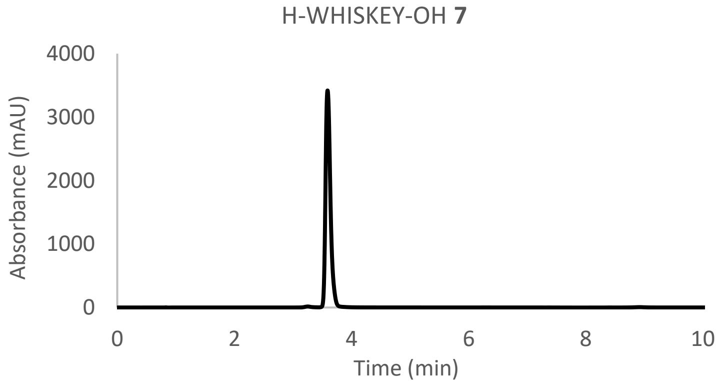

Figure S6. Analytical HPLC chromatogram for purified H-WHISKEY-OH **7** Rt = 3.59 min on analytical gradient 10-80% B over 5 min, 0.6 mL min^{-1}, 280 nm.

Ac-CWHISKEY-NH$_2$ (8)

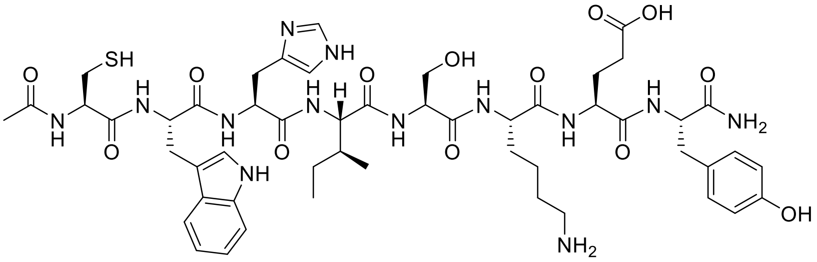

Prepared via SPPS using rink amide resin (250 µmol). The crude peptide was cleaved from the resin using mixture of TFA, TIPS, thioanisole and water (85:5:5:5 v/v/v) and purified by preparative RP-HPLC (2-95% B over 30 min) to give the title compound **8** (48.6 mg, 43.9 µmol, 18%) as a colourless powder; ^{1}H NMR (400 MHz, CD_3OD) δ 8.72 (s, 1H), 7.52 (d, 1H, *J* = 7.7 Hz), 7.35 (d, 1H, *J* = 8.1 Hz), 7.17 (s, 1H), 7.14 (s, 1H), 7.13 – 7.04 (m, 3H), 7.01 (dd, 1H, *J* = 7.7, 7.3 Hz), 6.69 (d, 2H, *J* = 8.1 Hz), 4.61 (dd, 2H, J = 8.1, 6.6 Hz), 4.50 (dd, 1H, *J* = 8.8, 5.5 Hz), 4.42 (t, 1H, J = 5.7 Hz), 4.36 – 4.22 (m, 3H), 4.15 (d, 1H, *J* = 7.7 Hz), 3.91 (dd, 1H, *J* = 11.0, 5.7 Hz), 3.82 (dd, 1H, *J* = 11.0, 5.7 Hz), 3.27 – 3.15 (m, 3H), 3.12 – 2.98 (m, 2H), 2.97 – 2.67 (m, 5H), 2.39 – 2.26 (m, 2H), 2.07 – 1.80 (m, 7H), 1.80 – 1.71 (m, 1H), 1.71 – 1.59 (m, 2H), 1.59 – 1.51 (m, 1H), 1.50 – 1.39 (m, 2H), 1.24 – 1.16 (m, 1H), 0.96 (d, 3H, *J* = 6.8 Hz), 0.91 (t, 3H, *J* = 7.4 Hz); HRMS (ESI) m/z: $[M + H]^+$ Calcd for $C_{51}H_{71}N_{12}O_{14}S$ 1107.4933; Found 1107.4940 (0.6 ppm error).

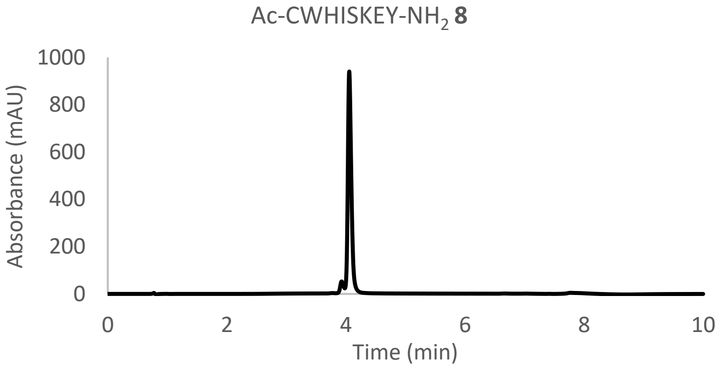

Figure S7. Analytical HPLC chromatogram for purified Ac-CWHISKEY-NH$_2$ **8** Rt = 4.06 min on analytical gradient 10-70% B over 5 min, 0.6 mL min^{-1}, 280 nm.

Ac-WFMTREY-NH$_2$ (9)

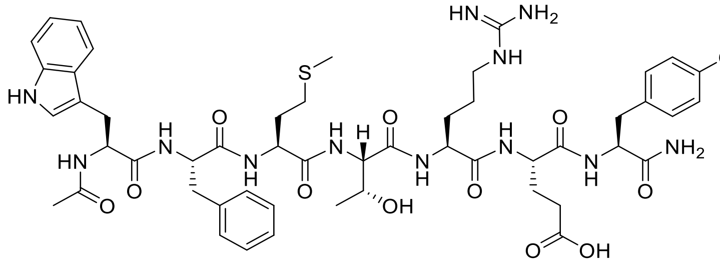

Prepared via SPPS using rink amide resin (250 µmol). The crude peptide was cleaved from the resin using mixture of TFA, TIPS, thioanisole and water (85:5:5:5 v/v/v) and purified by preparative RP-HPLC (20-80% B over 30 min then 2-95% B over 30 min) to give the title compound **9** (19.6 mg, 18.3 µmol, 7%) as a colourless powder; ^{1}H NMR (400 MHz, CD_3OD) δ 7.49 (d, 1H, *J* = 7.9 Hz), 7.36 (d, 1H, *J* = 8.2 Hz), 7.32 – 7.20 (m, 3H), 7.18 – 7.09 (m, 2H), 7.08 (d, 2H, *J* = 8.5 Hz), 7.06 – 6.98 (m, 3H), 6.69 (d, 2H, *J* = 8.5 Hz), 4.56 – 4.37 (m, 3H), 4.35 – 4.29 (m, 1H), 4.29 – 4.18 (m, 4H), 3.19 – 3.11 (m, 4H), 3.11 – 3.03 (m, 1H), 2.93 (dd, 1H, *J* = 14.0, 7.3 Hz), 2.86 (dd, 1H, *J* = 14.1, 9.0 Hz), 2.75 (dd, 1H, *J* = 14.0, 5.8 Hz), 2.43 – 2.26 (m, 3H), 2.16 – 2.08 (m, 1H), 2.07 (s, 3H), 2.03 – 1.88 (m, 3H), 1.87 (s, 3H), 1.85 – 1.67 (m, 2H), 1.67 – 1.56 (m, 2H), 1.24 (d, 3H, *J* = 6.1 Hz); HRMS (ESI) m/z: $[M + H]^+$ Calcd for $C_{51}H_{69}N_{12}O_{12}S$ 1073.4879; Found 1073.4798 (7.5 ppm error).

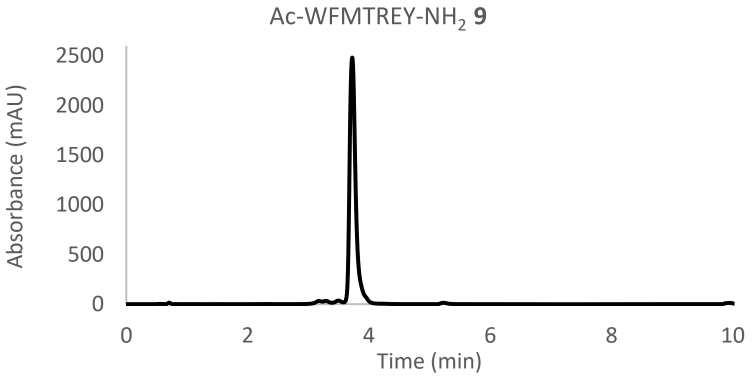

Figure S8. Analytical HPLC chromatogram for purified Ac-WFMTREY-NH$_2$ **9** Rt = 3.73 min on analytical gradient 20-100% B over 5 min, 0.6 mL min^{-1}, 280 nm.

Ac-WLAHKAL-NH_2 (10)

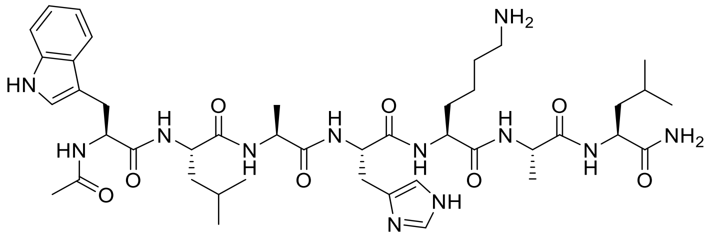

Prepared via SPPS using rink amide resin (250 µmol). The crude peptide was cleaved from the resin using mixture of TFA, TIPS and water (90:5:5 v/v/v) and purified by preparative RP-HPLC (20-80% B over 30 min) to give the title compound **10** (61.6 mg, 70.1 µmol, 28%) as a colourless powder; ^{1}H NMR (400 MHz, CD_3OD) δ 8.73 (d, 1H, *J* = 1.4 Hz), 7.53 (ddd, 1H, *J* = 7.9, 1.2, 1.0 Hz), 7.38 – 7.31 (m, 2H), 7.18 (s, 1H), 7.10 (ddd, 1H, *J* = 8.2, 7.0, 1.2 Hz), 6.99 (ddd, 1H, *J* = 7.9, 7.0, 1.0 Hz), 4.63 – 4.45 (m, 2H), 4.44 – 4.22 (m, 3H), 4.21 – 4.07 (m, 2H), 3.31 – 3.12 (m, 4H), 2.93 (dd, 2H, *J* = 8.8, 7.4 Hz), 2.01 (s, 3H), 1.95 – 1.80 (m, 1H), 1.80 – 1.55 (m, 6H), 1.55 – 1.42 (m, 5H), 1.39 (d, 3H, *J* = 7.2 Hz), 1.34 (d, 3H, *J* = 7.2 Hz), 0.96 – 0.87 (m, 9H), 0.82 (d, 3H, *J* = 6.1 Hz); HRMS (ESI) m/z: $[M + H]^+$ Calcd for $C_{43}H_{67}N_{12}O_8$ 879.5205; Found 879.5190 (1.7 ppm error).

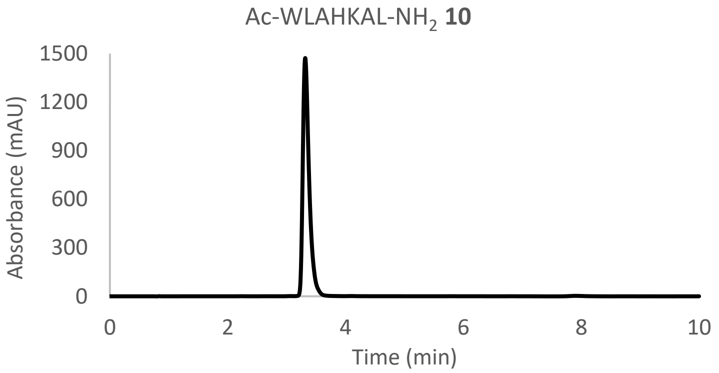

Figure S9. Analytical HPLC chromatogram for purified Ac-WLAHKAL-NH_2 **10** Rt = 3.32 min on analytical gradient 20-80% B over 5 min, 0.6 mL min^{-1}, 280 nm.

Ac-VVYPWYQ-NH₂ (11)

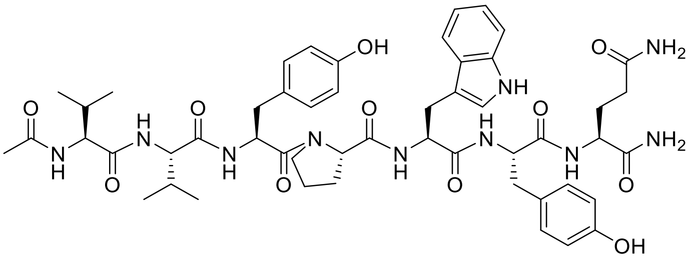

Prepared via SPPS using rink amide resin (250 µmol). The crude peptide was cleaved from the resin using mixture of TFA, TIPS and water (90:5:5 v/v/v) and purified by preparative RP-HPLC (20-70% B over 30 min) to give the title compound **11** (26 mg, 26.1 µmol, 14%) as a colourless powder; ^{1}H NMR (400 MHz, DMSO-d_6) δ 10.78 (d, 1H, *J* = 2.4 Hz), 9.13 (d, 2H, *J* = 13.7 Hz), 8.05 (d, 1H, *J* = 8.0 Hz), 7.96 – 7.82 (m, 3H), 7.70 (d, 1H, *J* = 7.7 Hz), 7.62 (d, 1H, *J* = 9.1 Hz), 7.51 (d, 1H, *J* = 7.8 Hz), 7.30 (d, 1H, *J* = 8.1 Hz), 7.25 (s, 1H), 7.14 – 7.05 (m, 3H), 7.05 – 7.00 (m, 3H), 6.99 – 6.92 (m, 3H), 6.78 (s, 1H), 6.67 – 6.54 (m, 4H), 4.64 – 4.53 (m, 1H), 4.49 – 4.33 (m, 2H), 4.30 – 4.23 (m, 1H), 4.20 – 4.06 (m, 3H), 3.59 – 3.52 (m, 1H), 3.38 – 3.33 (m, 1H), 3.17 – 3.01 (m, 1H), 3.01 – 2.90 (m, 1H), 2.88 – 2.59 (m, 4H), 2.18 – 2.02 (m, 2H), 1.97 – 1.79 (m, 7H), 1.79 – 1.60 (m, 4H), 0.86 – 0.68 (m, 12H); HRMS (ESI) m/z: [M + Na]$^+$ Calcd for $C_{51}H_{66}N_{10}O_{11}Na$ 1017.4810; Found 1017.4774 (3.5 ppm error).

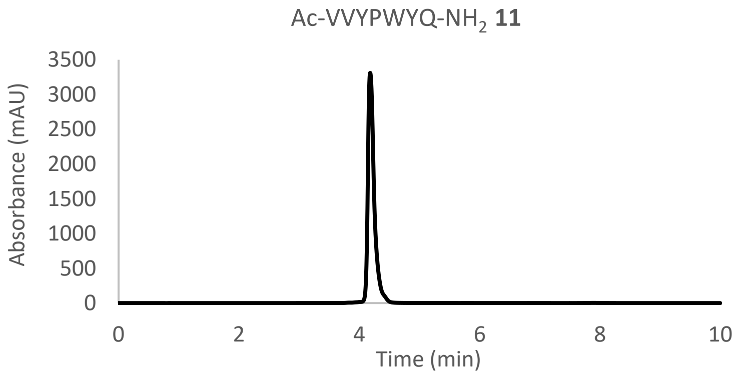

Figure S10. Analytical HPLC chromatogram for purified Ac-VVYPWYQ-NH₂ **11** Rt = 4.18 min on analytical gradient 20-80% B over 5 min, 0.6 mL min^{-1}, 280 nm.

Ac-DKVGINYW-NH$_2$ (12)

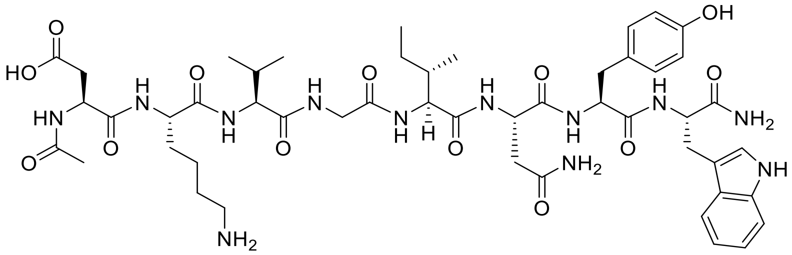

Prepared via SPPS using rink amide resin (250 µmol). The crude peptide was cleaved from the resin using mixture of TFA, TIPS and water (90:5:5 v/v/v) and purified by preparative RP-HPLC (20-80% B over 30 min then 2-70% B over 30 min) to give the title compound **12** (35.3 mg, 34.1 µmol, 14%) as a colourless powder; ^{1}H NMR (400 MHz, CD_3OD) δ 7.63 (dd, 1H, *J* = 7.8, 0.9 Hz), 7.34 (dd, 1H, *J* = 8.1, 0.9 Hz), 7.16 (s, 1H), 7.11 (ddd, 1H, *J* = 8.1, 7.3, 0.9 Hz), 7.04 (ddd, 1H, *J* = 7.8, 7.4, 0.9 Hz), 6.78 (d, 2H, *J* = 8.4 Hz), 6.56 (d, 2H, *J* = 8.4 Hz), 4.68 – 4.60 (m, 2H), 4.57 (dd, 1H, *J* = 9.7, 4.7 Hz), 4.39 (dd, 1H, *J* = 9.2, 5.2 Hz), 4.24 (dd, 1H, *J* = 7.8, 6.6 Hz), 4.13 (dd, 2H, *J* = 10.0, 7.2 Hz), 3.99 (d, 1H, *J* = 16.4 Hz, 1H), 3.76 (d, 1H, *J* = 16.4 Hz), 3.37 (dd, 1H, *J* = 14.7, 4.5 Hz), 3.22 – 3.14 (m, 1H), 2.96 – 2.83 (m, 3H), 2.82 – 2.62 (m, 6H), 2.18 – 2.06 (m, 1H), 1.98 (s, 3H), 1.92 – 1.80 (m, 2H), 1.74 – 1.55 (m, 2H), 1.52 – 1.37 (m, 2H), 1.25 – 1.11 (m, 1H), 1.00 – 0.93 (m, 7H), 0.89 (t, 3H, *J* = 7.5 Hz), 0.85 (d, 3H, *J* = 6.9 Hz); HRMS (ESI) m/z: $[M + H]^+$ Calcd for $C_{49}H_{71}N_{12}O_{13}$ 1035.5264; Found 1035.5168 (9.3 ppm error).

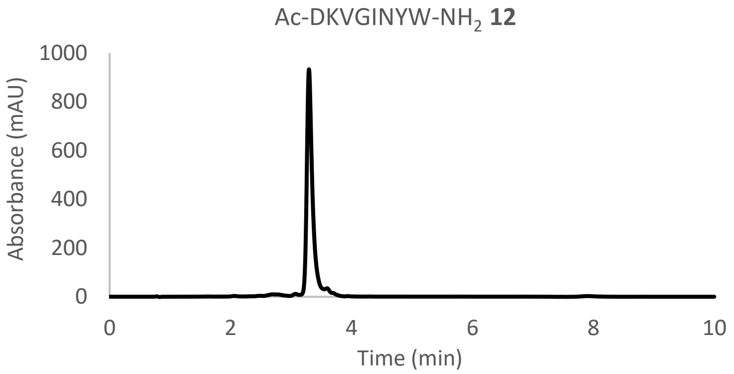

Figure S11. Analytical HPLC chromatogram for purified Ac-DKVGINYW-NH$_2$ **12** Rt = 3.30 min on analytical gradient 20-80% B over 5 min, 0.6 mL min^{-1}, 280 nm.

5. Photochemical alkylation of Ac-Trp-OEt

General Procedure C: Analytical scale photocatalytic difluoroalkylation of Ac-Trp-OEt (1)

A solution of Ac-Trp-OEt **1** (0.1 M in DMSO, 200 µL, 20.0 µmol, 1.00 eq.), 4CzIPN (0.01 M in DMSO, 20 µL, 0.200 µmol, 1 mol%) and bromodifluoroalkyl reagent (0.1 M in DMSO, 400 µL, 40.0 µmol, 2.00 eq.) in DMSO (1.380 mL, total solvent volume 2 mL) was purged with N_2 (5 min) and then irradiated with blue LEDs (PhotoRedOx Box) for 10 – 150 min at room temperature. α,α,α-trifluorotoluene (0.0165 M in DMSO, 404 µL, 6.67 µmol, 0.333 equiv.) was added, then the reaction mixture was analysed by quantitative NMR spectroscopy, analytical HPLC, and mass spectrometry.

General procedure D: Scale-up of the photocatalytic difluoroalkylation of Ac-Trp-OEt (1)

A solution of Ac-Trp-OEt **1** (54.9 mg, 200 µmol, 1.00 eq.), 4CzIPN (1.6 mg, 2.00 µmol, 1 mol%) and bromodifluoroalkyl reagent (400 µmol, 2.00 eq.) in DMSO (20 mL, 0.01 M) was purged with N_2 (5 min) and then irradiated with blue LEDs (PhotoRedOx Box) for 10 - 30 min at room temperature. α,α,α-trifluorotoluene (8.20 µL, 66.7 µmol, 0.333 eq.) was added to the reaction mixture, then quantitative NMR spectroscopy and analytical HPLC analysis were carried out. The reaction mixture was diluted with water (10 mL) and extracted with ethyl acetate (3 × 10 mL). The combined organic extracts were washed with brine (30 mL), dried over $MgSO_4$ and concentrated *in vacuo*. The resulting residue was purified by flash column chromatography.

Ethyl (*S*)-2-acetamido-3-(2-(2-ethoxy-1,1-difluoro-2-oxoethyl)-1*H*-indol-3-yl)propanoate (3a) - analytical scale

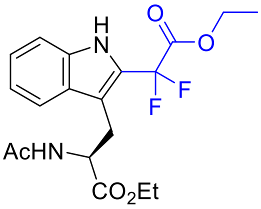

Prepared according to general procedure C using Ac-Trp-OEt **1** (0.1 M in DMSO, 200 µL, 20.0 µmol, 1.00 eq.), 4CzIPN (0.01 M in DMSO, 20 µL, 0.200 µmol, 1 mol%) and ethyl bromodifluoroacetate **2a** (0.1 M in DMSO, 400 µL, 40.0 µmol, 2.00 eq.). The reaction mixture was irradiated with blue LEDs for 15 min then the yield was determined by quantitative ^{19}F NMR spectroscopy (79% yield). ^{19}F NMR (376 MHz, DMSO) δ −99.0 (d, 1F, *J* = 264 Hz), −99.7 (d, 1F, *J* = 264 Hz); HRMS (ESI) m/z: $[M + H]^+$ Calcd for $C_{19}H_{23}F_2N_2O_5$ 397.1567; Found 397.1548 (4.0 ppm error).

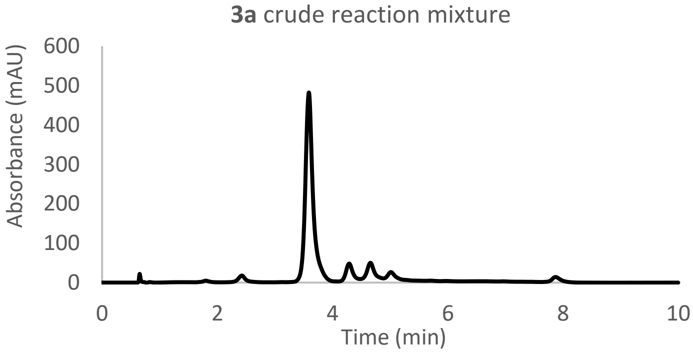

Figure S12. Reaction mixture analytical HPLC chromatogram for product **3a** Rt = 3.59 min on analytical gradient 40-80% B over 5 min, 0.6 mL min^{-1}, 280 nm.

Ethyl (*S*)-2-acetamido-3-(2-(2-ethoxy-1,1-difluoro-2-oxoethyl)-1*H*-indol-3-yl)propanoate (3a)

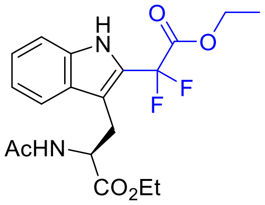

Prepared according to general procedure D using Ac-Trp-OEt **1** (54.9 mg, 200 µmol, 1.00 eq.), 4CzIPN (1.6 mg, 2.00 µmol, 1 mol%) and ethyl bromodifluoroacetate **2a** (51.3 µL, 400 µmol, 2.00 eq.). The reaction mixture was irradiated with blue LEDs for 10 min then was analysed by quantitative ^{19}F NMR spectroscopy (65% yield) and analytical HPLC. The crude material was purified by flash column chromatography (SiO_2, pentane/EtOAc, 1:1) to give the title compound **3a** (51.5 mg, 0.130 mmol, 65%) as an off white crystalline solid; R_f = 0.31 (pentane/EtOAc, 1:1); mp = 33-35 °C; υ_{max}/cm^{-1} (ATR) 2984, 2921, 2870, 2851, 1761, 1736, 1656, 1526, 1446, 1372; ^{1}H NMR (400 MHz, $CDCl_3$) δ 8.77 (s, 1H), 7.70 (d, 1H, *J* = 8.0 Hz), 7.37 (d, 1H, *J* = 8.2 Hz), 7.28 (ddd, 1H, *J* = 8.2, 7.0, 0.8 Hz), 7.17 (ddd, 1H, *J* = 8.0, 7.0, 1.0 Hz), 6.20 (d, 1H, *J* = 7.6 Hz), 4.90 (ddd, 1H, *J* = 7.6, 7.4, 6.0 Hz), 4.34 (q, 2H, *J* = 7.2 Hz), 4.21 – 4.01 (m, 2H), 3.45 – 3.30 (m, 2H), 1.92 (s, 3H), 1.34 (t, 3H, *J* = 7.2 Hz), 1.17 (t, 3H, *J* = 7.4 Hz); ^{13}C {^{1}H} NMR (101 MHz, $CDCl_3$) δ 172.0, 170.1, 163.6 (t, *J* = 35.5 Hz), 135.9, 127.9, 124.8, 124.6 (t, *J* = 30.1 Hz), 120.8, 120.0, 112.6 (t, *J* = 2.7 Hz), 111.91, 111.4 (t, *J* = 252 Hz), 64.0, 61.6, 53.0, 27.1, 23.1, 14.0, 14.0; ^{19}F NMR (377 MHz, $CDCl_3$) δ −99.6 (d, 1F, *J* = 267 Hz), −101.3 (d, 1F, *J* = 267 Hz); HRMS (ESI) m/z: [M + Na]$^+$ Calcd for $C_{19}H_{22}F_2N_2NaO_5$ 419.1389; Found 419.1408 (4.5 ppm error)].

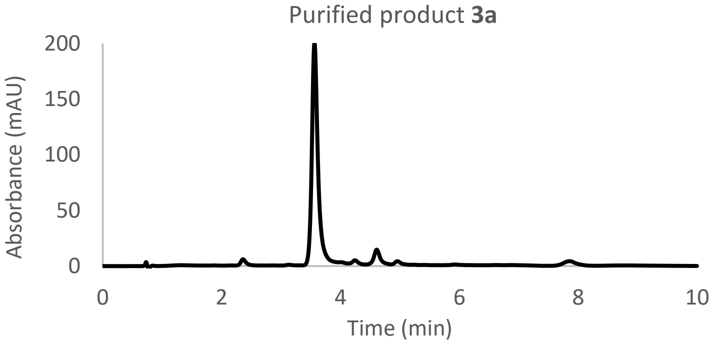

Figure S13. Analytical HPLC chromatogram for purified product **3a** Rt = 3.56 min on analytical gradient 40-80% B over 5 min, 0.6 mL min^{-1}, 280 nm.

Ethyl (*S*)-2-acetamido-3-(2-(2-(benzyloxy)-1,1-difluoro-2-oxoethyl)-1*H*-indol-3-yl)propanoate (3b) - analytical scale

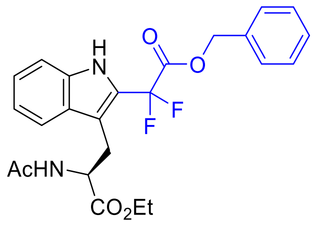

Prepared according to general procedure C using Ac-Trp-OEt **1** (0.1 M in DMSO, 200 µL, 20.0 µmol, 1.00 eq.), 4CzIPN (0.01 M in DMSO, 20 µL, 0.200 µmol, 1 mol%) and benzyl 2-bromo-2,2-difluoroacetate **2b** (0.1 M in DMSO, 400 µL, 40.0 µmol, 2.00 eq.). The reaction mixture was irradiated with blue LEDs for 10 min then the yield was determined by quantitative ^{19}F NMR spectroscopy (66% yield); ^{19}F NMR (376 MHz, DMSO) δ −98.8 (d, 1F, *J* = 263 Hz), −99.6 (d, 1F, *J* = 263 Hz); HRMS (ESI) m/z: $[M + H]^+$ Calcd for $C_{24}H_{25}F_2N_2O_5$ 459.1732; Found 459.1730 (0.4 ppm error).

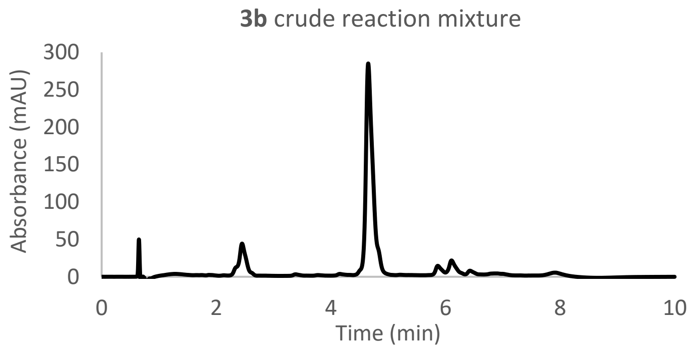

Figure S14. Reaction mixture analytical HPLC chromatogram for product **3b** Rt = 4.65 min on analytical gradient 40-80% B over 5 min, 0.6 mL min^{-1}, 280 nm.

Ethyl (*S*)-2-acetamido-3-(2-(2-(benzyloxy)-1,1-difluoro-2-oxoethyl)-1*H*-indol-3-yl)propanoate (3b)

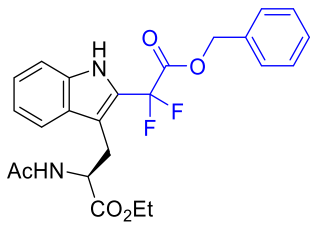

Prepared according to general procedure D using Ac-Trp-OEt **1** (54.9 mg, 200 µmol, 1.00 eq.), 4CzIPN (1.6 mg, 2.00 µmol, 1 mol%) and benzyl 2-bromo-2,2-difluoroacetate **2b** (106 mg, 400 µmol, 2.00 eq.). The reaction mixture was irradiated with blue LEDs for 10 min then was analysed by quantitative ^{19}F NMR spectroscopy (70% yield) and analytical HPLC. The crude material was purified by flash column chromatography (SiO_2, pentane/EtOAc, 6:4) to give the title compound **3b** (59.5 mg, 0.130 mmol, 65%) as an off white crystalline solid; R_f = 0.28 (pentant/EtOAc, 6:4); mp = 40-42 °C; υ_{max}/cm^{-1} (ATR) 3065, 3037, 2955, 2918, 2849, 1763, 1734, 1657; ^{1}H NMR (400 MHz, $CDCl_3$) δ 7.69 (d, 1H, *J* = 8.1 Hz), 7.37 – 7.20 (m, 7H), 7.16 (ddd, 1H, *J* = 8.1, 6.9, 1.2 Hz), 6.21 (d, 1H, *J* = 7.9 Hz), 5.28 (s, 2H), 4.90 (ddd, 1H, *J* = 7.9, 7.6, 6.1 Hz), 4.18 – 3.99 (m, 2H), 3.38 (dd 1H, *J* = 14.6, 6.1 Hz), 3.30 (dd, 1H, *J* = 14.6, 7.6 Hz), 1.87 (s, 3H), 1.15 (t, 3H, *J* = 7.1 Hz); ^{13}C {^{1}H} NMR (101 MHz, $CDCl_3$) δ 172.0, 170.2, 163.4 (t, *J* = 35.9 Hz), 136.0, 133.9, 129.1, 128.9, 128.5, 127.8, 124.7, 124.2 (t, *J* = 29.8 Hz), 120.7, 119.9, 112.6 (t, *J* = 2.80 Hz), 112.0, 111.5 (t, *J* = 252 Hz), 69.2, 61.6, 53.0, 27.0, 23.0, 14.0; ^{19}F NMR (376 MHz, $CDCl_3$) δ −99.6 (d, 1F, *J* = 267 Hz), −101.1 (d, 1F, *J* = 267 Hz); HRMS (ESI) m/z: $[M + Na]^+$ Calcd for $C_{24}H_{24}F_2N_2NaO_5$ 481.1545; Found 481.1548 (0.6 ppm error).

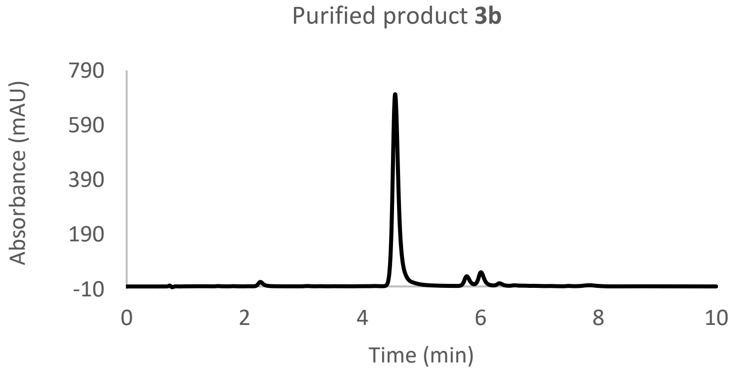

Figure S15. Analytical HPLC chromatogram for purified product **3b** Rt = 4.55 min on analytical gradient 40-80% B over 5 min, 0.6 mL min^{-1}, 280 nm.

Ethyl (*S*)-2-acetamido-3-(2-(2-((4-bromobenzyl)oxy)-1,1-difluoro-2-oxoethyl)-1*H*-indol-3-yl)propanoate (3c) - analytical scale

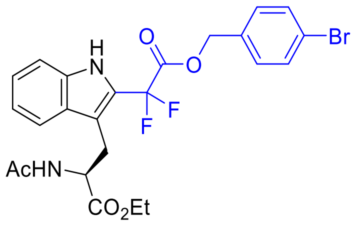

Prepared according to general procedure C using Ac-Trp-OEt **1** (0.1 M in DMSO, 200 µL, 20.0 µmol, 1.00 eq.), 4CzIPN (0.01 M in DMSO, 20 µL, 0.200 µmol, 1 mol%) and 4-bromobenzyl 2-bromo-2,2-difluoroacetate **2c** (0.1 M in DMSO, 400 µL, 40.0 µmol, 2.00 eq.). The reaction mixture was irradiated with blue LEDs for 10 min then the yield was determined by quantitative ^{19}F NMR spectroscopy (62% yield); ^{19}F NMR (376 MHz, DMSO) δ −98.0 (d, 1F, *J* = 264 Hz), −99.0 (d, 1F, *J* = 264 Hz); HRMS (ESI) m/z: [M + H]$^+$ Calcd for $C_{24}H_{24}{}^{79}BrF_2N_2O_5$ 537.0831; Found 537.0817 (2.7 ppm error).

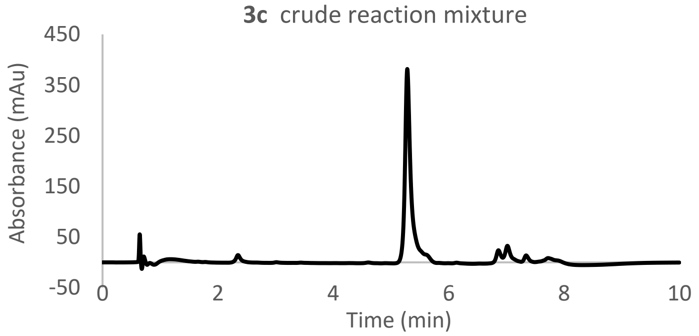

Figure S16. Reaction mixture analytical HPLC chromatogram for product **3c** Rt = 5.28 min on analytical gradient 40-80% B over 5 min, 0.6 mL min^{-1}, 280 nm.

Ethyl (*S*)-2-acetamido-3-(2-(28,28-difluoro-27-oxo-2,5,8,11,14,17,20,23,26-nonaoxaoctacosan-28-yl)-1*H*-indol-3-yl)propanoate (3d) - analytical scale

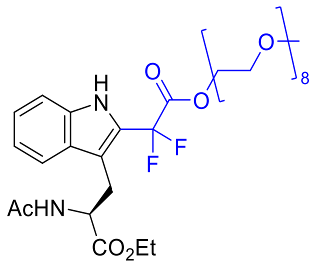

Prepared according to general procedure C using Ac-Trp-OEt **1** (0.1 M in DMSO, 200 µL, 20.0 µmol, 1.00 eq.), 4CzIPN (0.01 M in DMSO, 20 µL, 0.200 µmol, 1 mol%) and 2,5,8,11,14,17,20,23-octaoxapentacosan-25-yl 2-bromo-2,2-difluoroacetate **2d** (0.1 M in DMSO, 400 µL, 40.0 µmol, 2.00 eq.). The reaction mixture was irradiated with blue LEDs for 10 min then the yield was determined by quantitative ^{19}F NMR spectroscopy (60% yield); ^{19}F NMR (376 MHz, DMSO) δ −98.6 (s, 2F); HRMS (ESI) m/z: $[M + H]^+$ Calcd for $C_{34}H_{53}F_2N_2O_{13}$ 735.3516; Found 735.3535 (2.6 ppm error).

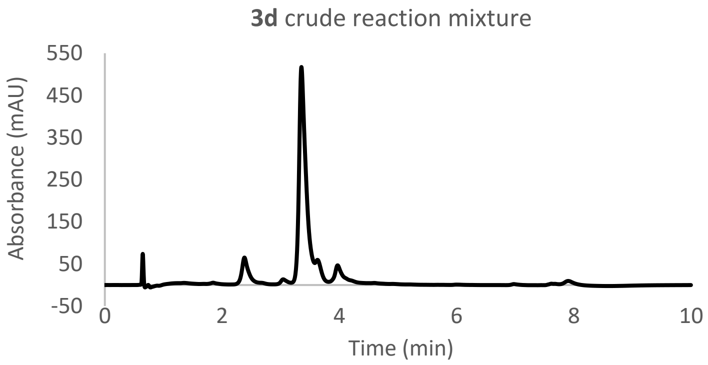

Figure S17. Reaction mixture analytical HPLC chromatogram for product **3d** Rt = 3.35 min on analytical gradient 40-80% B over 5 min, 0.6 mL min^{-1}, 280 nm.

Ethyl (*S*)-2-acetamido-3-(2-(1,1-difluoro-2-(hex-5-yn-1-yloxy)-2-oxoethyl)-1*H*-indol-3-yl)propanoate (3e) - analytical scale

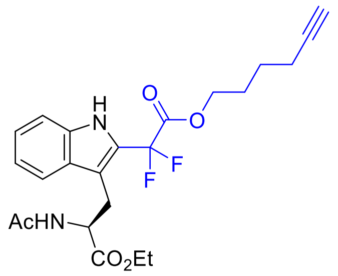

Prepared according to general procedure C using Ac-Trp-OEt **1** (0.1 M in DMSO, 200 µL, 20.0 µmol, 1.00 eq.), 4CzIPN (0.01 M in DMSO, 20 µL, 0.200 µmol, 1 mol%) and hex-5-yn-1-yl 2-bromo-2,2-difluoroacetate **2e** (0.1 M in DMSO, 400 µL, 40.0 µmol, 2.00 eq.). The reaction mixture was irradiated with blue LEDs for 30 min then the yield was determined by quantitative ^{19}F NMR spectroscopy (21% yield); ^{19}F NMR (376 MHz, DMSO) δ −98.7 (s, 2F); HRMS (ESI) m/z: $[M + H]^+$ Calcd for $C_{23}H_{27}F_2N_2O_5$ 449.1888; Found 449.1871 (3.8 ppm error).

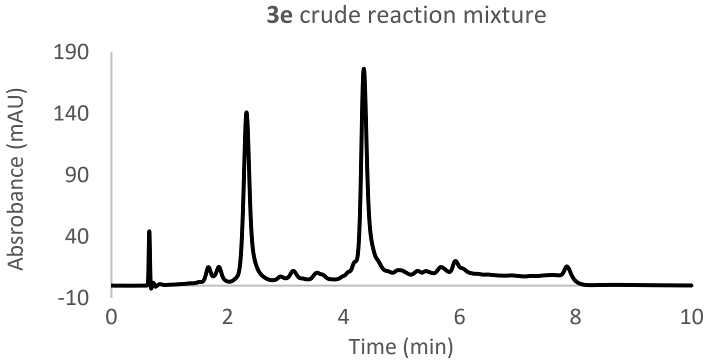

Figure S18. Reaction mixture analytical HPLC chromatogram for product **3e** Rt = 4.35 min on analytical gradient 40-80% B over 5 min, 0.6 mL min^{-1}, 280 nm.

Ethyl (*S*)-2-acetamido-3-(2-(1,1-difluoro-2-morpholino-2-oxoethyl)-1*H*-indol-3-yl)propanoate (3f) - analytical scale

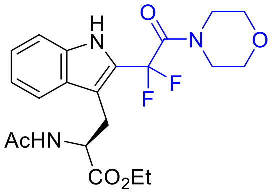

Prepared according to general procedure C using Ac-Trp-OEt **1** (0.1 M in DMSO, 200 µL, 20.0 µmol, 1.00 eq.), 4CzIPN (0.01 M in DMSO, 20 µL, 0.200 µmol, 1 mol%) and 2-bromo-2,2-difluoro-1-morpholinoethan-1-one **2f** (0.1 M in DMSO, 400 µL, 40.0 µmol, 2.00 eq.). The reaction mixture was irradiated with blue LEDs for 15 min then the yield was determined by quantitative ^{19}F NMR spectroscopy (39% yield); ^{19}F NMR (376 MHz, DMSO) δ −88.7 (d, *J* = 274 Hz), −89.8 (d, *J* = 274 Hz); HRMS (ESI) m/z: $[M + H]^+$ Calcd for $C_{21}H_{26}F_2N_3O_5$ 438.1841; Found 438.1838 (0.7 ppm error).

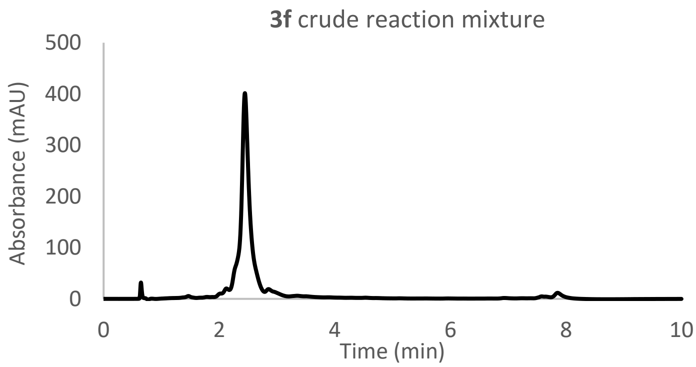

Figure S19. Reaction mixture analytical HPLC chromatogram for product **3f** Rt = 2.45 min on analytical gradient 40-80% B over 5 min, 0.6 mL min^{-1}, 280 nm.

Ethyl (*S*)-2-acetamido-3-(2-(1,1-difluoro-2-oxo-2-((pyridin-3-ylmethyl)amino)ethyl)-1*H*-indol-3-yl)propanoate (3g) - analytical scale

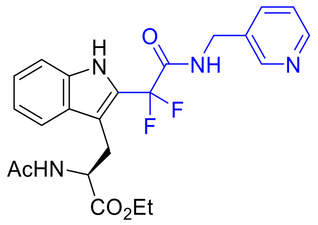

Prepared according to general procedure C using Ac-Trp-OEt **1** (0.1 M in DMSO, 200 µL, 20.0 µmol, 1.00 eq.), 4CzIPN (0.01 M in DMSO, 20 µL, 0.200 µmol, 1 mol%) and 2-bromo-2,2-difluoro-*N*-(pyridin-3-ylmethyl)acetamide **2g** (0.1 M in DMSO, 400 µL, 40.0 µmol, 2.00 eq.). The reaction mixture was irradiated with blue LEDs for 30 min then the yield was determined by quantitative ^{19}F NMR spectroscopy (48%); ^{19}F NMR (376 MHz, DMSO) δ −96.5 (d, 1F, *J* = 267 Hz), −97.2 (d, 1F, *J* = 267 Hz); HRMS (ESI) m/z: $[M + H]^+$ Calcd for $C_{23}H_{25}F_2N_4O_4$ 459.1838; Found 459.1846 (1.6 ppm error).

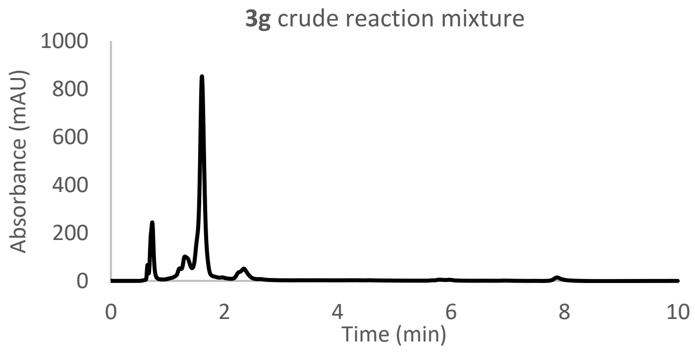

Figure S20. Reaction mixture analytical HPLC chromatogram for product **3g** Rt = 1.61 min on analytical gradient 40-80% B over 5 min, 0.6 mL min^{-1}, 280 nm.

Ethyl (*S*)-2-acetamido-3-(2-(2-(benzylamino)-1,1-difluoro-2-oxoethyl)-1*H*-indol-3-yl)propanoate (3h) - analytical scale

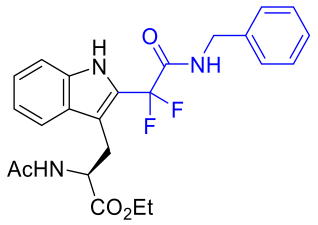

Prepared according to general procedure C using Ac-Trp-OEt **1** (0.1 M in DMSO, 200 µL, 20.0 µmol, 1.00 eq.), 4CzIPN (0.01 M in DMSO, 20 µL, 0.200 µmol, 1 mol%) and 2-bromo-*N*-benzyl-2,2-difluoroacetamide **2h** (0.1 M in DMSO, 400 µL, 40.0 µmol, 2.00 eq.). The reaction mixture was irradiated with blue LEDs for 60 min then the yield was determined by quantitative ^{19}F NMR spectroscopy (53% yield); ^{19}F NMR (376 MHz, DMSO) δ −96.4 (d, 1F, *J* = 266 Hz), −97.3 (d, 1F, *J* = 266 Hz); HRMS (ESI) m/z: $[M - H]^-$ Calcd for $C_{24}H_{24}F_2N_3O_4$ 456.1735; Found 456.1731 (0.9 ppm error).

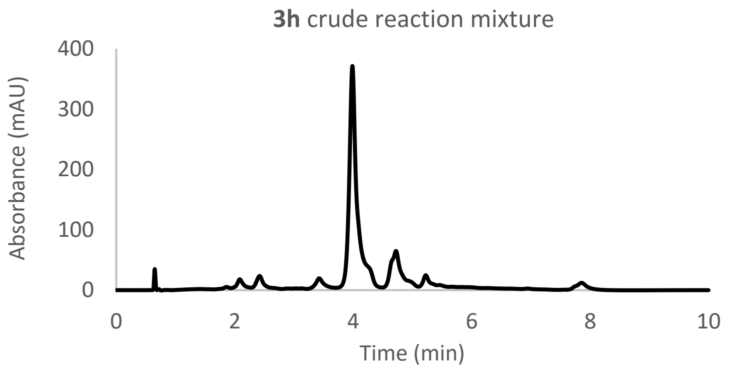

Figure S21. Reaction mixture analytical HPLC chromatogram for product **3h** Rt = 3.99 min on analytical gradient 40-80% B over 5 min, 0.6 mL min^{-1}, 280 nm.

Ethyl (*S*)-2-acetamido-3-(2-(2-(benzylamino)-1,1-difluoro-2-oxoethyl)-1*H*-indol-3-yl)propanoate (3h)

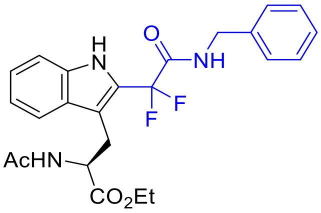

Prepared according to general procedure D using Ac-Trp-OEt **1** (54.9 mg, 200 µmol, 1.00 eq.), 4CzIPN (1.6 mg, 2.00 µmol, 1 mol%) and 2-bromo-N-benzyl-2,2-difluoroacetamide **2h** (106 mg, 400 µmol, 2.00 eq.). The reaction mixture was irradiated with blue LEDs for 30 min then was analysed by quantitative ^{19}F NMR spectroscopy (51% yield) and analytical HPLC. The crude material was purified by flash column chromatography (SiO_2, pentane/EtOAc, 1:1) to give the title compound **3h** (48.1 mg, 0.105 mmol, 53%) as a colourless crystalline solid; R_f = 0.48 (pentane/EtOAc, 1:1); mp = 60-62 °C; $υ_{max}$/cm^{-1} (ATR) 3283, 3112, 3064, 3032, 2980, 2921, 2850, 1731, 1688, 1650; ^{1}H NMR (400 MHz, $CDCl_3$) δ 9.19 (s, 1H), 7.80 (t, 1H, *J* = 5.0 Hz), 7.58 (d, 1H, *J* = 8.0 Hz), 7.35 (d, 1H, *J* = 8.2 Hz), 7.32 – 7.19 (m, 6H), 7.13 (ddd, 1H, *J* = 8.0, 6.9, 0.9 Hz), 6.71 (d, 1H, *J* = 7.3 Hz), 4.73 (ddd, 1H, *J* = 7.3, 7.2, 7.2 Hz), 4.64 – 4.39 (m, 2H), 4.12 – 3.87 (m, 2H), 3.42 – 3.23 (m, 2H), 1.85 (s, 3H), 1.03 (t, 3H, *J* = 7.1 Hz); ^{13}C {^{1}H} NMR (101 MHz, $CDCl_3$) δ 172.2, 170.8, 163.7 (t, *J* = 31.2 Hz), 136.7, 135.8, 128.9, 128.1, 128.1, 127.8, 125.3 (t, *J* = 29.9 Hz), 124.5, 120.6, 119.6, 112.8 (t, *J* = 251 Hz), 112.1, 111.8 (t, *J* = 2.0 Hz), 61.8, 53.6, 44.0, 27.2, 22.8, 13.9; ^{19}F NMR (376 MHz, $CDCl_3$) δ −97.7 (d, 1F, *J* = 268 Hz), −101.6 (d, 1F, *J* = 268 Hz); HRMS (ESI) m/z: [M + Na]$^+$ Calcd for $C_{24}H_{25}F_2N_3NaO_4$ 480.1705; Found 480.1706 (0.2 ppm error).

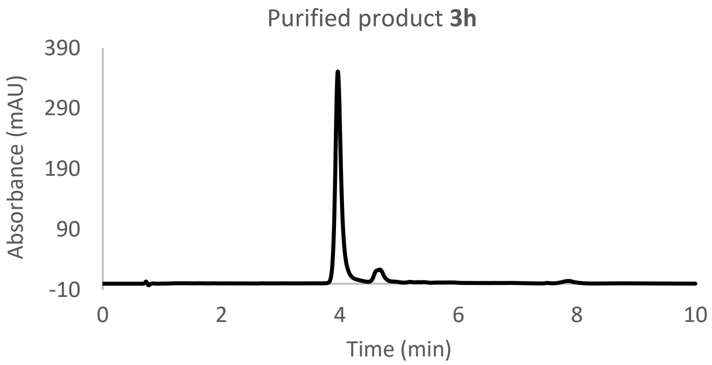

Figure S22. Analytical HPLC chromatogram for purified product **3h** Rt = 3.97 min on analytical gradient 40-80% B over 5 min, 0.6 mL min^{-1}, 280 nm.

Ethyl (*S*)-2-acetamido-3-(2-(2-(cyclopropylamino)-1,1-difluoro-2-oxoethyl)-1*H*-indol-3-yl)propanoate (3i) - analytical scale

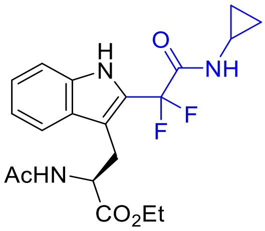

Prepared according to general procedure C using Ac-Trp-OEt **1** (0.1 M in DMSO, 200 µL, 20.0 µmol, 1.00 eq.), 4CzIPN (0.01 M in DMSO, 20 µL, 0.200 µmol, 1 mol%) and 2-bromo-N-cyclopropyl-2,2-difluoroacetamide **2i** (0.1 M in DMSO, 400 µL, 40.0 µmol, 2.00 eq.). The reaction mixture was irradiated with blue LEDs for 60 min then the yield was determined by quantitative ^{19}F NMR spectroscopy (65% yield); ^{19}F NMR (376 MHz, DMSO) δ −96.1 (d, 1F, *J* = 265 Hz), −97.0 (d, 1F, *J* = 265 Hz); HRMS (ESI) m/z: $[M + H]^+$ Calcd for $C_{20}H_{24}F_2N_3O_4$ 408.1735; Found 408.1719 (3.9 ppm error).

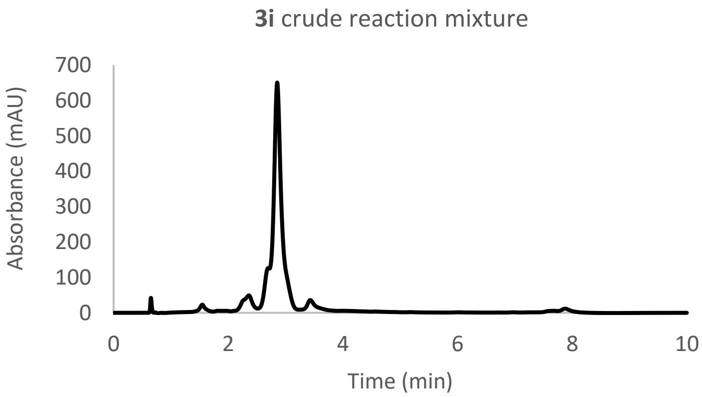

Figure S23. Reaction mixture analytical HPLC chromatogram for product **3i** Rt = 2.85 min on analytical gradient 40-80% over 5 min, 0.6 mL min^{-1}, 280 nm.

Ethyl (*S*)-2-acetamido-3-(2-(2-(*tert*-butylamino)-1,1-difluoro-2-oxoethyl)-1*H*-indol-3-yl)propanoate (3j) - analytical scale

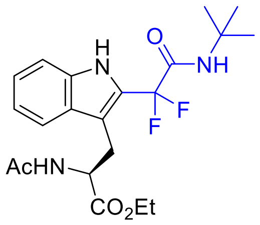

Prepared according to general procedure C using Ac-Trp-OEt **1** (0.1 M in DMSO, 200 µL, 20.0 µmol, 1.00 eq.), 4CzIPN (0.01 M in DMSO, 20 µL, 0.200 µmol, 1 mol%) and *2*-bromo-*N*-(*tert*-butyl)-2,2-difluoroacetamide **2j** (0.1 M in DMSO, 400 µL, 40.0 µmol, 2.00 eq.). The reaction mixture was irradiated with blue LEDs for 75 min then the yield was determined by quantitative ^{19}F NMR spectroscopy (50% yield); ^{19}F NMR (376 MHz, DMSO) δ −95.4 (d, *J* = 262 Hz), −97.4 (d, *J* = 262 Hz); HRMS (ESI) m/z: $[M + H]^+$ Calcd for $C_{21}H_{28}F_2N_3O_4$ 424.2048; Found 424.2057 (2.1 ppm error).

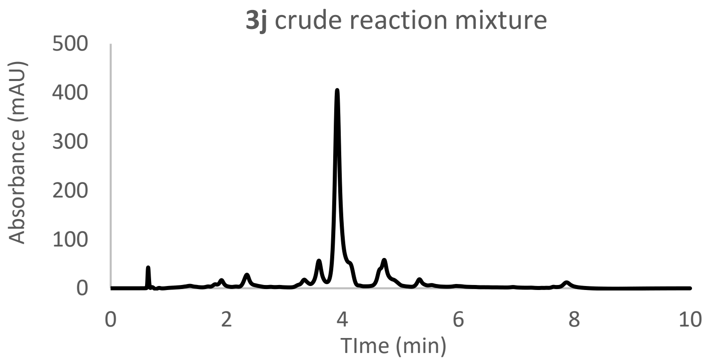

Figure S24. Reaction mixture analytical HPLC chromatogram for product **3j** Rt = 3.91 min on analytical gradient 40-80% B over 5 min, 0.6 mL min^{-1}, 280 nm.

Ethyl (*S*)-2-acetamido-3-(2-(1,1-difluoro-2-oxo-2-((2-(5-((3a*S*,4*S*,6a*R*)-2-oxohexahydro-1*H*-thieno[3,4-d]imidazol-4-yl)pentanamido)ethyl)amino)ethyl)-1*H*-indol-3-yl)propanoate (3k) - analytical scale

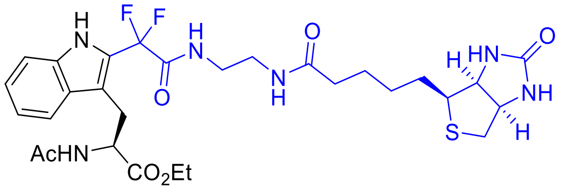

Prepared according to general procedure C using Ac-Trp-OEt **1** (0.1 M in DMSO, 200 µL, 20.0 µmol, 1.00 eq.), 4CzIPN (0.01 M in DMSO, 20 µL, 0.200 µmol, 1 mol%) and *N*-(2-(2-bromo-2,2-difluoroacetamido)ethyl)-5-((3a*S*,4*S*,6a*R*)-2-oxohexahydro-1*H*-thieno[3,4-d]imidazol-4 yl)pentanamide **2k** (0.1 M in DMSO, 400 µL, 40.0 µmol, 2.00 eq.). The reaction mixture was irradiated with blue LEDs for 150 min then the yield was determined by quantitative ^{19}F NMR spectroscopy (40% yield); ^{19}F NMR (376 MHz, DMSO) δ −97.4 (s, 2F); HRMS (ESI) m/z: [M + H]$^+$ Calcd for $C_{29}H_{39}F_2N_6O_6S$ 637.2620; found: 637.2591 (4.6 ppm error).

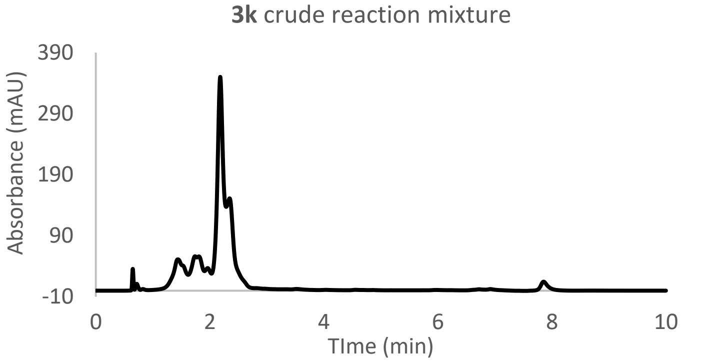

Figure S25. Reaction mixture analytical HPLC chromatogram for product **3k** Rt = 2.18 min on analytical gradient 40-80% B over 5 min, 0.6 mL min^{-1}, 280 nm.

6. Photochemical alkylation of Trp-containing peptides

General Procedure E: Photocatalytic difluoroalkylation of Trp-containing peptides

A solution of peptide (25 mM in DMSO, 200 µL, 5.00 µmol, 1.00 eq.), 4CzIPN (2.5 mM in DMSO, 20 µL, 0.0500 µmol, 1 mol%) and bromodifluoroalkyl reagent (100 mM in DMSO, 100 µL, 10.0 µmol, 2.00 eq.) in DMSO (180 µL, total solvent volume 500 µL) was purged with N_2 (5 min) and then irradiated with blue LEDs (PhotoRedOx Box) for 10 - 150 min at room temperature. α,α,α-trifluorotoluene (101 µL, 16.5 mM in DMSO, 1.65 µmol, 0.333 eq.) was added to the reaction mixture, then quantitative NMR spectroscopy and analytical HPLC analysis were carried out. The reaction mixture was diluted with 0.1% TFA in H_2O (2.60 mL) and then purified by semi-preparative HPLC.

Ac-WHISKEY-NH_2 conjugated with ethyl bromodifluoroacetate (5a)

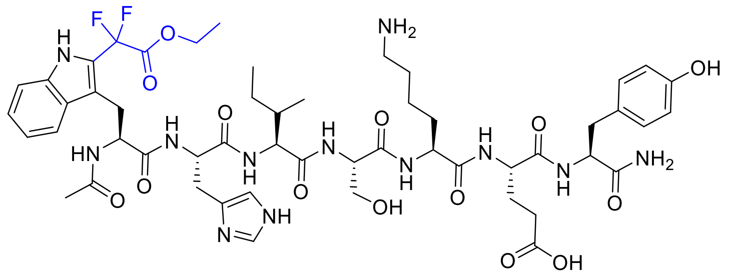

Prepared according to general procedure E using Ac-WHISKEY-NH_2 **4** (25.0 mM in DMSO, 200 µL, 5.00 µmol, 1.00 eq.), 4CzIPN (2.50 mM in DMSO, 20 µL, 0.0500 µmol, 1 mol%) and ethyl bromodifluoroacetate **2a** (0.1 M in DMSO, 100 µL, 10.0 µmol, 2.00 eq.). The reaction mixture was irradiated with blue LEDs for 10 min, then was analysed by quantitative ^{19}F NMR spectroscopy (55% yield). The reaction mixture was then purified by semi-preparative RP-HPLC (2-70% B over 30 min) to give the title compound **5a** (2.4 mg, 2.13 µmol, 43%) as a colourless powder; ^{1}H NMR (500 MHz, CD_3OD) δ 8.69 (d, 1H, *J* = 1.4 Hz), 7.58 (dd, 1H, *J* = 8.2, 1.1 Hz), 7.36 (dd, 1H, *J* = 8.3, 1.0 Hz), 7.24 (d, 1H, *J* = 1.4 Hz), 7.20 (ddd, 1H, *J* = 8.3, 6.9, 1.1 Hz), 7.11 – 7.00 (m, 3H), 6.69 (d, 2H, *J* = 8.5 Hz), 4.50 (dd, 1H, *J* = 8.7, 5.6 Hz), 4.46 – 4.39 (m, 3H), 4.38 – 4.31 (m, 2H), 4.31 – 4.23 (m, 2H), 4.04 (d, 1H, *J* = 7.3 Hz), 3.83 (dd, 1H, *J* = 10.9, 5.7 Hz), 3.77 (dd, 1H, *J* = 10.9, 6.0 Hz), 3.28 – 3.21 (m, 2H), 3.08 – 3.00 (m, 2H), 2.94 – 2.88 (m, 2H), 2.88 – 2.80 (m, 1H), 2.40 – 2.23 (m, 2H), 2.07 – 1.97 (m, 1H), 1.96 (s, 3H), 1.93 – 1.77 (m, 3H), 1.76 – 1.68 (m, 1H), 1.68 – 1.58 (m, 2H), 1.54 – 1.46 (m, 1H), 1.47 – 1.38 (m, 2H), 1.33 – 1.26 (m, 4H), 1.22 – 1.10 (m, 1H), 1.00 – 0.86 (m, 6H); ^{19}F NMR (376 MHz, CD_3OD) δ −77.0 (s, TFA), −101.4 (d, 1F, *J* = 266 Hz), −103.5 (d, 1F, J = 266 Hz); HRMS (ESI) m/z: $[M + H]^+$ Calcd for $C_{52}H_{71}F_2N_{12}O_{14}$ 1125.5181; Found 1125.5141 (3.6 ppm error).

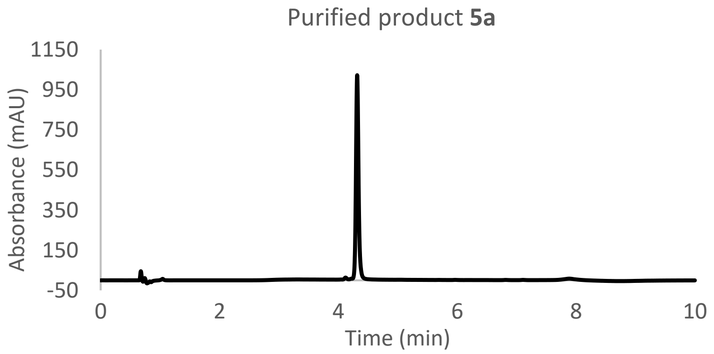

Figure S26. Analytical HPLC chromatogram for purified product **5a** Rt = 4.32 min on analytical gradient 2-95% B over 5 min, 0.6 mL min^{-1}, 280 nm.

Confirmation of regioselectivity and chemoselectivity was carried out using ^1H NMR spectroscopy to show the disappearance of the C2 proton on Trp. COSY/ TOCSY experiments were carried out to show that the two signals in the aromatic region of the product spectrum (8.69 and 7.24 ppm) are coupling which proved that they correspond to the histidine proton signals. This was carried out for every alkylated peptide product and a representative example is shown below.

Stacked **^{1}H NMR** spectrum of peptide **4** and product **5a** (400/500 MHz, CD_3OD)

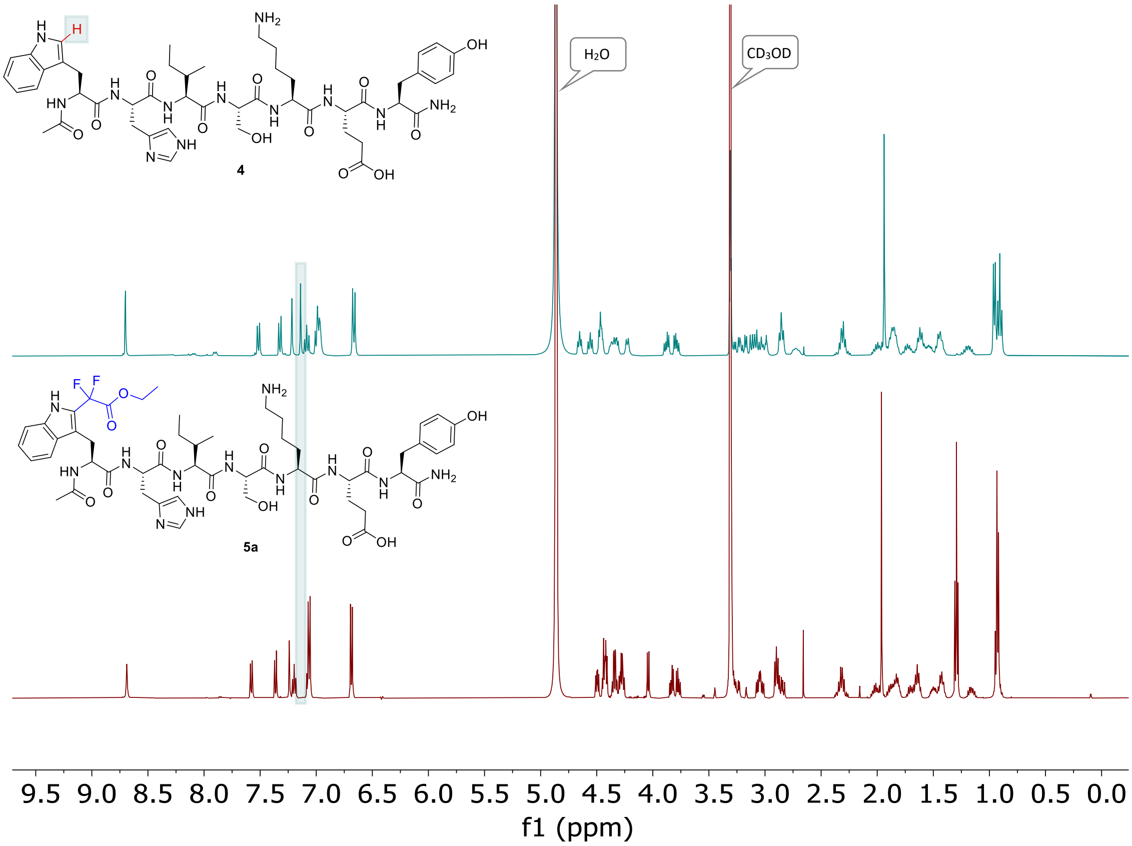

COSY spectrum of product **5a** (500 MHz, CD_3OD)

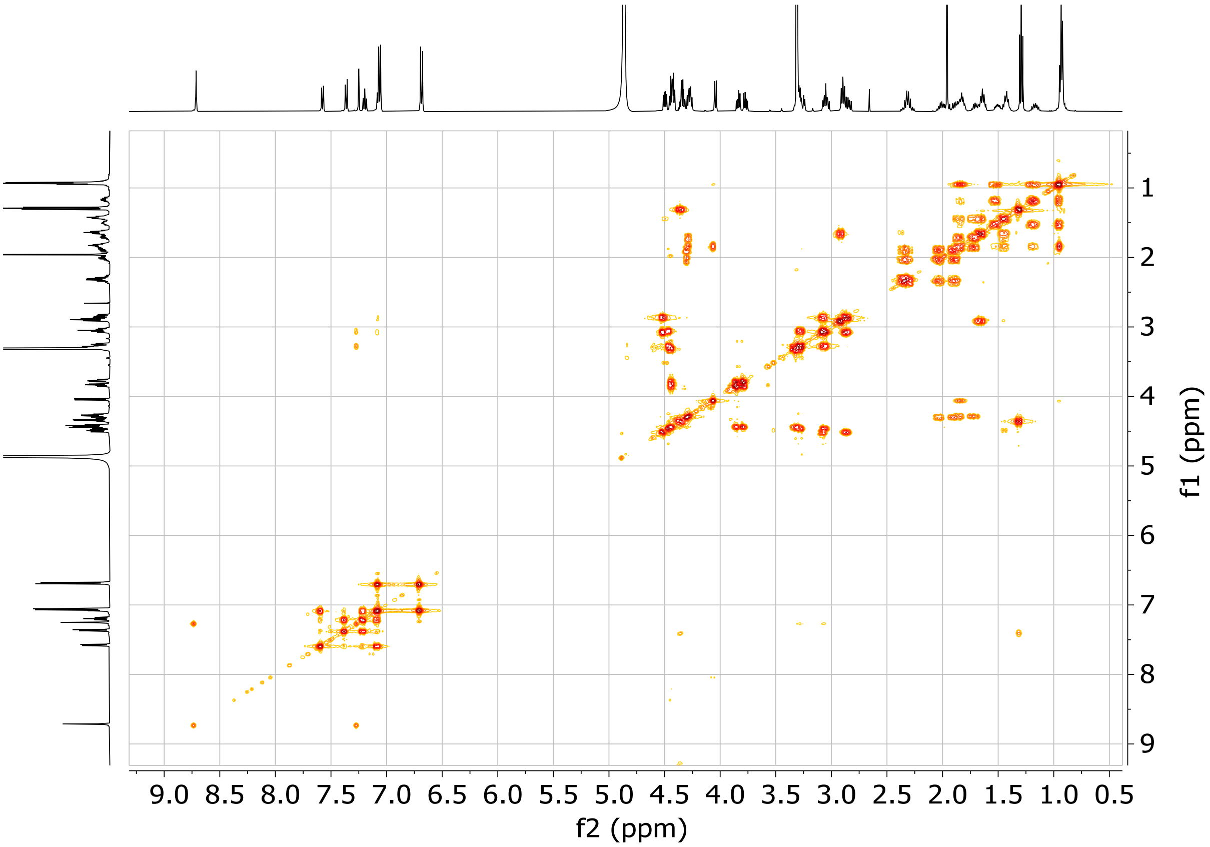

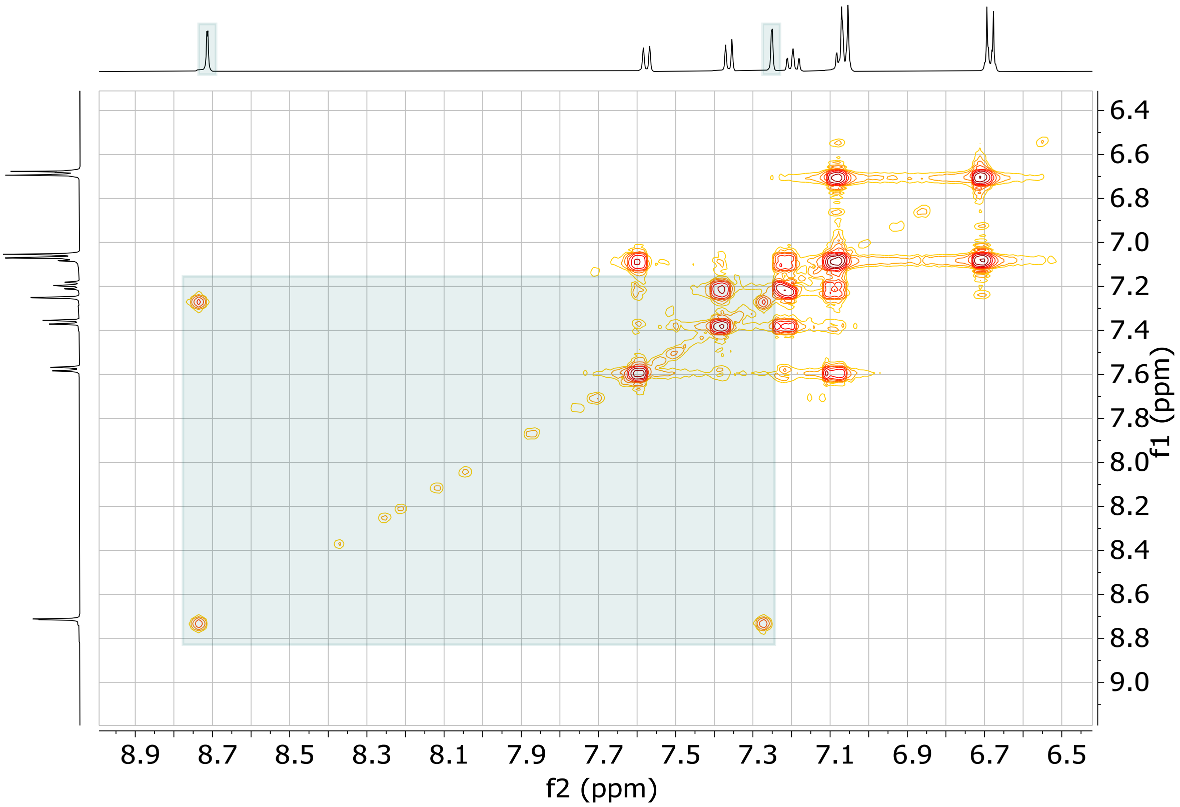

Ac-WHISKEY-NH_2 conjugated with benzyl 2-bromo-2,2-difluoroacetate (5b)

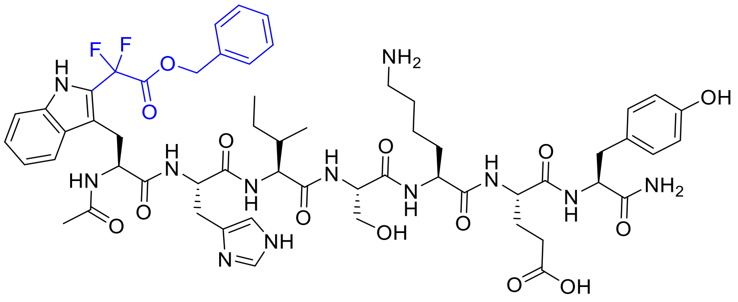

Prepared according to general procedure E using Ac-WHISKEY-NH_2 **4** (25.0 mM in DMSO, 200 µL, 5.00 µmol, 1.00 eq.), 4CzIPN (2.50 mM in DMSO, 20 µL, 0.0500 µmol, 1 mol%) and benzyl 2-bromo-2,2-difluoroacetate **2b** (0.1 M in DMSO, 100 µL, 10.0 µmol, 2.00 eq.). The reaction mixture was irradiated with blue LEDs for 10 min, then was analysed by quantitative ^{19}F NMR spectroscopy (42% yield). The reaction mixture was purified by semi-preparative RP-HPLC (2-70% B over 30 min) to give the title compound **5b** (2.80 mg, 2.36 µmol, 47%) as a colourless powder; ^{1}H NMR (400 MHz, CD_3OD) δ 8.55 (s, 1H), 7.57 (dd, 1H, *J* = 8.2, 0.6 Hz), 7.36 (dd, 1H, *J* = 8.3, 0.9 Hz), 7.35 – 7.24 (m, 5H), 7.24 – 7.16 (m, 2H), 7.12 – 7.00 (m, 3H), 6.68 (d, 2H, *J* = 8.4 Hz), 5.32 (d, 1H, *J* = 12.2 Hz), 5.32 (d, 1H, *J* = 12.2 Hz), 4.53 – 4.46 (m, 1H), 4.46 – 4.34 (m, 3H), 4.31 – 4.20 (m, 2H), 4.03 (d, 1H, *J* = 7.2 Hz), 3.90 – 3.74 (m, 2H), 3.28 – 3.14 (m, 3H), 3.11 – 2.98 (m, 2H), 2.96 – 2.79 (m, 3H), 2.39 – 2.24 (m, 2H), 2.07 – 1.96 (m, 1H), 1.94 (s, 3H), 1.92 – 1.76 (m, 3H), 1.75 – 1.66 (m, 1H), 1.66 – 1.57 (m, 2H), 1.54 – 1.45 (m, 1H), 1.45 – 1.35 (m, 2H), 1.23 – 1.06 (m, 1H), 1.00 – 0.82 (m, 6H); ^{19}F NMR (377 MHz, CD_3OD) δ −76.9 (s, TFA), −102.2 (d, 1F, *J* = 266 Hz), −103.1 (d, 1F, *J* = 266 Hz); HRMS (ESI) m/z: $[M + H]^+$ Calcd for $C_{57}H_{73}F_2N_{12}O_{14}$ 1187.5337; Found 1187.5307 (2.5 ppm error).

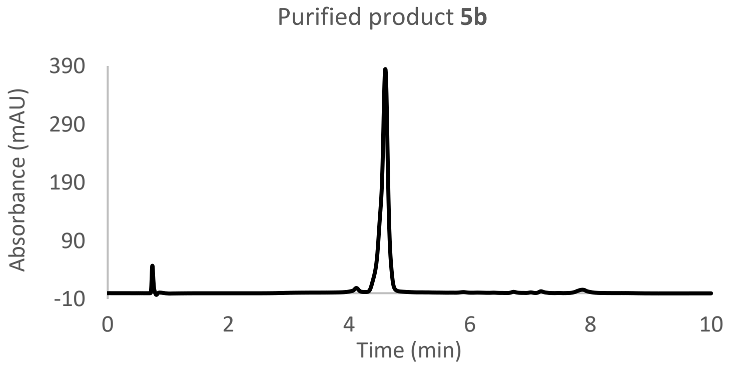

Figure S27. Analytical HPLC chromatogram for purified product **5b** Rt = 4.60 min on analytical gradient 2-95% B over 5 min, 0.6 mL min^{-1}, 280 nm.

Ac-WHISKEY-NH_2 conjugated with 4-bromobenzyl 2-bromo-2,2-difluoroacetate (5c)

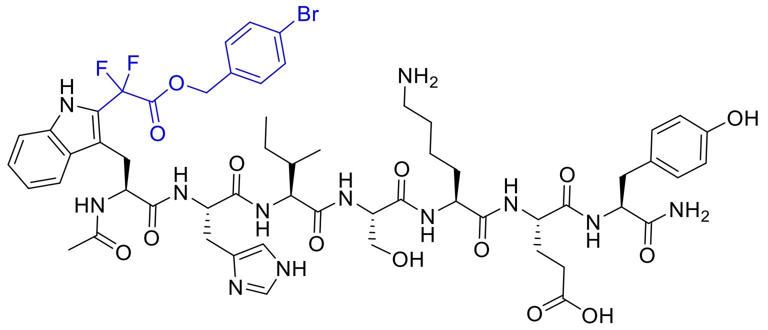

Prepared according to general procedure E using Ac-WHISKEY-NH_2 **4** (25.0 mM in DMSO, 200 µL, 5.00 µmol, 1.00 eq.), 4CzIPN (2.50 mM in DMSO, 20 µL, 0.0500 µmol, 1 mol%) and 4-bromobenzyl 2-bromo-2,2-difluoroacetate **2c** (0.1 M in DMSO, 100 µL, 10.0 µmol, 2.00 eq.). The reaction mixture was irradiated with blue LEDs for 10 min, then was analysed by quantitative ^{19}F NMR spectroscopy (43% yield). The reaction mixture was purified by semi-preparative RP-HPLC (2-70% B over 30 min) to give to give the title compound **5c** (2.4 mg, 1.90 µmol, 38%) as a colourless powder; ^{1}H NMR (500 MHz, DMSO-d_6) δ 14.20 – 13.70 (m, 1H), 12.09 (s, 1H), 11.64 (s, 1H), 9.16 (s, 1H), 8.94 (s, 1H), 8.16 (d, 1H, *J* = 7.4 Hz), 8.08 (d, 1H, *J* = 7.8 Hz), 8.03 – 7.93 (m, 3H), 7.82 (d, 1H, *J* = 8.3 Hz), 7.76 (d, 1H, *J* = 8.1 Hz), 7.69 (d, 1H, *J* = 8.1 Hz), 7.64 – 7.56 (m, 3H), 7.53 (d, 2H, *J* = 8.4 Hz), 7.37 (d, 1H, *J* = 8.2 Hz), 7.35 – 7.31 (m, 1H), 7.29 (d, 2H, *J* = 8.4 Hz), 7.27 – 7.23 (m, 1H), 7.20 (dd, 1H, *J* = 8.2, 7.6 Hz), 7.07 – 7.03 (m, 2H), 6.99 (d, 2H, *J* = 8.5 Hz), 6.63 (d, 2H, *J* = 8.5 Hz), 5.32 (s, 2H), 4.63 – 4.53 (m, 1H), 4.45 – 4.37 (m, 1H), 4.37 – 4.27 (m, 2H), 4.26 – 4.11 (m, 3H), 3.66 – 3.50 (m, 2H), 3.23 – 3.14 (m, 1H), 3.13 – 3.05 (m, 1H), 3.05 – 2.91 (m, 2H), 2.91 – 2.81 (m, 1H), 2.77 – 2.67 (m, 3H), 2.23 – 2.13 (m, 2H), 1.91 – 1.81 (m, 1H), 1.73 – 1.62 (m, 6H), 1.54 – 1.45 (m, 3H), 1.45 – 1.35 (m, 1H), 1.33 – 1.23 (m, 2H), 1.10 – 1.02 (m, 1H), 0.85 – 0.71 (m, 6H); ^{19}F NMR (376 MHz, DMSO-d_6) δ −73.5 (s, TFA), −98.3 (d, 1F, *J* = 265 Hz), −99.1 (d, 1F, *J* = 265 Hz); HRMS (ESI) m/z: $[M + H]^+$ Calcd for $C_{57}H_{72}{}^{79}BrF_2N_{12}O_{14}$ 1265.4442; Found 1265.4322 (9.5 ppm error).

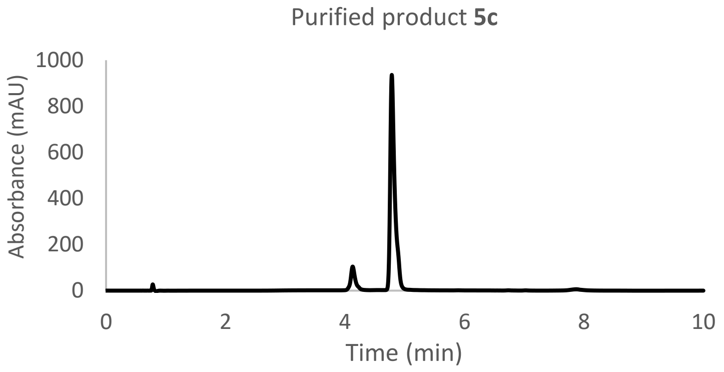

Figure S28. Analytical HPLC chromatogram for purified product **5c** Rt = 4.78 min on analytical gradient 2-95% B over 5 min, 0.6 mL min^{-1}, 280 nm.

Ac-WHISKEY-NH$_2$ conjugated with 2,5,8,11,14,17,20,23-octaoxapentacosan-25-yl 2-bromo-2,2-difluoroacetate (5d)

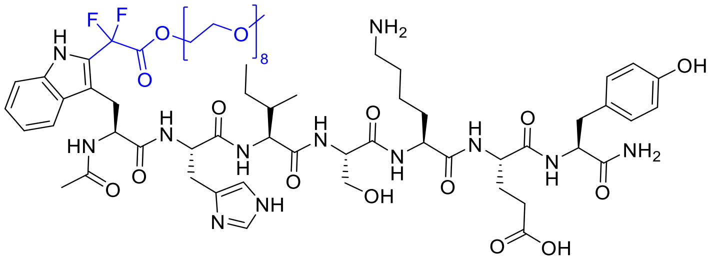

Prepared according to general procedure E using Ac-WHISKEY-NH$_2$ **4** (25.0 mM in DMSO, 200 µL, 5.00 µmol, 1.00 eq.), 4CzIPN (2.50 mM in DMSO, 20 µL, 0.0500 µmol, 1 mol%) and 2,5,8,11,14,17,20,23-octaoxapentacosan-25-yl 2-bromo-2,2-difluoroacetate **2d** (0.1 M in DMSO, 100 µL, 10.0 µmol, 2.00 eq.). The reaction mixture was irradiated with blue LEDs for 10 min, then was analysed by quantitative ^{19}F NMR spectroscopy (42% yield). The reaction mixture was purified by semi-preparative RP-HPLC (2-70% B over 30 min) to give the title compound **5d** (3.5 mg, 2.39 µmol, 48%) as a colourless powder; ^{1}H NMR (400 MHz, CD$_3$OD) δ 8.71 (d, 1H, *J* = 1.0 Hz), 7.59 (d, 1H, *J* = 8.0 Hz), 7.38 (d, 1H, *J* = 8.2 Hz), 7.25 (d, 1H, *J* = 1.0 Hz), 7.21 (ddd, 1H, *J* = 8.2, 6.9, 1.1 Hz), 7.12 – 7.03 (m, 3H), 6.68 (d, 2H, *J* = 8.3 Hz), 4.56 – 4.36 (m, 5H), 4.34 – 4.22 (m, 2H), 4.05 (d, 1H, *J* = 7.2 Hz), 3.84 (dd, 1H, *J* = 10.9, 5.6 Hz), 3.77 (dd, 1H, *J* = 10.9, 6.0 Hz), 3.71 (dd, 2H, *J* = 5.3, 4.5 Hz), 3.69 – 3.57 (m, 20H), 3.57 – 3.47 (m, 8H), 3.46 – 3.41 (m, 2H), 3.34 (s, 3H), 3.25 – 3.20 (m, 1H), 3.11 – 2.97 (m, 2H), 2.94 – 2.87 (m, 2H), 2.87 – 2.78 (m, 1H), 2.48 – 2.20 (m, 2H), 2.10 – 1.98 (m, 1H), 1.96 (s, 3H), 1.94 – 1.78 (m, 3H), 1.77 – 1.68 (m, 1H), 1.68 – 1.58 (m, 2H), 1.56 – 1.47 (m, 1H), 1.48 –1.38 (m, 2H), 1.37 – 1.23 (m, 2H), 1.22 – 1.10 (m, 1H), 0.97 – 0.87 (m, 6H); ^{19}F NMR (376 MHz, CD$_3$OD) δ −77.0 (s, TFA) −101.2 (d, 1F, J = 265 Hz), −103.8 (d, 1F, J = 265 Hz); HRMS (ESI) m/z: [M – H + $C_2HF_3O_2$]$^-$ Calcd for $C_{67}H_{99}F_2N_{12}O_{22}$ 1575.6894; Found 1575.6959 (4.1 ppm error).

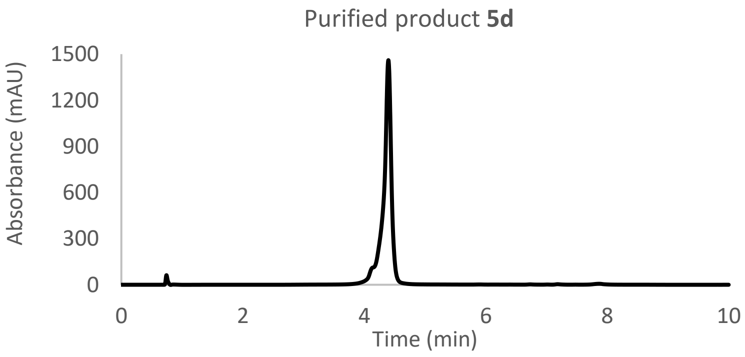

Figure S29. Analytical HPLC chromatogram for purified product **5d** Rt = 4.40 min on analytical gradient 2-95% B over 5 min, 0.6 mL min^{-1}, 280 nm.

Ac-WHISKEY-NH$_2$ conjugated with hex-5-yn-1-yl 2-bromo-2,2-difluoroacetate (5e)

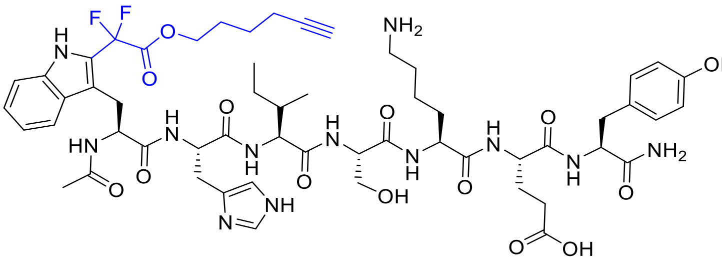

Prepared according to general procedure E using Ac-WHISKEY-NH$_2$ **4** (25.0 mM in DMSO, 200 µL, 5.00 µmol, 1.00 eq.), 4CzIPN (2.50 mM in DMSO, 20 µL, 0.0500 µmol, 1 mol%) and hex-5-yn-1-yl 2-bromo-2,2-difluoroacetate **2e** (0.100 M in DMSO, 100 µL, 10.0 µmol, 2.00 eq.). The reaction mixture was irradiated with blue LEDs for 30 min, then was analysed by quantitative ^{19}F NMR spectroscopy (15% yield). The reaction mixture was purified by semi-preparative RP-HPLC (2-70% B over 30 min) to give the title compound **5e** (1.0 mg, 0.85 µmol, 17%) as a colourless powder; ^{1}H NMR (400 MHz, CD$_3$OD) δ 8.59 (s, 1H), 7.58 (d, 1H, *J* = 8.2 Hz), 7.37 (d, 1H, *J* = 8.2 Hz), 7.22 (s, 1H), 7.21 – 7.16 (m, 1H), 7.09 – 6.98 (m, 3H), 6.69 (d, 2H, *J* = 8.4 Hz), 4.58 – 4.44 (m, 1H), 4.47 – 4.36 (m, 3H), 4.33 (dd, 2H, *J* = 6.9, 6.4 Hz), 4.30 – 4.18 (m, 2H), 4.04 (d, 1H, *J* = 7.2 Hz), 3.84 (dd, 1H, *J* = 10.9, 5.7 Hz), 3.78 (dd, 1H, *J* = 10.9, 5.8 Hz), 3.27 – 3.16 (m, 2H), 3.11 – 2.99 (m, 2H), 2.93 – 2.86 (m, 3H), 2.86 – 2.80 (m, 1H), 2.39 – 2.26 (m, 2H), 2.18 (dd, 1H, *J* = 3.0, 2.6 Hz), 2.13 (td, 2H, *J* = 6.9, 2.6 Hz), 2.07 – 1.85 (m, 3H), 1.97 (s, 3H), 1.85 – 1.74 (m, 3H), 1.74 – 1.57 (m, 3H), 1.55 – 1.39 (m, 5H), 1.22 – 1.10 (m, 1H), 0.96 – 0.90 (m, 6H); ^{19}F NMR (376 MHz, CD$_3$OD) δ −77.0 (s, TFA), −101.7 (d, 1F, *J* = 266 Hz), −103.3 (d, 1F, *J* = 266 Hz); HRMS (ESI) m/z: [M – H + CF$_3$CO$_2$H]$^-$ Calcd for $C_{56}H_{73}F_2N_{12}O_{14}$ 1289.5266; Found 1289.5311 (3.5 ppm error).

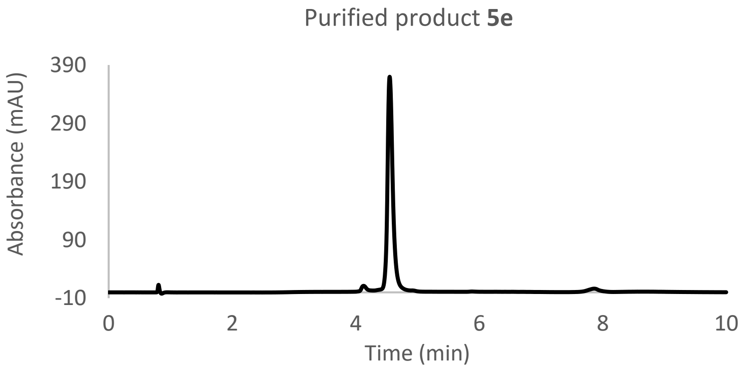

Figure S30. Analytical HPLC chromatogram for purified product **5e** Rt = 4.55 min on analytical gradient 2-95% B over 5 min, 0.6 mL min^{-1}, 280 nm.

Ac-WHISKEY-NH$_2$ conjugated with 2-bromo-2,2-difluoro-1-morpholinoethan-1-one (5f)

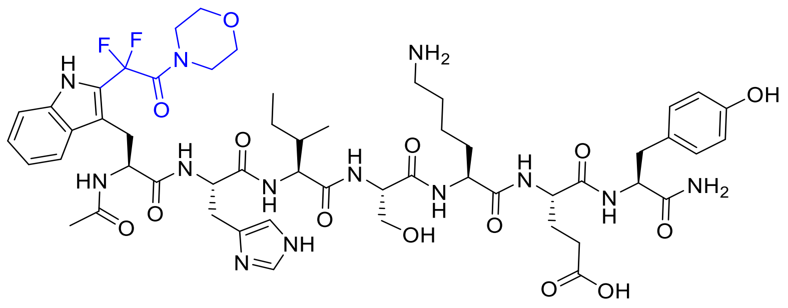

Prepared according to general procedure E using Ac-WHISKEY-NH$_2$ **4** (25.0 mM in DMSO, 200 µL, 5.00 µmol, 1.00 eq.), 4CzIPN (2.50 mM in DMSO, 20 µL, 0.0500 µmol, 1 mol%) and 2-bromo-2,2-difluoro-1-morpholinoethan-1-one **2f** (0.1 M in DMSO, 100 µL, 10.0 µmol, 2.00 eq.). The reaction mixture was irradiated with blue LEDs for 15 min, then was analysed by quantitative ^{19}F NMR spectroscopy (35% yield). The reaction mixture was then purified by semi-preparative RP-HPLC (2-70% B over 30 min) to give the title compound **5f** (2.20 mg, 1.89 µmol, 38%) as a colourless powder; ^{1}H NMR (500 MHz, CD_3OD) δ 8.71 (s, 1H), 7.65 (d, 1H, *J* = 8.1 Hz), 7.40 (d, 1H, *J* = 8.2 Hz), 7.29 (d, 1H, *J* = 1.4 Hz), 7.23 (dd, 1H, *J* = 8.2, 7.6 Hz), 7.10 (dd, 1H, *J* = 8.1, 7.6 Hz), 7.06 (d, 2H, *J* = 8.5 Hz), 6.69 (d, 2H, *J* = 8.5 Hz), 4.54 (dd, 1H, *J* = 6.9, 6.1 Hz), 4.50 (dd, 1H, *J* = 8.5, 5.7 Hz), 4.47 – 4.39 (m, 2H), 4.34 – 4.26 (m, 2H), 4.11 (d, 1H, *J* = 7.2 Hz), 3.86 (dd, 1H, *J* = 10.9, 5.7 Hz), 3.79 (dd, 1H, *J* = 10.9, 6.0 Hz), 3.77 – 3.63 (m, 4H), 3.58 – 3.51 (m, 2H), 3.50 – 3.42 (m, 2H), 3.28 – 3.20 (m, 2H), 3.19 – 3.01 (m, 3H), 2.94 – 2.87 (m, 2H), 2.88 – 2.79 (m, 1H), 2.46 – 2.23 (m, 2H), 2.13 – 1.98 (m, 1H), 1.89 (s, 3H), 1.88 – 1.80 (m, 3H), 1.78 – 1.70 (m, 1H), 1.70 – 1.60 (m, 2H), 1.57 – 1.49 (m, 1H), 1.48 – 1.38 (m, 2H), 1.25 – 1.12 (m, 1H), 0.99 – 0.87 (m, 6H); ^{19}F NMR (376 MHz, CD_3OD) δ −77.0 (s, TFA), −92.2 (d, 1F, *J* = 279 Hz), −93.3 (d, 1F, *J* = 279 Hz); HRMS (ESI) m/z: $[M + H]^+$ Calcd for $C_{54}H_{74}F_2N_{13}O_{14}$ 1166.5446; Found 1166.5426 (1.7 ppm error).

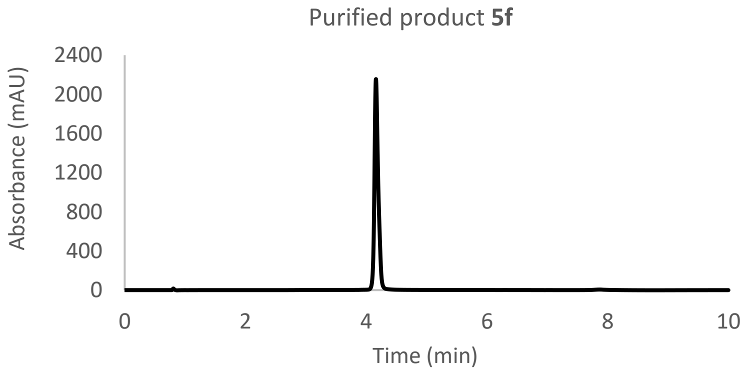

Figure S31. Analytical HPLC chromatogram for purified product **5f** Rt = 4.16 min on analytical gradient 2-95% B over 5 min, 0.6 mL min^{-1}, 280 nm.

Ac-WHISKEY-NH$_2$ conjugated with 2-bromo-2,2-difluoro-*N*-(pyridin-3-ylmethyl)acetamide (5g)

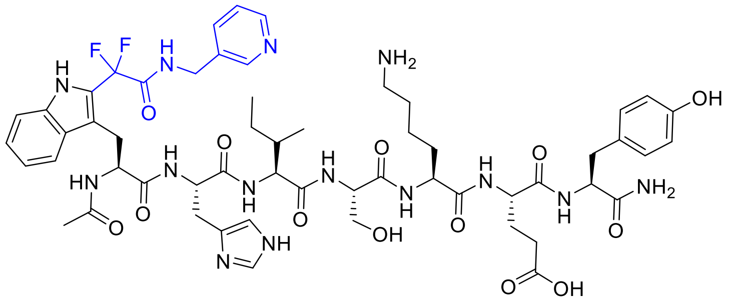

Prepared according to general procedure E using Ac-WHISKEY-NH$_2$ **4** (25.0 mM in DMSO, 200 µL, 5.00 µmol, 1.00 eq.), 4CzIPN (2.50 mM in DMSO, 20 µL, 0.0500 µmol, 1 mol%) and 2-bromo-2,2-difluoro-*N*-(pyridin-3-ylmethyl)acetamide **2g** (0.1 M in DMSO, 100 µL, 10.0 µmol, 2.00 eq.). The reaction mixture was irradiated with blue LEDs for 60 min, then was analysed by quantitative ^{19}F NMR spectroscopy (34% yield). The reaction mixture was then purified by semi-preparative RP-HPLC (2-70% B over 30 min) to give the title compound **5g** (2.50 mg, 2.10 µmol, 42%) as a colourless powder; ^{1}H NMR (500 MHz, CD_3OD) δ 8.72 (d, 1H, *J* = 1.4 Hz), 8.58 (d, 1H, *J* = 2.1 Hz), 8.51 (dd, 1H, *J* = 5.1, 1.6 Hz), 7.91 (ddd, 1H, *J* = 8.0, 2.1, 1.6 Hz), 7.65 (d, 1H, *J* = 8.2 Hz), 7.50 (dd, 1H, *J* = 8.0, 5.1 Hz), 7.37 (d, 1H, *J* = 8.3 Hz), 7.28 (d, 1H, *J* = 1.4 Hz), 7.21 (ddd, 1H, *J* = 8.3, 7.0, 1.1 Hz), 7.13 – 7.02 (m, 3H), 6.68 (d, 2H, *J* = 8.5 Hz), 4.68 – 4.47 (m, 6H), 4.44 (dd, 1H, *J* = 6.1, 5.7 Hz), 4.33 – 4.25 (m, 2H), 4.11 (d, 1H, *J* = 7.4 Hz), 3.85 (dd, 1H, *J* = 10.9, 5.7 Hz), 3.78 (dd, 1H, *J* = 10.9, 6.1 Hz), 3.28 – 3.21 (m, 2H), 3.10 – 2.99 (m, 2H), 2.90 (dd, 2H, *J* = 8.9, 7.6 Hz), 2.84 (dd, 1H, *J* = 14.0, 8.5 Hz), 2.40 – 2.23 (m, 2H), 2.10 – 1.97 (m, 1H), 1.90 (s, 3H), 1.89 – 1.79 (m, 3H), 1.77 – 1.69 (m, 1H), 1.67 – 1.58 (m, 2H), 1.55 – 1.40 (m, 3H), 1.23 – 1.09 (m, 1H), 0.96 – 0.86 (m, 6H); ^{19}F NMR (376 MHz, DMSO-*d*$_6$) δ −73.5 (s, TFA), −96.7 (d, 1F, *J* = 267 Hz), −98.1 (d, 1F, *J* = 267 Hz); HRMS (ESI) m/z: [M + H]$^+$ Calcd for $C_{56}H_{73}F_2N_{14}O_{13}$ 1187.5450; Found 1187.5459 (0.8 ppm error).

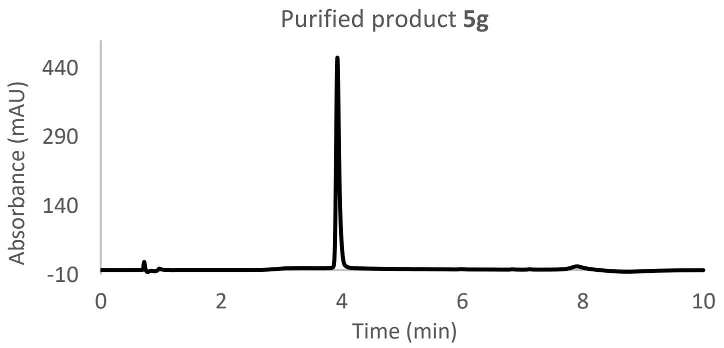

Figure S32. Analytical HPLC chromatogram for purified product **5g** Rt = 3.93 min on analytical gradient 2-95% B over 5 mins 0.6 mL min^{-1}, 280 nm.

Ac-WHISKEY-NH$_2$ conjugated with 2-bromo-*N*-benzyl-2,2-difluoroacetamide (5h)

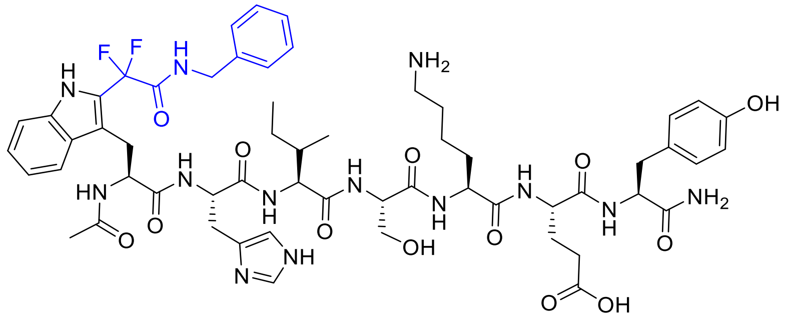

Prepared according to general procedure E using Ac-WHISKEY-NH$_2$ **4** (25.0 mM in DMSO, 200 µL, 5.00 µmol, 1.00 eq.), 4CzIPN (2.50 mM in DMSO, 20 µL, 0.0500 µmol, 1 mol%) and 2-bromo-*N*-benzyl-2,2-difluoroacetamide **2h** (0.1 M in DMSO, 100 µL, 10.0 µmol, 2.00 eq.). The reaction mixture was irradiated with blue LEDs for 60 min, then was analysed by quantitative ^{19}F NMR spectroscopy (28% yield). The reaction mixture was then purified by semi-preparative RP-HPLC (2-70% B over 30 min) to give the title compound **5h** (1.90 mg, 1.60 µmol, 32%) as a colourless powder; ^{1}H NMR (500 MHz, CD_3OD) δ 8.67 (s, 1H), 7.67 (d, 1H, *J* = 8.1 Hz), 7.39 (d, 1H, *J* = 8.2 Hz), 7.34 – 7.18 (m, 7H), 7.12 – 7.01 (m, 3H), 6.68 (d, 2H, *J* = 8.4 Hz), 4.63 – 4.54 (m, 2H), 4.53 – 4.46 (m, 3H), 4.43 (dd, 1H, *J* = 6.2, 5.8 Hz), 4.29 (dd, 2H, *J* = 8.8, 5.5 Hz), 4.11 (d, 1H, *J* = 7.3 Hz), 3.87 (dd, 1H, *J* = 10.9, 5.8 Hz), 3.80 (dd, 1H, *J* = 10.9, 6.2 Hz), 3.28 – 3.21 (m, 1H), 3.10 – 3.01 (m, 2H), 2.93 – 2.81 (m, 3H), 2.39 – 2.24 (m, 2H), 2.06 – 1.91 (m, 2H), 1.92 – 1.81 (m, 5H), 1.77 – 1.69 (m, 1H), 1.69 – 1.60 (m, 2H), 1.56 – 1.48 (m, 1H), 1.49 – 1.40 (m, 2H), 1.24 – 1.11 (m, 1H), 0.96 – 0.88 (m, 6H); ^{19}F NMR (376 MHz, DMSO-*d*$_6$) δ −73.4 (s, TFA), −96.6 (d, 1F, *J* = 266 Hz), −98.0 (d, 1F, *J* = 266 Hz); HRMS (ESI) m/z: $[M + H]^+$ Calcd for $C_{57}H_{74}F_2N_{13}O_{13}$1186.5497; Found 1186.5465 (2.7 ppm error).

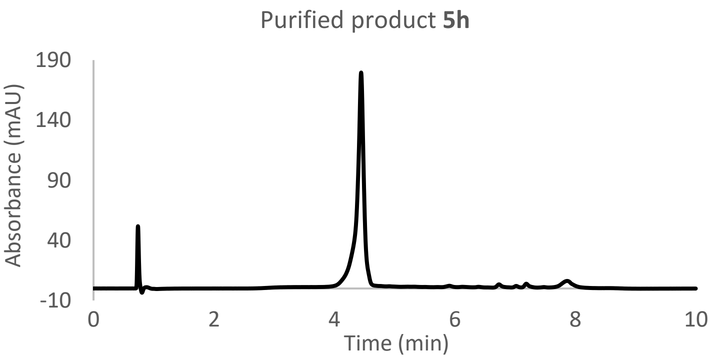

Figure S33. Analytical HPLC chromatogram for purified product **5h** Rt = 4.44 min on analytical gradient 2-95% B over 5 min, 0.6 mL min^{-1}, 280 nm.

Ac-WHISKEY-NH$_2$ conjugated with 2-bromo-*N*-cyclopropyl-2,2-difluoroacetamide (5i)

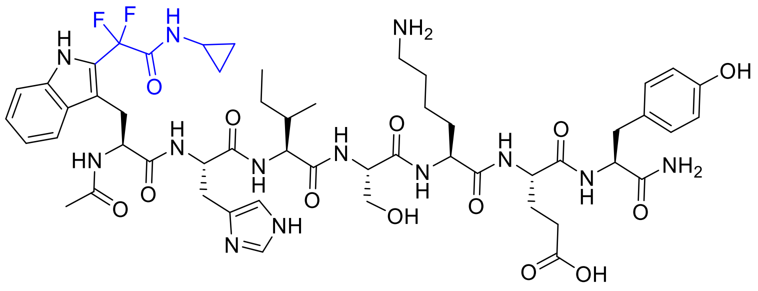

Prepared according to general procedure E using Ac-WHISKEY-NH$_2$ **4** (25.0 mM in DMSO, 200 µL, 5.00 µmol, 1.00 eq.), 4CzIPN (2.50 mM in DMSO, 20 µL, 0.0500 µmol, 1 mol%) and 2-bromo-*N*-cyclopropyl-2,2-difluoroacetamide **2i** (0.1 M in DMSO, 100 µL, 10.0 µmol, 2.00 eq.). The reaction mixture was irradiated with blue LEDs for 60 min, then was analysed by quantitative ^{19}F NMR spectroscopy (31% yield). The reaction mixture was then purified by semi-preparative RP-HPLC (2-70% B over 30 min) to give the title compound **5i** (2.10 mg, 1.85 µmol, 37%) as a colourless powder; ^{1}H NMR (500 MHz, CD$_3$OD) δ 8.71 (d, 1H, *J* = 1.4 Hz), 7.65 (d, 1H, *J* = 8.1 Hz), 7.37 (d, 1H, *J* = 8.3 Hz), 7.28 (d, 1H, *J* = 1.4 Hz), 7.21 (ddd, 1H, *J* = 8.3, 7.0, 1.1 Hz), 7.11 – 7.00 (m, 3H), 6.69 (d, 2H, *J* = 8.5 Hz), 4.61 – 4.53 (m, 2H), 4.50 (dd, 1H, *J* = 8.3, 5.7 Hz), 4.43 (dd, 1H, *J* = 6.1, 5.6 Hz), 4.29 (dd, 2H, *J* = 8.9, 5.5 Hz), 4.16 – 4.05 (m, 1H), 3.86 (dd, 1H, *J* = 10.9, 5.6 Hz), 3.79 (dd, 1H, *J* = 10.9, 6.1 Hz), 3.30 – 3.23 (m, 3H), 3.10 – 3.01 (m, 2H), 2.90 (dd, 2H, *J* = 8.7, 7.5 Hz), 2.88 – 2.80 (m, 2H), 2.40 – 2.25 (m, 2H), 2.09 – 1.97 (m, 1H), 1.95 (s, 3H), 1.93 – 1.81 (m, 3H), 1.77 – 1.69 (m, 1H), 1.66 (dd, 2H, *J* = 8.7, 7.4 Hz), 1.57 – 1.49 (m, 1H), 1.49 – 1.39 (m, 2H), 1.25 – 1.12 (m, 1H), 0.98 – 0.87 (m, 6H), 0.85 – 0.73 (m, 2H), 0.73 – 0.61 (m, 2H); ^{19}F NMR (376 MHz, DMSO) δ −73.5 (s, TFA), −96.2 (d, 1F, *J* = 266 Hz), −98.0 (d, 1F, *J* = 266 Hz); HRMS (ESI) m/z: [M + H]$^+$ Calcd for $C_{53}H_{72}F_2N_{13}O_{13}$ 1136.5341; Found 1136.5301 (3.5 ppm error).

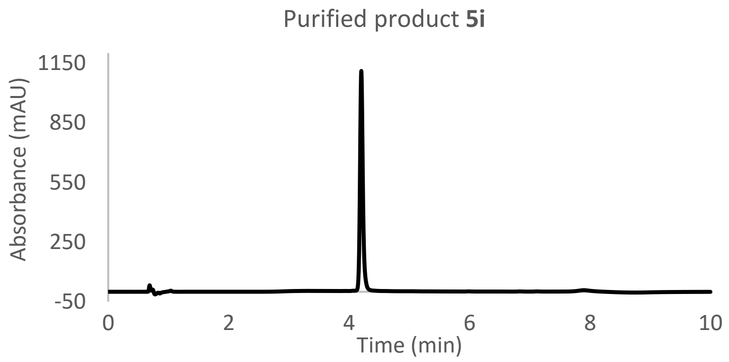

Figure S34. Analytical HPLC chromatogram for purified product **5i** Rt = 4.20 min on analytical gradient 2-95% B over 5 min, 0.6 mL min^{-1}, 280 nm.

Ac-WHISKEY-NH$_2$ conjugated with 2-bromo-*N*-(*tert*-butyl)-2,2-difluoroacetamide (5j)

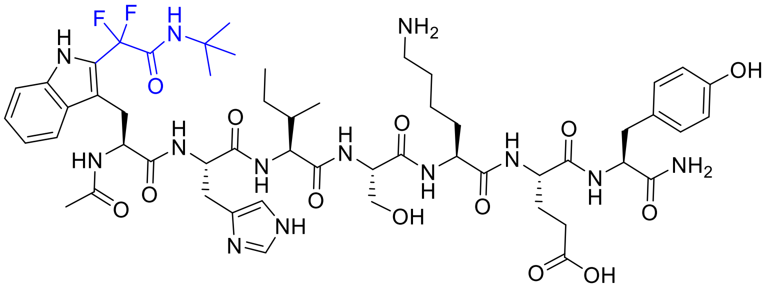

Prepared according to general procedure E using Ac-WHISKEY-NH$_2$ **4** (25.0 mM in DMSO, 200 µL, 5.00 µmol, 1.00 eq.), 4CzIPN (2.50 mM in DMSO, 20 µL, 0.0500 µmol, 1 mol%) and 2-bromo-*N*-(*tert*-butyl)-2,2-difluoroacetamide **2j** (0.1 M in DMSO, 100 µL, 10.0 µmol, 2.00 eq.). The reaction mixture was irradiated with blue LEDs for 75 min, then was analysed by quantitative ^{19}F NMR spectroscopy (33% yield). The reaction mixture was purified by semi-preparative RP-HPLC (2-70% B over 30 min) to give the title compound **5j** (2.1 mg, 1.82 µmol, 36%) as a colourless powder; ^{1}H NMR (500 MHz, CD$_3$OD) δ 8.63 (s, 1H), 7.67 (d, 1H, *J* = 8.1 Hz), 7.38 (d, 1H, *J* = 8.2 Hz), 7.26 (s, 1H), 7.22 (dd, 1H, *J* = 8.2, 7.6 Hz), 7.13 – 7.00 (m, 3H), 6.69 (d, 2H, *J* = 8.5 Hz), 4.63 – 4.57 (m, 1H), 4.55 (dd, 1H, *J* = 8.3, 7.5 Hz), 4.50 (dd, 1H, *J* = 8.5, 5.7 Hz), 4.41 (dd, 1H, *J* = 6.0, 5.6 Hz), 4.32 – 4.25 (m, 2H), 4.12 (d, 1H, *J* = 7.4 Hz), 3.87 (dd, 1H, *J* = 10.9, 5.6 Hz), 3.81 (dd, 1H, *J* = 10.9, 6.0 Hz), 3.34 – 3.32 (m, 2H), 3.27 – 3.20 (m, 1H), 3.11 – 3.03 (m, 2H), 2.94 – 2.88 (m, 2H), 2.85 (dd, 1H, *J* = 13.9, 8.5 Hz), 2.40 – 2.25 (m, 2H), 2.06 – 1.98 (m, 1H), 1.96 (s, 3H), 1.93 – 1.82 (m, 3H), 1.79 – 1.70 (m, 1H), 1.70 – 1.61 (m, 2H), 1.52 – 1.44 (m, 2H), 1.42 (s, 9H), 1.39 – 1.35 (m, 1H), 1.22 – 1.13 (m, 1H), 0.98 – 0.89 (m, 6H); ^{19}F NMR (377 MHz, CD$_3$OD) δ −76.9 (s, TFA), −100.4 (d, 1F, *J* = 266 Hz), −102.1 (d, 1F, *J* = 266 Hz); HRMS (ESI) m/z: [M + H]$^+$ Calcd for $C_{54}H_{76}F_2N_{13}O_{13}$ 1152.5648; Found 1152.5646 (0.2 ppm error).

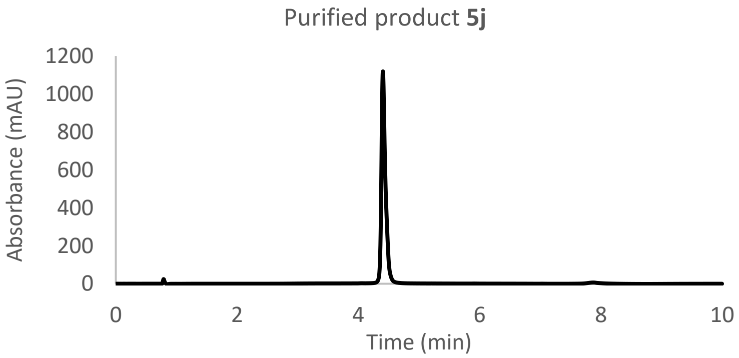

Figure S35. Analytical HPLC chromatogram for purified product **5j** Rt = 4.41 min on analytical gradient 2-95% B over 5 min, 0.6 mL min^{-1}, 280 nm.

Ac-WHISKEY-NH_2 conjugated with *N*-(2-(2-bromo-2,2-difluoroacetamido)ethyl)-5-((3a*S*,4*S*,6a*R*)-2-oxohexahydro-1*H*-thieno[3,4-d]imidazol-4-yl)pentanamide (5k)

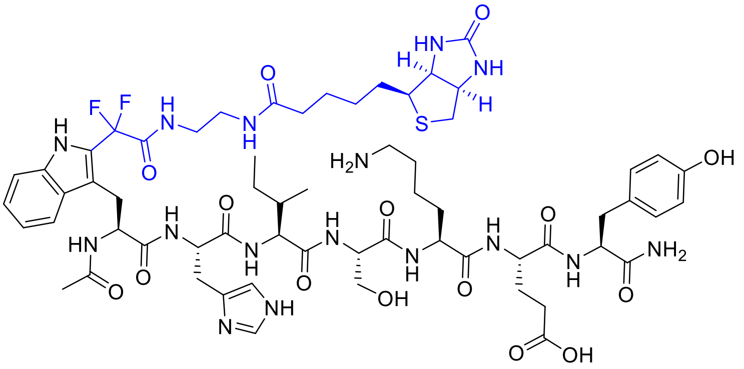

Prepared according to general procedure E using Ac-WHISKEY-NH_2 **4** (25.0 mM in DMSO, 200 µL, 5.00 µmol, 1.00 eq.), 4CzIPN (12.5 mM in DMSO, 100 µL, 0.250 µmol, 5 mol%) and *N*-(2-(2-bromo-2,2-difluoroacetamido)ethyl)-5-((3a*S*,4*S*,6a*R*)-2-oxohexahydro-1*H*-thieno[3,4-d]imidazol-4-yl)pentanamide **2k** (0.1 M in DMSO, 75 µL, 7.50 µmol, 1.50 eq.). The reaction mixture was irradiated with blue LEDs for 120 min, then was analysed by quantitative ^{19}F NMR spectroscopy (19% yield). The reaction mixture was then purified by semi-preparative RP-HPLC (2-70% B over 30 min with an isocratic hold at 23% B) to give the title compound **5k** (1.20 mg, 0.89 µmol, 18%) as a colourless powder; ^{1}H NMR (400 MHz, CD_3OD) δ 8.70 (s, 1H), 7.68 (d, 1H, *J* = 8.1 Hz), 7.40 (d, 1H, *J* = 8.3 Hz), 7.29 (s, 1H), 7.22 (dd, 1H, *J* = 8.3, 7.6 Hz), 7.13 – 7.03 (m, 3H), 6.69 (d, 2H, *J* = 8.5 Hz), 4.64 – 4.55 (m, 2H), 4.53 – 4.46 (m, 2H), 4.46 – 4.40 (m, 1H), 4.35 – 4.22 (m, 3H), 4.12 (d, 1H, *J* = 7.3 Hz), 3.87 (dd, 1H, *J* = 10.8, 5.6 Hz), 3.80 (dd, 1H, *J* = 10.8, 5.9 Hz), 3.46 – 3.36 (m, 7H), 3.11 – 3.02 (m, 3H), 2.95 – 2.89 (m, 2H), 2.90 – 2.81 (m, 2H), 2.69 (d, 1H, *J* = 12.9 Hz), 2.37 – 2.28 (m, 2H), 2.18 – 2.06 (m, 2H), 2.01 – 1.97 (m, 1H), 1.93 (s, 3H), 1.91 – 1.80 (m, 3H), 1.75 – 1.62 (m, 3H), 1.58 – 1.38 (m, 7H), 1.36 – 1.25 (m, 2H), 1.23 – 1.03 (m, 1H), 0.98 – 0.90 (m, 6H); ^{19}F NMR (376 MHz, CD_3OD) δ −77.0 (s, TFA), −101.6 (s, 2F); HRMS (ESI) m/z: $[M + 2H]^{2+}$ Calcd for $C_{62}H_{88}F_2N_{16}O_{15}S$ 683.3152; Found 683.3148 (0.6 ppm error).

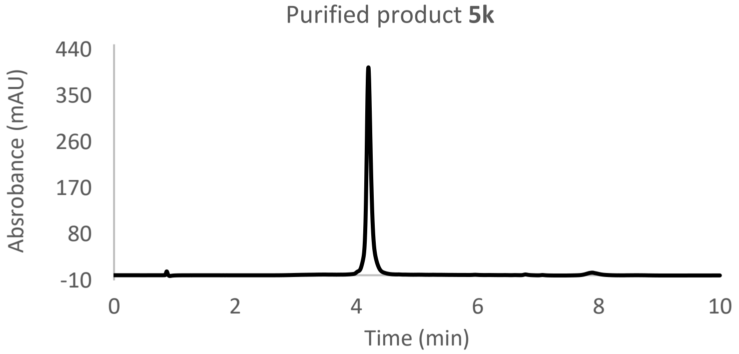

Figure S36. Analytical HPLC chromatogram for purified product **5k** Rt = 4.20 min on analytical gradient 2-95% B over 5 mins,0.6 mL min^{-1}, 280 nm.

Ac-WHISKEY-NH$_2$ conjugated with *N*-((2*R*,3*R*,4*R*,5*S*,6*R*)-3-acetamido-4,5-dihydroxy-6-(hydroxymethyl)tetrahydro-2*H*-pyran-2-yl)-2-bromo-2,2-difluoroacetamide (5l)

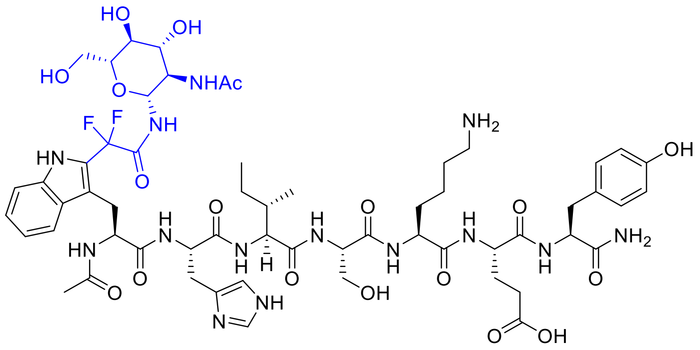

Prepared according to general procedure E using Ac-WHISKEY-NH$_2$ **4** (25.0 mM in DMSO, 200 µL, 5.00 µmol, 1.00 eq.), 4CzIPN (2.50 mM in DMSO, 20 µL, 0.0500 µmol, 1 mol%) and *N*-((2*R*,3*R*,4*R*,5*S*,6*R*)-3-acetamido-4,5-dihydroxy-6-(hydroxymethyl) tetrahydro-2*H*-pyran-2-yl)-2-bromo-2,2-difluoroacetamide **2l** (0.1 M in DMSO, 100 µL, 10.0 µmol, 2.00 eq.). The reaction mixture was irradiated with blue LEDs for 90 min, then was analysed by quantitative ^{19}F NMR spectroscopy (31% yield). The reaction mixture was purified by semi-preparative RP-HPLC (2-70% B over 30 min) to give the title compound **5l** (2.50 mg, 1.92 µmol, 38%) as a colourless powder; ^{1}H NMR (500 MHz, CD$_3$OD) δ 8.61 (s, 1H), 7.64 (d, 1H, *J* = 8.2 Hz), 7.38 (d, 1H, *J* = 8.3 Hz), 7.24 (s, 1H), 7.21 (dd, 1H, *J* = 8.3, 7.6 Hz), 7.13 – 7.00 (m, 3H), 6.69 (d, 1H, *J* = 8.5 Hz), 5.05 (d, 1H, *J* = 9.7 Hz), 4.62 – 4.52 (m, 2H), 4.54 – 4.45 (m, 1H), 4.41 (dd, 1H, *J* = 6.6, 5.7 Hz), 4.35 – 4.17 (m, 2H), 4.11 (d, 1H, *J* = 7.3 Hz), 3.92 – 3.77 (m, 4H), 3.75 – 3.62 (m, 1H), 3.58 – 3.48 (m, 1H), 3.38 – 3.33 (m, 3H), 3.26 – 3.19 (m, 2H), 3.12 – 2.99 (m, 2H), 2.98 – 2.79 (m, 3H), 2.40 – 2.25 (m, 2H), 2.02 – 1.97 (m, 1H), 1.91 (s, 3H), 1.88 – 1.79 (m, 3H), 1.76 (s, 3H), 1.74 – 1.68 (m, 1H), 1.68 – 1.59 (m, 2H), 1.55 – 1.47 (m, 1H), 1.46 – 1.38 (m, 2H), 1.23 – 1.07 (m, 1H), 0.98 – 0.87 (m, 6H); ^{19}F NMR (376 MHz, CD$_3$OD) δ −77.0 (s, TFA), −102.1 (d, 1F, *J* = 267 Hz), −103.5 (d, 1F, *J* = 267 Hz); HRMS (ESI) m/z: [M + H]$^+$ Calcd for $C_{58}H_{81}F_2N_{14}O_{18}$ 1299.5821; Found 1299.5795 (2.1 ppm error).

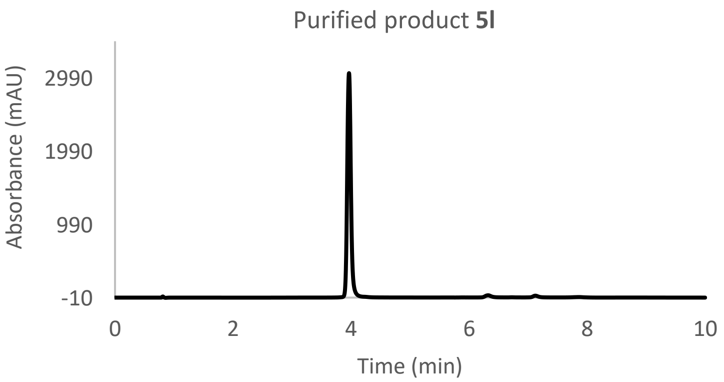

Figure S37. Analytical HPLC chromatogram for purified product **5l** Rt = 3.97 min on analytical gradient 2-95% B over 5 mins,0.6 mL min^{-1}, 280 nm.

Ac-HISKEY-NH$_2$ control using ethyl bromodifluoroacetate (S3)

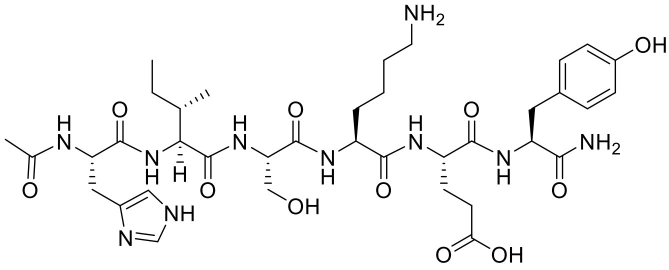

Prepared according to general procedure E using Ac-HISKEY-NH$_2$ **6** (25.0 mM in DMSO, 200 µL, 5.00 µmol, 1.00 eq.), 4CzIPN (2.50 mM in DMSO, 20 µL, 0.0500 µmol, 1 mol%) and ethyl bromodifluoroacetate **2a** (0.100 M in DMSO, 100 µL, 10.0 µmol, 2.00 eq.). The reaction mixture was irradiated with blue LEDs for 10 min, then was analysed by quantitative ^{19}F NMR spectroscopy (0% yield).

H-WHISKEY-OH conjugated with ethyl bromodifluoroacetate (13)

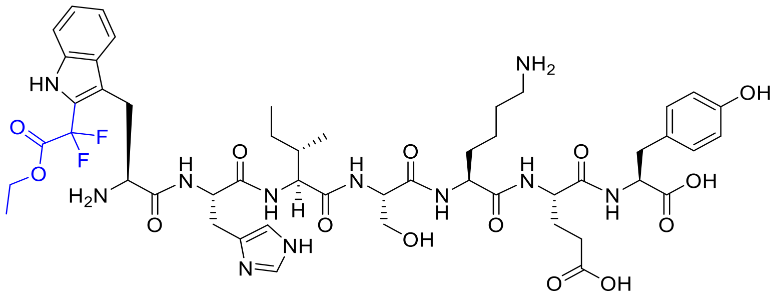

Prepared according to general procedure E using H-WHISKEY-OH **7** (25.0 mM in DMSO, 200 µL, 5.00 µmol, 1.00 eq.), 4CzIPN (2.50 mM in DMSO, 20 µL, 0.0500 µmol, 1 mol%) and ethyl bromodifluoroacetate **2a** (0.100 M in DMSO, 100 µL, 10.0 µmol, 2.00 eq.). The reaction mixture was irradiated with blue LEDs for 10 min, then was analysed by quantitative ^{19}F NMR spectroscopy (38% yield). The reaction mixture was then purified by semi-preparative RP-HPLC (2-70% B over 30 min) to give the title compound **13** (1.90 mg, 1.75 µmol, 35%) as a colourless powder; ^{1}H NMR (500 MHz, CD_3OD) δ 8.62 (s, 1H), 7.59 (d, 1H, *J* = 8.0 Hz), 7.40 (d, 1H, *J* = 8.3 Hz), 7.27 – 7.20 (m, 2H), 7.11 (dd, 1H, *J* = 8.0, 7.0 Hz), 7.06 (d, 2H, *J* = 8.5 Hz), 6.69 (d, 2H, *J* = 8.5 Hz), 4.53 – 4.42 (m, 3H), 4.38 – 4.26 (m, 4H), 4.22 (dd, 1H, *J* = 9.0, 6.9 Hz), 3.98 (d, 1H, *J* = 6.8 Hz), 3.81 (dd, 1H, *J* = 10.9, 5.6 Hz), 3.76 (dd, 1H, *J* = 10.9, 6.1 Hz), 3.45 – 3.36 (m, 2H), 3.23 – 3.16 (m, 1H), 3.11 – 3.01 (m, 2H), 2.94 – 2.82 (m, 3H), 2.40 – 2.25 (m, 2H), 2.08 – 1.97 (m, 1H), 1.91 – 1.82 (m, 2H), 1.80 – 1.74 (m, 1H), 1.73 – 1.61 (m, 3H), 1.56 – 1.48 (m, 1H), 1.47 – 1.38 (m, 2H), 1.29 (t, 3H, *J* = 7.1 Hz), 1.22 – 1.11 (m, 1H), 1.01 – 0.89 (m, 6H); ^{19}F NMR (376 MHz, CD_3OD) δ −76.9 (s, TFA), −101.9 (d, 1F, *J* = 266 Hz), −104.3 (d, 1F, *J* = 266 Hz); HRMS (ESI) m/z: $[M + H]^+$ Calcd for $C_{50}H_{68}F_2N_{11}O_{14}$ 1084.4915; Found 1084.4980 (6.0 ppm error).

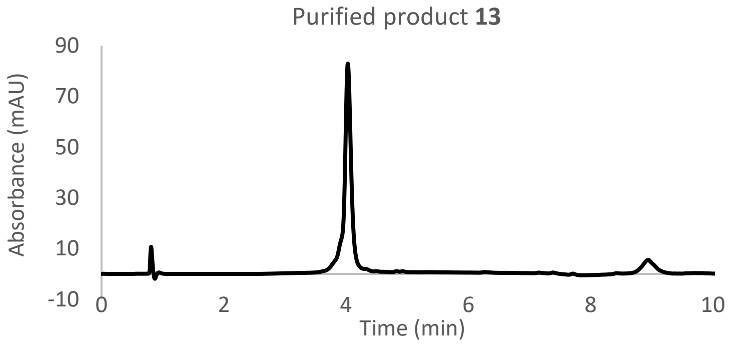

Figure S38. Analytical HPLC chromatogram for purified product **13** Rt = 4.03 min on analytical gradient 10-80% B over 5 min, 0.6 mL min^{-1}, 280 nm.

Ac-CWHISKEY-NH_2 conjugated with ethyl bromodifluoroacetate (14)

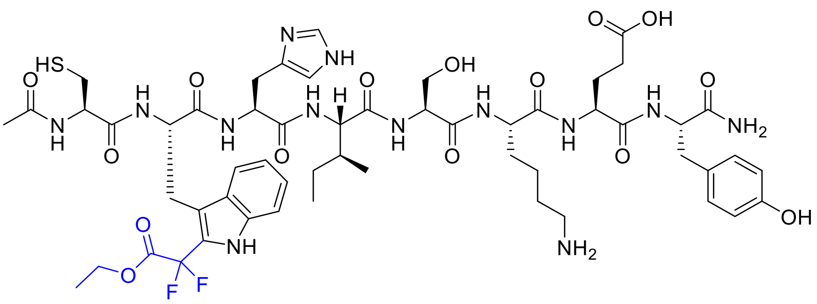

Prepared according to general procedure E using Ac-CWHISKEY-NH_2 **8** (25.0 mM in DMSO, 200 µL, 5.00 µmol, 1.00 eq.), 4CzIPN (2.50 mM in DMSO, 20 µL, 0.0500 µmol, 1 mol%) and ethyl bromodifluoroacetate **2a** (0.100 M in DMSO, 100 µL, 10.0 µmol, 2.00 eq.). The reaction mixture was irradiated with blue LEDs for 10 min, then was analysed by quantitative ^{19}F NMR spectroscopy (36% yield). The reaction mixture was then purified by semi-preparative RP-HPLC (2-70% B over 30 min) to give the title compound **14** (2.00 mg, 1.62 µmol, 33%) as a colourless powder; ^{1}H NMR (500 MHz, CD_3OD) δ 8.71 (s, 1H), 7.60 (d, 1H, *J* = 8.2 Hz), 7.39 (d, 1H, *J* = 8.3 Hz), 7.30 (s, 1H), 7.23 (dd, 1H, *J* = 8.3, 7.6 Hz), 7.12 – 7.02 (m, 3H), 6.69 (d, 2H, *J* = 8.5 Hz), 4.59 – 4.47 (m, 3H), 4.43 – 4.37 (m, 1H), 4.37 – 4.31 (m, 3H), 4.30 – 4.22 (m, 2H), 4.08 (d, 1H, *J* = 7.5 Hz), 3.86 (dd, 1H, *J* = 11.0, 5.7 Hz), 3.80 (dd, 1H, *J* = 11.0, 5.8 Hz), 3.43 – 3.35 (m, 2H), 3.28 – 3.23 (m, 1H), 3.14 – 3.03 (m, 2H), 2.94 – 2.81 (m, 3H), 2.77 – 2.68 (m, 2H), 2.40 – 2.24 (m, 2H), 2.06 – 2.00 (m, 1H), 1.99 (s, 3H), 1.93 – 1.80 (m, 3H), 1.77 – 1.68 (m, 1H), 1.68 – 1.60 (m, 2H), 1.58 – 1.51 (m, 1H), 1.48 – 1.40 (m, 2H), 1.30 (t, 3H, *J* = 7.1 Hz), 1.24 – 1.16 (m, 1H), 0.97 – 0.88 (m, 6H); ^{19}F NMR (376 MHz, CD_3OD) δ −77.0 (s, TFA), −100.9 (d, 1F, *J* = 266 Hz), −102.2 (1F, d, *J* = 266 Hz); HRMS (ESI) m/z: $[M]^+$ Calcd for $C_{55}H_{74}F_2N_{12}O_{16}S$: 1228.5035; Found 1228.5118 (6.8 ppm error).

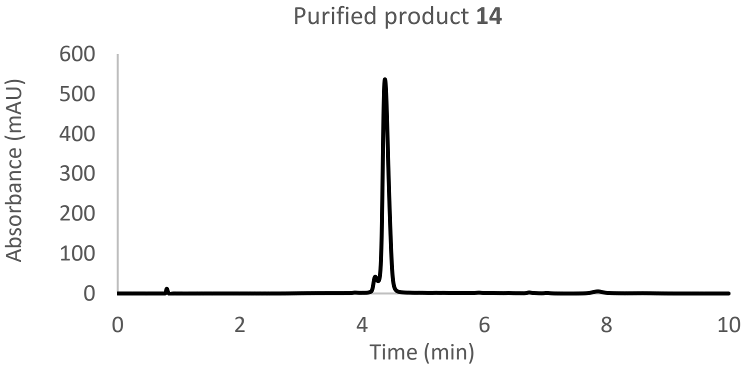

Figure S39. Analytical HPLC chromatogram for purified product **14** Rt = 4.37 min on analytical gradient 2-95% B over 5 min, 0.6 mL min^{-1}, 280 nm.

Ac-WFMTREY-NH2 conjugated with ethyl bromodifluoroacetate (15)

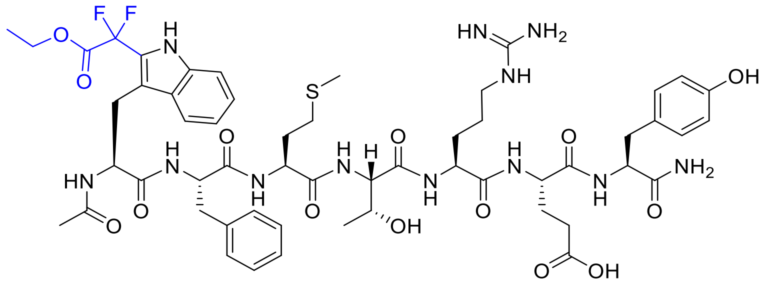

Prepared according to general procedure E using Ac-WFMTREY-NH2 **9** (25.0 mM in DMSO, 200 µL, 5.00 µmol, 1.00 eq.), 4CzIPN (2.50 mM in DMSO, 20 µL, 0.0500 µmol, 1 mol%) and ethyl bromodifluoroacetate **2a** (0.100 M in DMSO, 100 µL, 10.0 µmol, 2.00 eq.). The reaction mixture was irradiated with blue LEDs for 10 min, then was analysed by quantitative ^{19}F NMR spectroscopy (49% yield). The reaction mixture was then purified by semi-preparative RP-HPLC (2-70% B over 30 min) to give the title compound **15** (2.40 mg, 2.01 µmol, 40%) as a colourless powder; ^{1}H NMR (400 MHz, CD_3OD) δ 7.52 (d, 1H, *J* = 8.1 Hz), 7.39 (d, 1H, *J* = 8.3 Hz), 7.35 – 7.27 (m, 2H), 7.27 – 7.17 (m, 4H), 7.12 – 7.02 (m, 3H), 6.69 (d, 2H, *J* = 8.6 Hz), 4.50 (dd, 1H, *J* = 9.0, 5.1 Hz), 4.44 (dd, 1H, *J* = 8.1, 7.5 Hz), 4.40 – 4.28 (m, 4H), 4.27 – 4.06 (m, 4H), 3.18 – 2.95 (m, 6H), 2.86 (dd, 1H, *J* = 14.0, 9.2 Hz), 2.59 – 2.37 (m, 2H), 2.36 – 2.22 (m, 2H), 2.14 – 2.03 (m, 2H), 2.09 (s, 3H), 2.03 – 1.91 (m, 3H), 1.91 – 1.83 (m, 4H), 1.80 – 1.59 (m, 2H), 1.59 – 1.48 (m, 2H), 1.30 (t, 3H, *J* = 7.1 Hz), 1.17 (d, 3H, *J* = 5.7 Hz); ^{19}F NMR (376 MHz, CD_3OD) δ −77.1 (s, TFA), −101.4 (d, 1F, *J* = 266 Hz), −102.4 (d, 1F, *J* = 266 Hz); HRMS (ESI) m/z: [M + 2H]$^{2+}$ Calcd for $C_{55}H_{74}F_2N_{12}O_{14}S$ 598.2568; Found 598.2576 (1.3 ppm error).

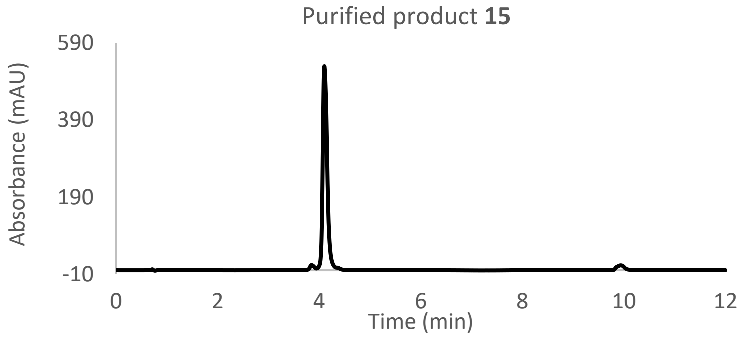

Figure S40. Analytical HPLC chromatogram for purified product **15** Rt = 4.10 min on analytical gradient 20-100% B over 5 min, 0.6 mL min^{-1}, 280 nm.

Ac-WLAHKAL-NH2 conjugated with ethyl bromodifluoroacetate (16)

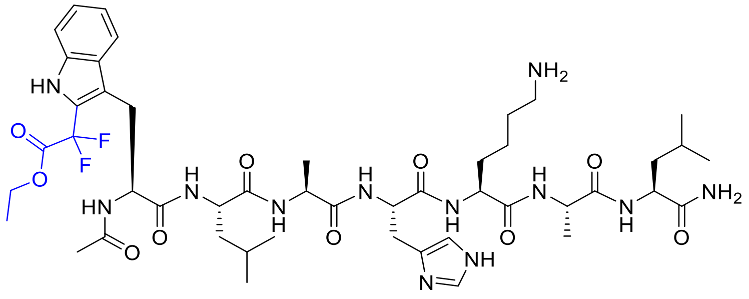

Prepared according to general procedure E using Ac-WLAHKAL-NH2 **10** (25.0 mM in DMSO, 200 µL, 5.00 µmol, 1.00 eq.), 4CzIPN (2.50 mM in DMSO, 20 µL, 0.0500 µmol, 1 mol%) and ethyl bromodifluoroacetate **2a** (0.100 M in DMSO, 100 µL, 10.0 µmol, 2.00 eq.). The reaction mixture was irradiated with blue LEDs for 10 min, then was analysed by quantitative ^{19}F NMR spectroscopy (41% yield). The reaction mixture was then purified by semi-preparative RP-HPLC (2-70% B over 30 min) to give the title compound **16** (2.00 mg, 2.00 µmol, 40%) as a colourless powder; ^{1}H NMR (500 MHz, CD_3OD) δ 8.69 (s, 1H), 7.62 (d, 1H, *J* = 8.0 Hz), 7.40 (d, 1H, *J* = 8.2 Hz), 7.28 (s, 1H), 7.23 (ddd, 1H, *J* = 8.2, 7.0, 1.1 Hz), 7.07 (ddd, 1H, *J* = 8.0, 7.0, 1.0 Hz), 4.49 – 4.41 (m, 2H), 4.39 – 4.31 (m, 3H), 4.27 (q, 1H, *J* = 7.2 Hz), 4.22 – 4.02 (m, 3H), 3.43 – 3.33 (m, 2H), 3.23 (dd, 1H, *J* = 15.4, 5.7 Hz), 3.10 (dd, 1H, *J* = 15.4, 8.2 Hz), 2.91 (dd, 2H, *J* = 8.2, 7.5 Hz), 2.00 (s, 3H), 1.84 – 1.75 (m, 1H), 1.74 – 1.53 (m, 9H), 1.44 – 1.38 (m, 5H), 1.36 (d, 3H, *J* = 7.2 Hz), 1.30 (t, 3H, *J* = 7.1 Hz), 0.98 – 0.89 (m, 12H); ^{19}F NMR (376 MHz, CD_3OD) δ −77.0 (s, TFA), −101.4 (d, 1F, *J* = 265 Hz), −102.5 (d, 1F, *J* = 265 Hz); HRMS (ESI) m/z: $[M + H]^+$ Calcd for $C_{47}H_{71}F_2N_{12}O_{10}$ 1001.5384; Found 1001.5333 (5.1 ppm error).

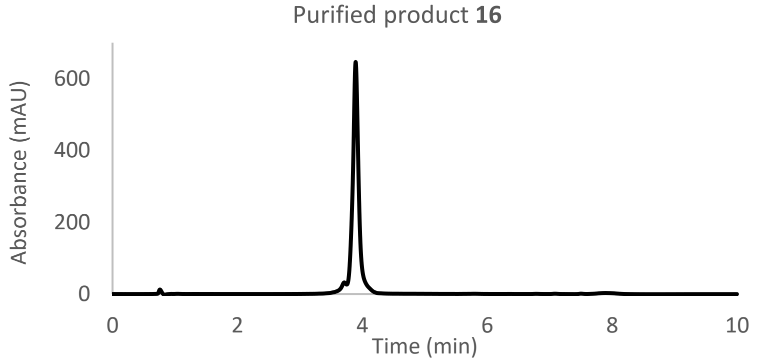

Figure S41. Analytical HPLC chromatogram for purified product **16** Rt = 3.89 min on analytical gradient 20-80% B over 5 min, 0.6 mL min^{-1}, 280 nm.

Ac-VVYPWYQ-NH2 conjugated with ethyl bromodifluoroacetate (17)

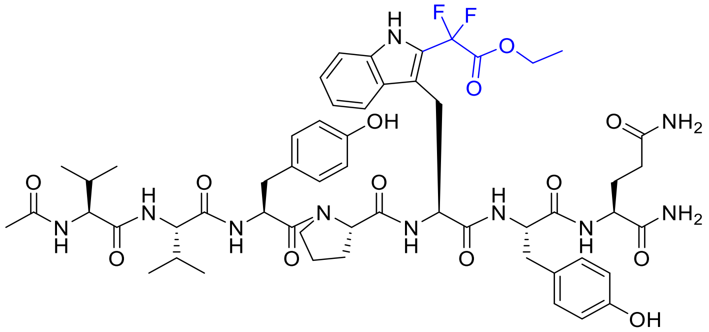

Prepared according to general procedure E using Ac-VVYPWYQ-NH2 **11** (25.0 mM in DMSO, 200 µL, 5.00 µmol, 1.00 eq.), 4CzIPN (2.50 mM in DMSO, 20 µL, 0.0500 µmol, 1 mol%) and ethyl bromodifluoroacetate **2a** (0.100 M in DMSO, 100 µL, 10.0 µmol, 2.00 eq.). The reaction mixture was irradiated with blue LEDs for 15 min, then was analysed by quantitative ^{19}F NMR spectroscopy (42% yield). The reaction mixture was then purified by semi-preparative RP-HPLC (2-70% B over 30 min) to give the title compound **17** (2.40 mg, 2.15 µmol, 43%) as a colourless powder; ^{1}H NMR (500 MHz, DMSO-d_6) δ 11.63 (s, 1H), 9.13 (d, 2H, *J* = 6.6 Hz), 8.08 (d, 1H, *J* = 7.9 Hz), 7.88 (d, 2H, *J* = 8.4 Hz), 7.75 (d, 1H, *J* = 8.0 Hz), 7.63 (d, 1H, *J* = 9.0 Hz), 7.50 (d, 1H, *J* = 7.3 Hz), 7.45 (d, 1H, *J* = 8.0 Hz), 7.37 (d, 1H, *J* = 8.2 Hz), 7.23 – 7.14 (m, 2H), 7.06 – 6.98 (m, 5H), 6.98 – 6.91 (m, 1H), 6.75 (s, 1H), 6.69 – 6.56 (m, 4H), 6.54 (s, 1H), 4.68 – 4.57 (m, 1H), 4.41 – 4.28 (m, 3H), 4.27 – 4.21 (m, 1H), 4.19 – 4.09 (m, 3H), 4.08 – 4.00 (m, 1H), 3.63 – 3.53 (m, 1H), 3.53 – 3.46 (m, 1H), 3.25 – 3.17 (m, 1H), 3.13 – 3.04 (m, 1H), 3.01 – 2.93 (m, 1H), 2.92 – 2.79 (m, 2H), 2.75 – 2.66 (m, 1H), 2.14 – 1.99 (m, 2H), 1.92 – 1.80 (m, 7H), 1.79 – 1.59 (m, 3H), 1.60 – 1.50 (m, 1H), 1.22 (t, 3H, *J* = 7.1 Hz), 0.79 – 0.68 (m, 12H); ^{19}F NMR (376 MHz, DMSO-d_6) δ −73.6 (s, TFA), −98.6 (s, 2F); HRMS (ESI) m/z: $[M + H]^+$ Calcd for $C_{55}H_{71}F_2N_{10}O_{13}$ 1117.5170; Found 1117.5201 (2.8 ppm error).

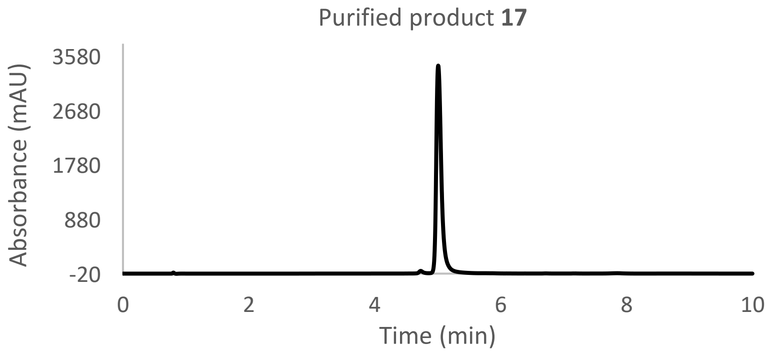

Figure S42. Analytical HPLC chromatogram for purified product **17** Rt = 5.00 min on analytical gradient 2-95% B over 5 min, 0.6 mL min^{-1}, 280 nm.

Ac-DKVGINYW-NH₂ conjugated with ethyl bromodifluoroacetate (18)

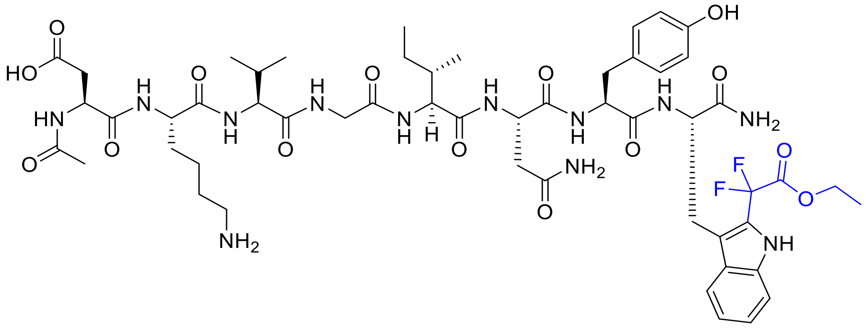

Prepared according to general procedure D using Ac-DKVGINYW-NH_2 **12** (25.0 mM in DMSO, 200 µL, 5.00 µmol, 1.00 eq.), 4CzIPN (2.50 mM in DMSO, 20 µL, 0.0500 µmol, 1 mol%) and ethyl bromodifluoroacetate **2a** (0.100 M in DMSO, 100 µL, 10.0 µmol, 2.00 eq.). The reaction mixture was irradiated with blue LEDs for 10 min, then was analysed by quantitative ^{19}F NMR spectroscopy (49% yield). The reaction mixture was then purified by semi-preparative RP-HPLC (2-70% B over 30 min) to give the title compound **18** (2.60 mg, 2.25 µmol, 45%) as a colourless powder; ^{1}H NMR (400 MHz, CD_3OD) δ 7.85 (d, 1H, *J* = 8.0 Hz), 7.41 (d, 1H, *J* = 8.2 Hz), 7.24 (dd, 1H, *J* = 8.2, 7.6 Hz), 7.15 (dd, 1H, *J* = 8.0, 7.6 Hz), 6.68 (d, 2H, *J* = 8.4 Hz), 6.56 (d, 2H, *J* = 8.4 Hz), 4.73 – 4.60 (m, 3H), 4.46 – 4.39 (m, 1H), 4.35 (q, 2H, *J* = 7.1 Hz), 4.18 (d, 1H, *J* = 7.2 Hz), 4.13 (dd, 2H, *J* = 7.7, 6.3 Hz), 4.01 (d, 1H, *J* = 16.3 Hz), 3.75 (d, 1H, *J* = 16.3 Hz), 3.61 – 3.53 (m, 1H), 3.26 – 3.16 (m, 1H), 2.89 (t, 2H, *J* = 7.4 Hz), 2.85 – 2.62 (m, 4H), 2.50 (dd, 1H, *J* = 14.0, 6.8 Hz), 2.41 (dd, 1H, *J* = 14.0, 8.1 Hz), 2.16 – 2.06 (m, 1H), 1.97 (s, 3H), 1.93 – 1.79 (m, 2H), 1.75 – 1.55 (m, 3H), 1.55 – 1.37 (m, 3H), 1.30 (t, 3H, *J* = 7.1 Hz), 1.24 – 1.11 (m, 1H), 0.97 (dd, 7H, *J* = 7.3, 6.3 Hz), 0.89 (dd, 3H, *J* = 8.0, 7.4 Hz), 0.83 (d, 3H, *J* = 7.2 Hz); ^{19}F NMR (376 MHz, CD_3OD) δ −77.1 (s, TFA), −101.0 (d, 1F, *J* = 266 Hz), −102.0 (d, 1F, *J* = 266 Hz); HRMS (ESI) m/z: [M + 2H]$^{2+}$ Calcd for $C_{53}H_{76}F_2N_{12}O_{15}$ 579.2761; Found 579.2775 (2.4 ppm error).

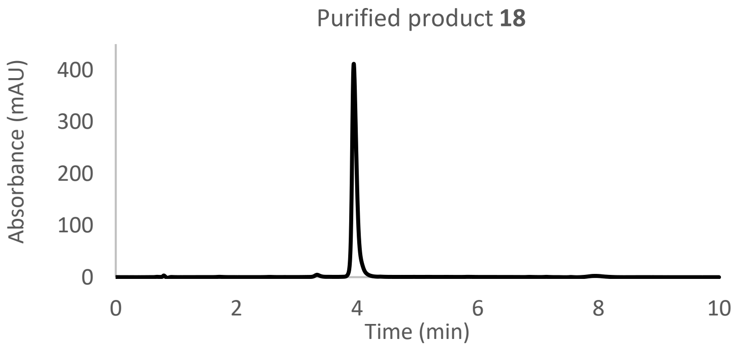

Figure S43. Analytical HPLC chromatogram for purified product **18** Rt = 3.95 min on analytical gradient 20-80% B over 5 min, 0.6 mL min^{-1}, 280 nm.

Ac-WLAHKAL-NH_2 conjugated with 2-Bromo-2,2-difluoro-*N*-(pyridin-3-ylmethyl)acetamide (19)

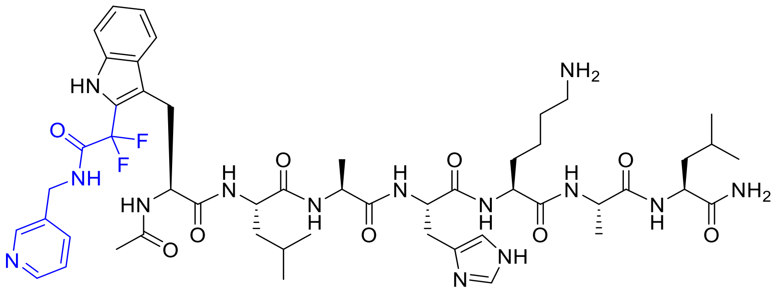

Prepared according to general procedure E using Ac-WLAHKAL-NH_2 **10** (25.0 mM DMSO, 200 µL, 5.00 µmol, 1.00 eq.), 4CzIPN (2.50 mM in DMSO, 20 µL, 0.0500 µmol, 1 mol%) and 2-Bromo-2,2-difluoro-*N*-(pyridin-3-ylmethyl)acetamide **2g** (0.1 M in DMSO, 100 µL, 10.0 µmol, 2.00 eq.). The reaction mixture was irradiated with blue LEDs for 30 min, then was analysed by quantitative ^{19}F NMR spectroscopy (36% yield). The reaction mixture was then purified by semi-preparative RP-HPLC (2-70% B over 30 min) to give the title compound **19** (2.10 mg, 1.98 µmol, 40%) as a colourless powder; ^{1}H NMR (400 MHz, CD_3OD) δ 8.76 (d, 1H, *J* = 1.4 Hz), 8.58 (d, 1H, *J* = 2.2 Hz), 8.54 (dd, 1H, *J* = 5.1, 1.6 Hz), 7.98 (ddd, 1H, *J* = 8.0, 2.2, 1.6 Hz), 7.67 (d, 1H, *J* = 8.0 Hz), 7.57 (dd, 1H, *J* = 8.0, 5.1 Hz), 7.41 (d, 1H, *J* = 8.2 Hz), 7.35 (d, 1H, *J* = 1.4 Hz), 7.24 (ddd, 1H, *J* = 8.2, 7.0, 1.1 Hz), 7.07 (ddd, 1H, *J* = 8.0, 7.0, 1.0 Hz), 4.61 (dd, 2H, *J* = 22.7, 15.1 Hz), 4.58 – 4.44 (m, 2H), 4.40 – 4.32 (m, 1H), 4.32 – 4.28 (m, 1H), 4.28 – 4.22 (m, 1H), 4.22 – 4.10 (m, 2H), 3.42 (dd, 1H, *J* = 14.6, 5.8 Hz), 3.39 – 3.34 (m, 1H), 3.18 (dd, 1H, *J* = 15.4, 8.1 Hz), 2.94 (dd, 2H, *J* = 8.7, 7.2 Hz), 1.94 (s, 3H), 1.91 – 1.79 (m, 1H), 1.78 – 1.53 (m, 9H), 1.52 – 1.43 (m, 2H), 1.39 (d, 3H, *J* = 7.2 Hz), 1.35 (d, 3H, *J* = 7.3 Hz), 1.03 – 0.84 (m, 12H); ^{19}F NMR (376 MHz, CD_3OD) δ −77.2 (s, TFA), −101.1 (d, 1F, *J* = 269 Hz), −101.9 (d, 1F, *J* = 269 Hz); HRMS (ESI) m/z: [M + 2H]$^{2+}$ Calcd for $C_{51}H_{74}F_2N_{14}O_9$532.2866; Found 532.2878 (2.3 ppm error).

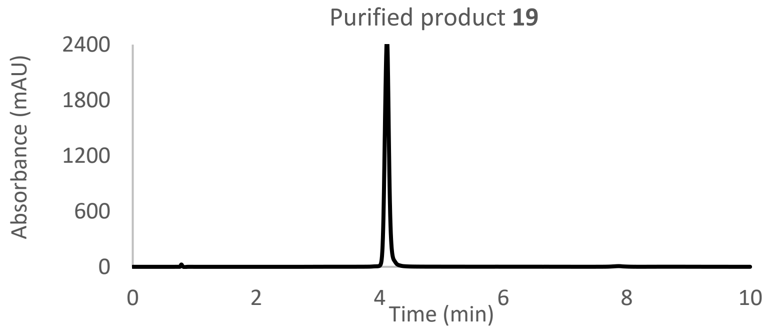

Figure S44. Analytical HPLC chromatogram for purified product **19** Rt = 4.12 min on analytical gradient 2-95% B over 5 min, 0.6 mL min^{-1}, 280 nm.

Ac-VVYPWYQ-NH_2 conjugated with 2,5,8,11,14,17,20,23-Octaoxapentacosan-25-yl 2-bromo-2,2-difluoroacetate (20)

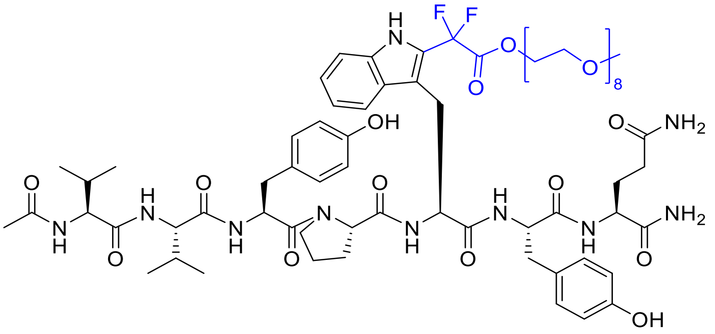

Prepared according to general procedure E using Ac-VVYPWYQ-NH_2 **11** (25.0 mM in DMSO, 200 µL, 5.00 µmol, 1.00 eq.), 4CzIPN (2.50 mM in DMSO, 20 µL, 0.0500 µmol, 1 mol%) and 2,5,8,11,14,17,20,23-octaoxapentacosan-25-yl 2-bromo-2,2-difluoroacetate **2d** (0.1 M in DMSO, 100 µL, 10.0 µmol, 2.00 eq.). The reaction mixture was irradiated with blue LEDs for 20 min, then was analysed by quantitative ^{19}F NMR spectroscopy (46% yield). The reaction mixture was then purified by semi-preparative RP-HPLC (2-70% B over 30 min) to give the title compound **20** (2.9 mg, 1.99 µmol, 40%) as a colourless powder; ^{1}H NMR (500 MHz, DMSO-d_6) δ 11.62 (s, 1H), 9.13 (d, 2H, *J* = 7.3 Hz), 8.08 (d, 1H, *J* = 8.0 Hz), 7.88 (d, 2H, *J* = 8.7 Hz), 7.75 (d, 1H, *J* = 8.1 Hz), 7.63 (d, 1H, *J* = 9.0 Hz), 7.51 (d, 1H, *J* = 7.1 Hz), 7.46 (d, 1H, *J* = 7.9 Hz), 7.37 (d, 1H, *J* = 8.3 Hz), 7.22 – 7.14 (m, 2H), 7.05 – 6.98 (m, 5H), 6.95 (s, 1H), 6.66 – 6.59 (m, 4H), 6.56 – 6.47 (m, 2H), 4.69 – 4.54 (m, 1H), 4.46 – 4.35 (m, 2H), 4.35 – 4.27 (m, 1H), 4.28 – 4.20 (m, 1H), 4.19 – 4.08 (m, 2H), 4.09 – 4.01 (m, 1H), 3.63 (t, 2H, *J* = 4.6 Hz), 3.61 – 3.55 (m, 1H), 3.52 – 3.45 (m, 22H), 3.44 – 3.39 (m, 8H), 3.23 (s, 3H), 3.26 – 3.30 (m, 1H), 3.13 – 3.05 (m, 1H), 2.99 – 2.93 (m, 1H), 2.92 – 2.78 (m, 2H), 2.76 – 2.67 (m, 1H), 2.08 – 2.00 (m, 2H), 1.93 – 1.79 (m, 7H), 1.79 – 1.59 (m, 3H), 1.60 – 1.50 (m, 1H), 0.80 – 0.75 (m, 6H), 0.74 – 0.68 (m, 6H); ^{19}F NMR (376 MHz, DMSO-d_6) δ −73.4 (s, TFA), −98.7 (s, 2F); HRMS (ESI) m/z: $[M + H + Na]^{2+}$ Calcd for $C_{70}H_{101}F_2N_{10}O_{21}Na$ 739.3505; Found 739.3518 (1.8 ppm error).

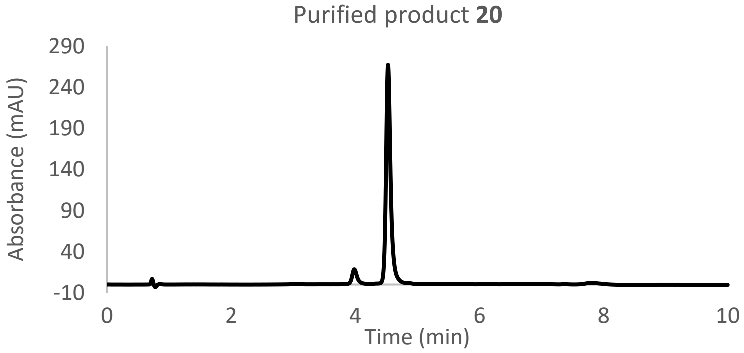

Figure S45. Analytical HPLC chromatogram for purified product **20** Rt = 4.53 min on analytical gradient 20-80% B over 5 min, 0.6 mL min^{-1}, 280 nm.

Ac-DKVGINYW-NH$_2$ conjugated with *N*-(2-(2-bromo-2,2-difluoroacetamido)ethyl)-5-((3a*S*,4*S*,6a*R*)-2-oxohexahydro-1*H*-thieno[3,4-d]imidazol-4-yl)pentanamide (21)

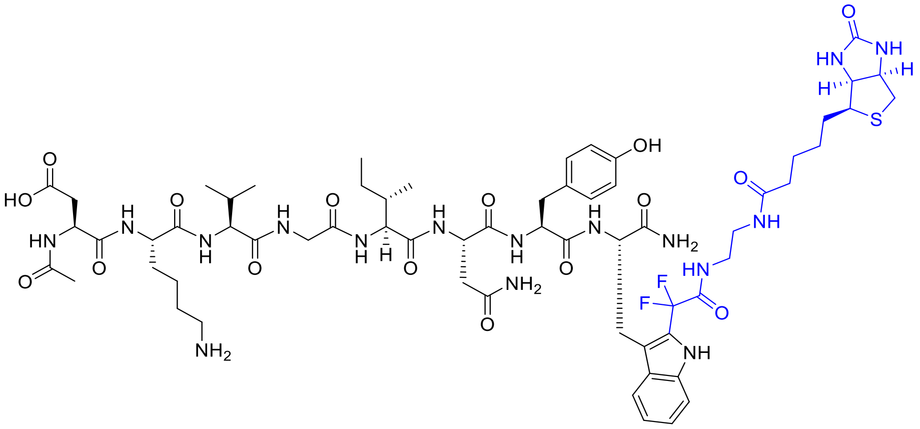

Prepared according to general procedure E using Ac-DKVGINYW-NH$_2$ **12** (25.0 mM in DMSO, 200 µL, 5.00 µmol, 1.00 eq.), 4CzIPN (2.50 mM in DMSO, 20 µL, 0.0500 µmol, 1 mol%) and *N*-(2-(2-bromo-2,2-difluoroacetamido)ethyl)-5-((3a*S*,4*S*,6a*R*)-2-oxohexahydro-1*H*-thieno[3,4-d]imidazol-4-yl)pentanamide **2k** (0.1 M in DMSO, 100 µL, 10.0 µmol, 2.00 eq.). The reaction mixture was irradiated with blue LEDs for 150 min, then was analysed by quantitative ^{19}F NMR spectroscopy (27% yield). The reaction mixture was then purified by semi-preparative RP-HPLC (2-70% B over 30 min) to give the title compound **21** (1.6 mg, 1.14 µmol, 22%) as a colourless powder; ^{1}H NMR (400 MHz, CD$_3$OD) δ 7.84 (d, 1H, *J* = 8.0 Hz), 7.44 (d, 1H, *J* = 8.3 Hz), 7.26 (dd, 1H, *J* = 8.3, 7.5 Hz), 7.16 (dd, 1H, *J* = 8.0, 7.5 Hz), 6.68 (d, 2H, *J* = 8.5 Hz), 6.54 (d, 2H, *J* = 8.5 Hz), 4.75 – 4.70 (m, 1H), 4.69 – 4.60 (m, 2H), 4.53 – 4.38 (m, 2H), 4.33 – 4.22 (m, 2H), 4.19 (d, 1H, *J* = 7.5 Hz), 4.14 (d, 1H, *J* = 7.2 Hz), 4.01 (d, 1H, *J* = 16.4 Hz), 3.78 (d, 1H, *J* = 16.4 Hz), 3.60 – 3.40 (m, 3H), 3.25 – 3.16 (m, 1H), 3.11 – 3.02 (m, 1H), 2.96 – 2.76 (m, 6H), 2.75 – 2.63 (m, 4H), 2.58 – 2.45 (m, 2H), 2.39 – 2.31 (m, 1H), 2.17 – 2.08 (m, 1H), 2.08 – 2.02 (m, 2H), 1.98 (s, 3H), 1.91 – 1.80 (m, 2H), 1.78 – 1.56 (m, 2H), 1.56 – 1.34 (m, 6H), 1.32 – 1.10 (m, 3H), 0.97 (dd, 7H, *J* = 7.2, 6.3 Hz), 0.88 (dd, *J* = 7.6, 7.4 Hz, 3H), 0.84 (d, 3H, *J* = 7.5 Hz); ^{19}F NMR (376 MHz, CD$_3$OD) δ −77.0 (s, TFA), −99.2 (d, 1F, *J* = 269 Hz), −102.0 (d, 1F, *J* = 269 Hz); HRMS (ESI) m/z: [M + H + Na]$^{2+}$ Calcd for $C_{63}H_{91}F_2N_{16}O_{16}SNa$ 710.3193; Found 710.3207 (2.0 ppm error).

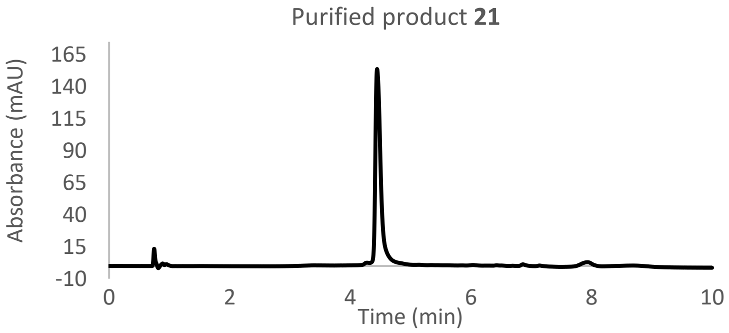

Figure S46. Analytical HPLC chromatogram for purified product **21** Rt = 4.45 min on analytical gradient 2-95% B over 5 min, 0.6 mL min^{-1}, 280 nm.

7. References

1 Karlsson, I.; Persson, E.; Ekebergh, A.; Mårtensson, J. and Börje, A.; *Chem. Res. Toxicol.*, **2014**, 27, 1294–1303.

2 Yang, J.; Sun, B.; Ding, H.; Huang, P.-Y.; Tang, X.-L.; Shi, R.-C.; Yan, Z.-Y.; Yu, C.-M. and Jin, C.; *Green Chem.*, **2021**, 23, 575–581.

3 Morimoto, H.; Fujiwara, R.; Shimizu, Y.; Morisaki, K. and Ohshima, T.; *Org. Lett.*, **2014**, 16, 2018–2021.

4 Uno, M.; Sumino, S.; Fukuyama, T.; Matsuura, M.; Kuroki, Y.; Kishikawa, Y. and Ryu, I.; *J. Org. Chem.*, **2019**, 84, 9330–9338.

5 Yu, C.; Ke, F.; Su, J.; Ma, X.; Li, X.and Song, Q.; *Org. Lett.*, **2022**, 24, 7861–7865.

6 Lv, Y.; Pu, W.; Chen, Q.; Wang, Q.; Niu, J. and Zhang, Q.; *J. Org. Chem.*, **2017**, 82, 8282–8289.

7 Griffiths, R. C.; Smith, F. R.; Long, J. E.; Williams, H. E. L.; Layfield, R. and Mitchell, N. J.; *Angew. Chem. Int. Ed.,* **2020**, 59, 23659-23667.

8. NMR spectra

¹H NMR spectrum of compound **1** (400 MHz, $CDCl_3$)

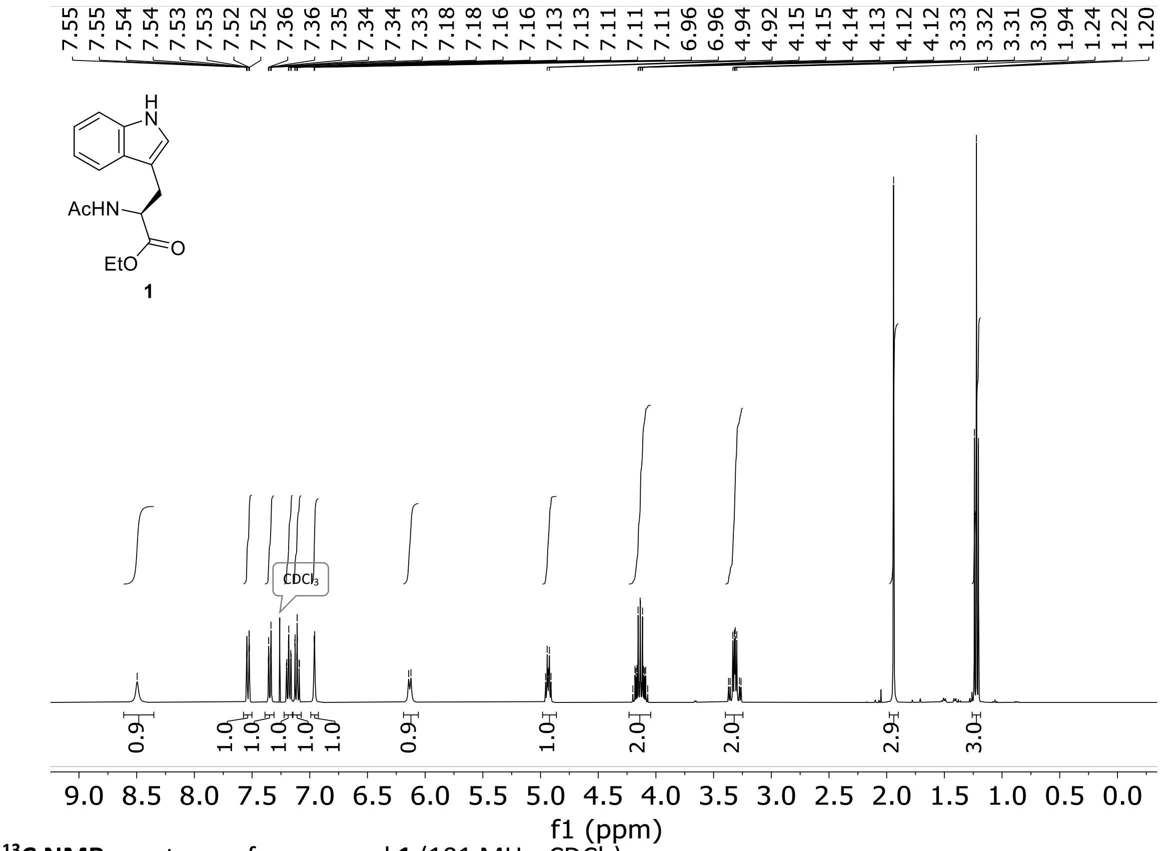

¹³C NMR spectrum of compound **1** (101 MHz, $CDCl_3$)

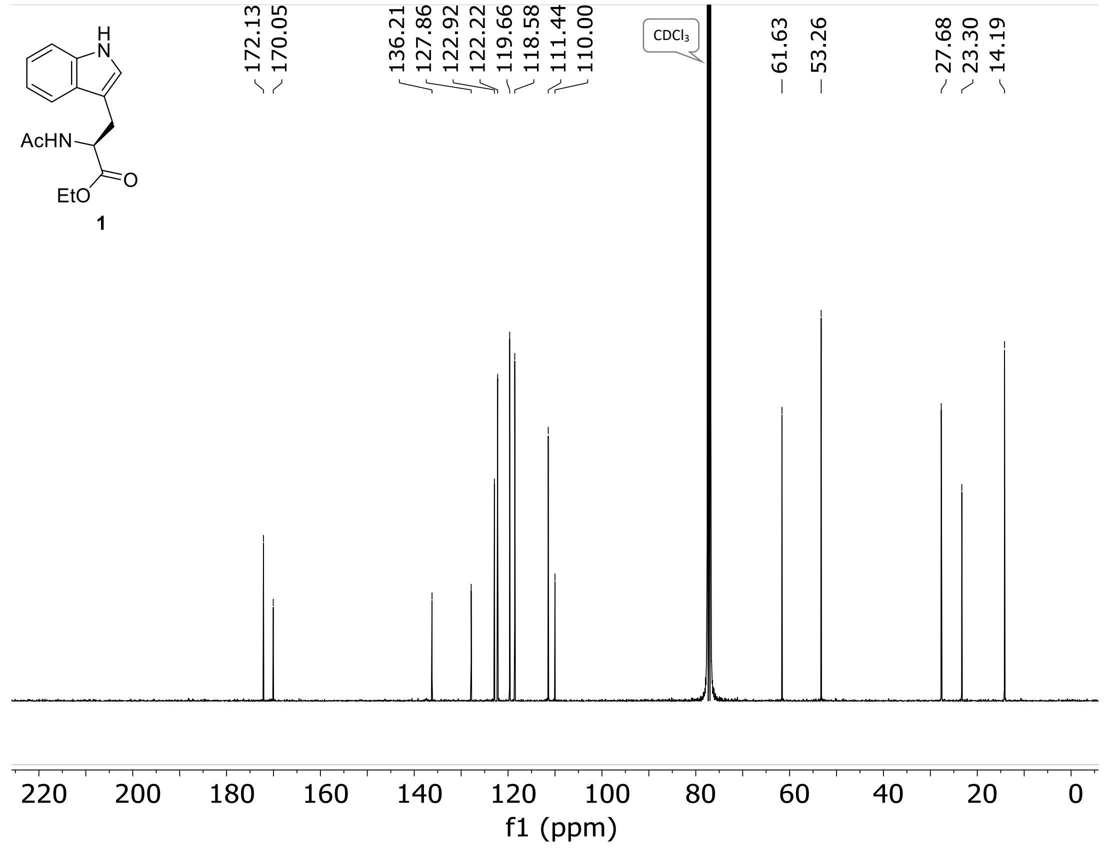

Radical precursors

^{1}H NMR spectrum of compound **2b** (400 MHz, $CDCl_3$)

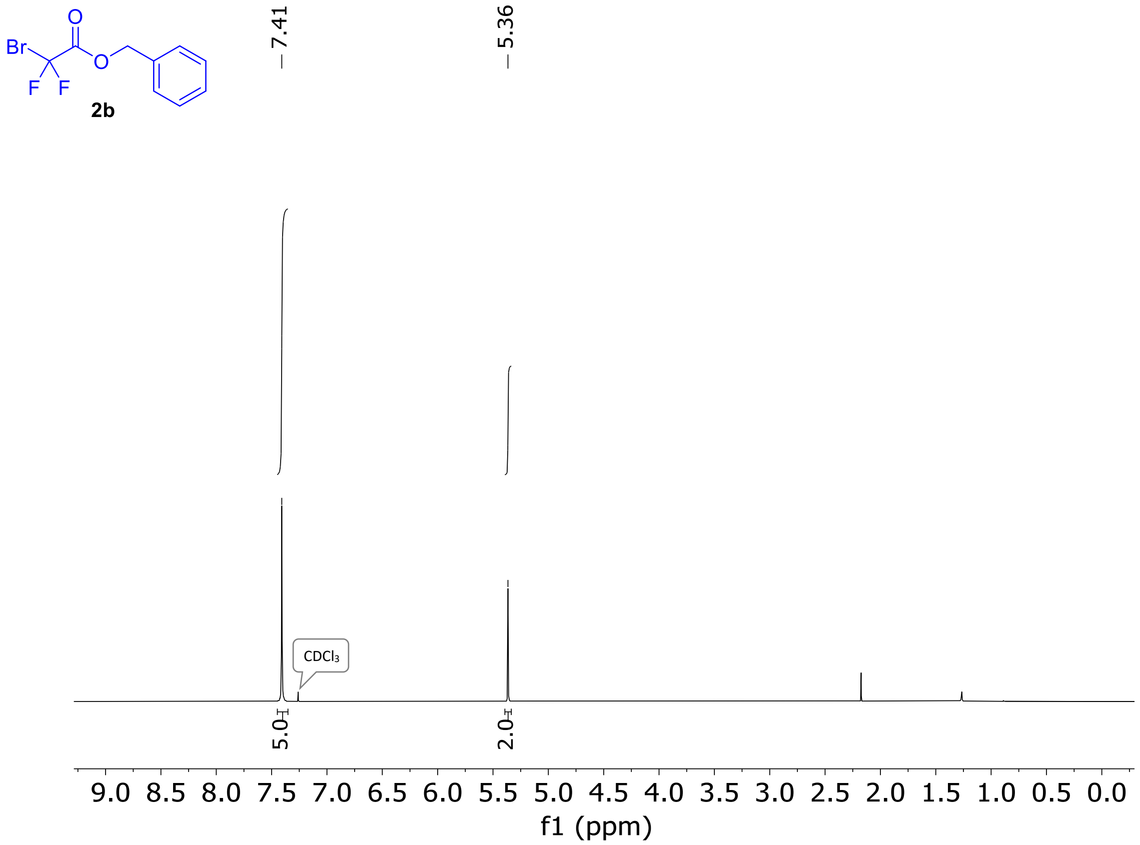

^{13}C NMR spectrum of compound **2b** (101 MHz, $CDCl_3$)

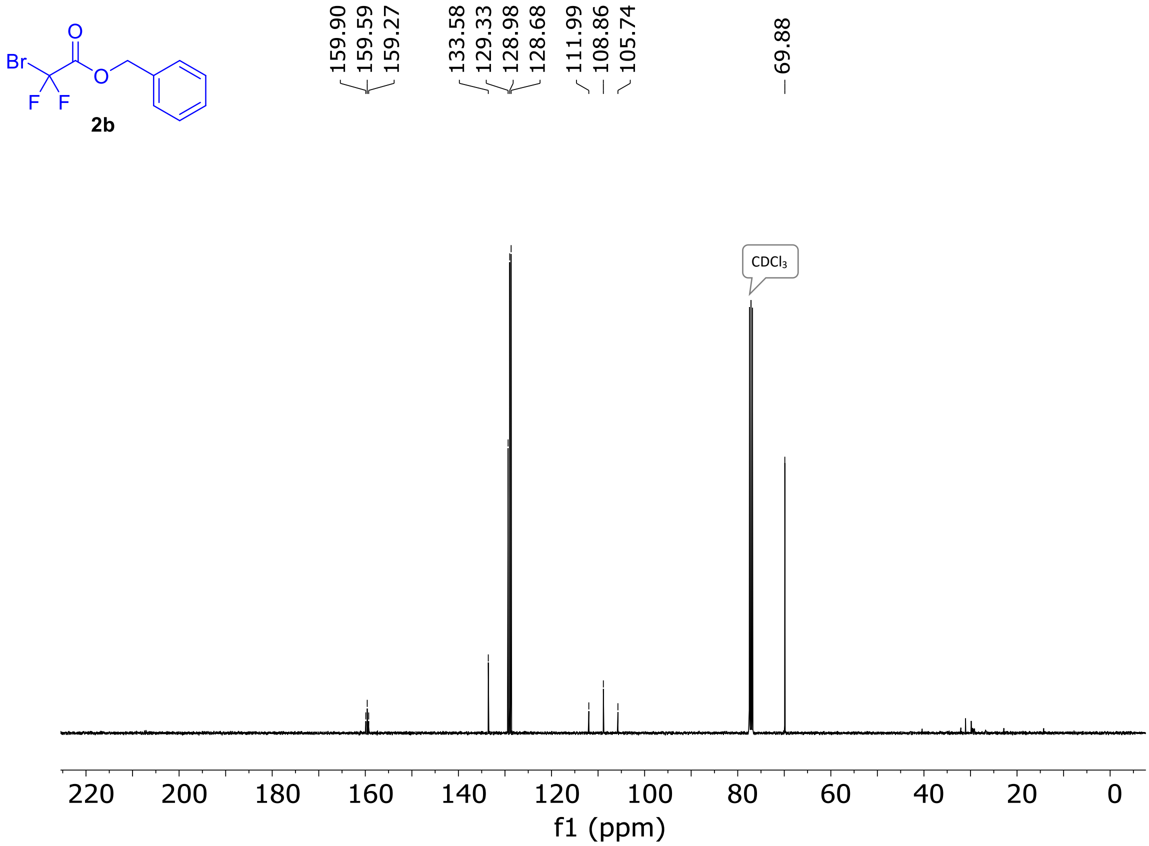

^{19}**F NMR** spectrum of compound **2b** (376 MHz, $CDCl_3$)

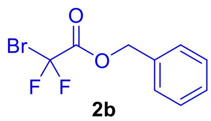

2b

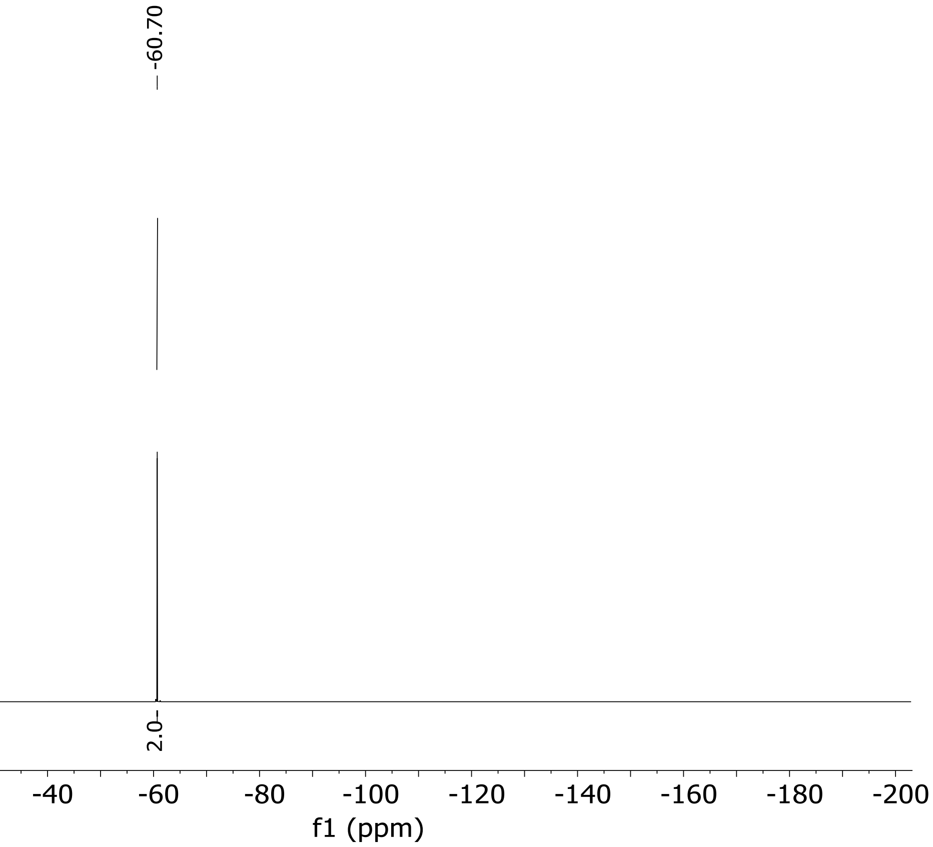

^{1}H NMR spectrum of compound **2c** (400 MHz, $CDCl_3$)

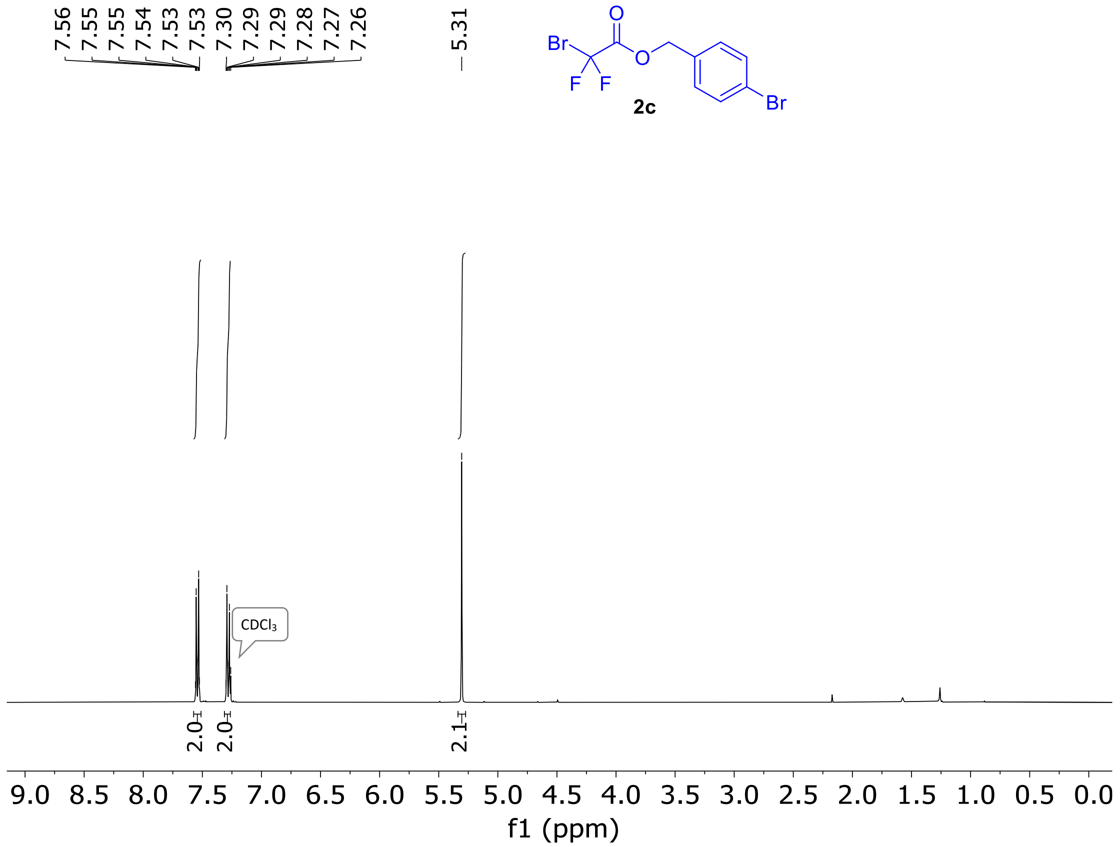

^{13}C NMR spectrum of compound **2c** (101 MHz, $CDCl_3$)

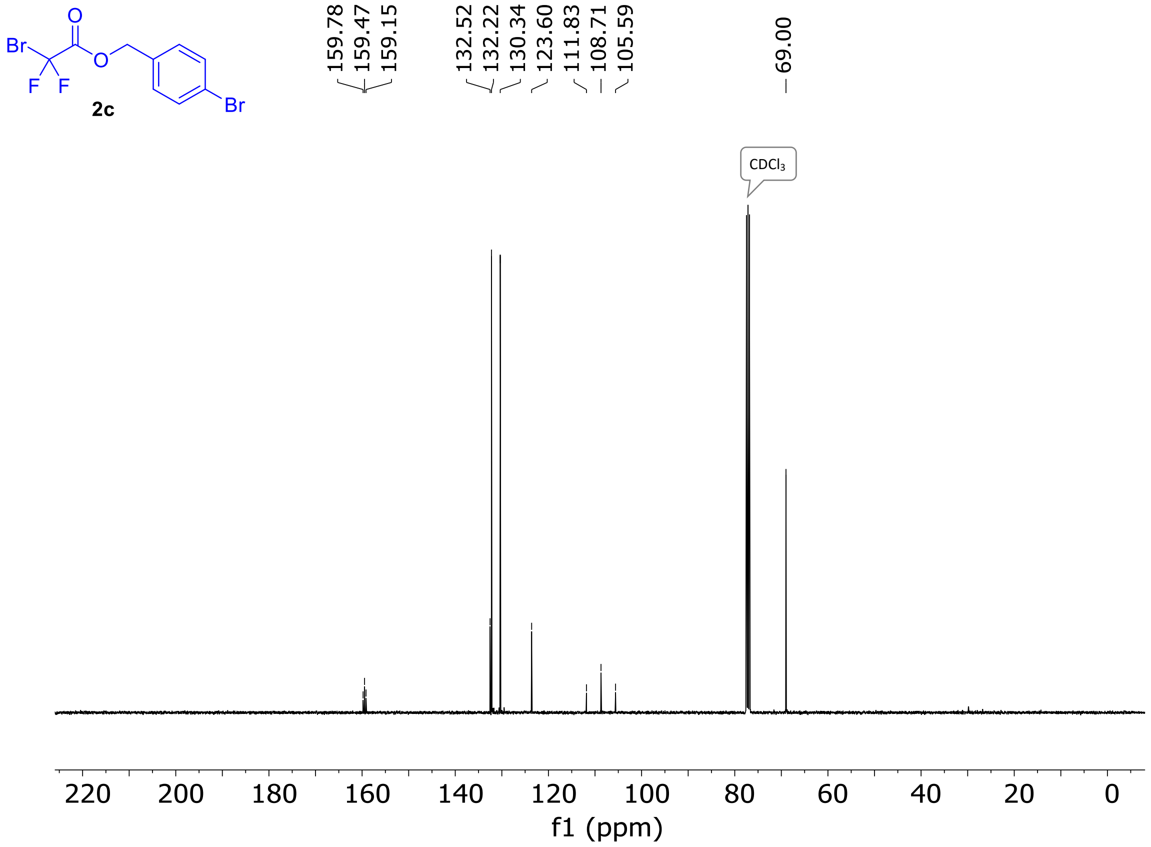

^{19}F NMR spectrum of compound **2c** (376 MHz, $CDCl_3$)

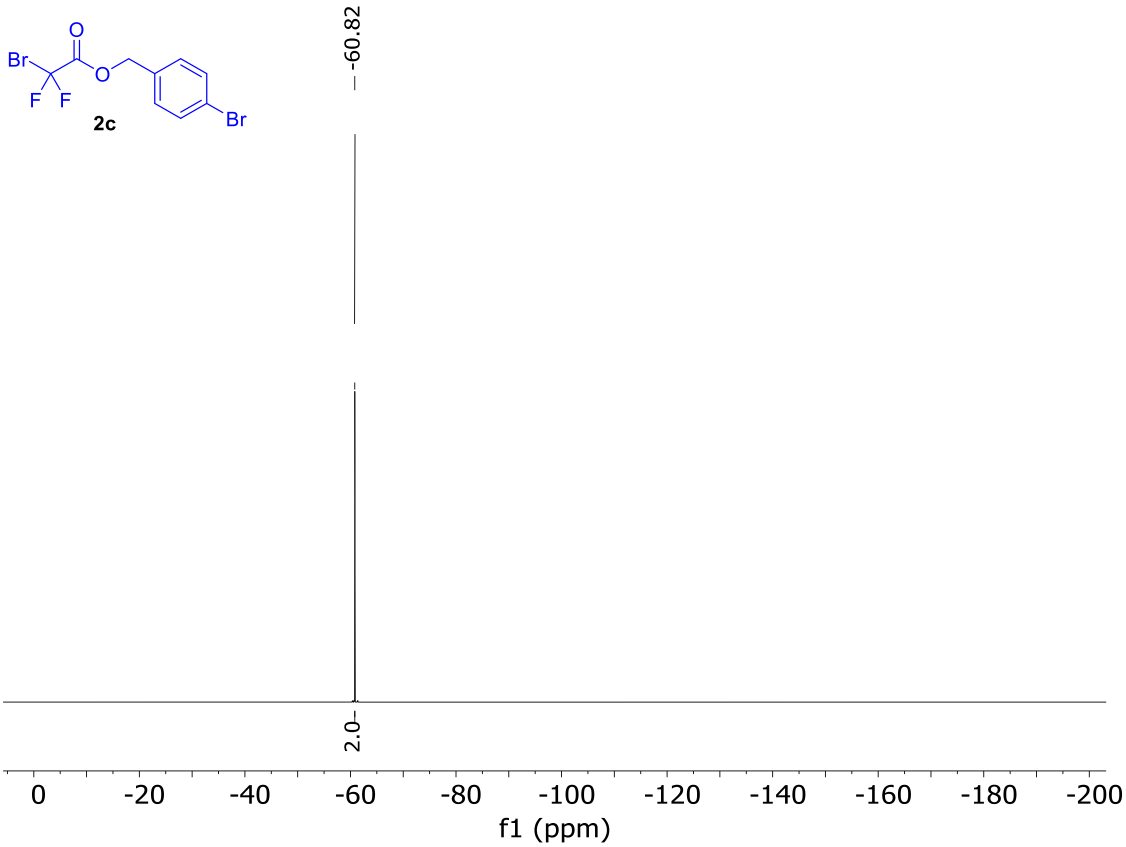

^{1}H NMR spectrum of compound **2d** (400 MHz, $CDCl_3$)

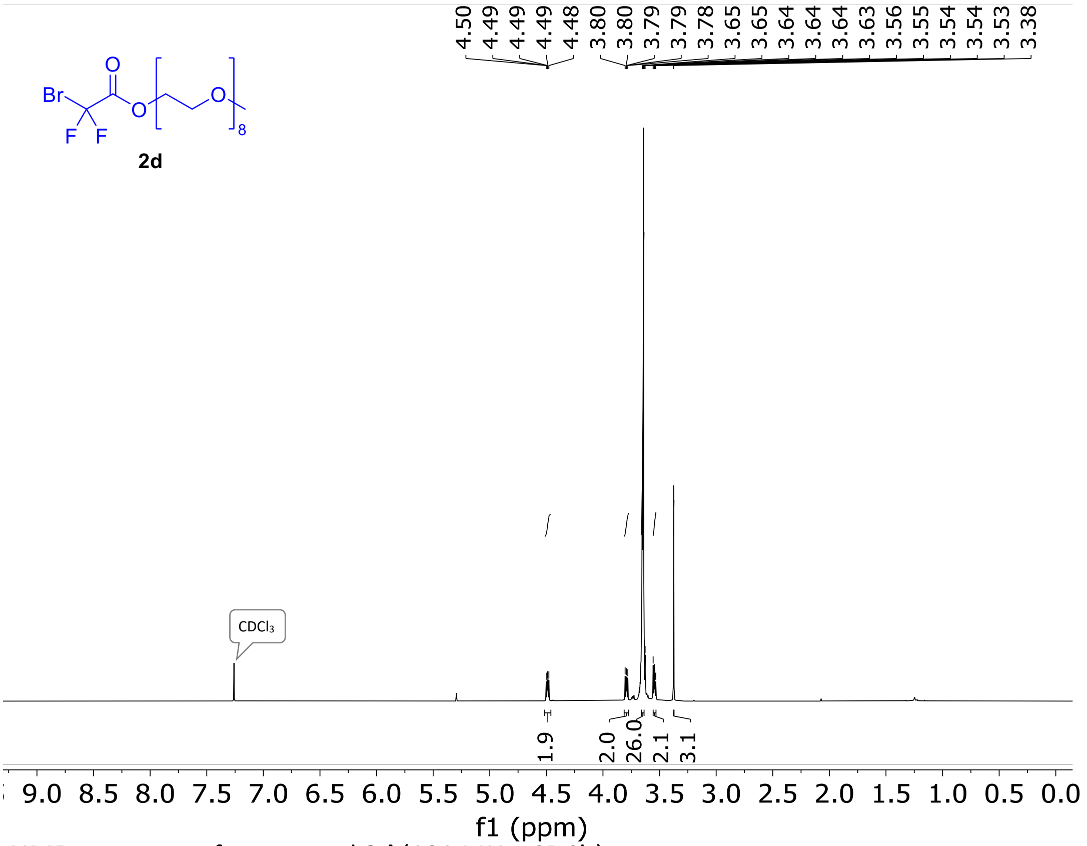

^{13}C NMR spectrum of compound **2d** (101 MHz, $CDCl_3$)

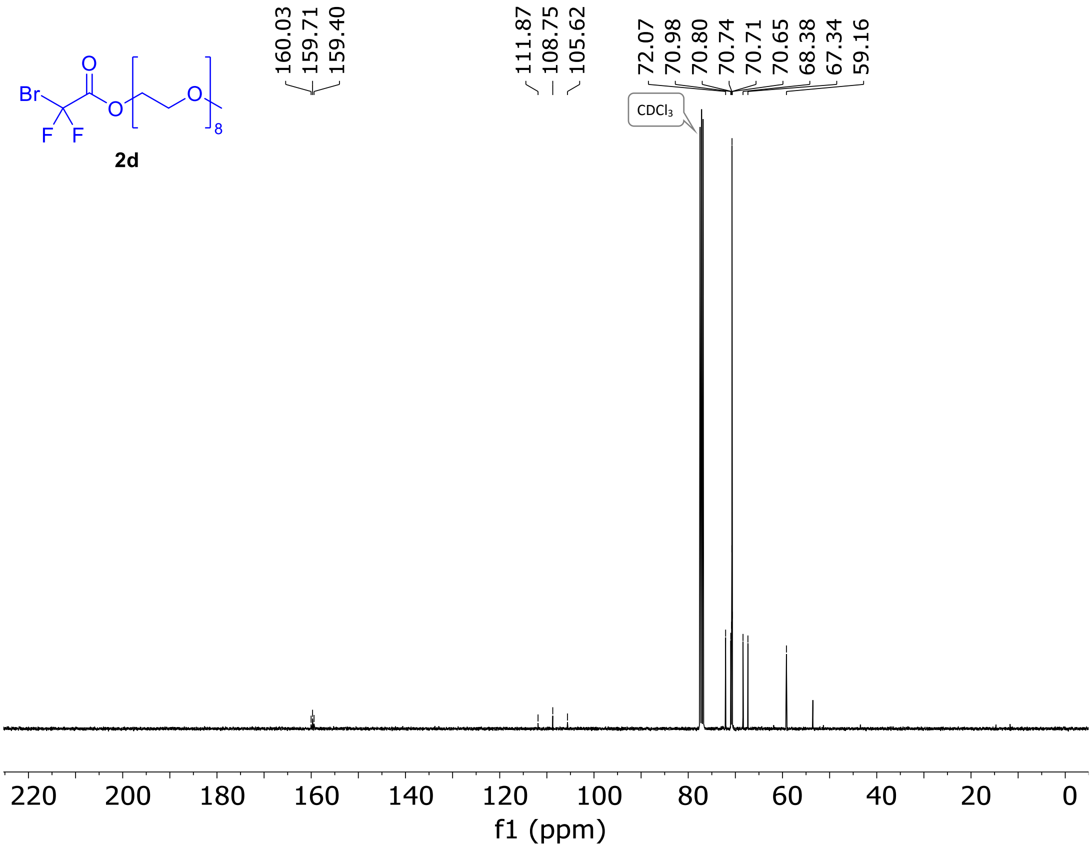

^{19}F NMR spectrum of compound **2d** (376 MHz, $CDCl_3$)

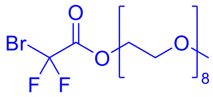

2d

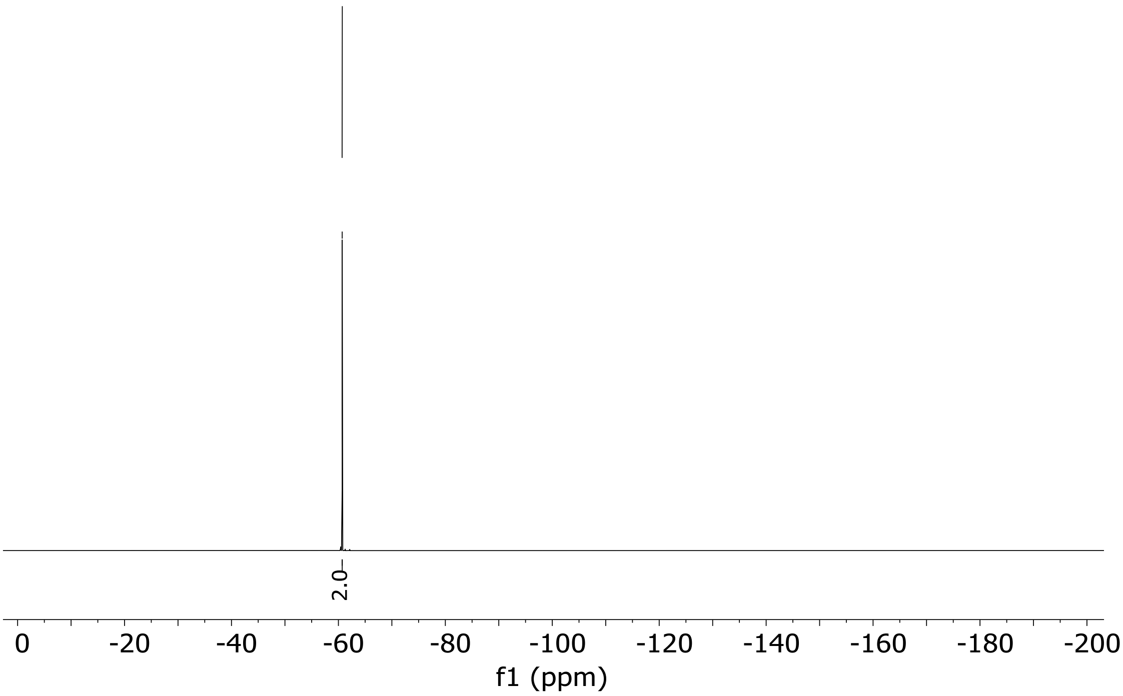

^{1}**H NMR** spectrum of compound **2e** (400 MHz, $CDCl_3$)

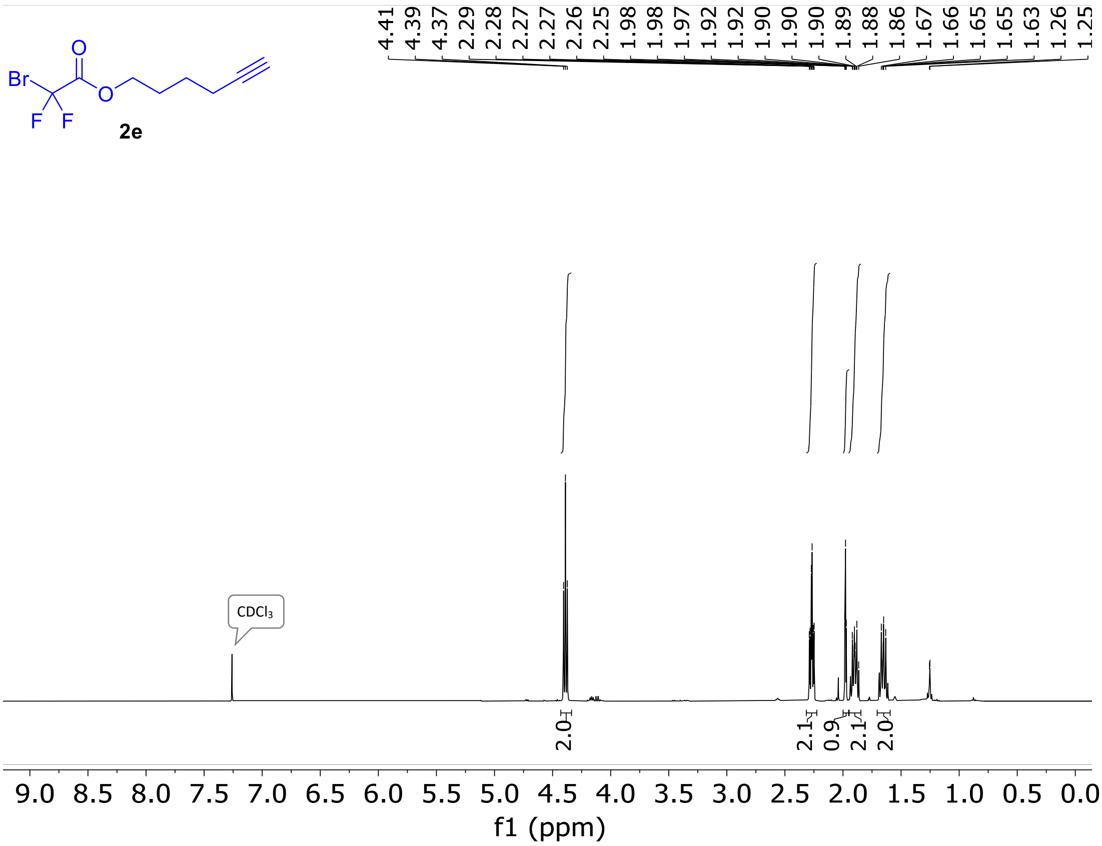

^{13}**C NMR** spectrum of compound **2e** (101 MHz, $CDCl_3$)

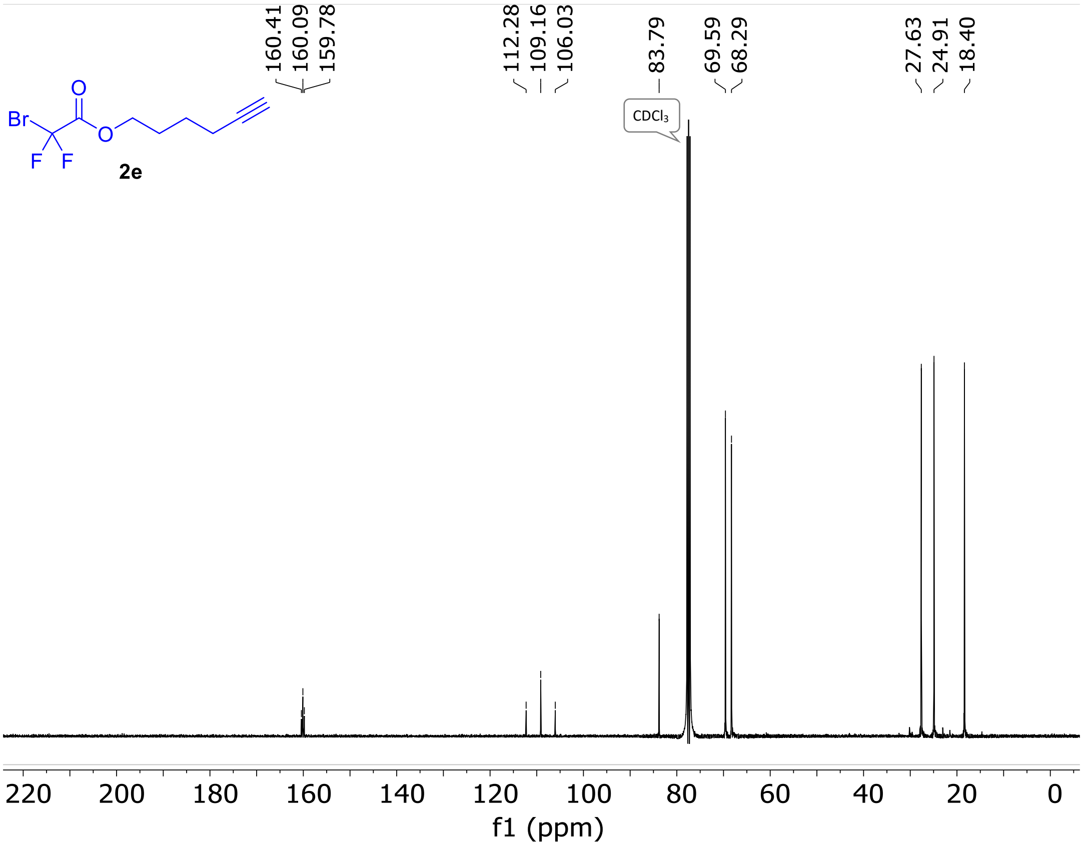

^{19}**F NMR** spectrum of compound **2e** (376 MHz, $CDCl_3$)

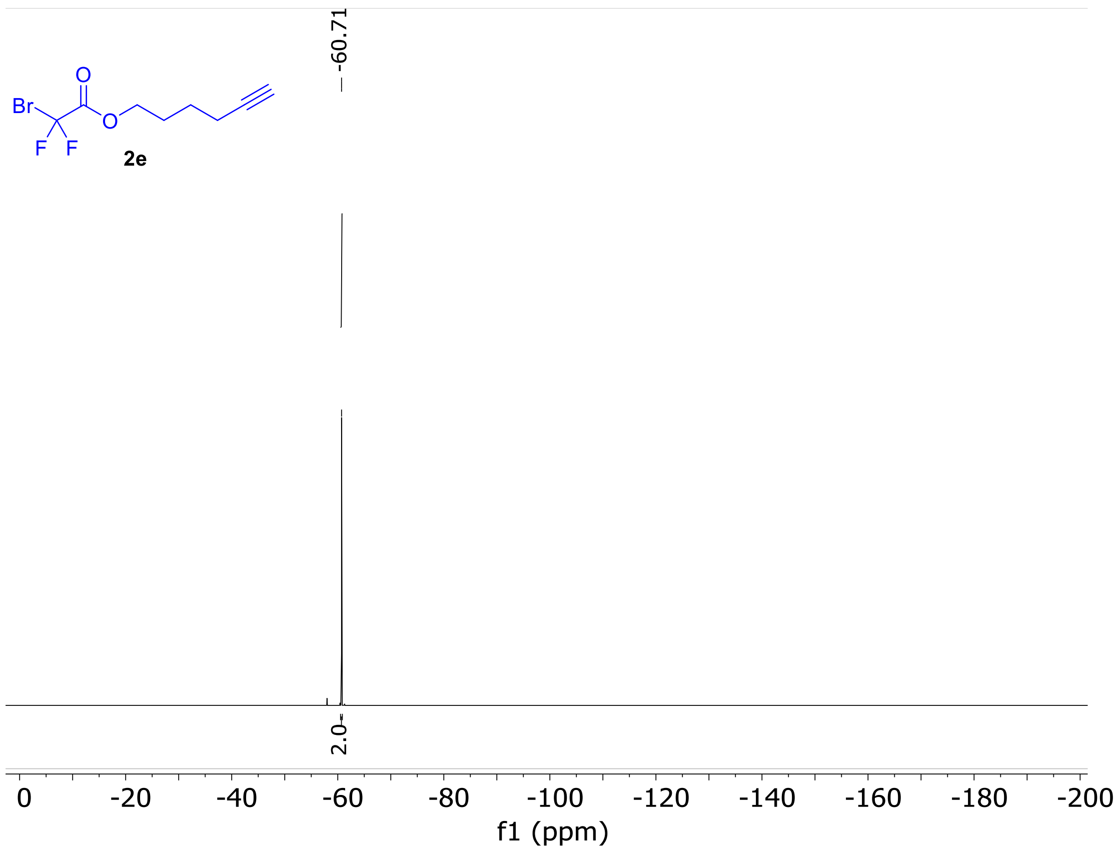

^{1}H NMR spectrum of compound **2f** (400 MHz, $CDCl_3$)

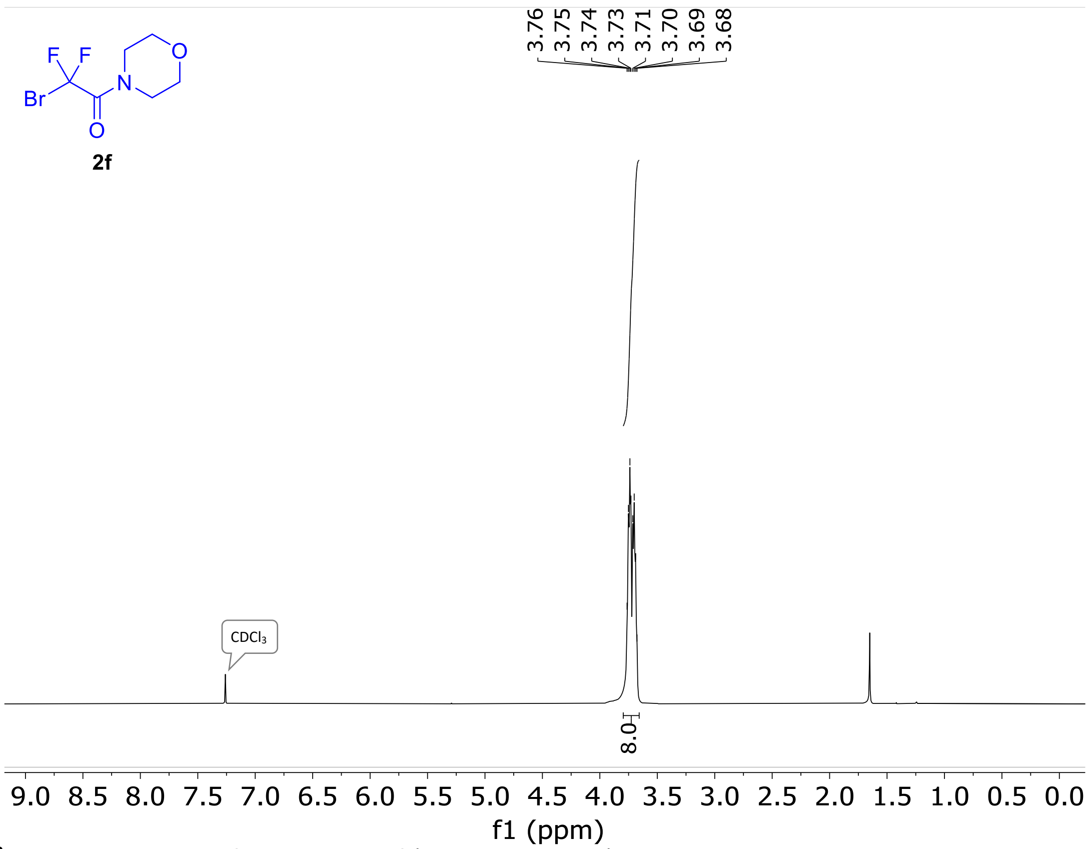

^{13}C NMR spectrum of compound **2f** (101 MHz, $CDCl_3$)

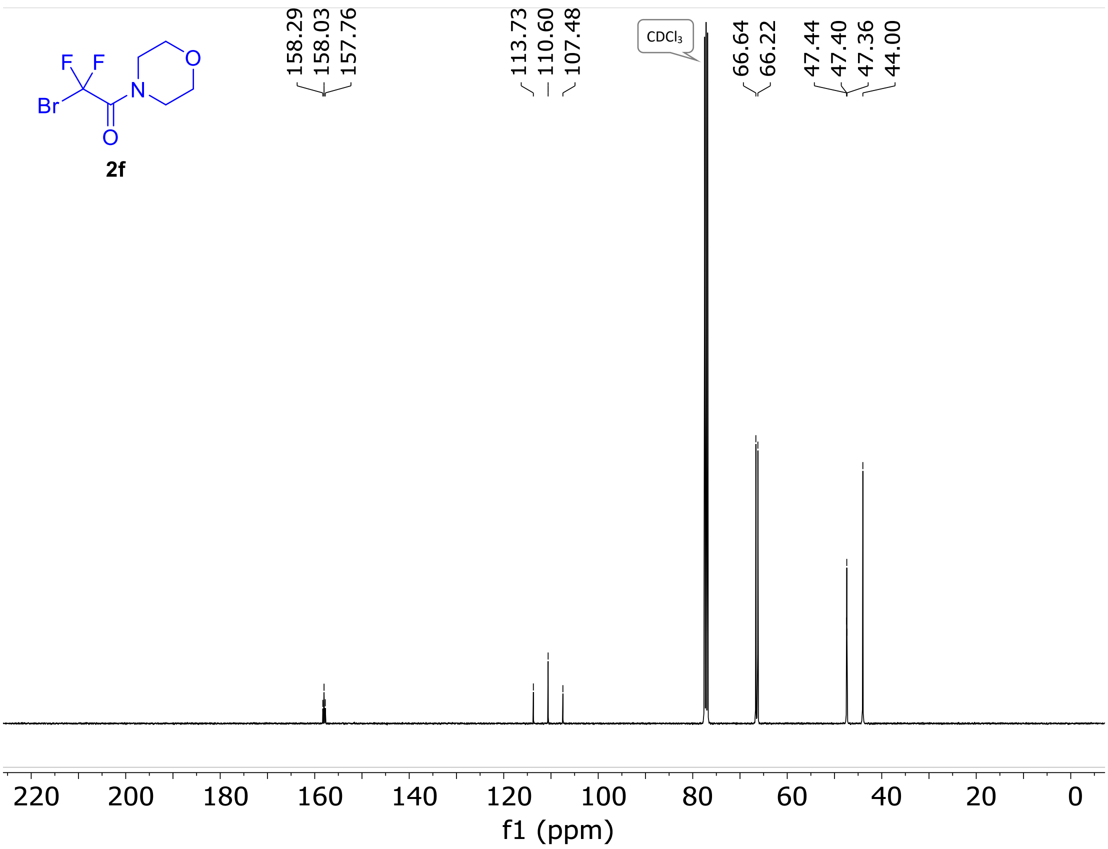

^{19}**F NMR** spectrum of compound **2f** (376 MHz, $CDCl_3$)

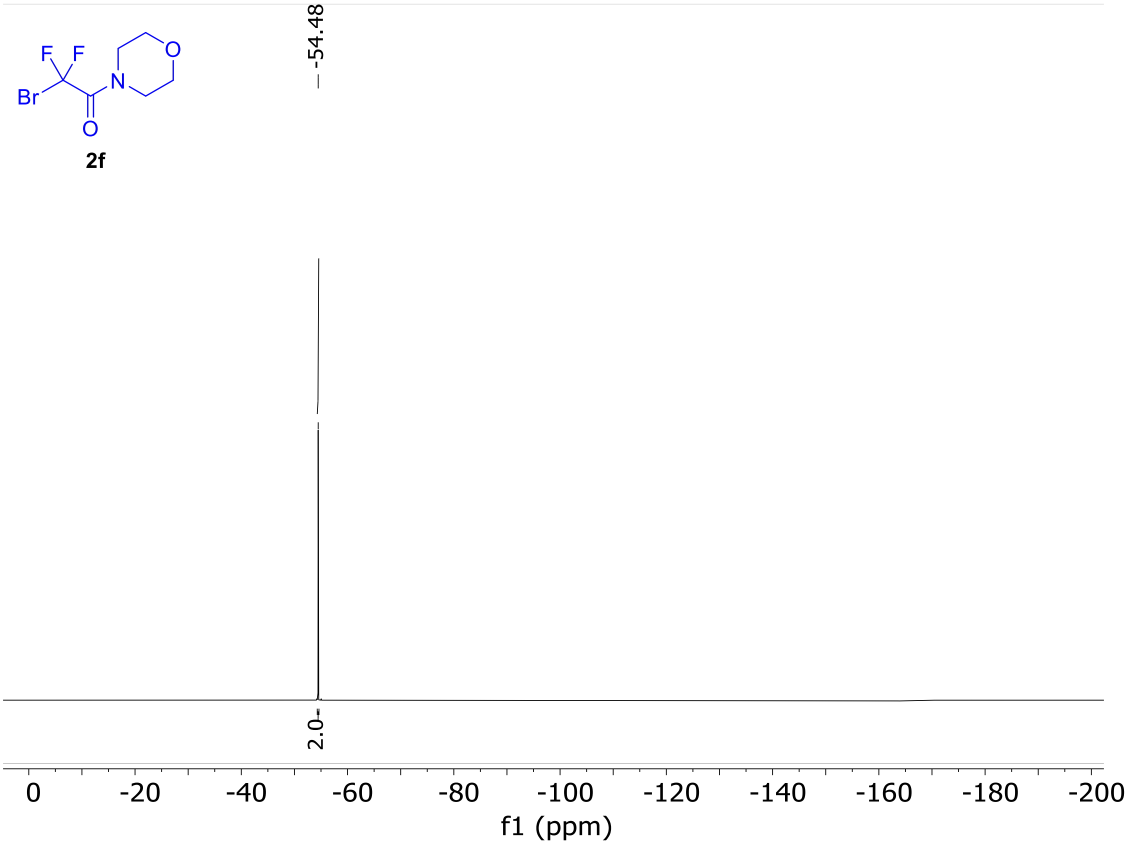

^{1}H NMR spectrum of compound **2g** (400 MHz, $CDCl_3$)

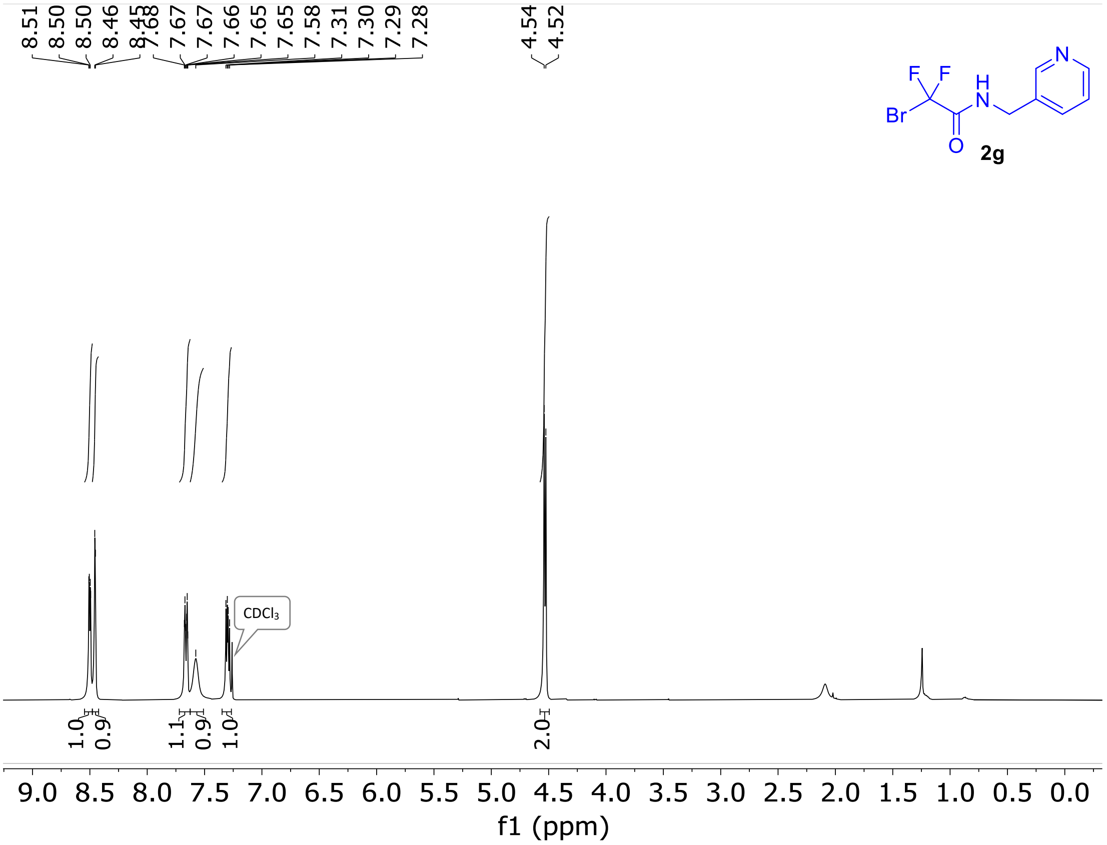

^{13}C NMR spectrum of compound **2g** (101 MHz, $CDCl_3$)

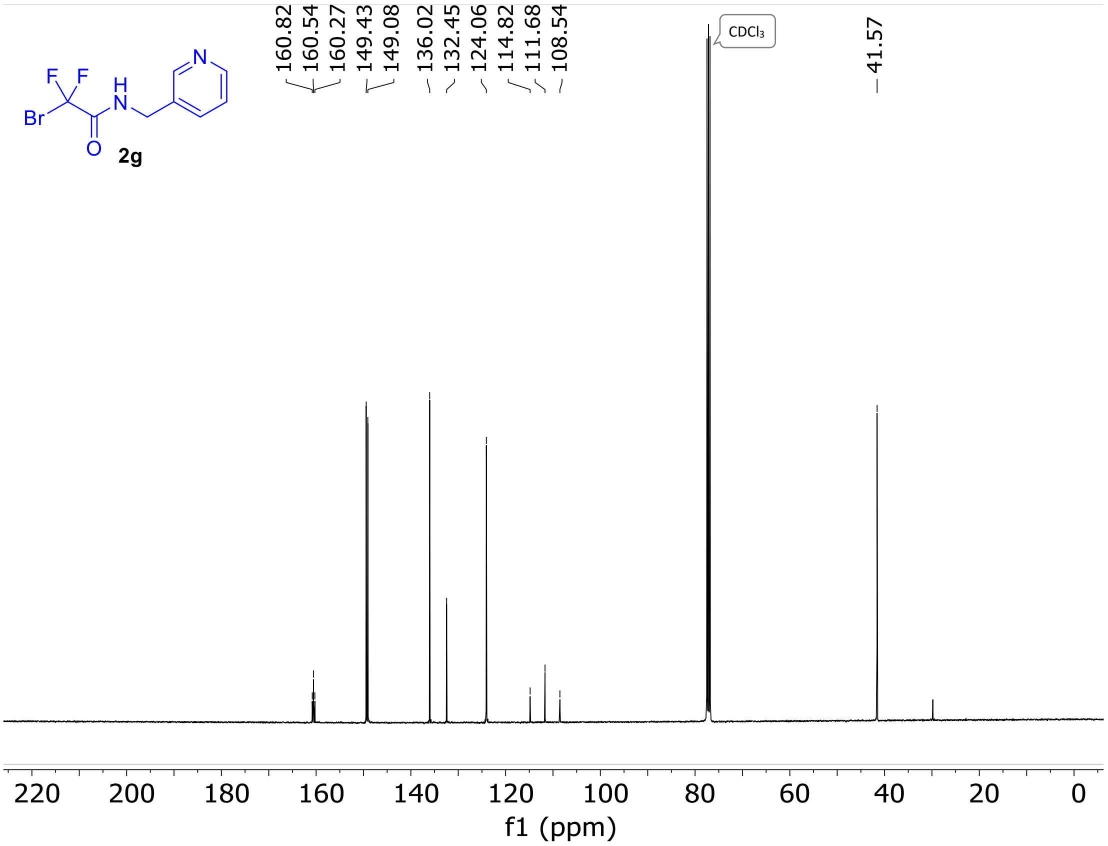

^{19}F NMR spectrum of compound **2g** (376 MHz, $CDCl_3$)

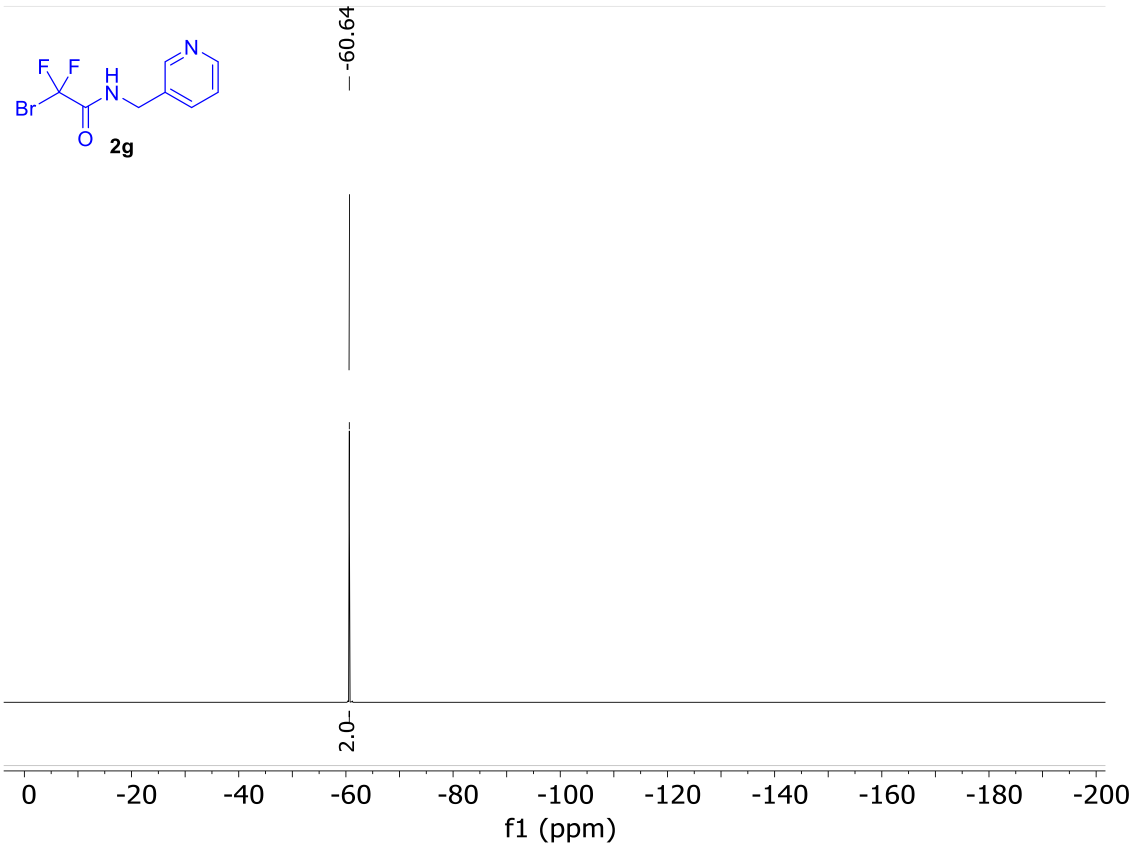

^{1}H NMR spectrum of compound **2h** (400 MHz, $CDCl_3$)

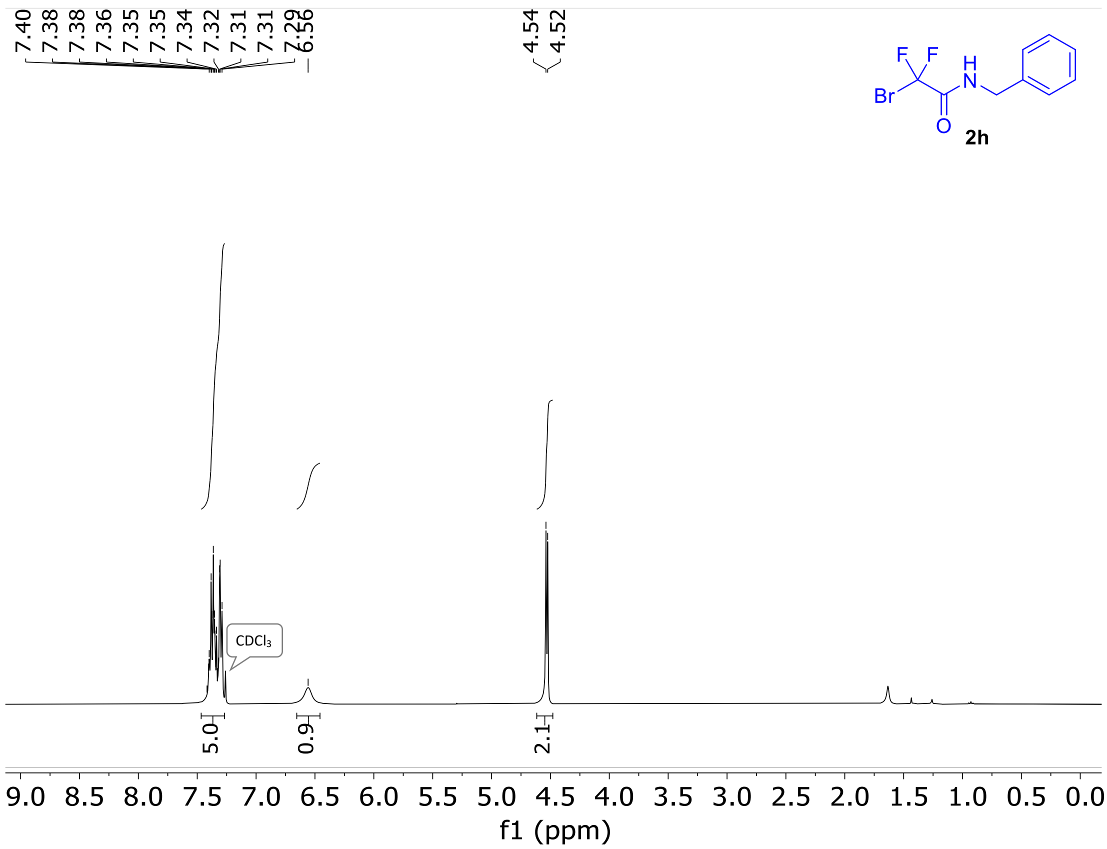

^{13}C NMR spectrum of compound **2h** (101 MHz, $CDCl_3$)

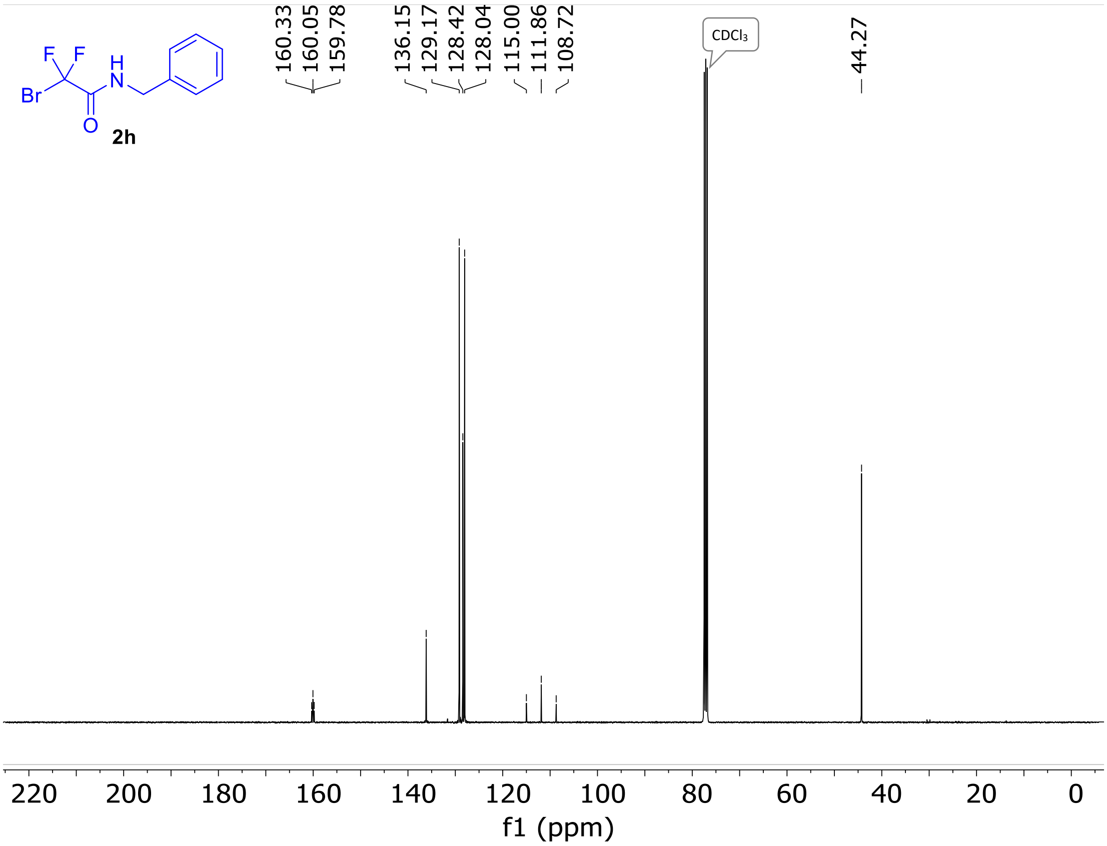

^{19}F NMR spectrum of compound **2h** (376 MHz, $CDCl_3$)

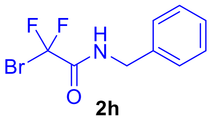

2h

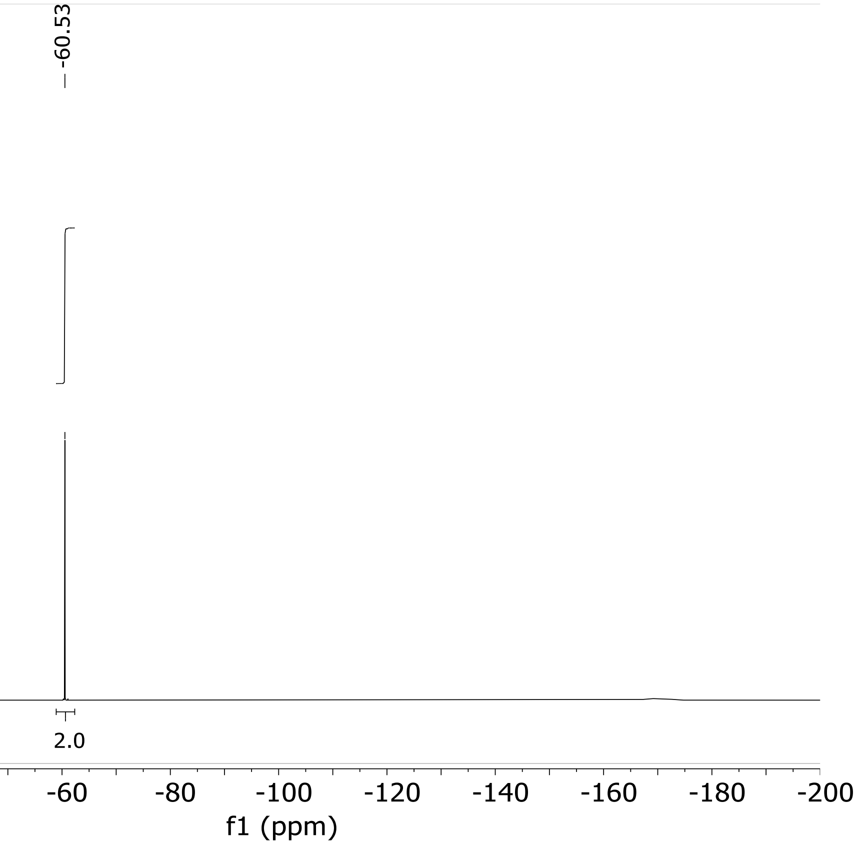

^{1}H NMR spectrum of compound **2i** (400 MHz, $CDCl_3$)

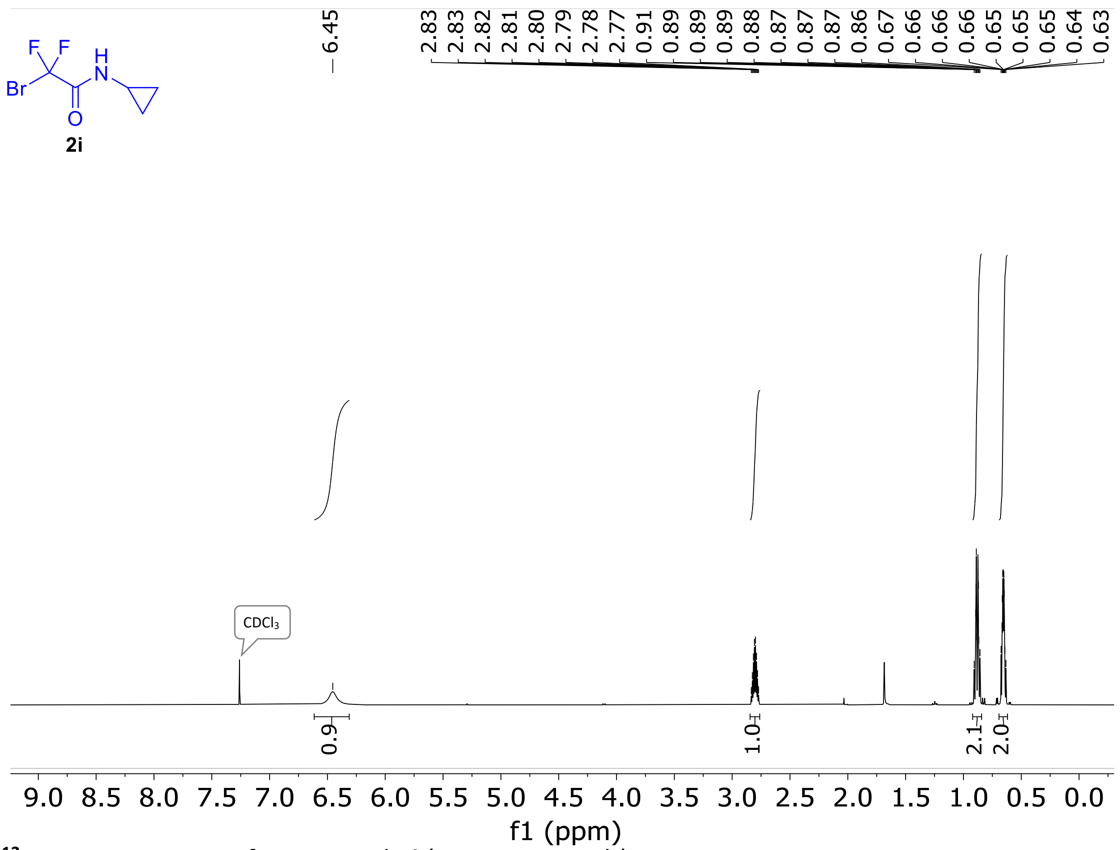

^{13}C NMR spectrum of compound **2i** (101 MHz, $CDCl_3$)

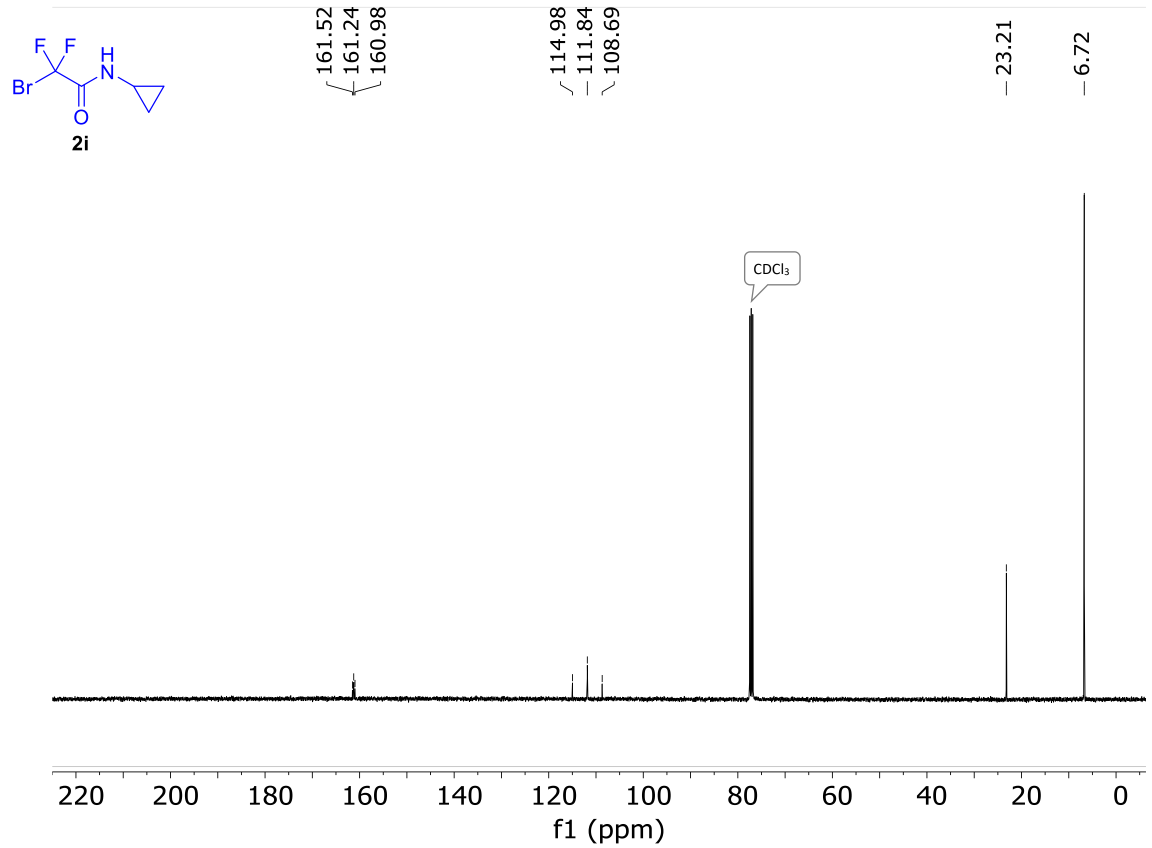

^{19}**F NMR** spectrum of compound **2i** (376 MHz, $CDCl_3$)

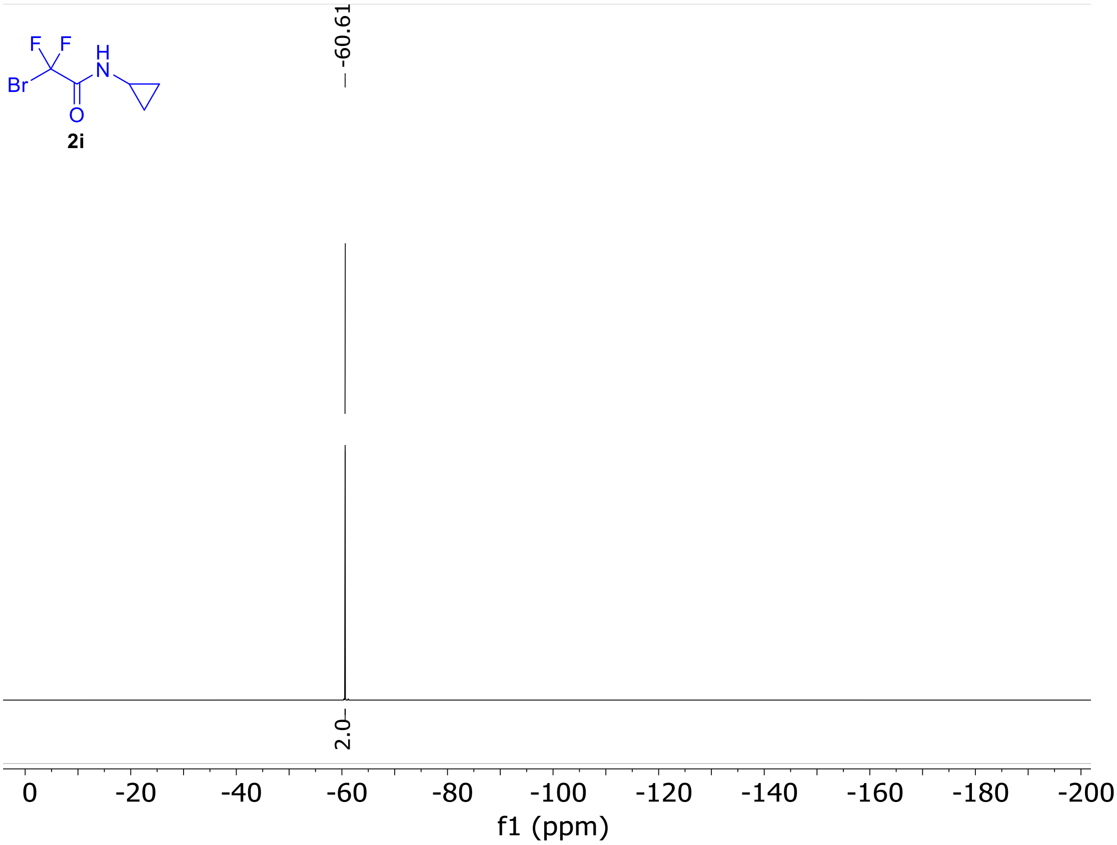

^{1}**H NMR** spectrum of compound **2j** (400 MHz, $CDCl_3$)

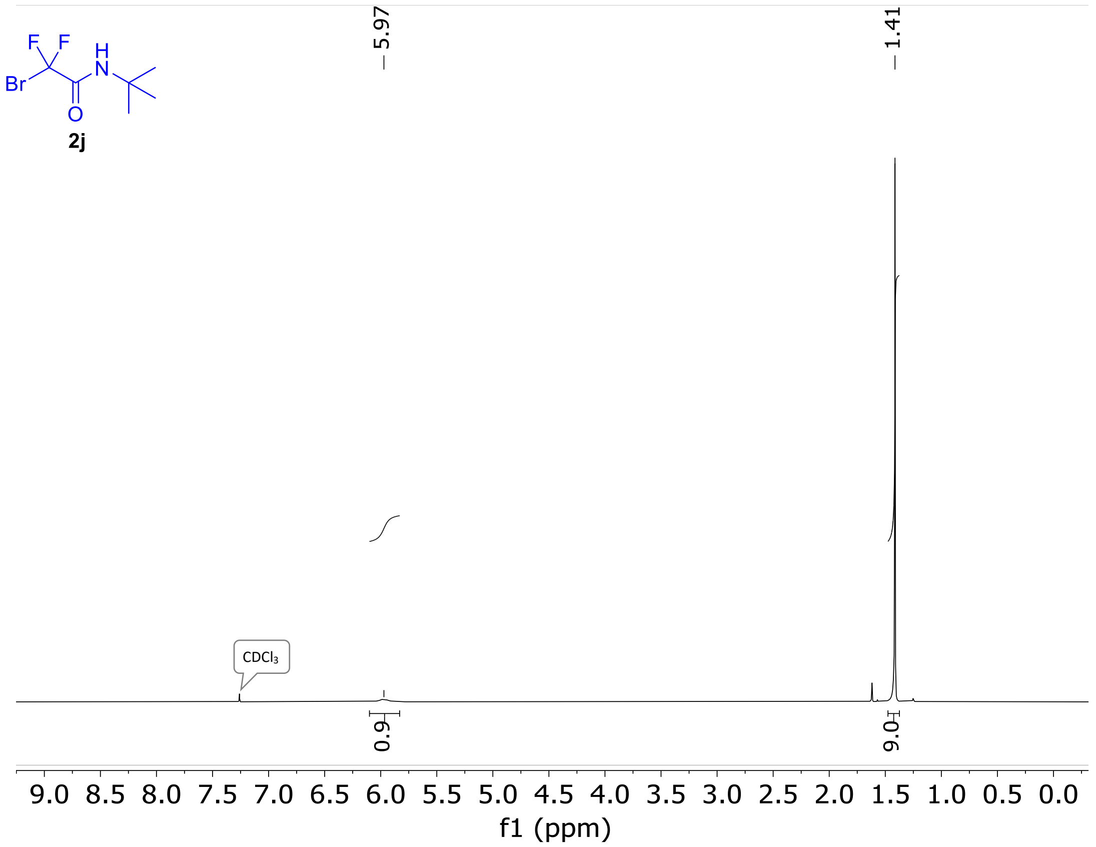

^{13}**C NMR** spectrum of compound **2j** (101 MHz, $CDCl_3$)

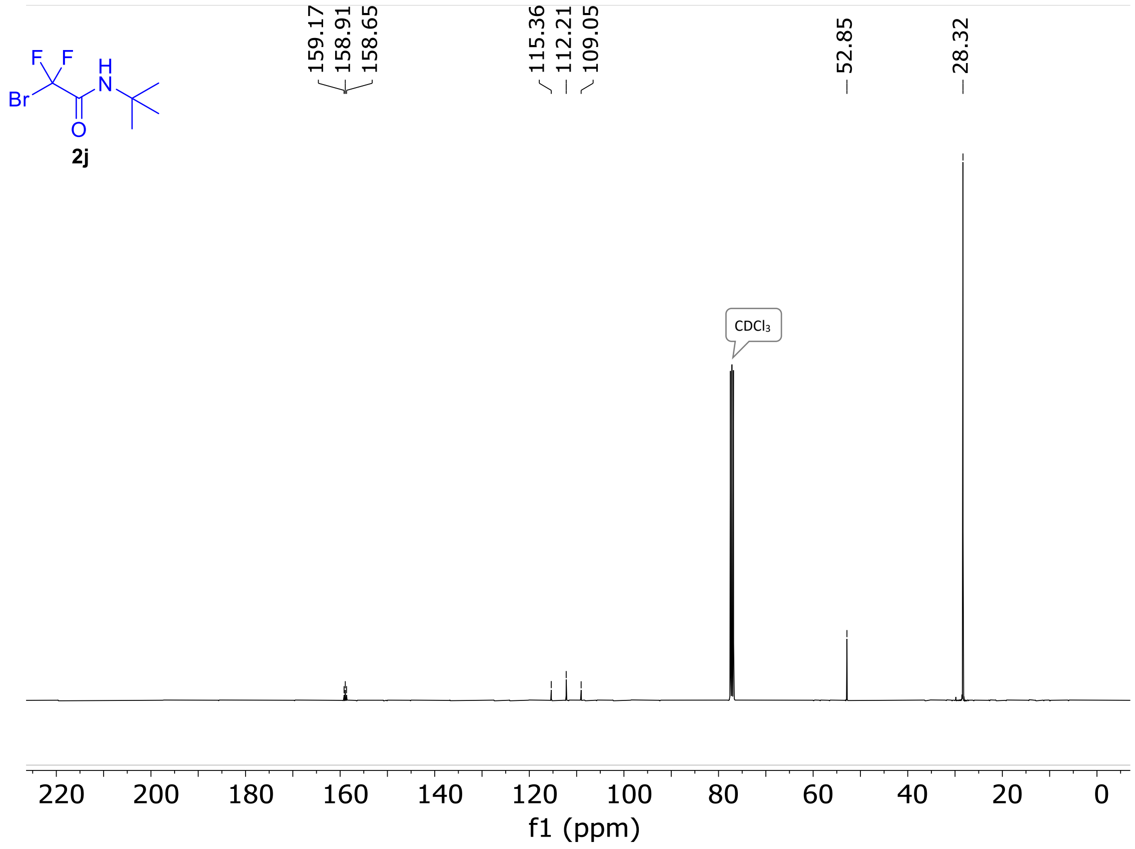

^{19}F NMR spectrum of compound **2j** (376 MHz, $CDCl_3$)

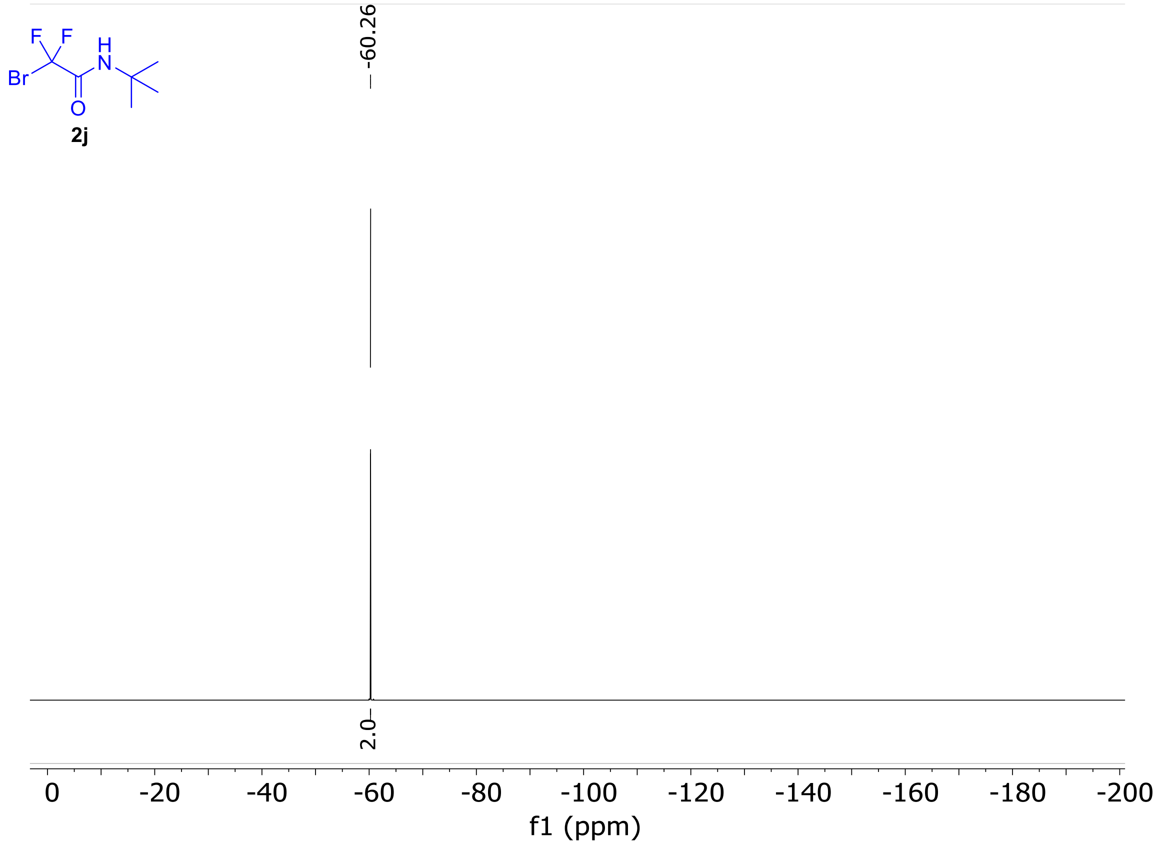

^{1}**H NMR** spectrum of compound **S1** (400 MHz, DMSO-d_6)

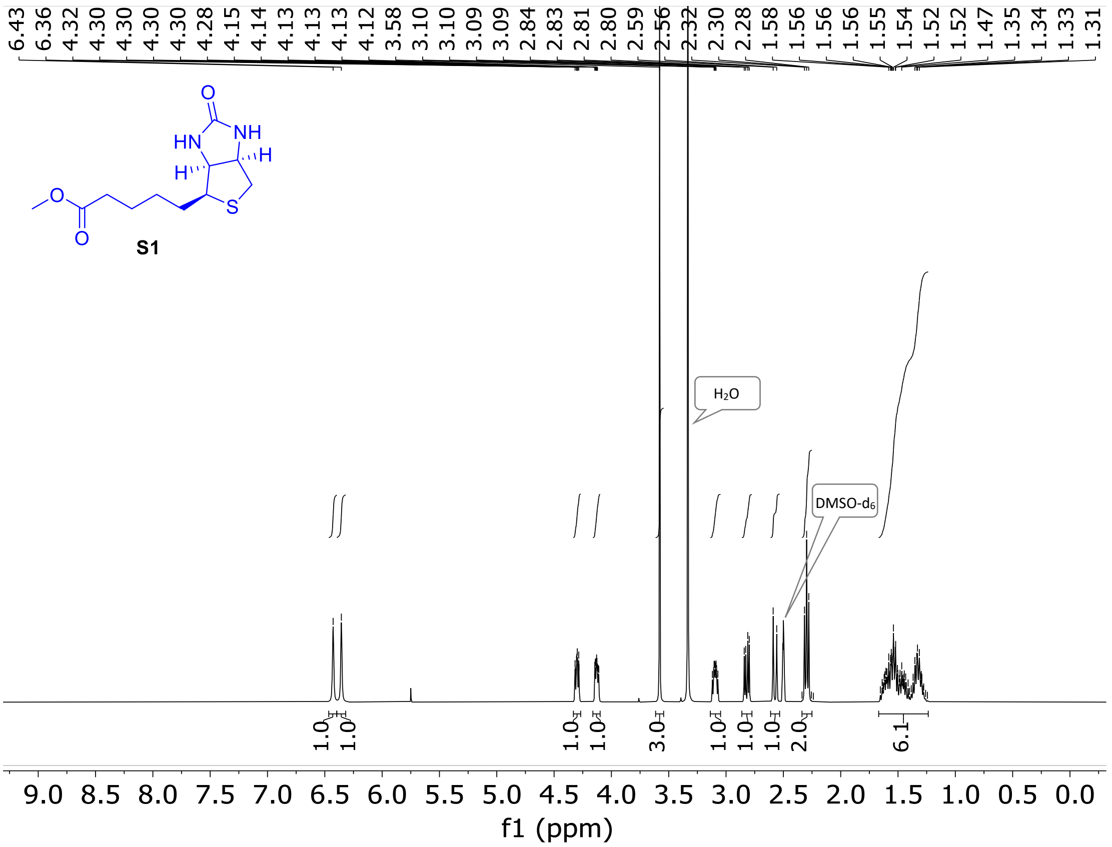

^{1}**H NMR** spectrum of compound **S2** (400 MHz, DMSO-d_6)

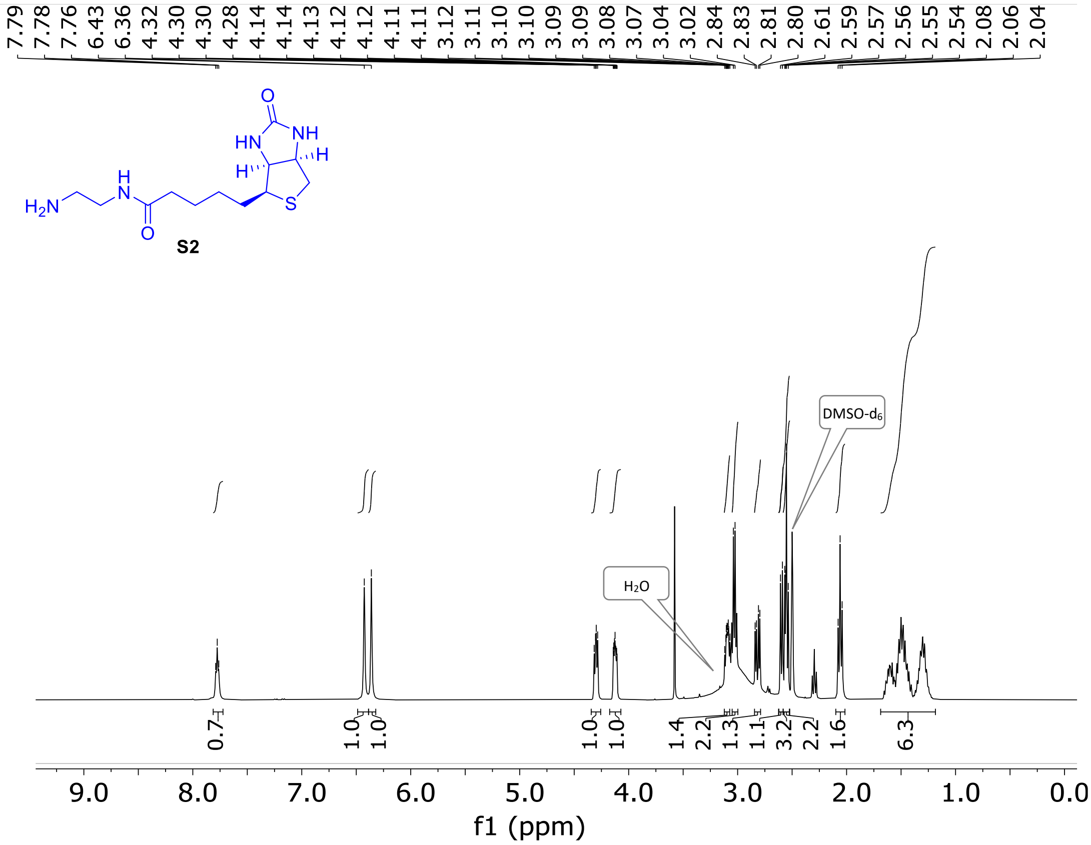

^{1}H NMR spectrum of compound **2k** (400 MHz, DMSO-d_6)

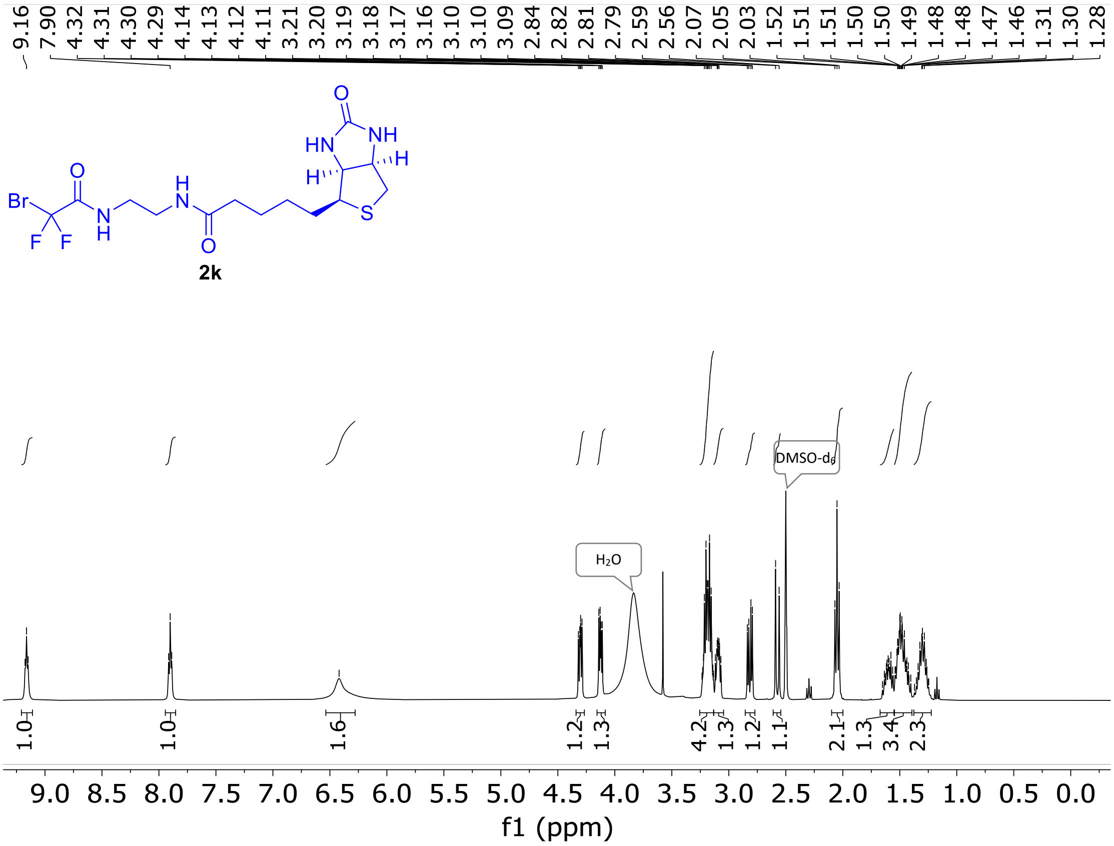

^{13}C NMR spectrum of compound **2k** (101 MHz, DMSO-d_6)

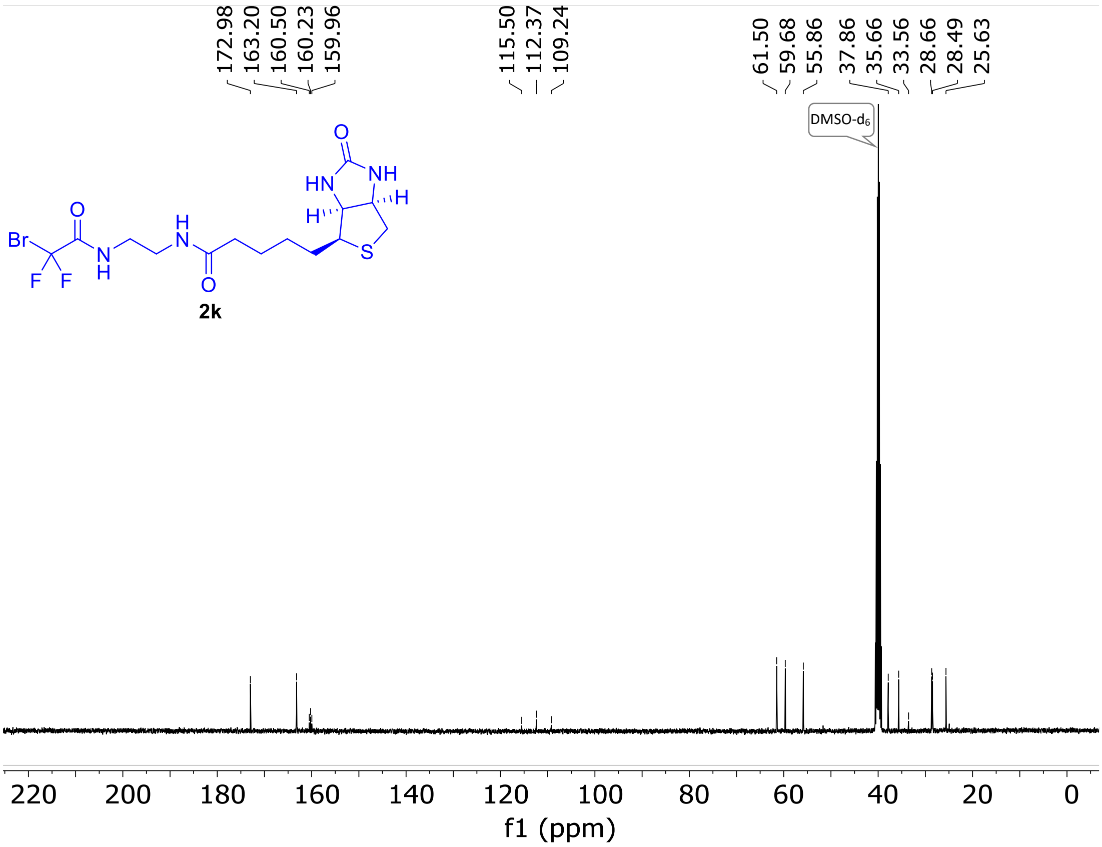

^{19}F NMR spectrum of compound **2k** (376 MHz, DMSO-d_6)

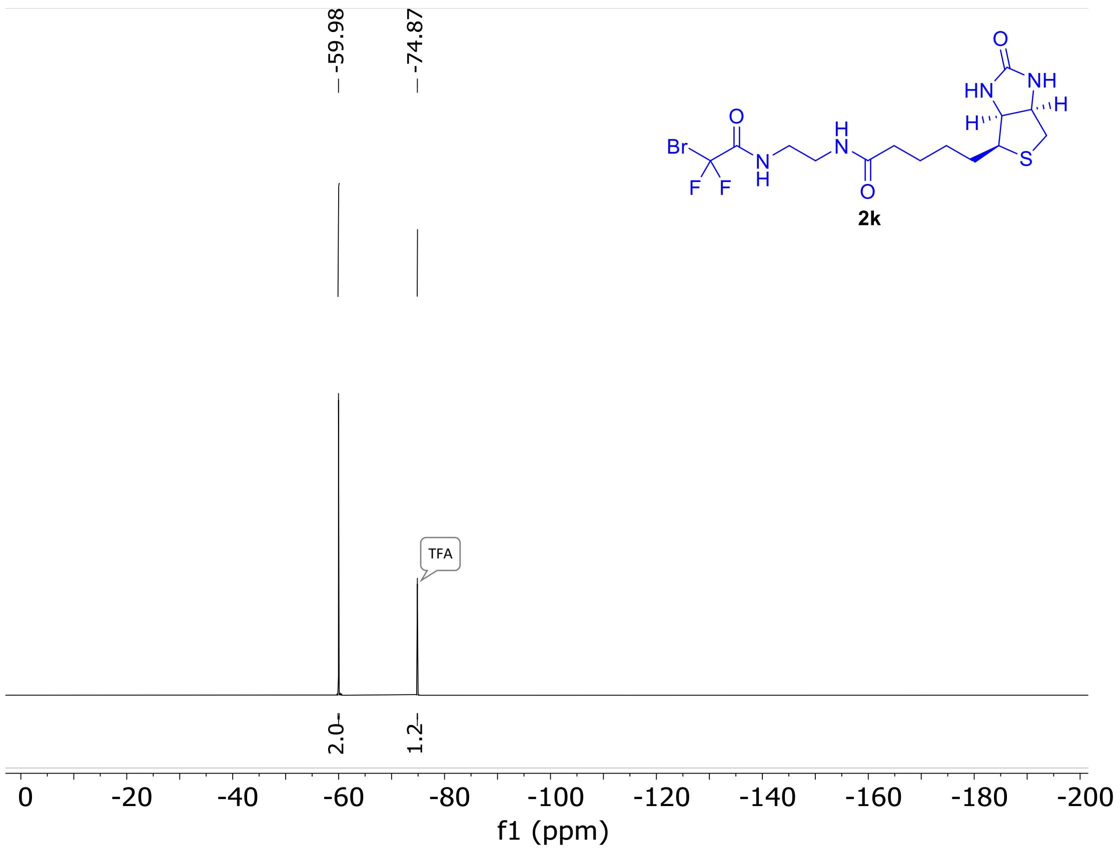

^{1}H NMR spectrum of compound **2l** (400 MHz, DMSO-d_6)

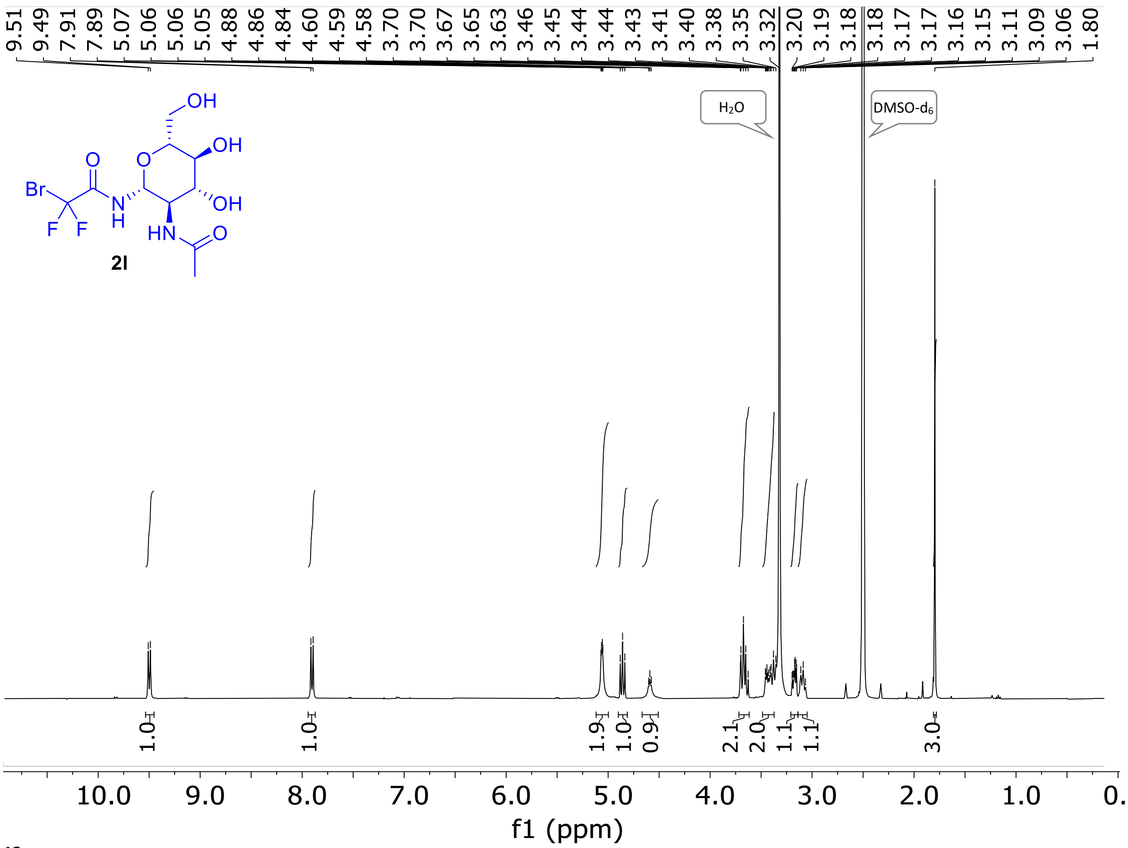

^{13}C NMR spectrum of compound **2l** (101 MHz, DMSO-d_6)

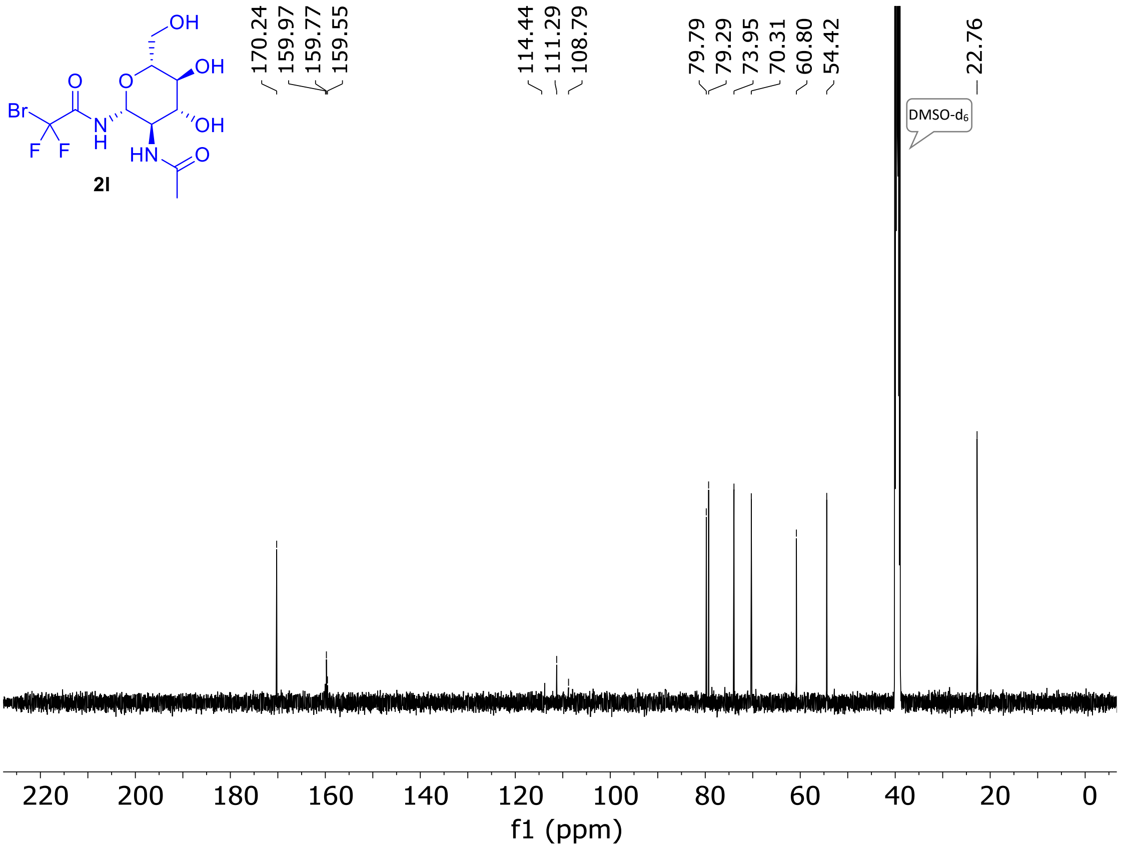

^{19}**F NMR** spectrum of compound **2l** (376 MHz, DMSO-d_6)

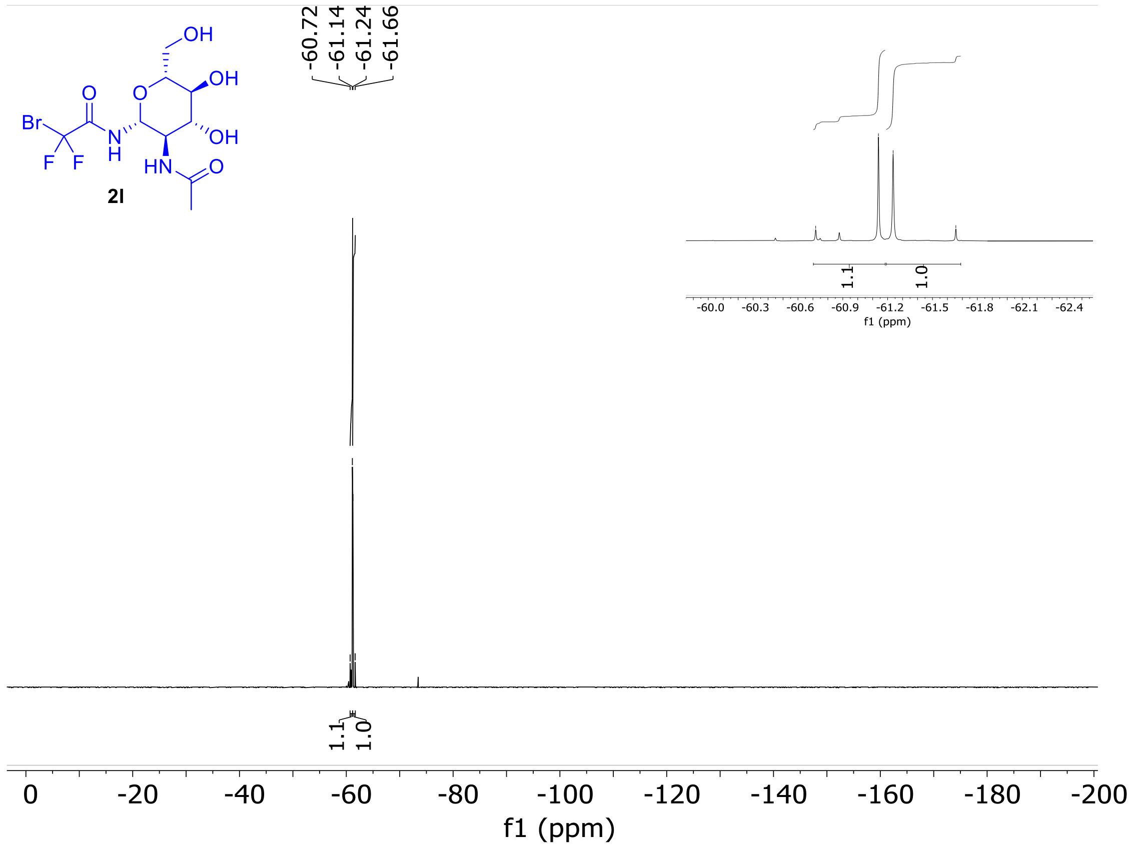

Photochemical alkylation of Ac-Trp-OEt

^{19}F NMR spectrum of crude product **3a** using α,α,α TFT as the internal standard (376 MHz, DMSO)

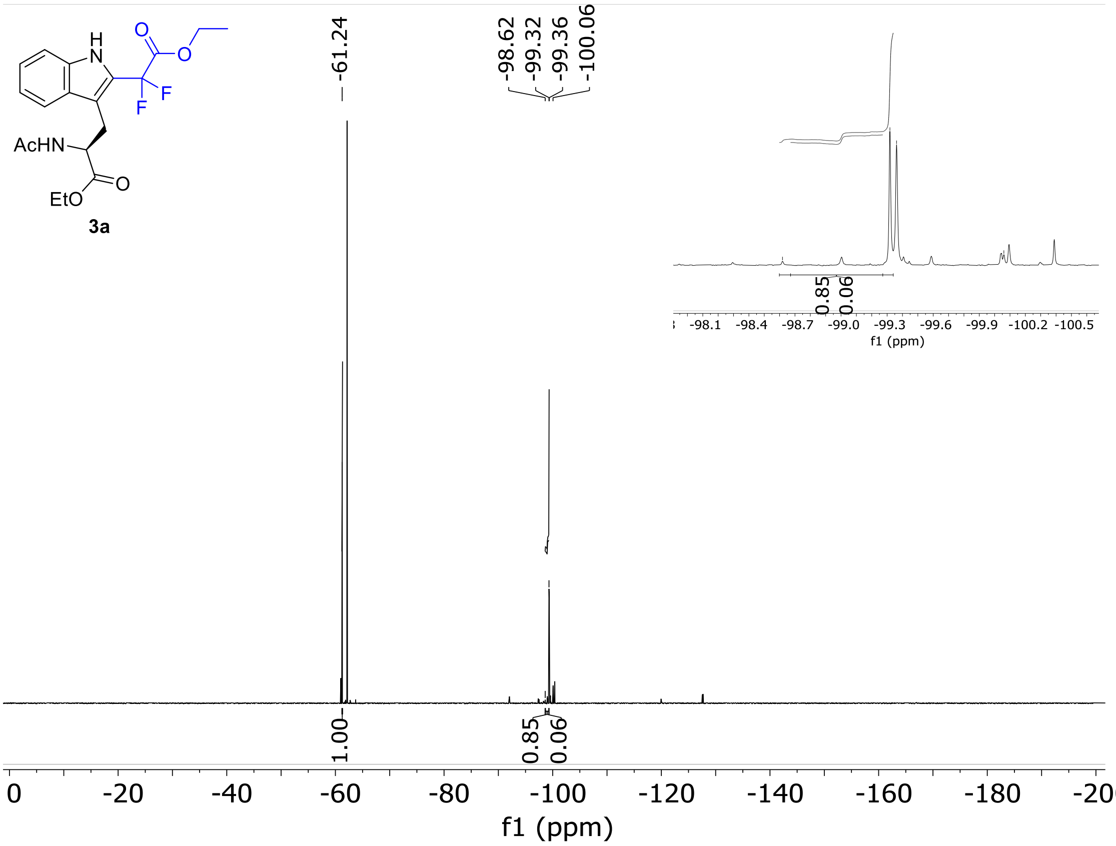

^{19}F NMR spectrum of crude product **3b** using α,α,α TFT as the internal standard (376 MHz, DMSO)

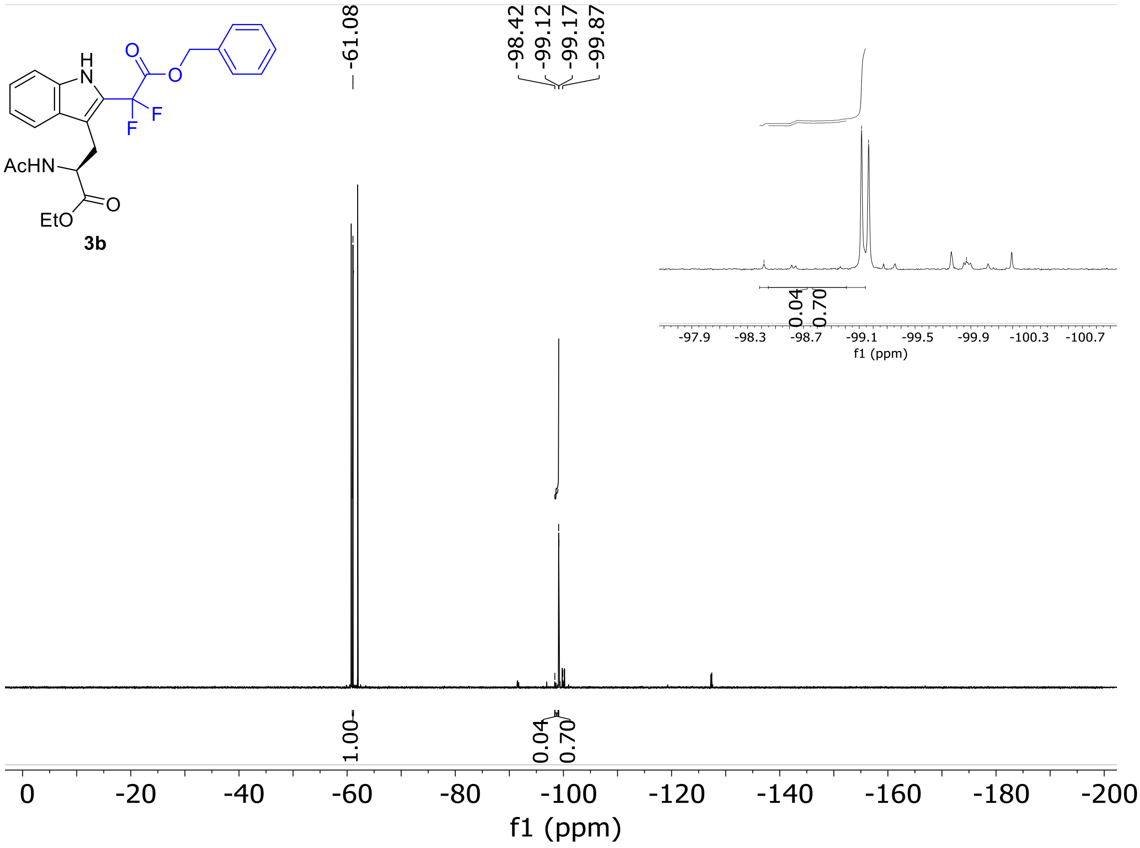

^{19}**F NMR** spectrum of crude product **3c** using α,α,α TFT as the internal standard (376 MHz, DMSO)

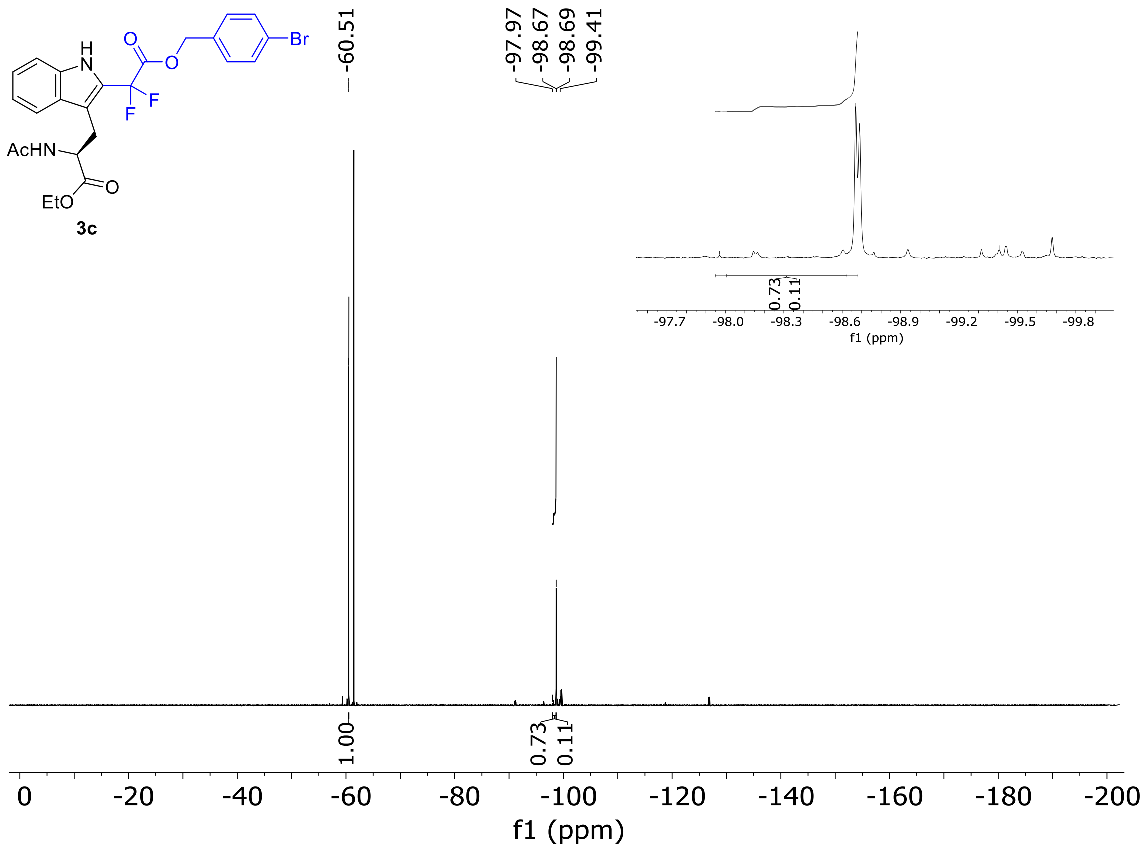

^{19}**F NMR** spectrum of crude product **3d** using α,α,α TFT as the internal standard (376 MHz, DMSO)

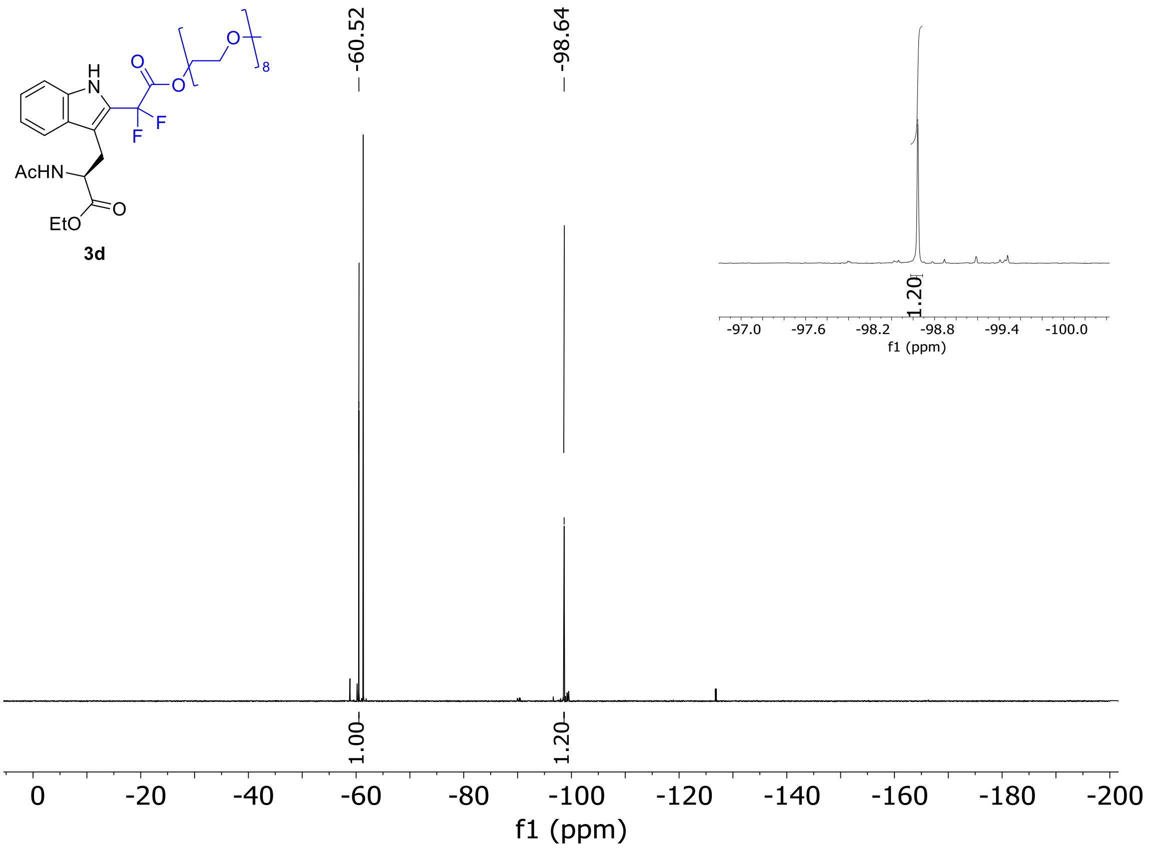

^{19}F NMR spectrum of crude product **3e** (376 MHz, DMSO) using α,α,α TFT as the internal standard

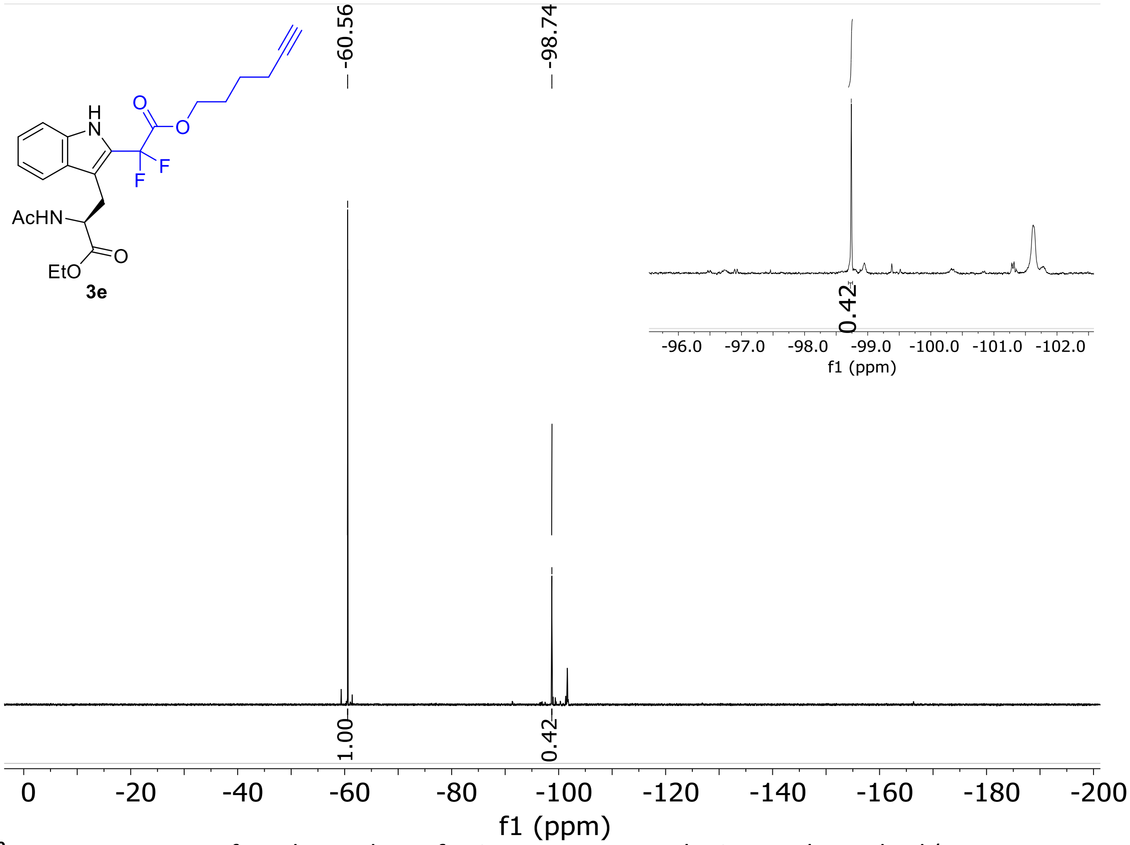

^{19}F NMR spectrum of crude product **3f** using α,α,α TFT as the internal standard (376 MHz, DMSO)

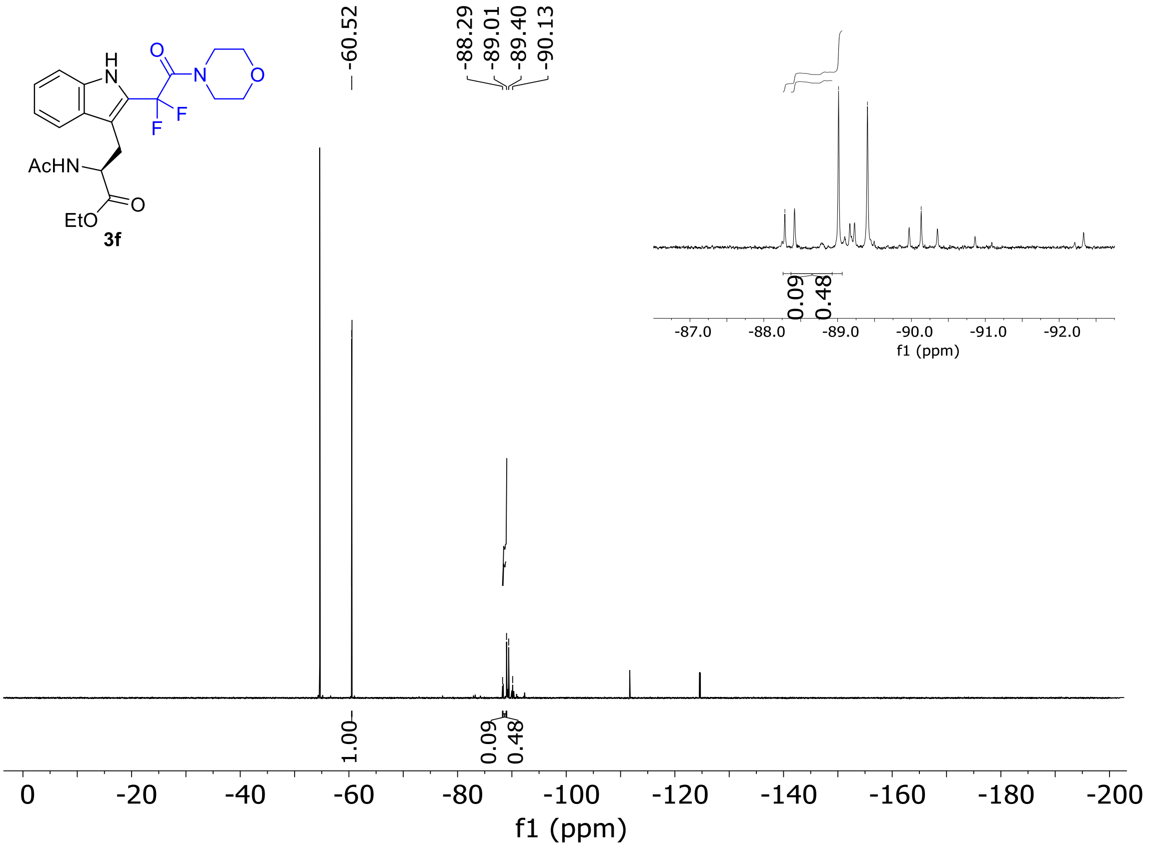

^{19}F NMR spectrum of crude product **3g** using α,α,α TFT as the internal standard (376 MHz, DMSO)

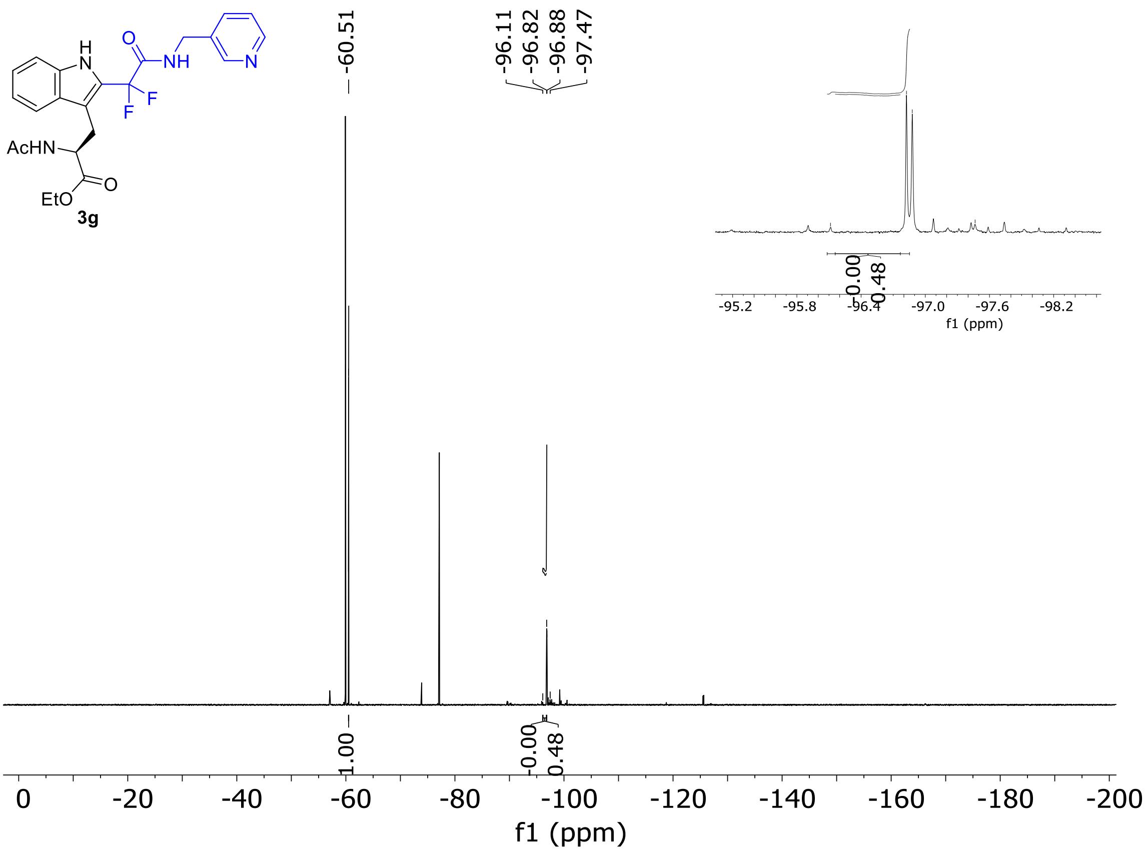

^{19}F NMR spectrum of crude product **3h** using α,α,α TFT as the internal standard (376 MHz, DMSO)

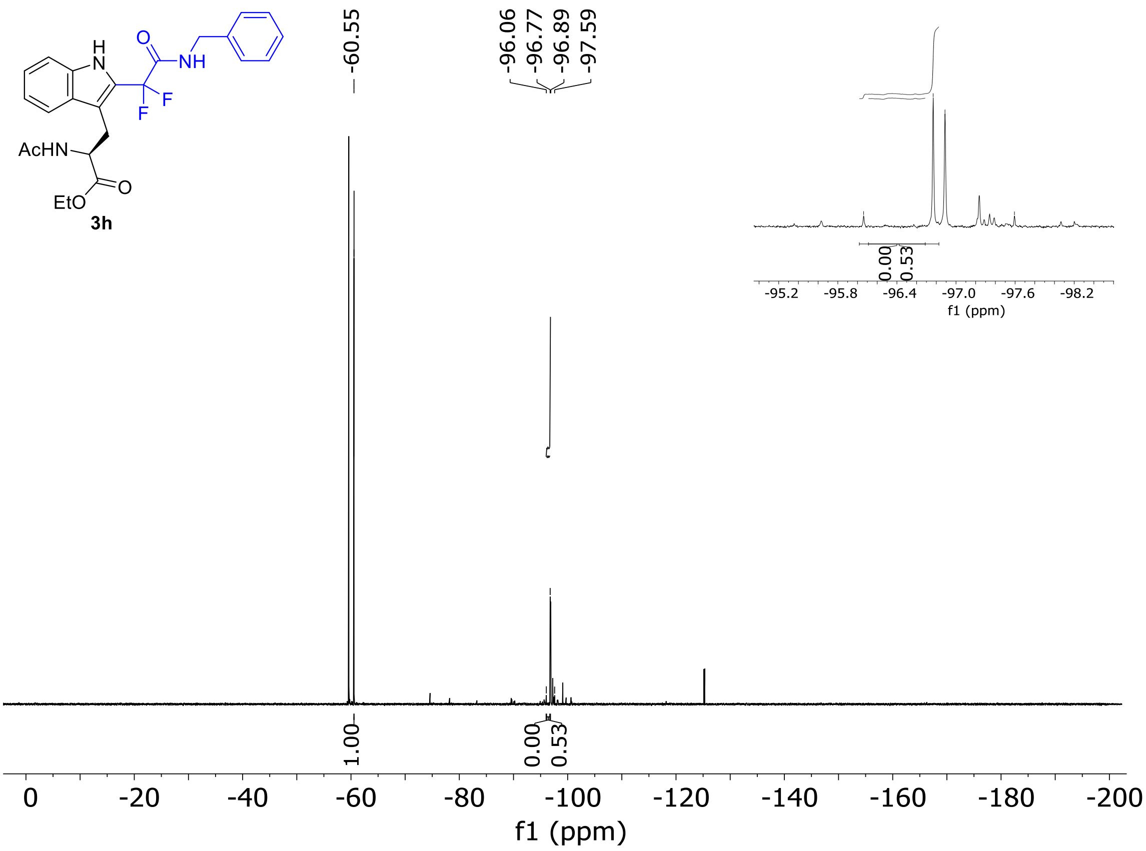

^{19}**F NMR** spectrum of crude product **3i** using α,α,α TFT as the internal standard (376 MHz, DMSO)

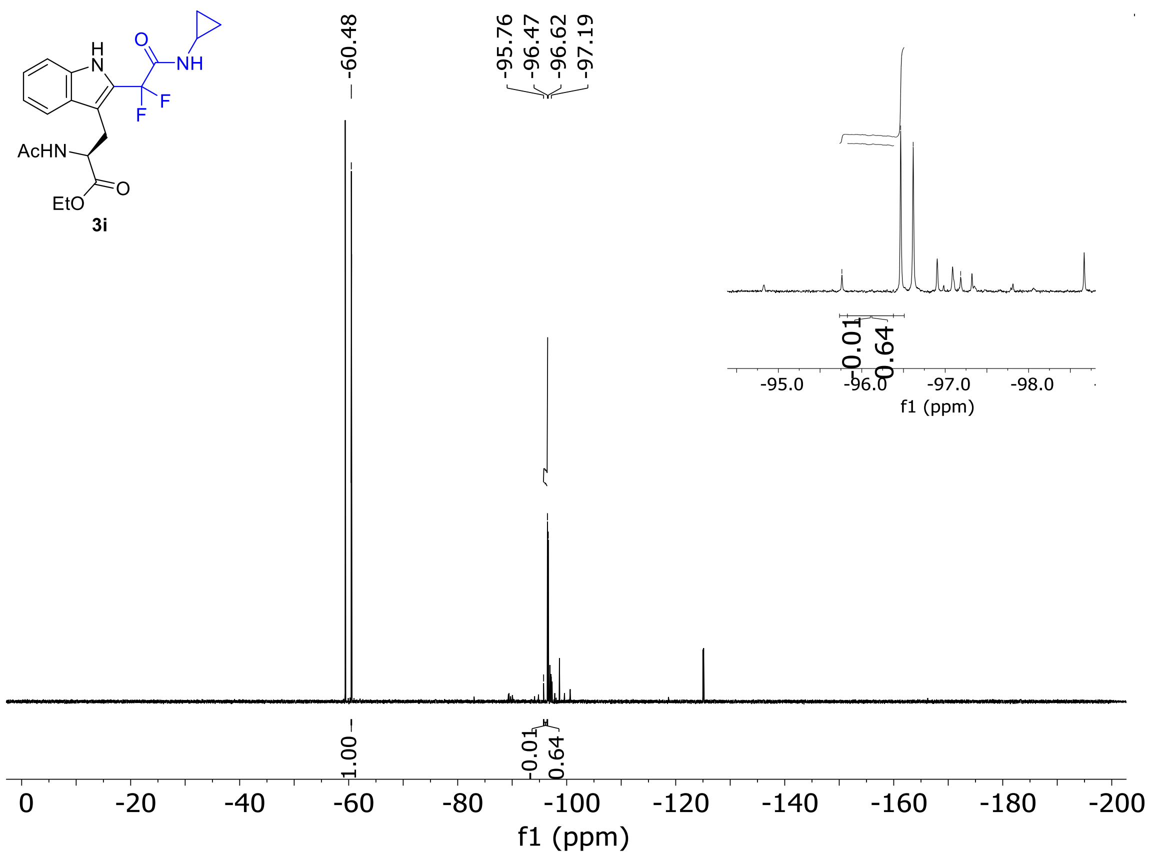

^{19}**F NMR** spectrum of crude product **3j** using α,α,α TFT as the internal standard (376 MHz, DMSO)

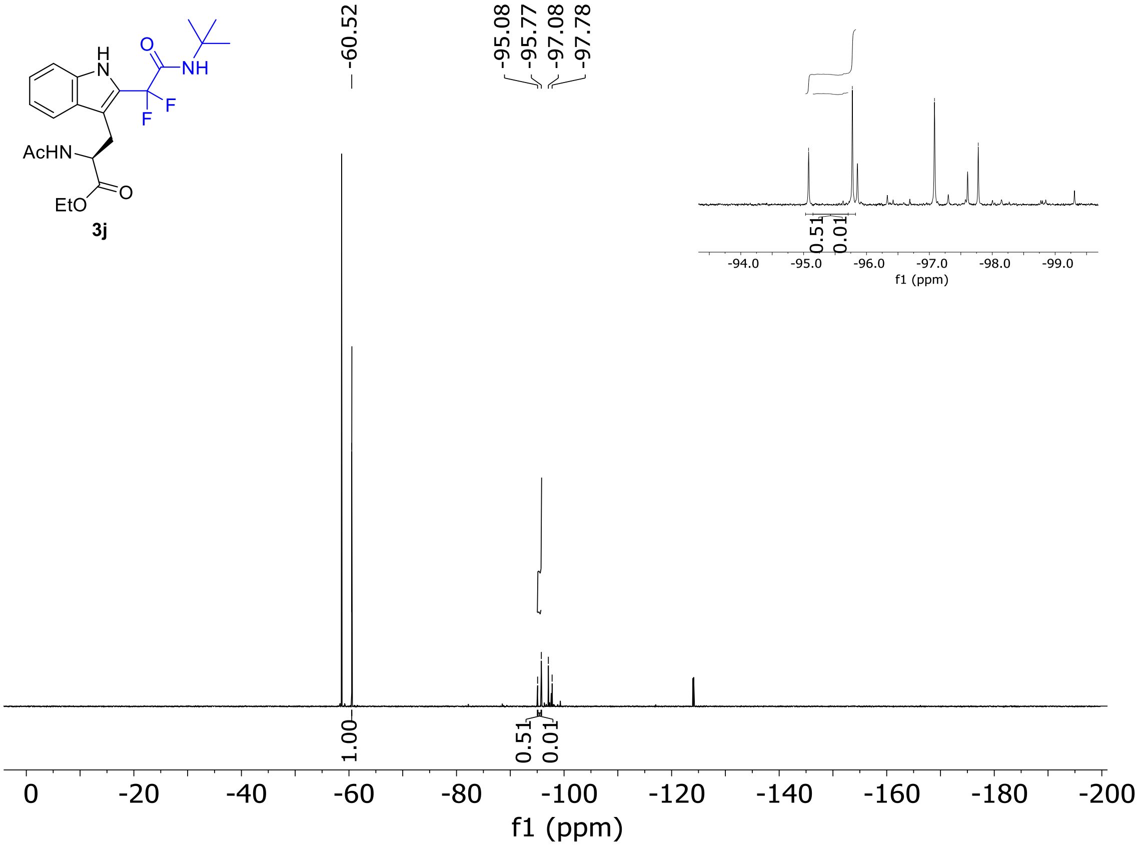

^{19}**F NMR** spectrum of crude product **3k** using α,α,α TFT as the internal standard (376 MHz, DMSO)

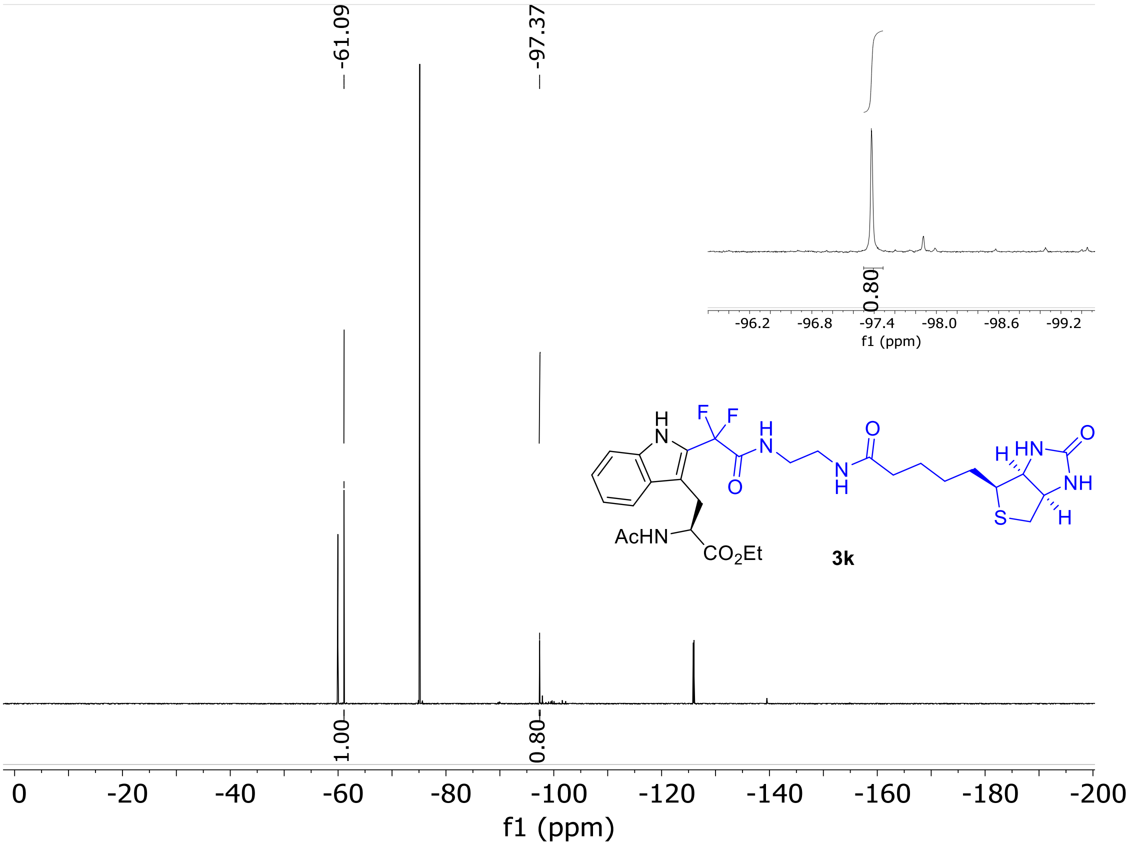

^{1}H NMR spectrum of product **3a** (400 MHz, $CDCl_3$)

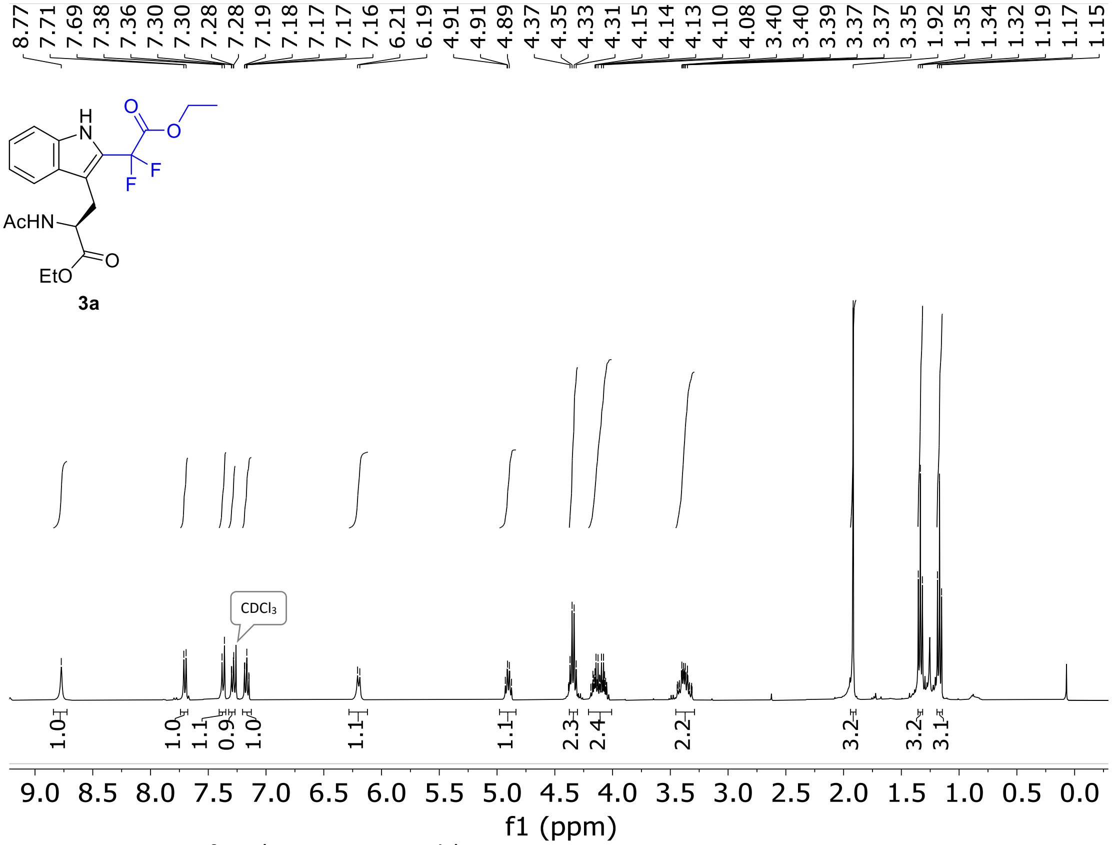

^{13}C NMR spectrum of **3a** (101 MHz, $CDCl_3$)

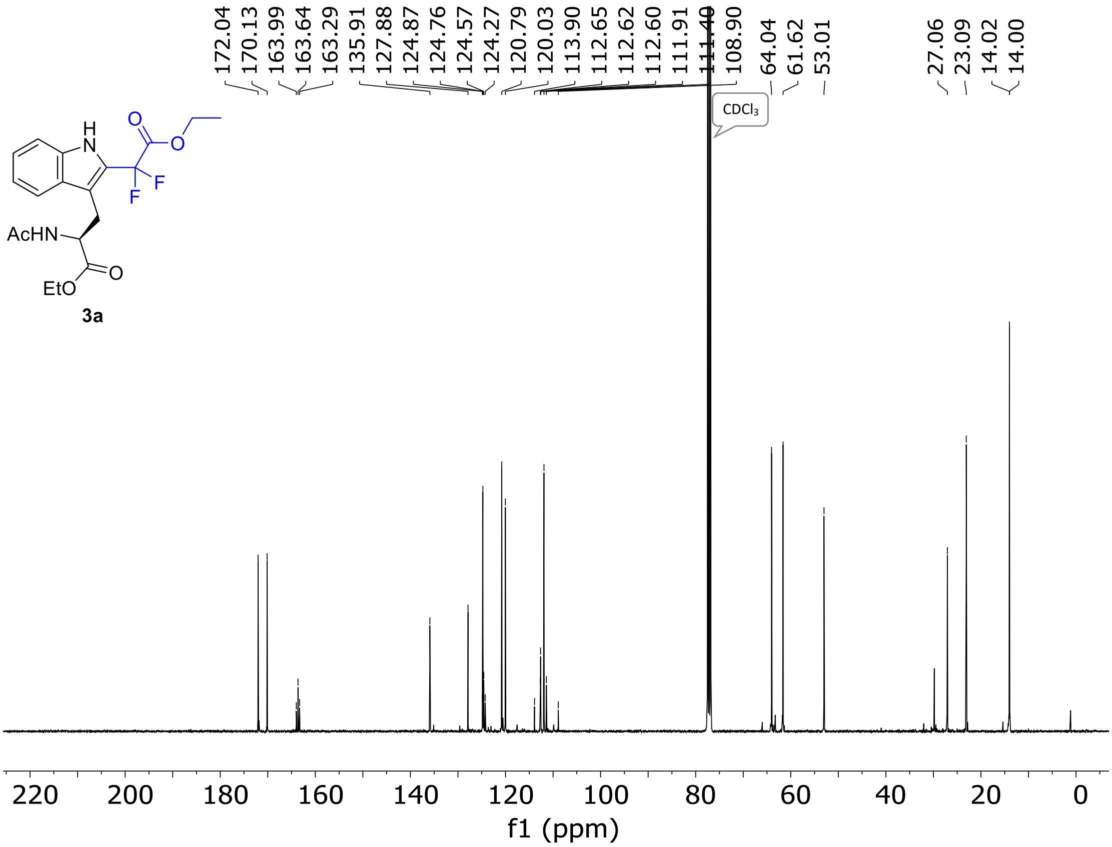

^{19}F NMR spectrum of product **3a** (377 MHz, $CDCl_3$)

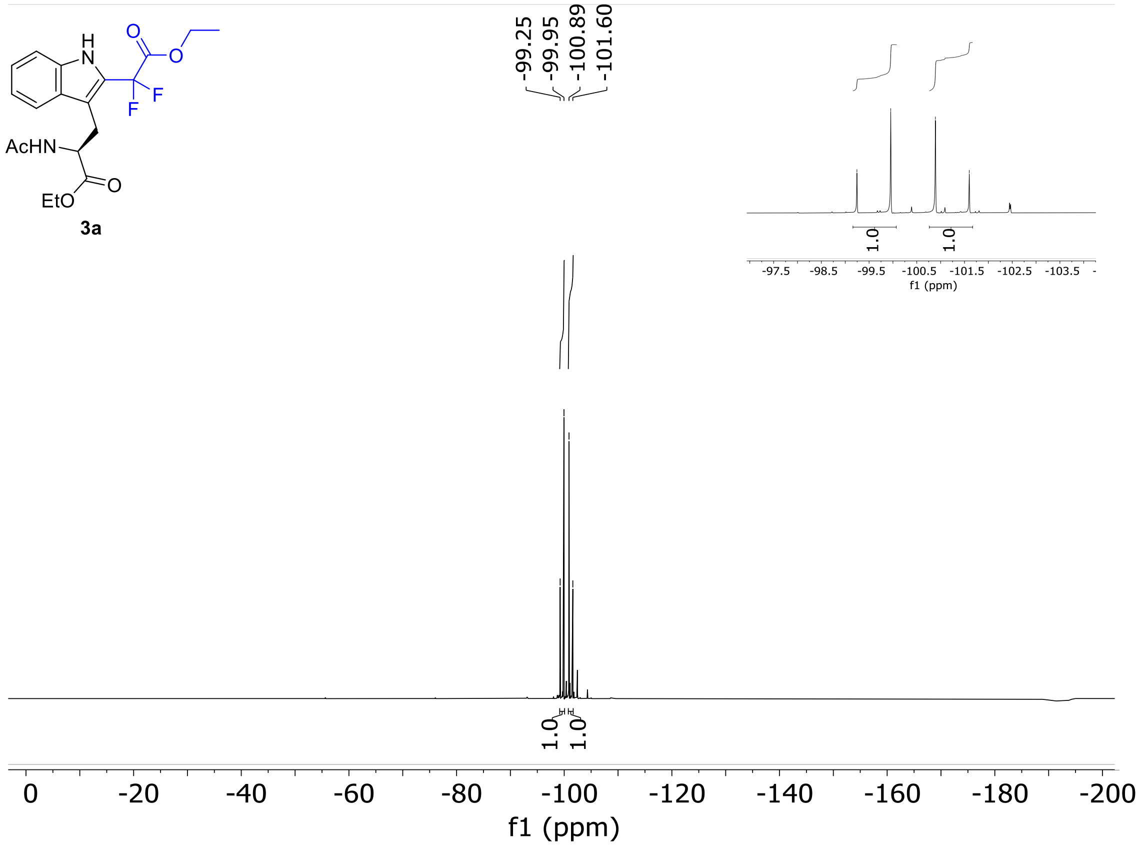

^{1}**H NMR** spectrum of product **3b** (400 MHz, $CDCl_3$)

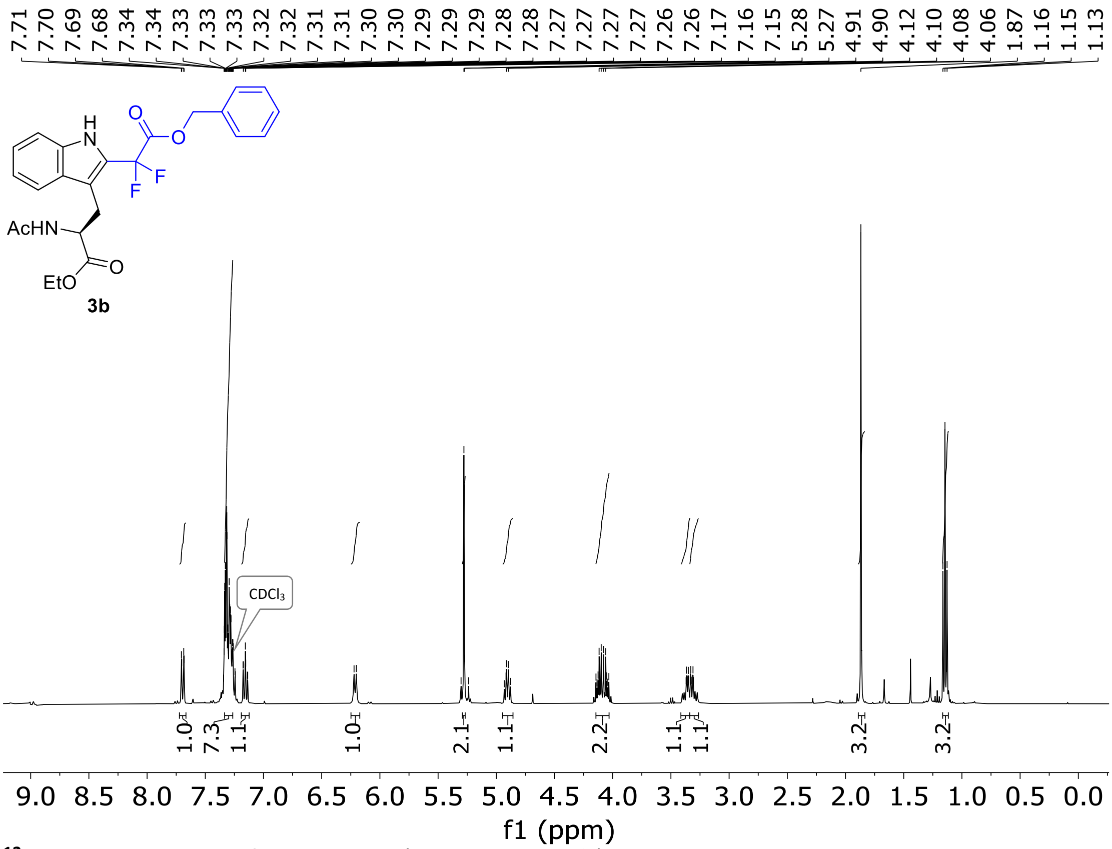

^{13}**C NMR** spectrum of product **3b** (101 MHz, $CDCl_3$)

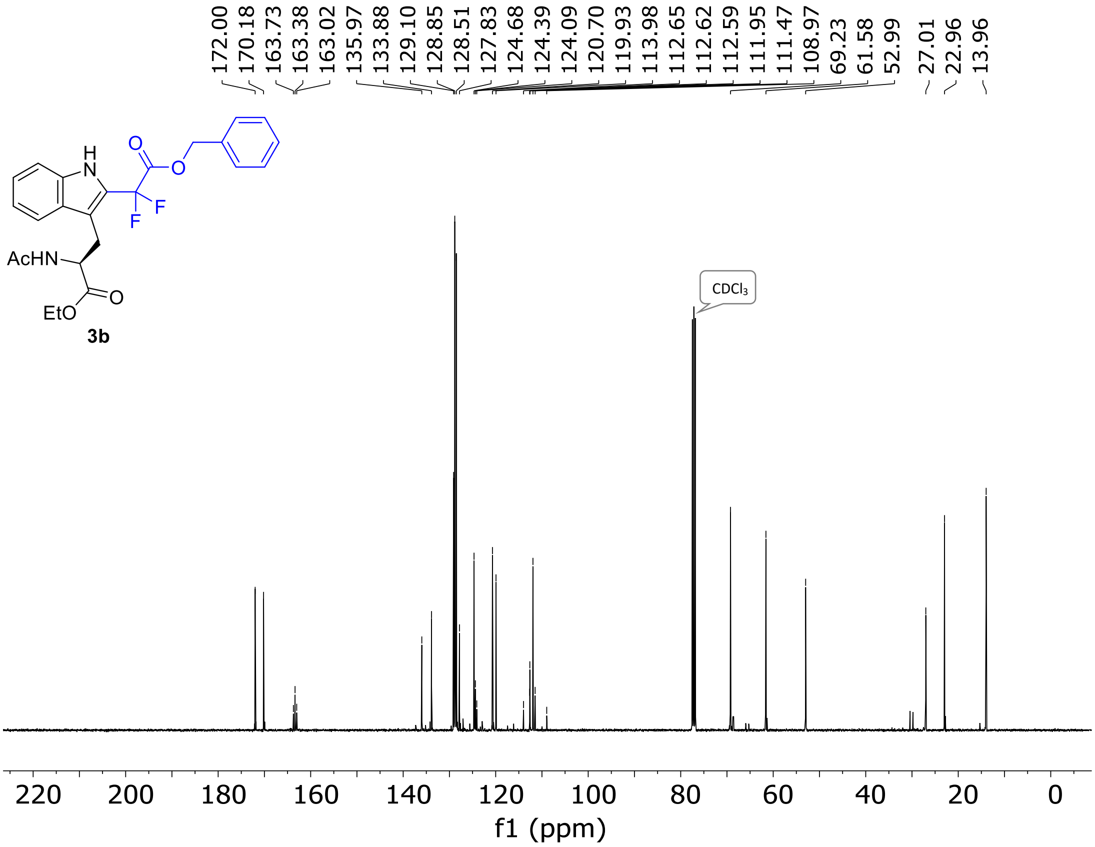

^{19}F NMR spectrum of product **3b** (376 MHz, $CDCl_3$)

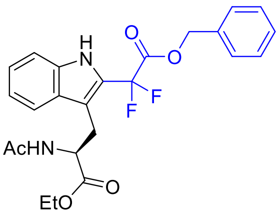
3b

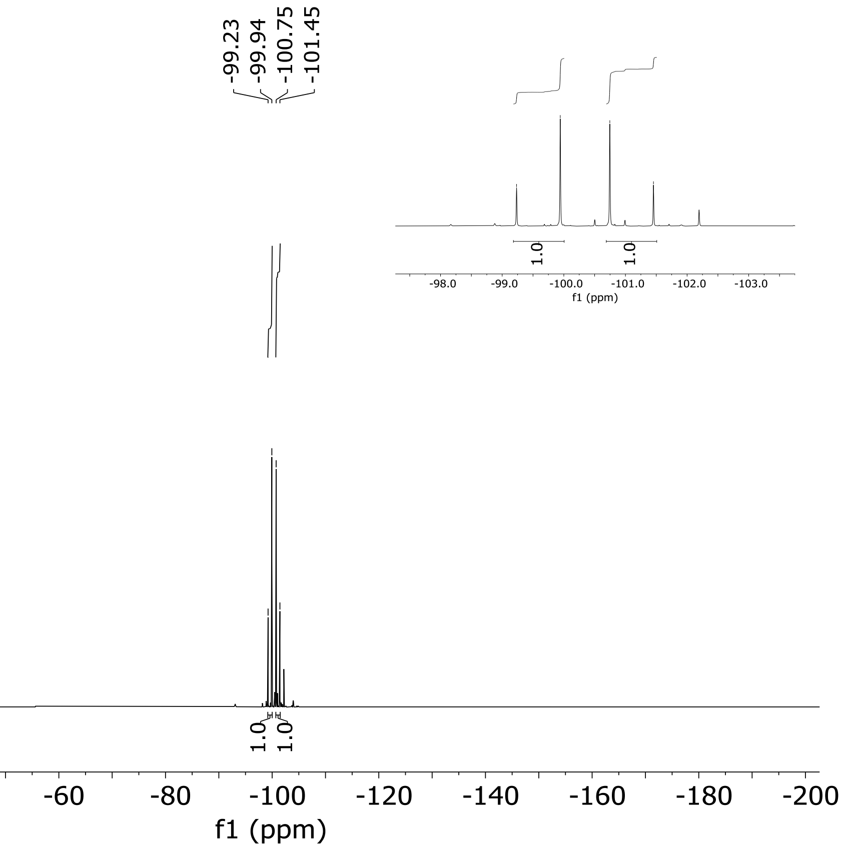

^{1}H NMR spectrum of product **3h** (400 MHz, $CDCl_3$)

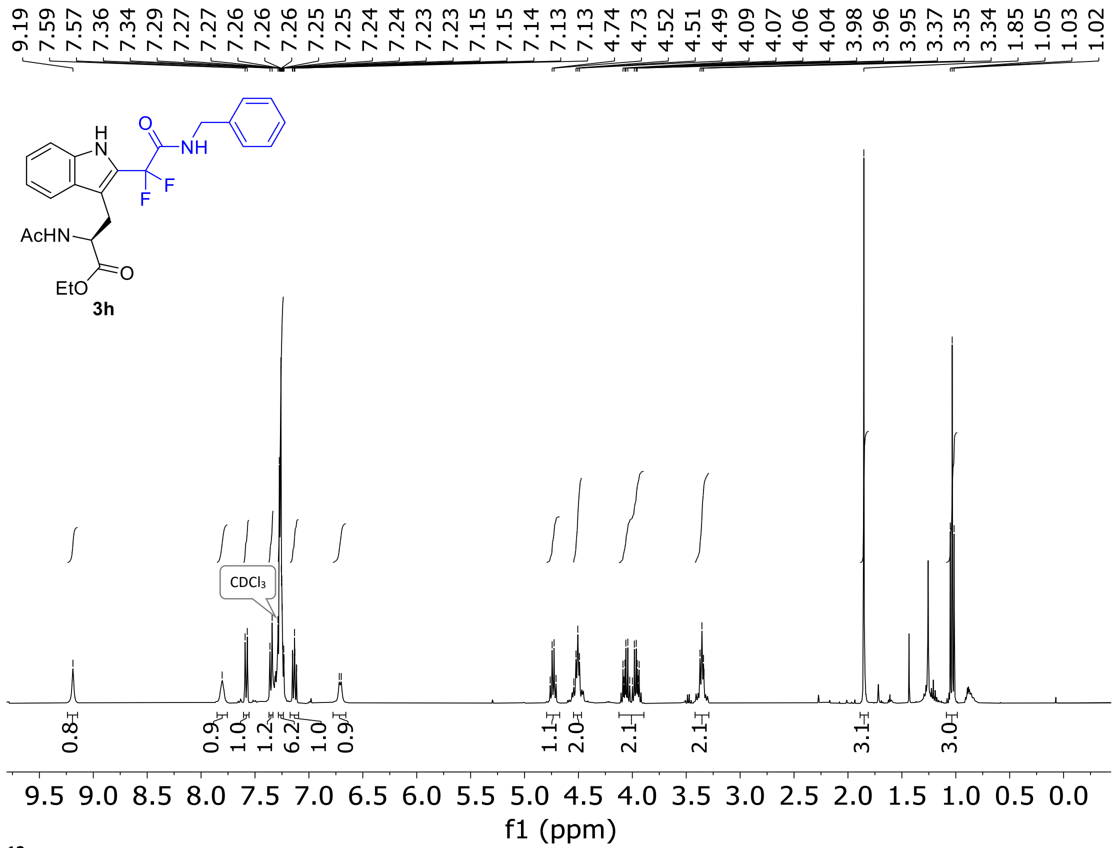

^{13}C NMR spectrum of product **3h** (101 MHz, $CDCl_3$)

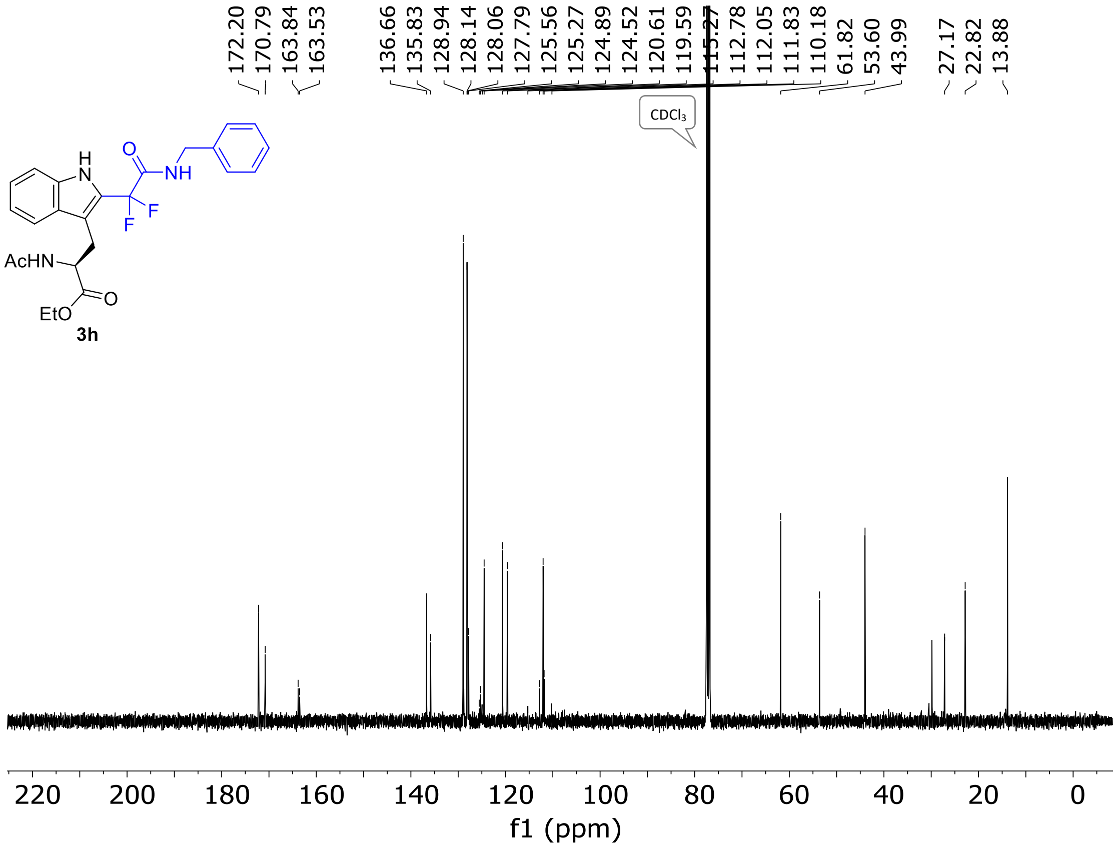

^{19}**F NMR** spectrum of product **3h** (376 MHz, $CDCl_3$)

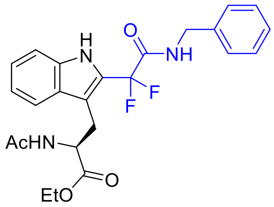
3h

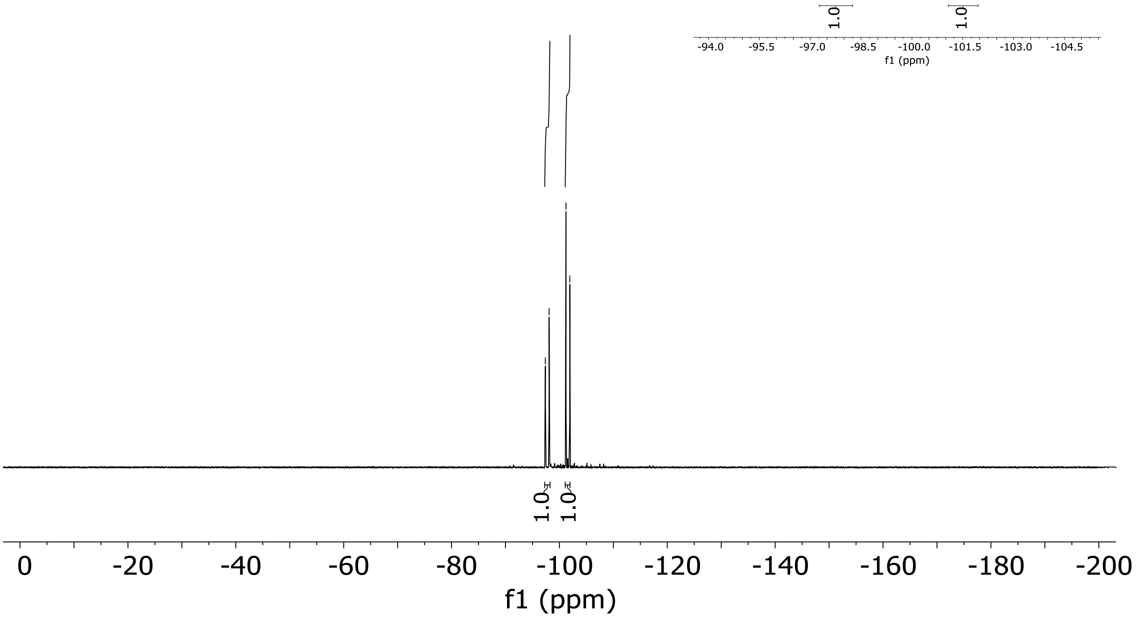

Peptide substrates

^{1}H NMR spectrum of Ac-WHISKEY-NH_2 **4** (400 MHz, CD_3OD)

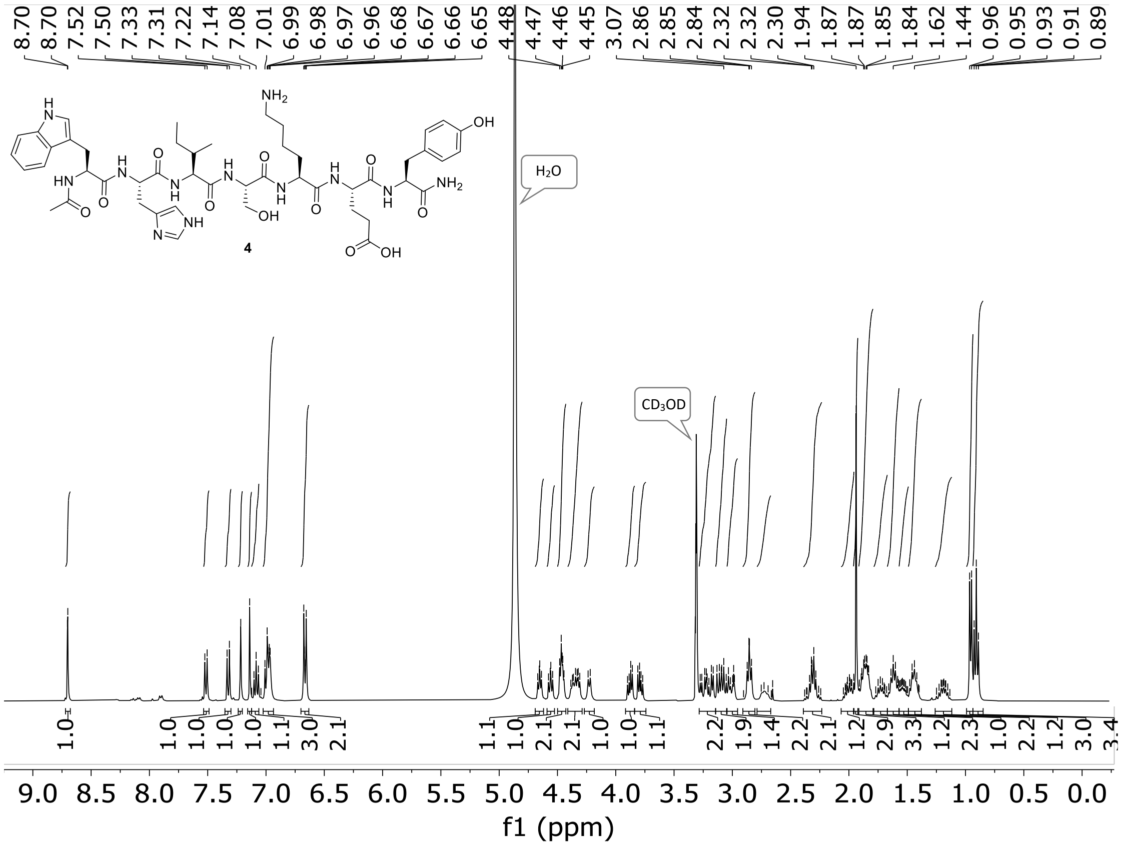

^{1}H NMR spectrum of Ac-HISKEY-NH_2 **6** (400 MHz, CD_3OD)

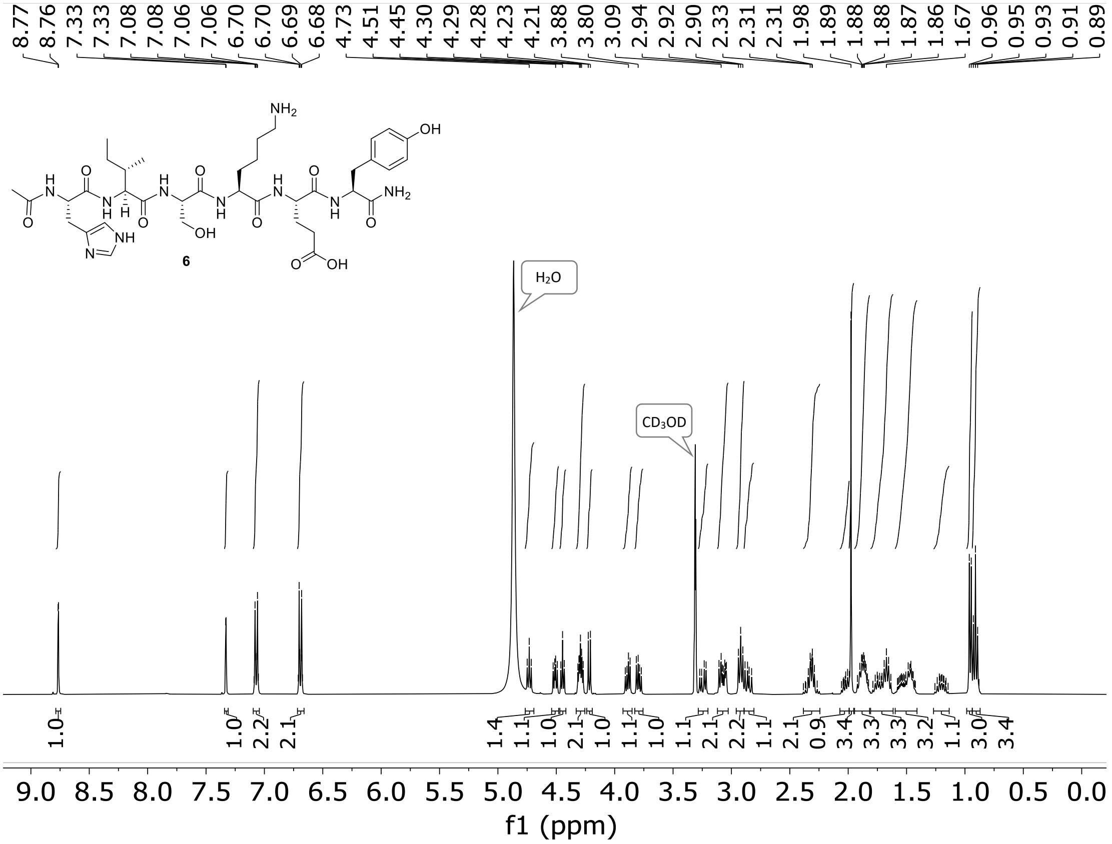

¹H NMR spectrum of H-WHISKEY-NH$_2$ **7** (400 MHz, CD_3OD)

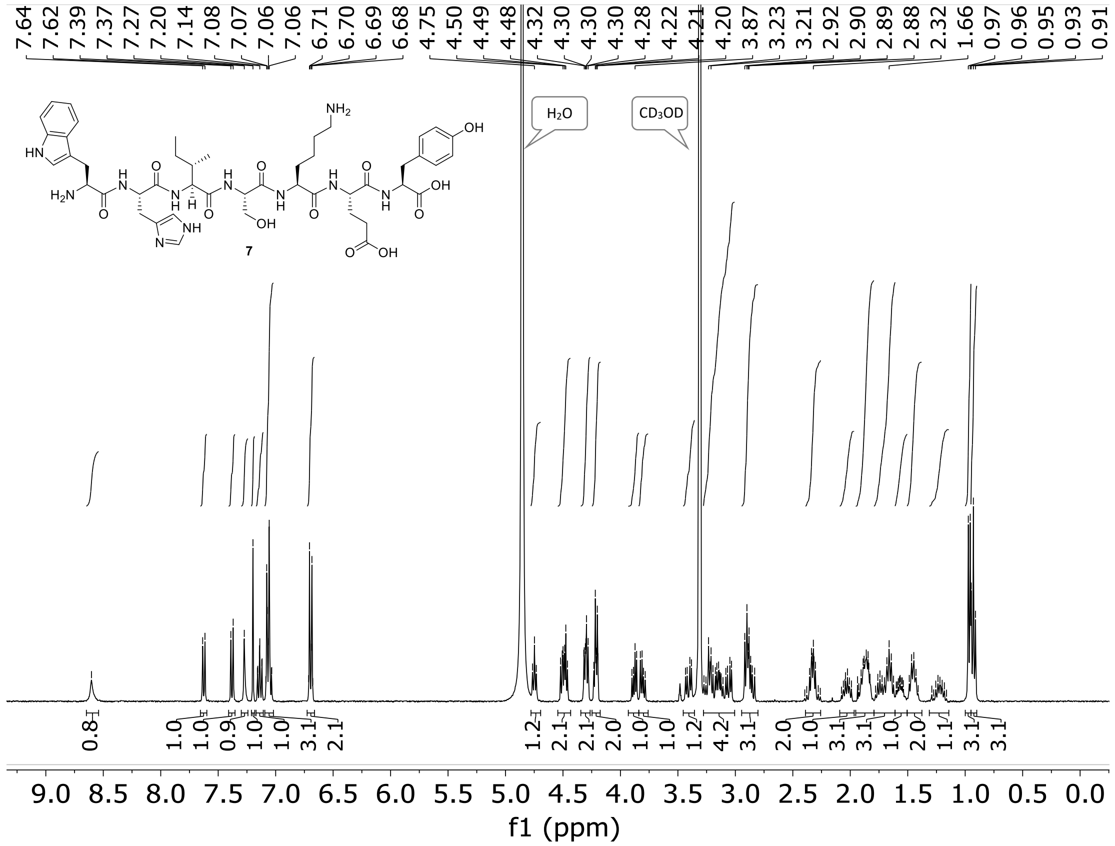

¹H NMR spectrum of Ac-CWHISKEY-NH$_2$ **8** (400 MHz, CD_3OD)

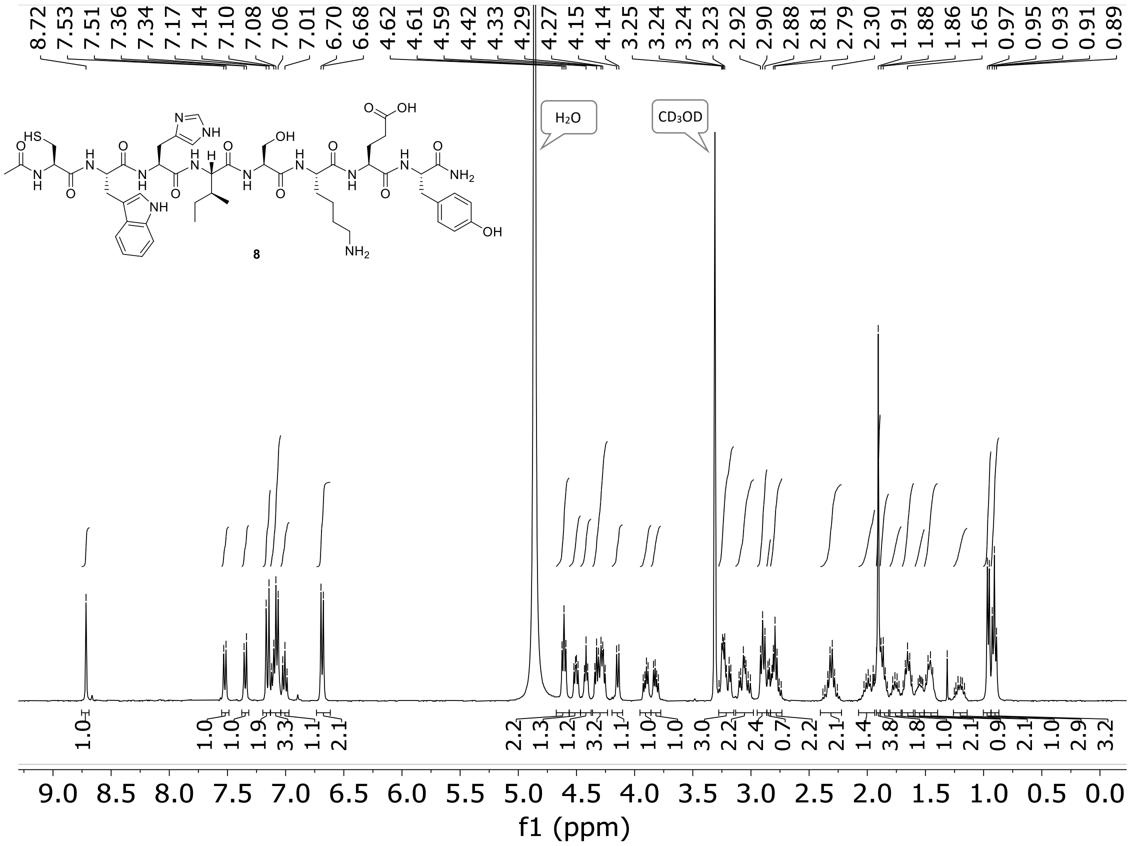

^{1}H NMR spectrum of Ac-WFMTREY-NH_2 **9** (400 MHz, CD_3OD)

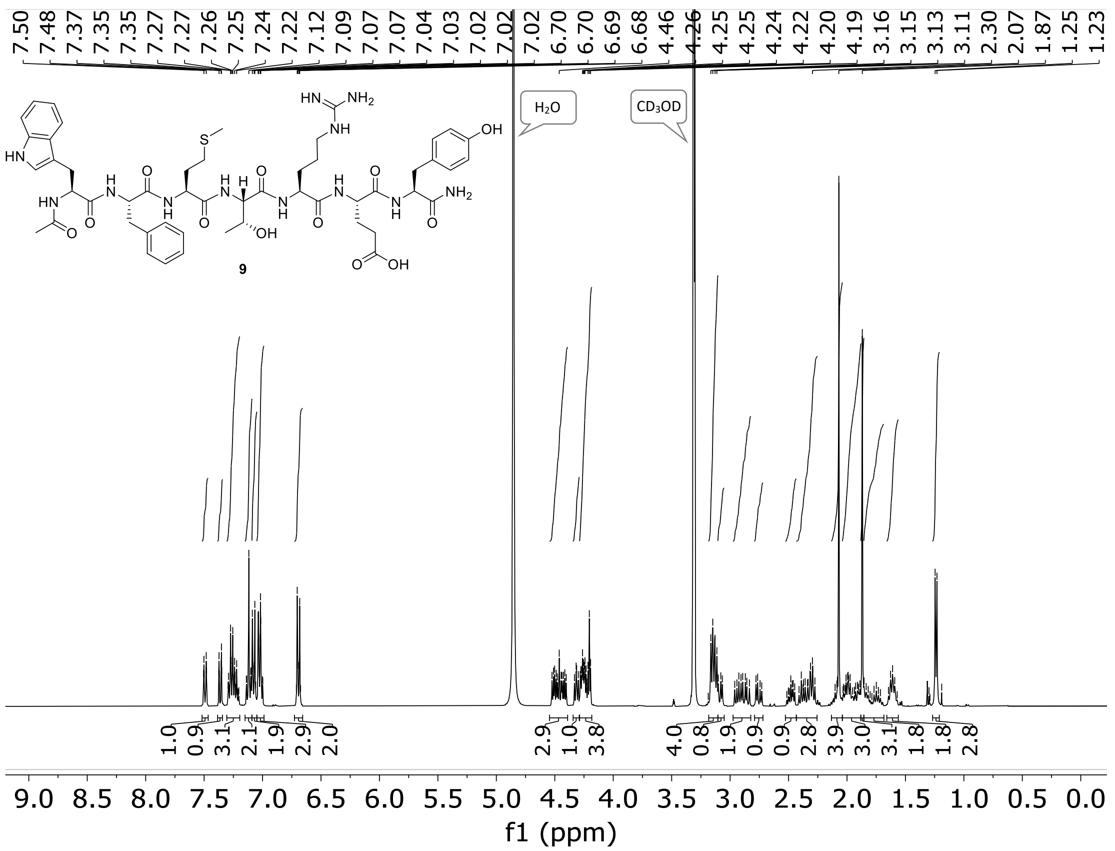

^{1}H NMR spectrum of Ac-WLAHKAL-NH_2 **10** (400 MHz, CD_3OD)

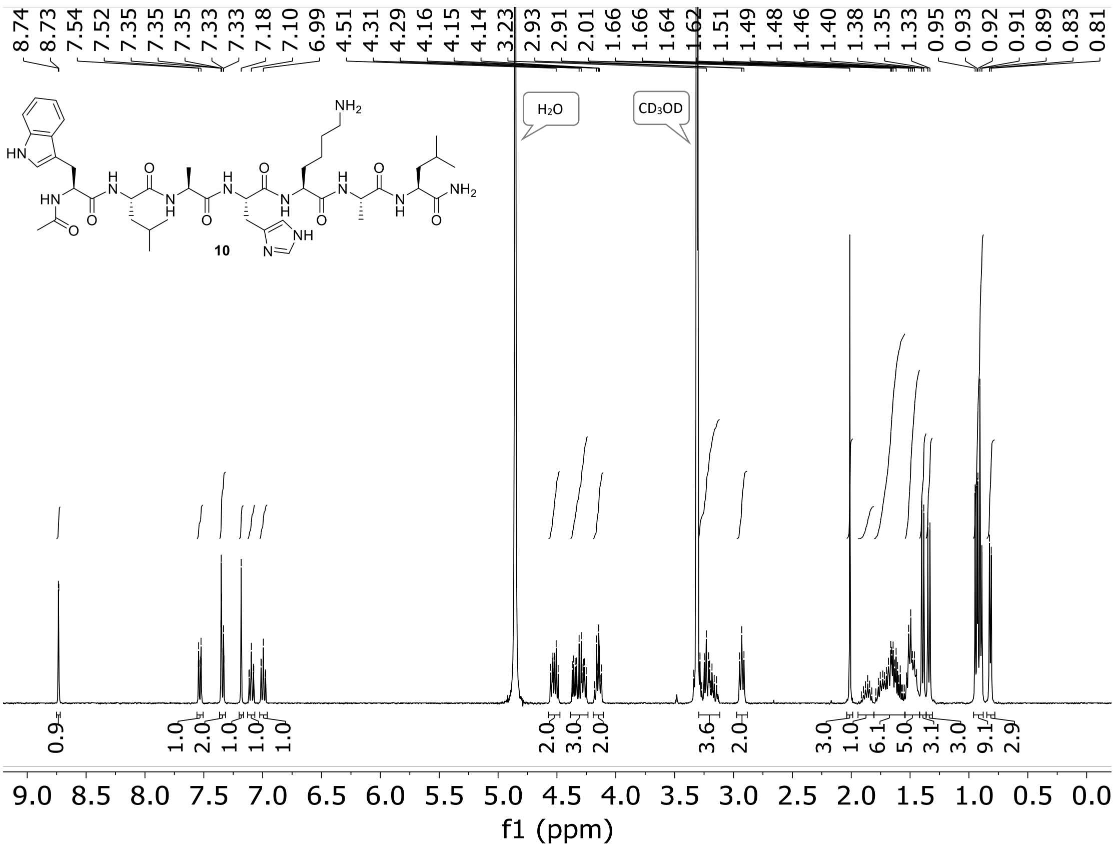

^{1}H NMR spectrum of Ac-VVYPWYQ-NH$_2$ **11** (400 MHz, DMSO-d_6)

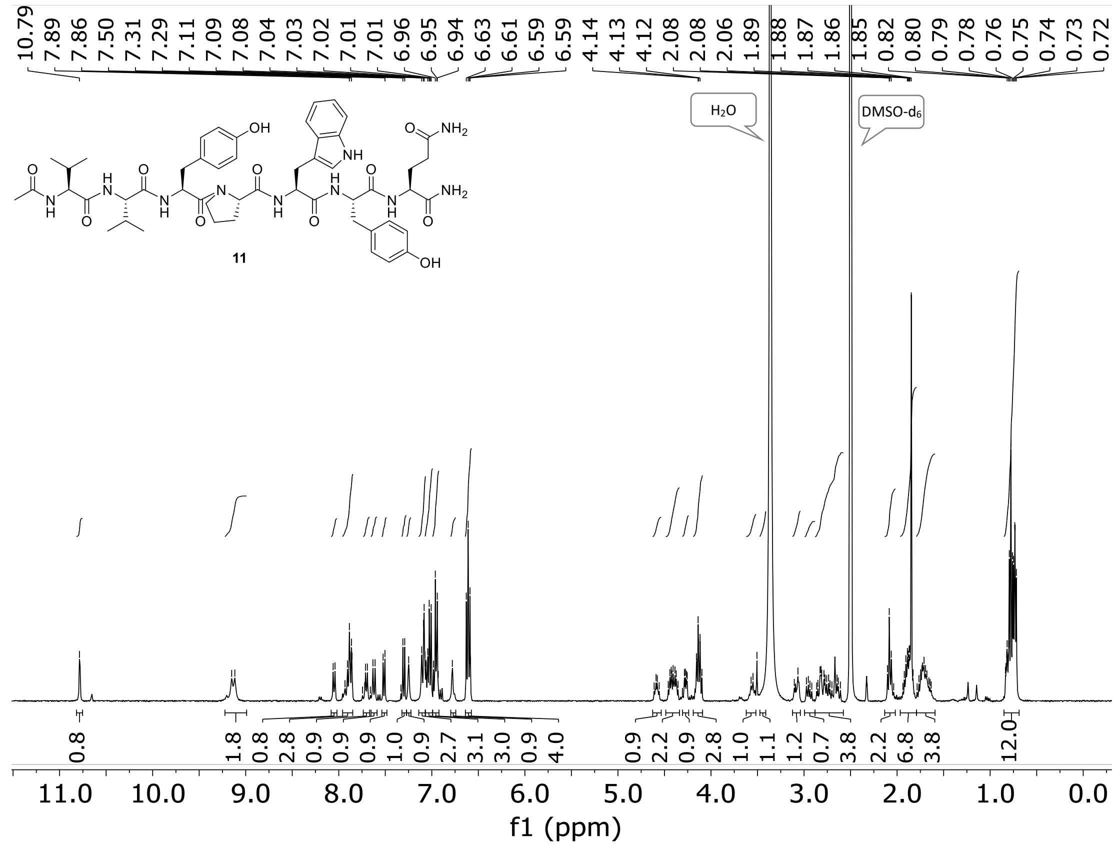

^{1}H NMR spectrum of Ac-DKVGINYW-NH$_2$ **12** (400 MHz, CD_3OD)

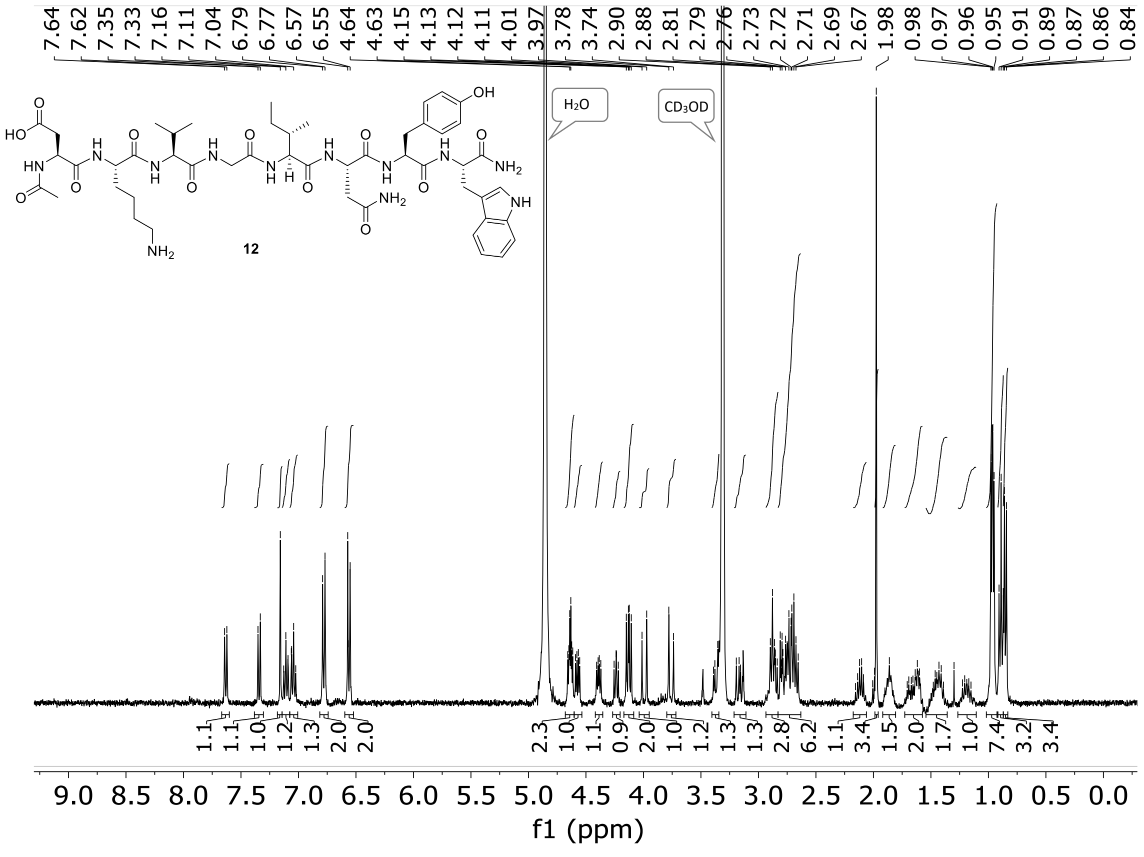

Photochemical alkylation of Trp-containing peptides

^{1}H NMR spectrum of product **5a** (500 MHz, CD_3OD)

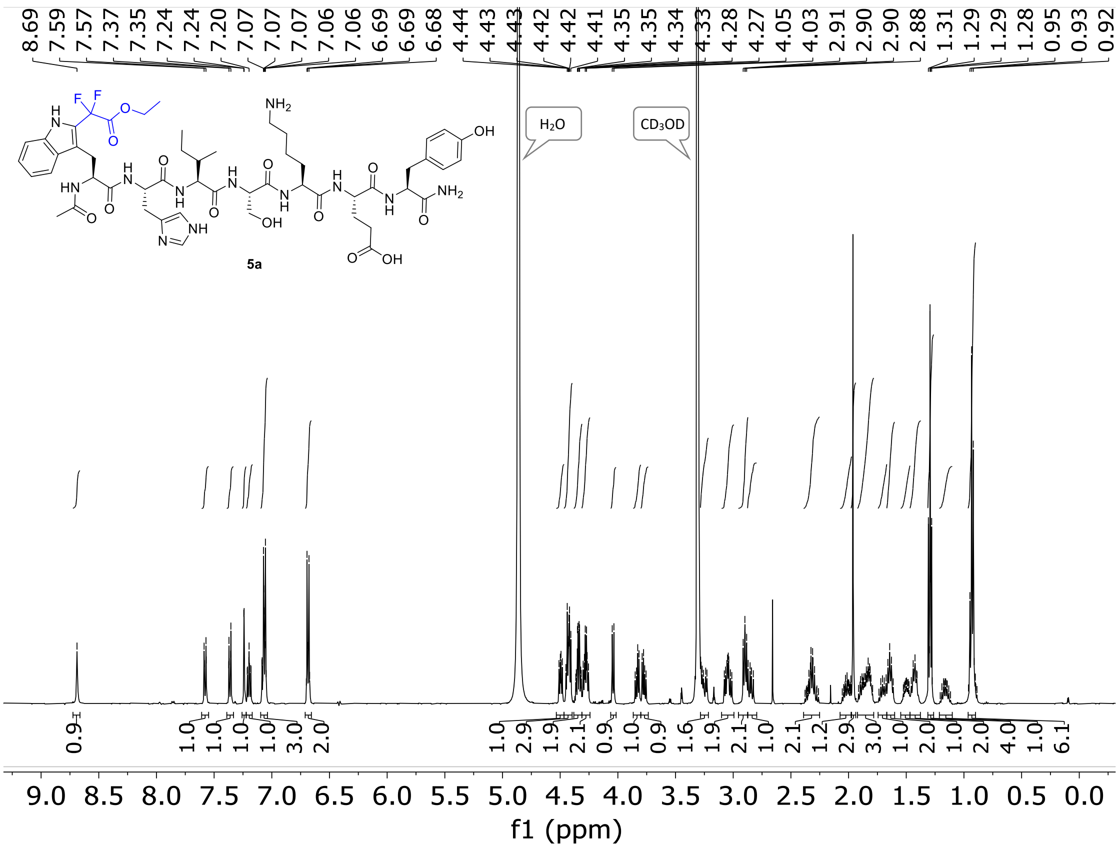

^{19}F NMR spectrum of product **5a** (376 MHz, CD_3OD)

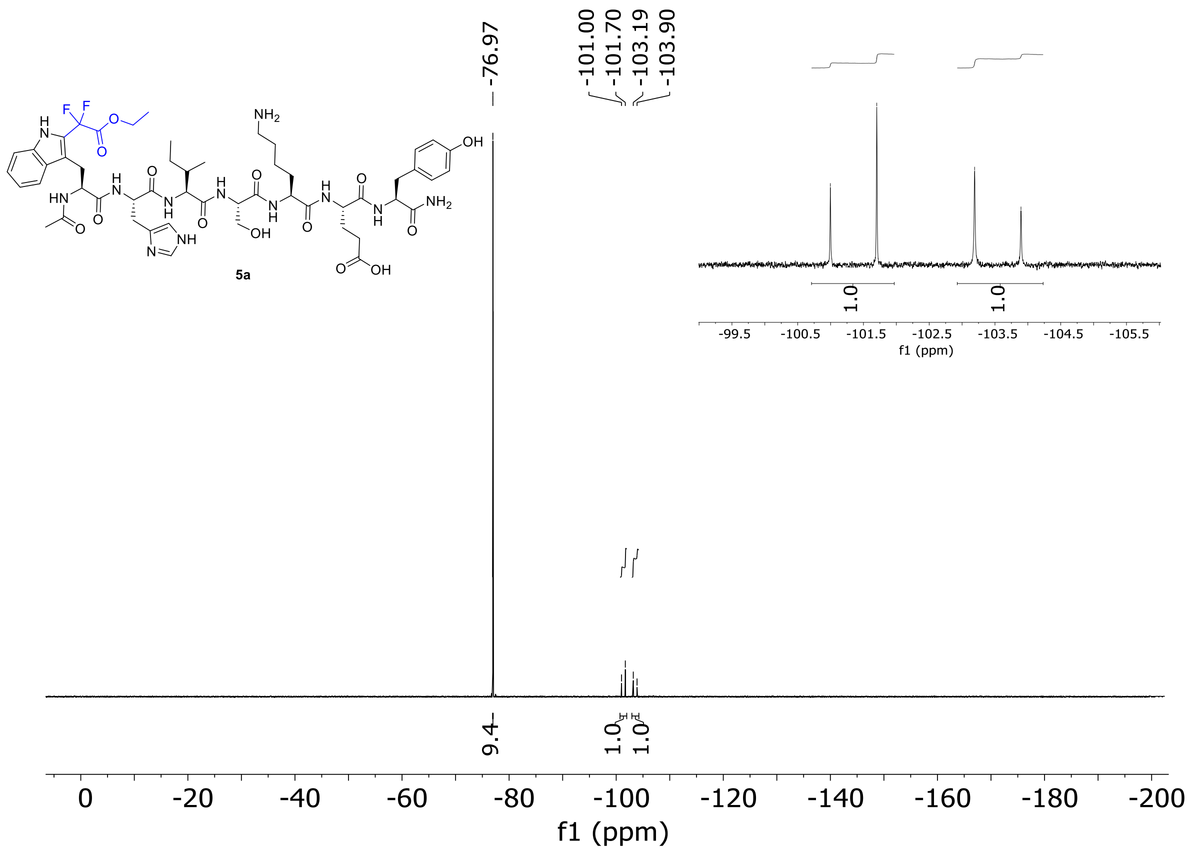

^{1}H NMR spectrum of product **5b** (500 MHz, CD_3OD)

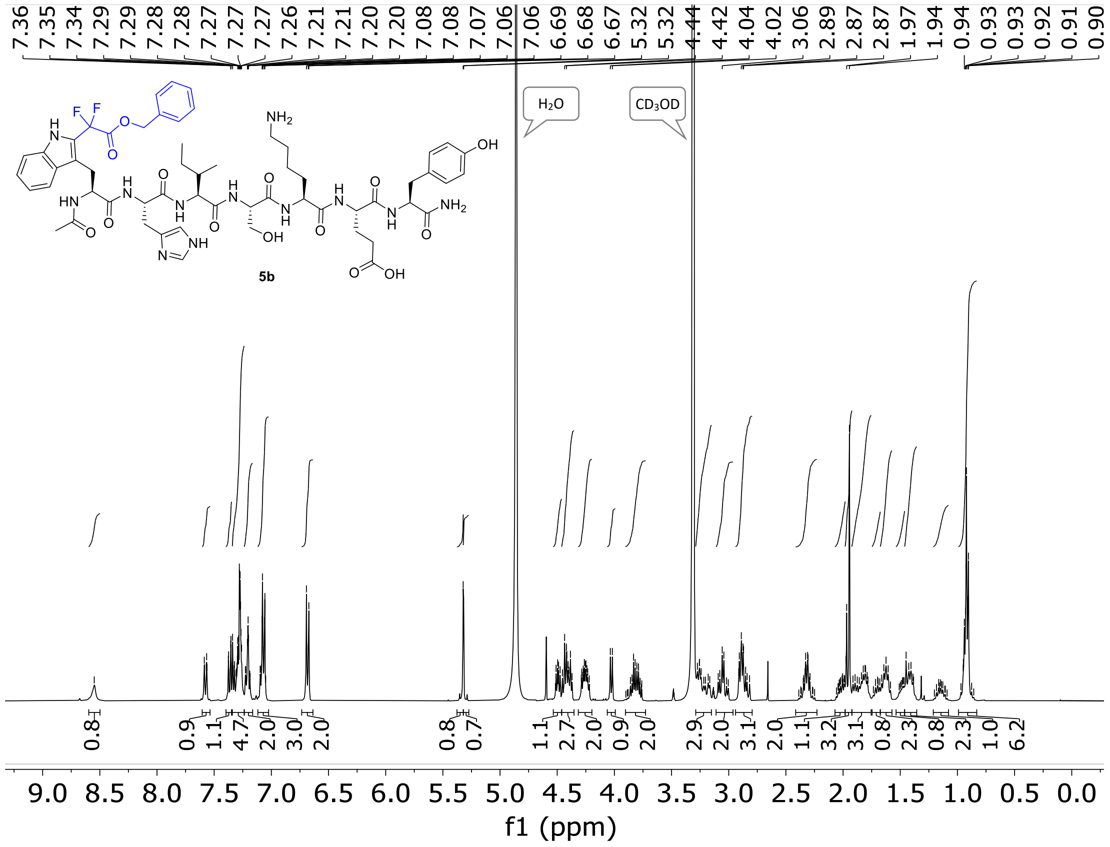

^{19}F NMR spectrum of product **5b** (376 MHz, DMSO-d_6)

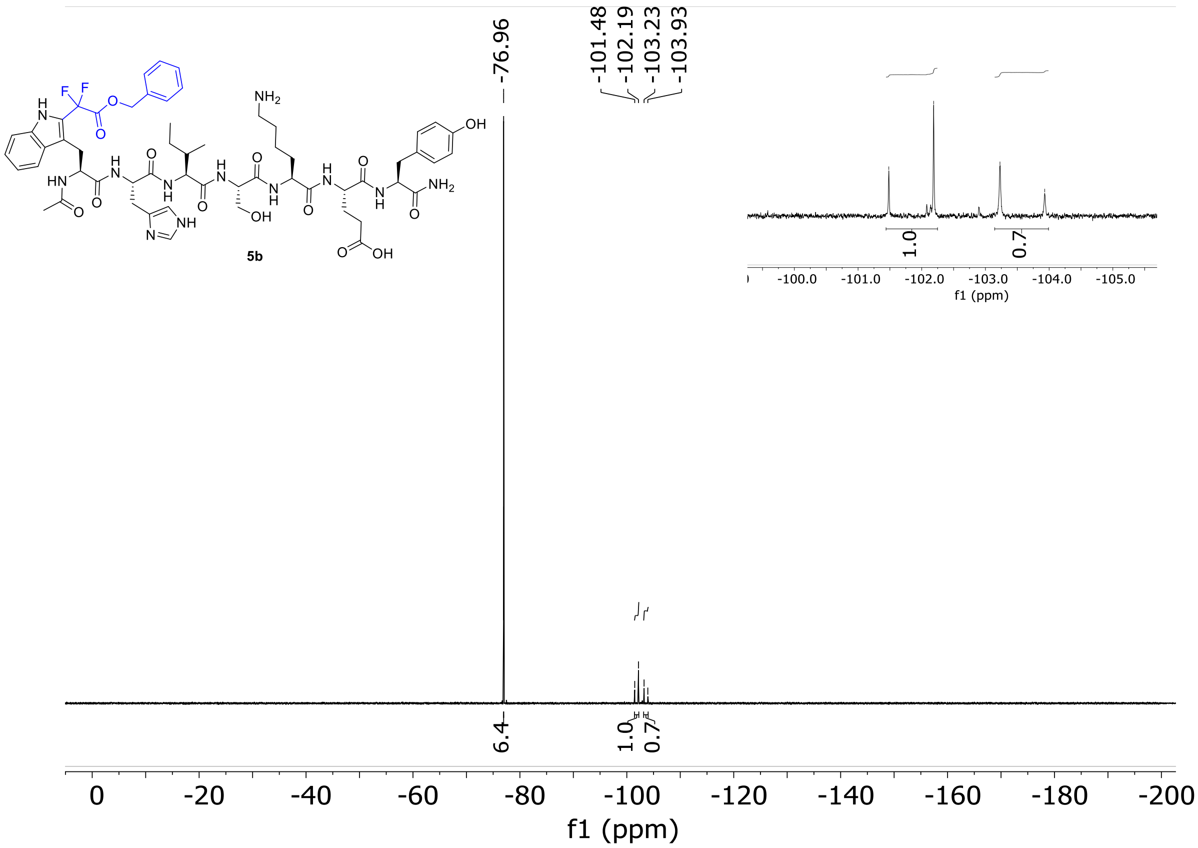

^{1}H NMR spectrum of product **5c** (500 MHz, DMSO-d_6)

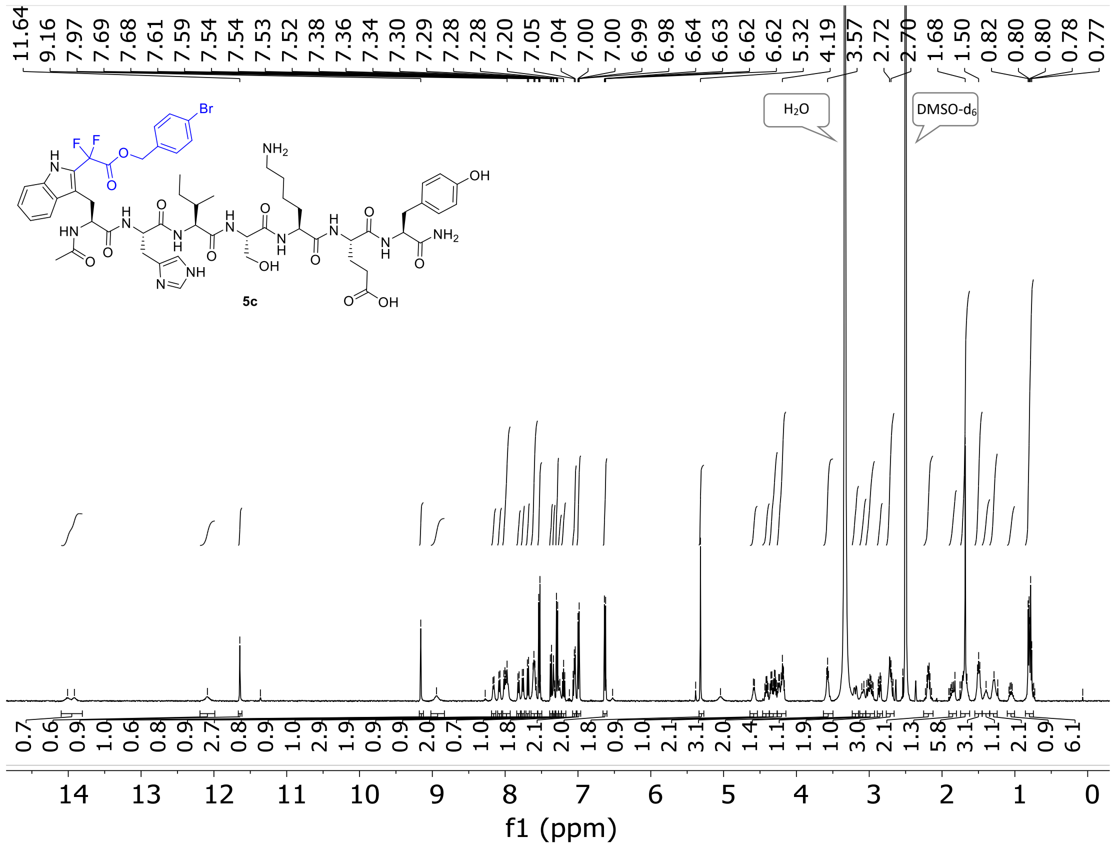

^{19}F NMR spectrum of product **5c** (376 MHz, DMSO-d_6)

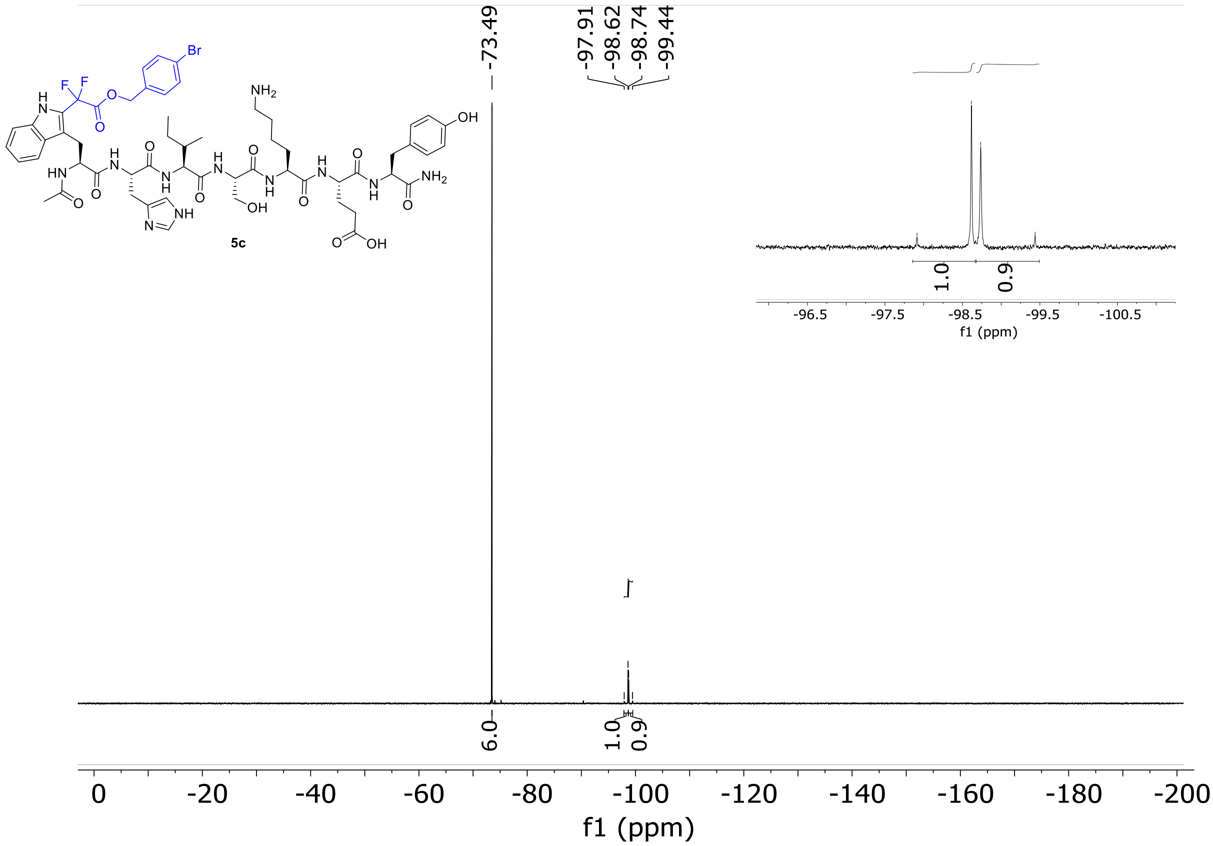

^{1}H NMR spectrum of product **5d** (400 MHz, CD_3OD)

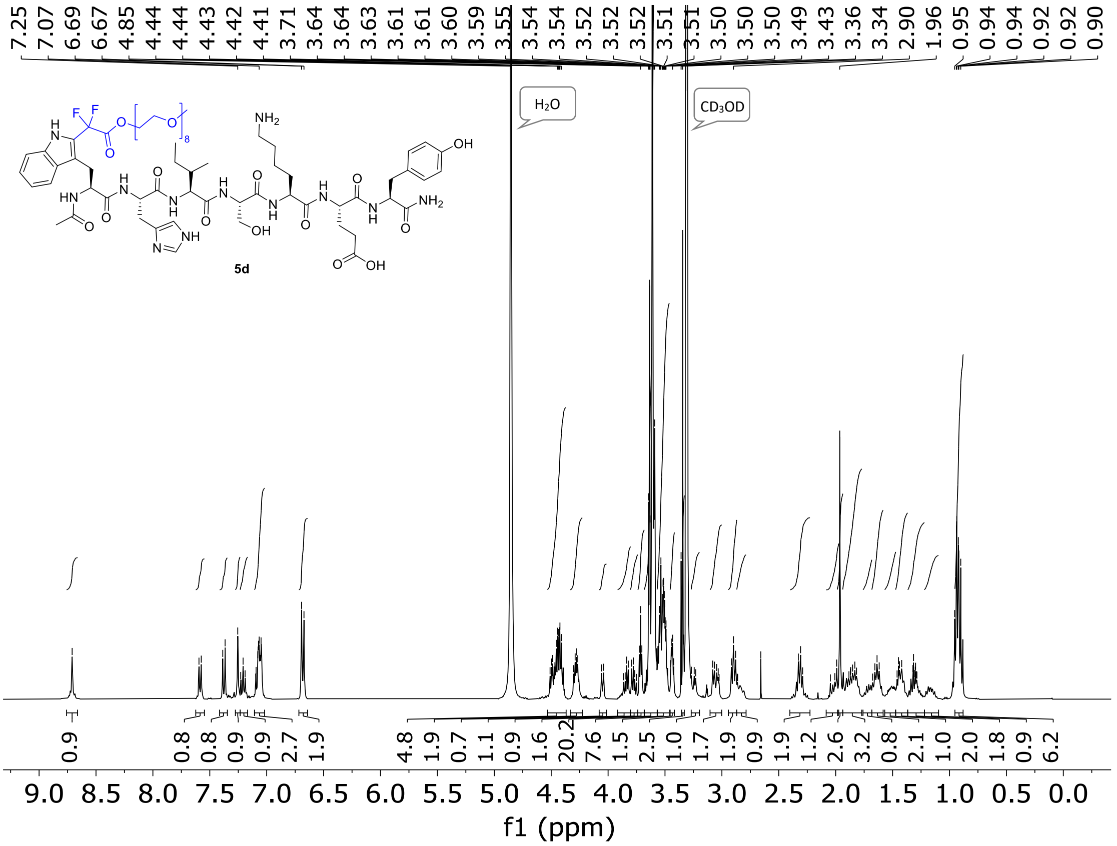

^{19}F NMR spectrum of product **5d** (376 MHz, CD_3OD)

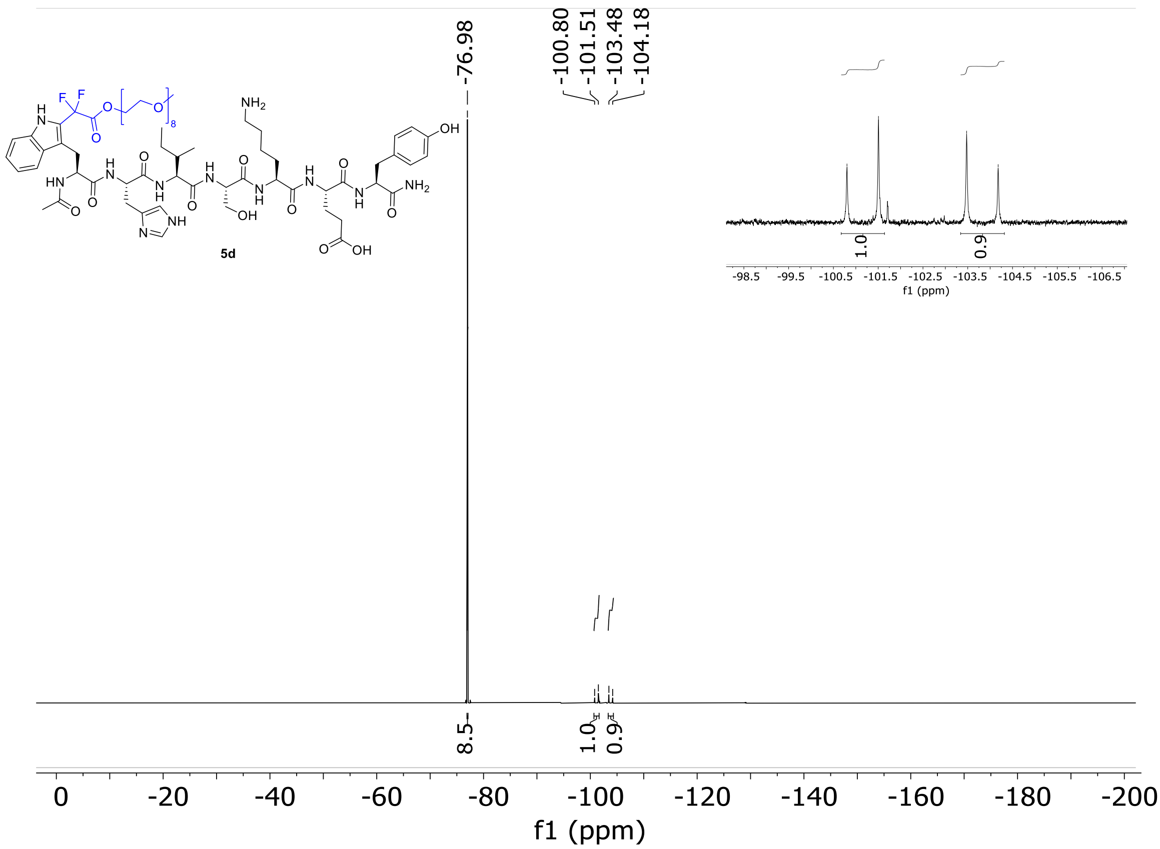

^{1}H NMR spectrum of product **5e** (400 MHz, CD_3OD)

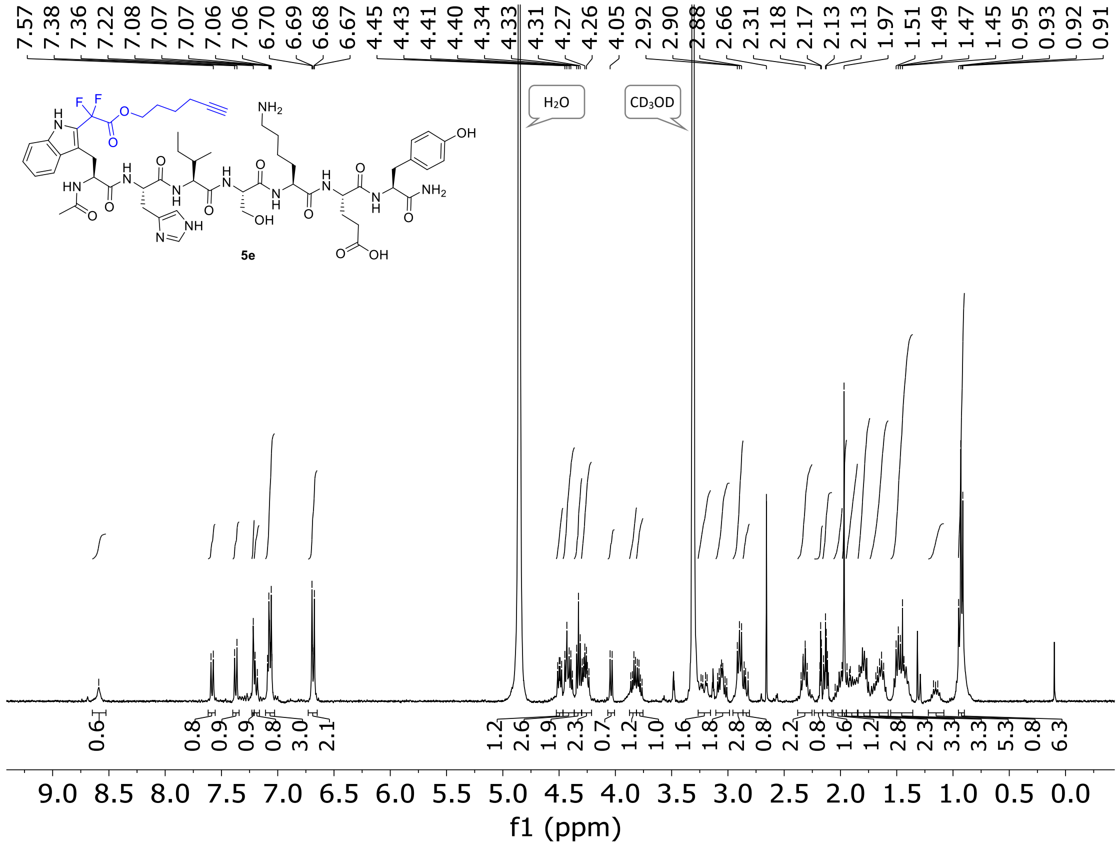

^{19}F NMR spectrum of product **5e** (376 MHz, CD_3OD)

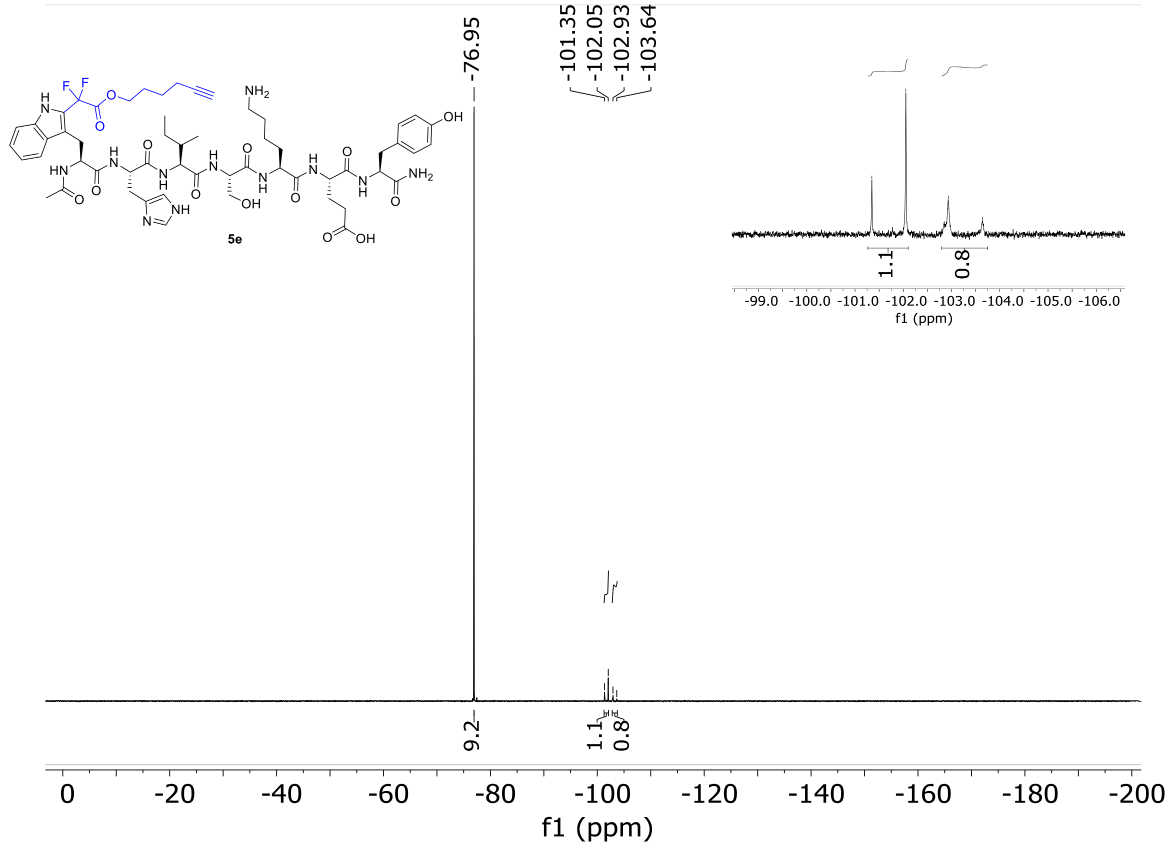

^{1}H NMR spectrum of product **5f** (500 MHz, CD_3OD)

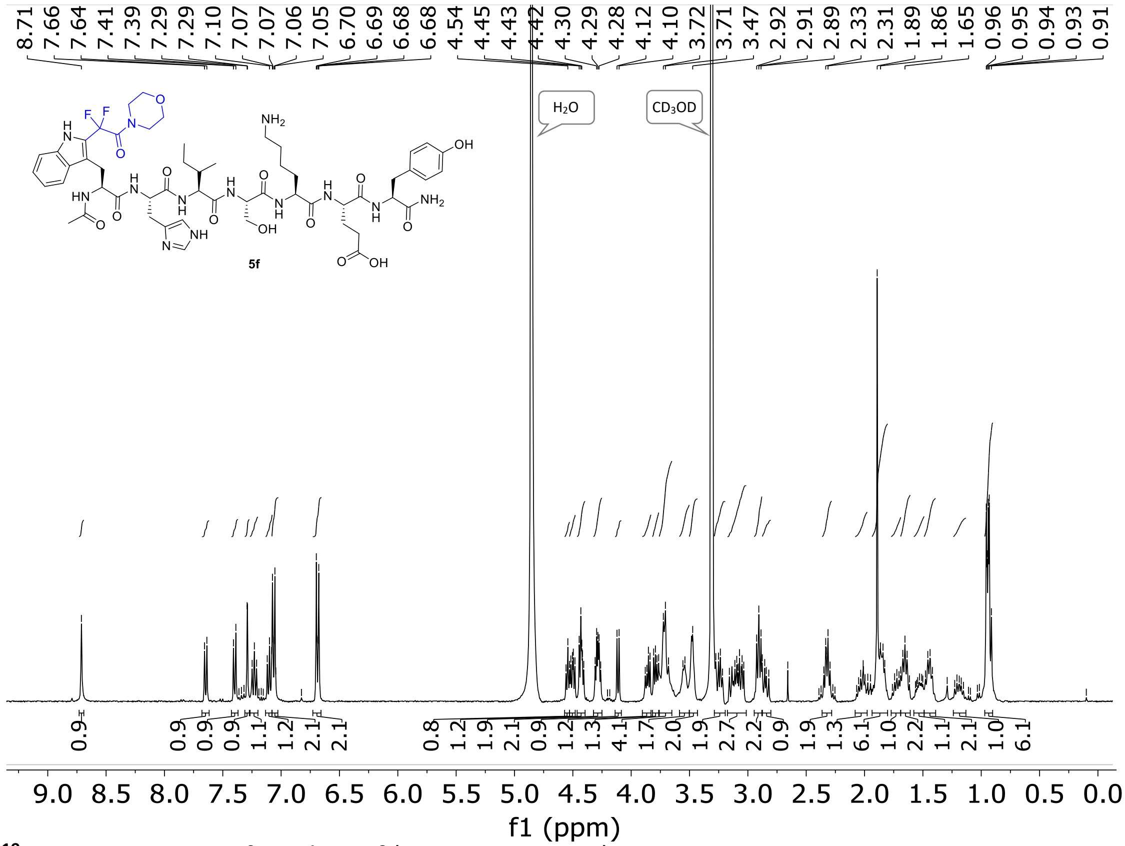

^{19}F NMR spectrum of product **5f** (376 MHz, CD_3OD)

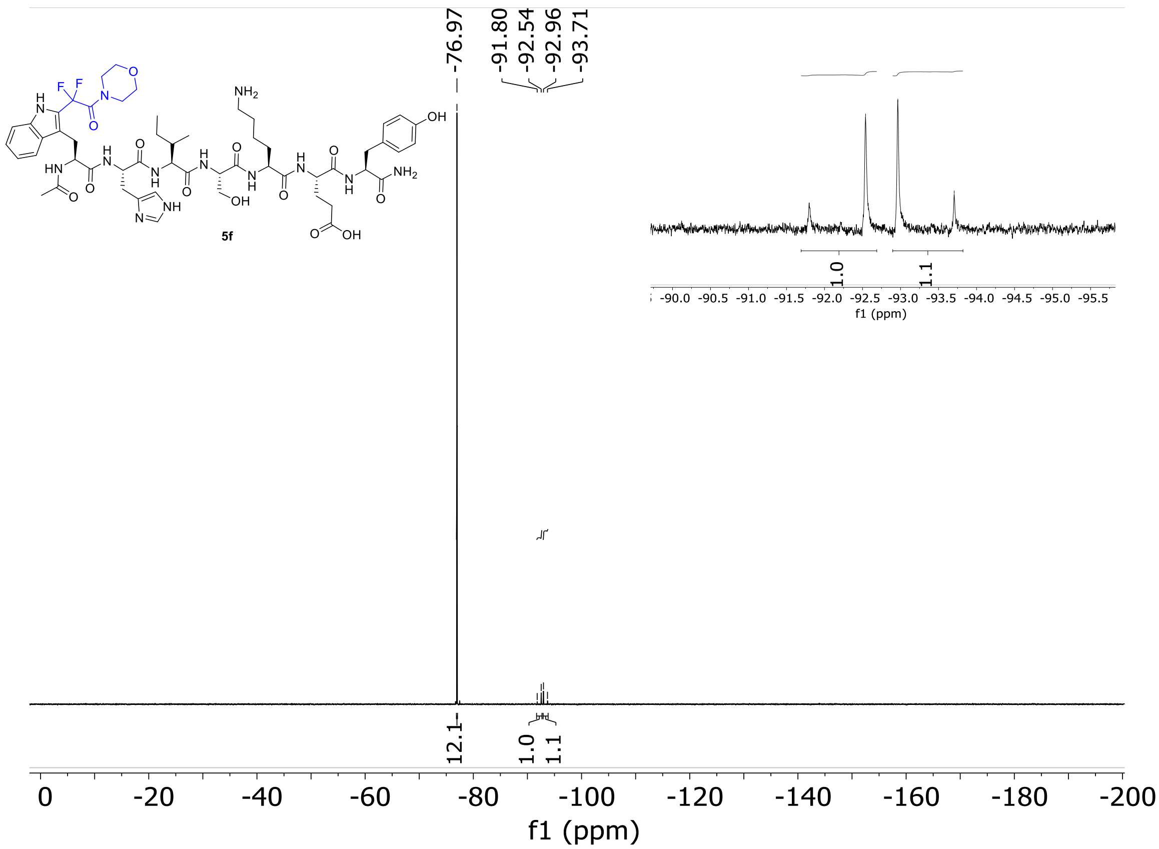

[1]**H NMR** spectrum of product **5g** (500 MHz, CD_3OD)

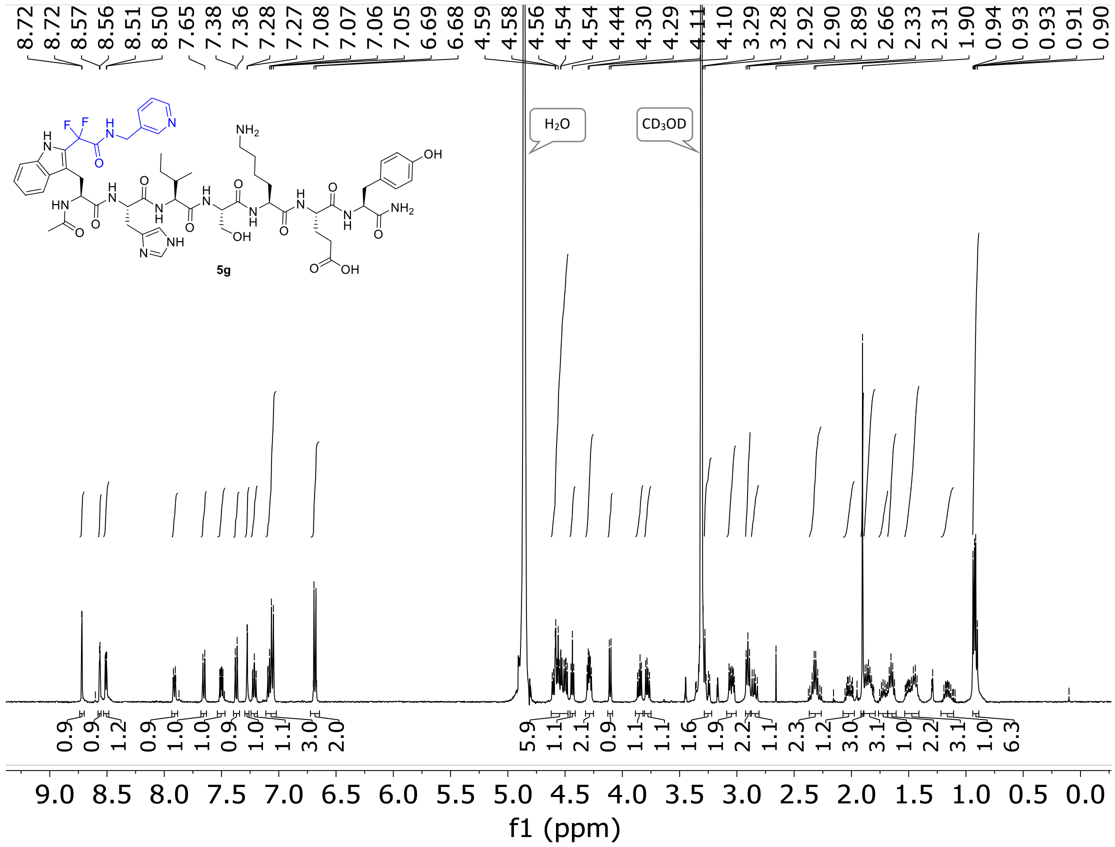

[19]**F NMR** spectrum of product **5g** (376 MHz, DMSO-d_6)

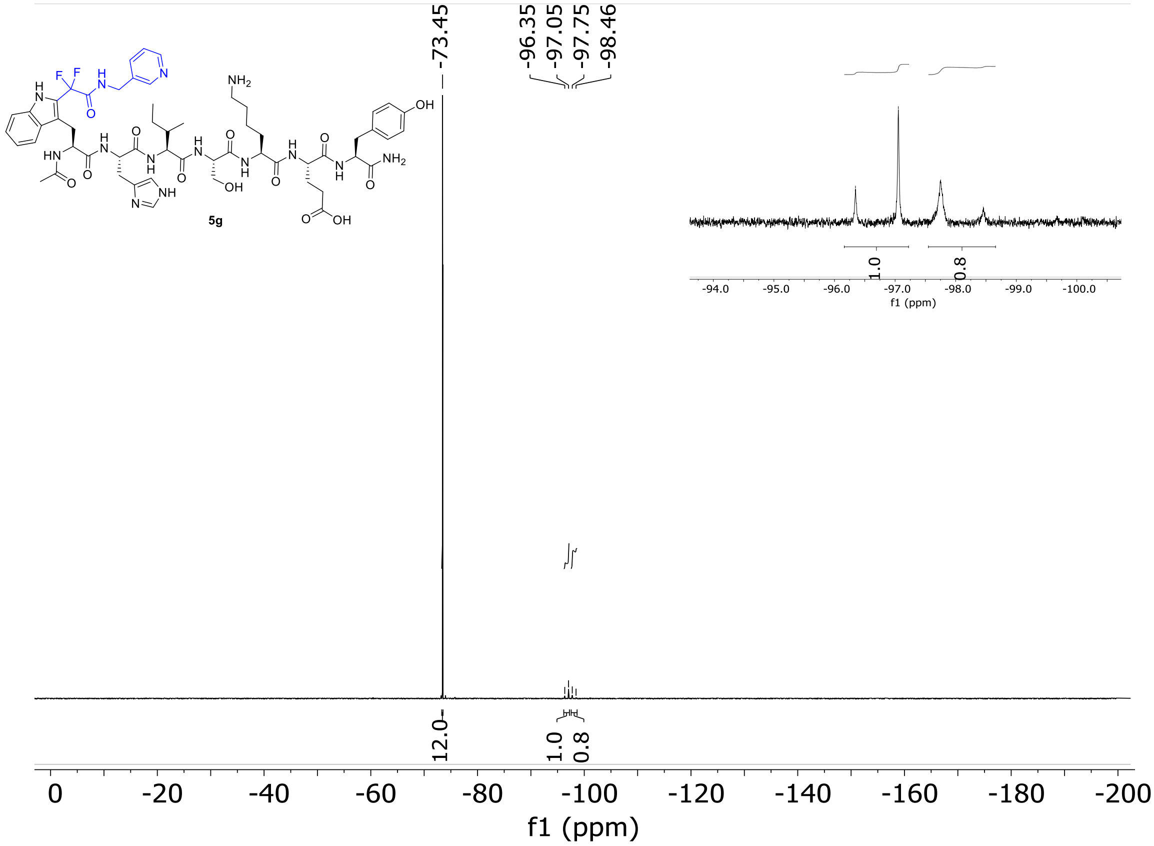

^{1}H NMR spectrum of product **5h** (500 MHz, CD_3OD)

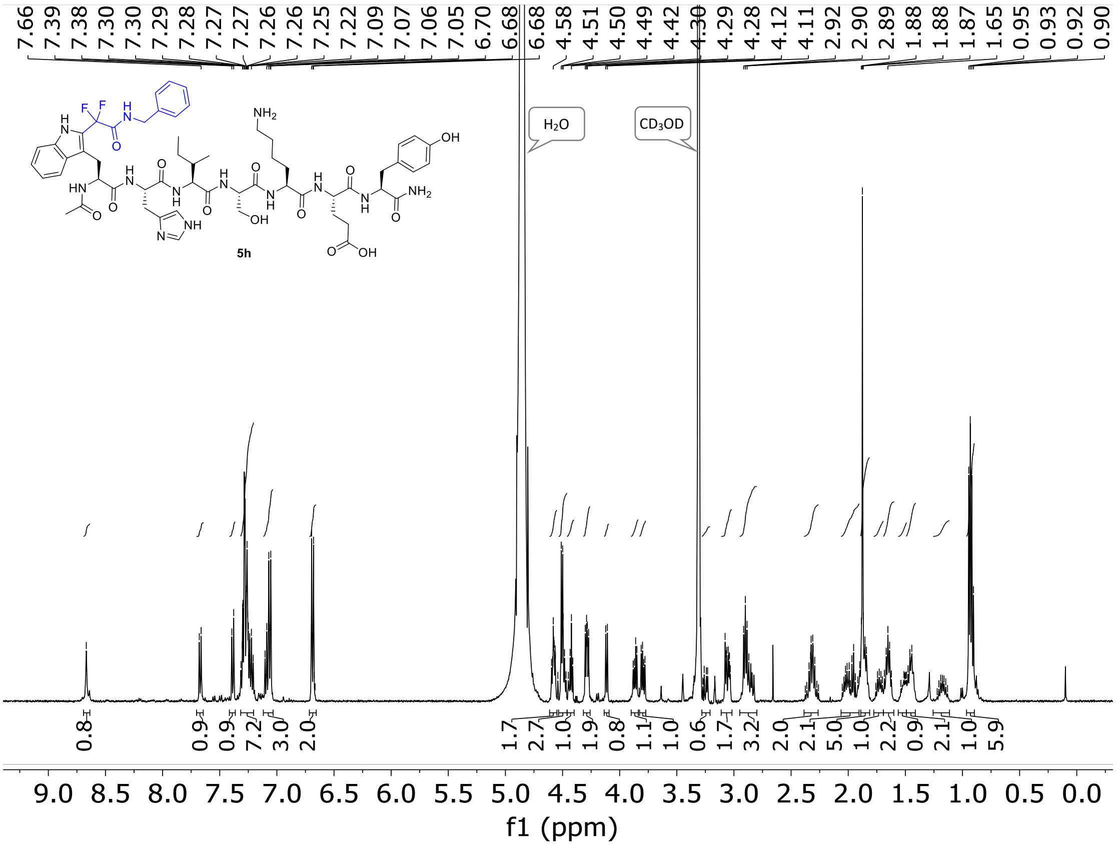

^{19}F NMR spectrum of product **5h** (376 MHz, DMSO-d_6)

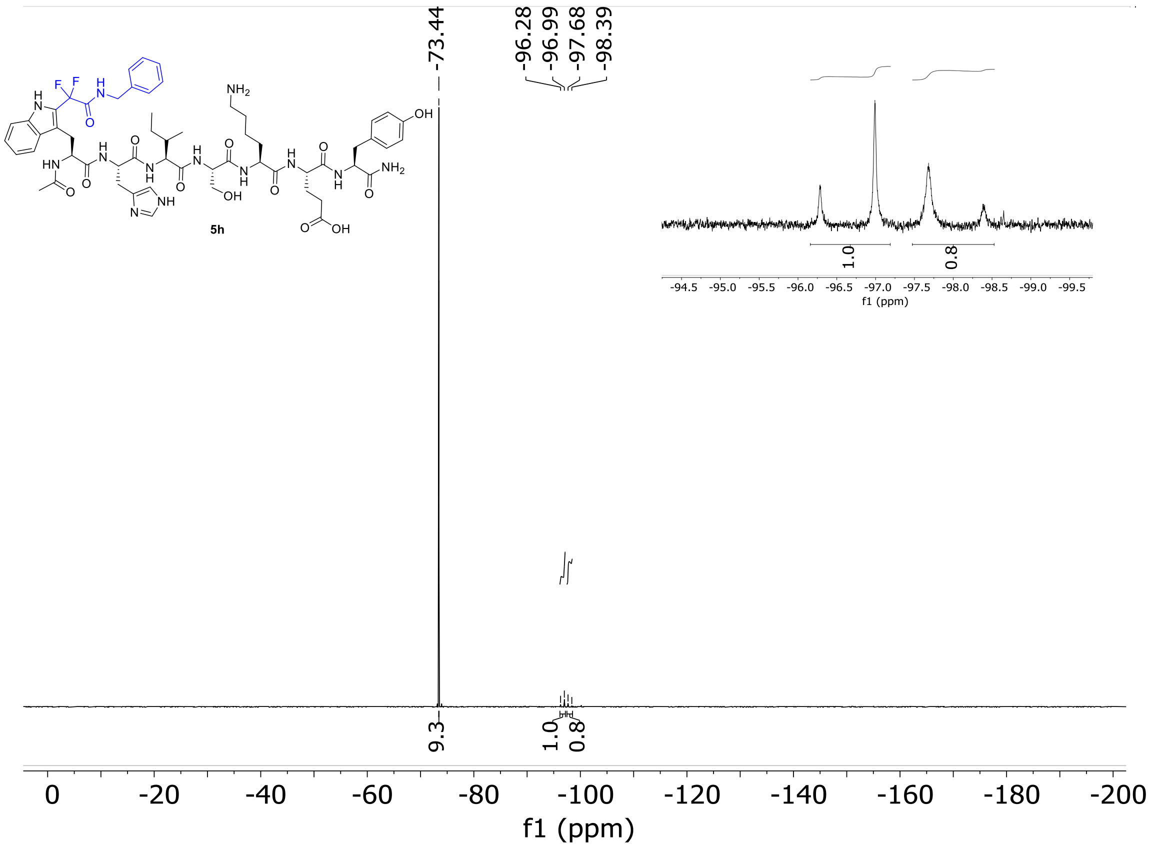

^{1}**H NMR** spectrum of product **5i** (500 MHz, CD_3OD)

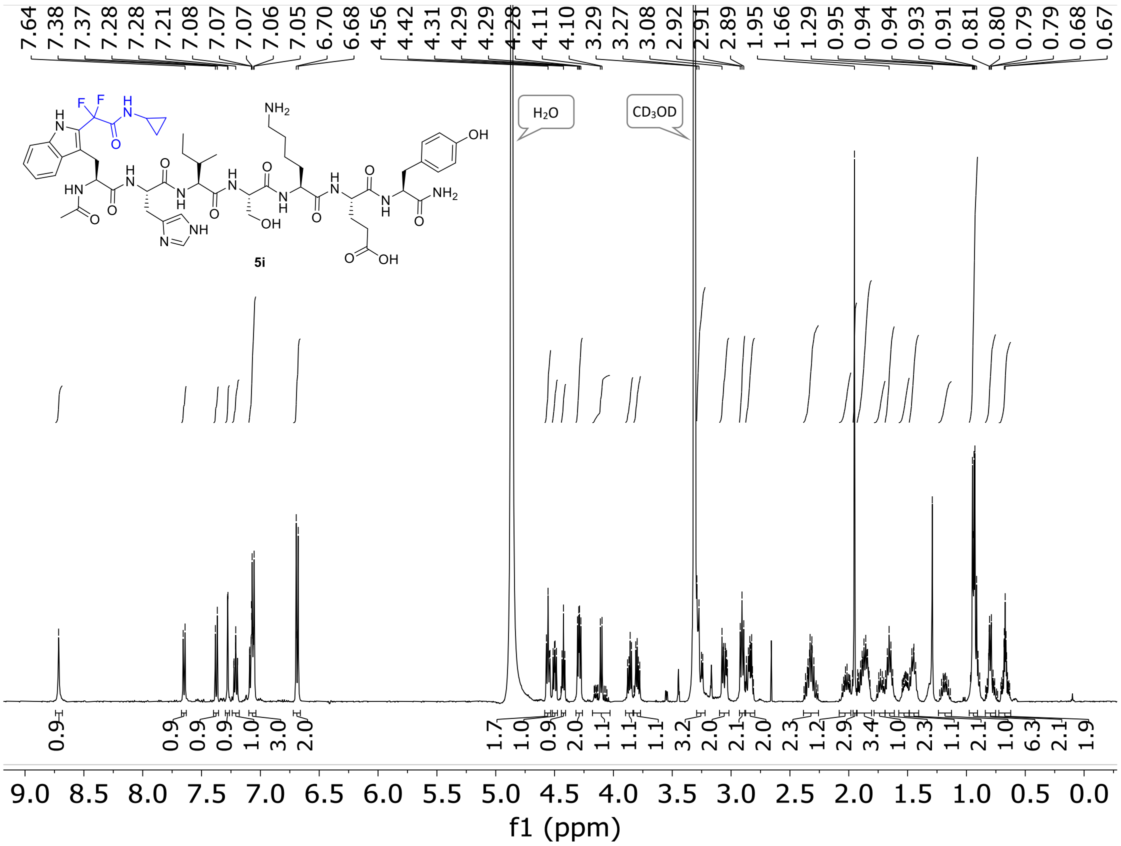

^{19}**F NMR** spectrum of product **5i** (376 MHz, DMSO-d_6)

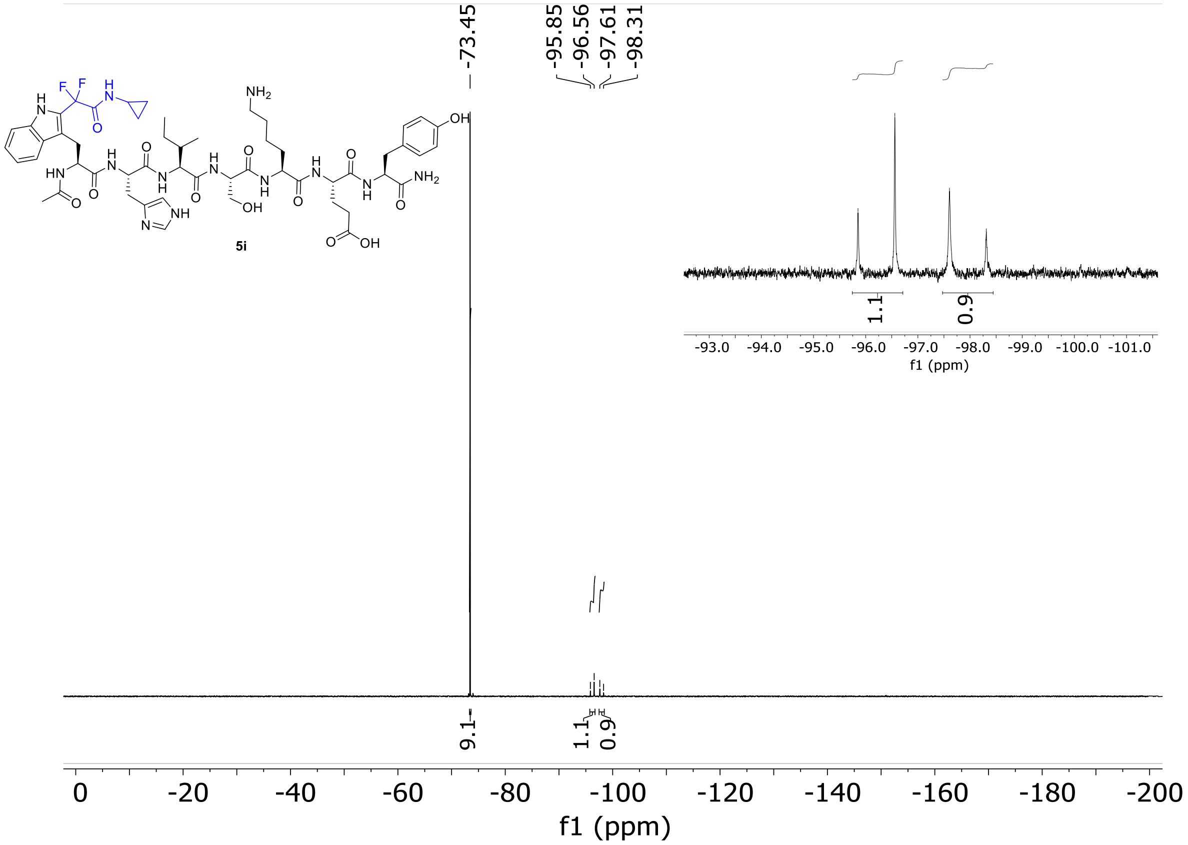

^{1}H NMR spectrum of product **5j** (500 MHz, CD_3OD)

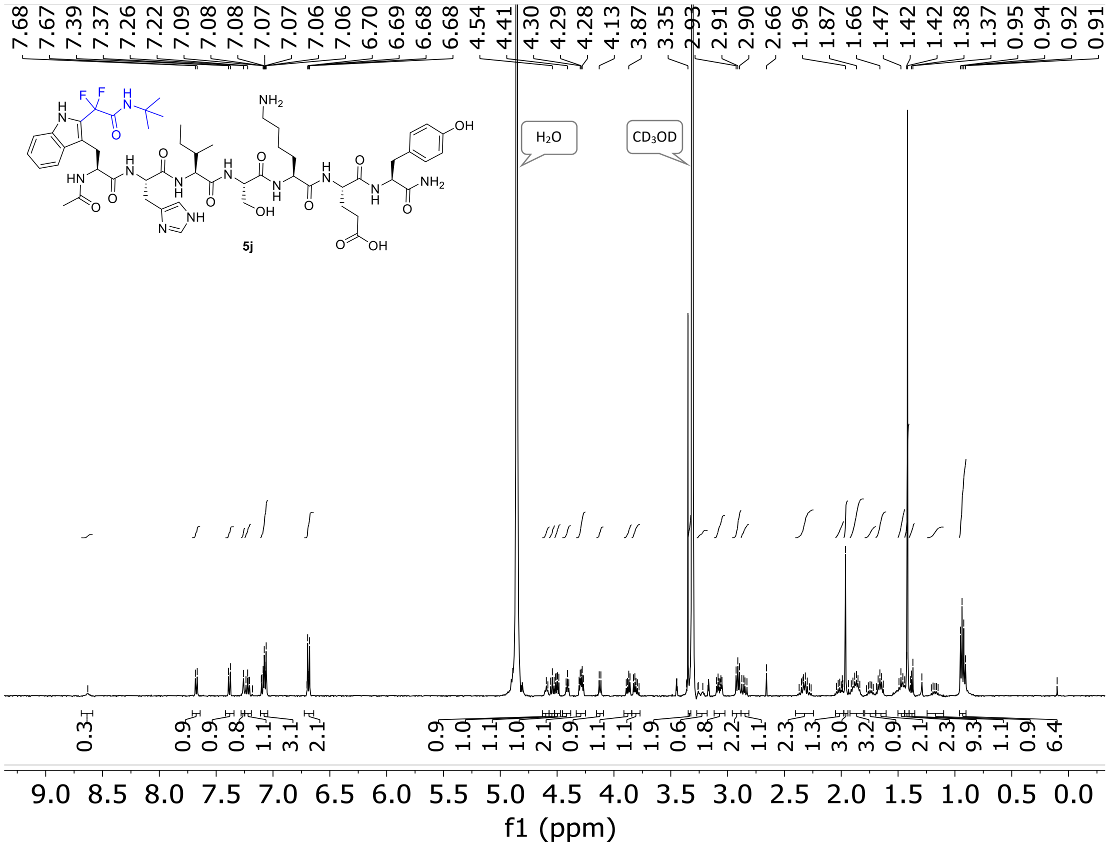

^{19}F NMR spectrum of product **5j** (376 MHz, CD_3OD)

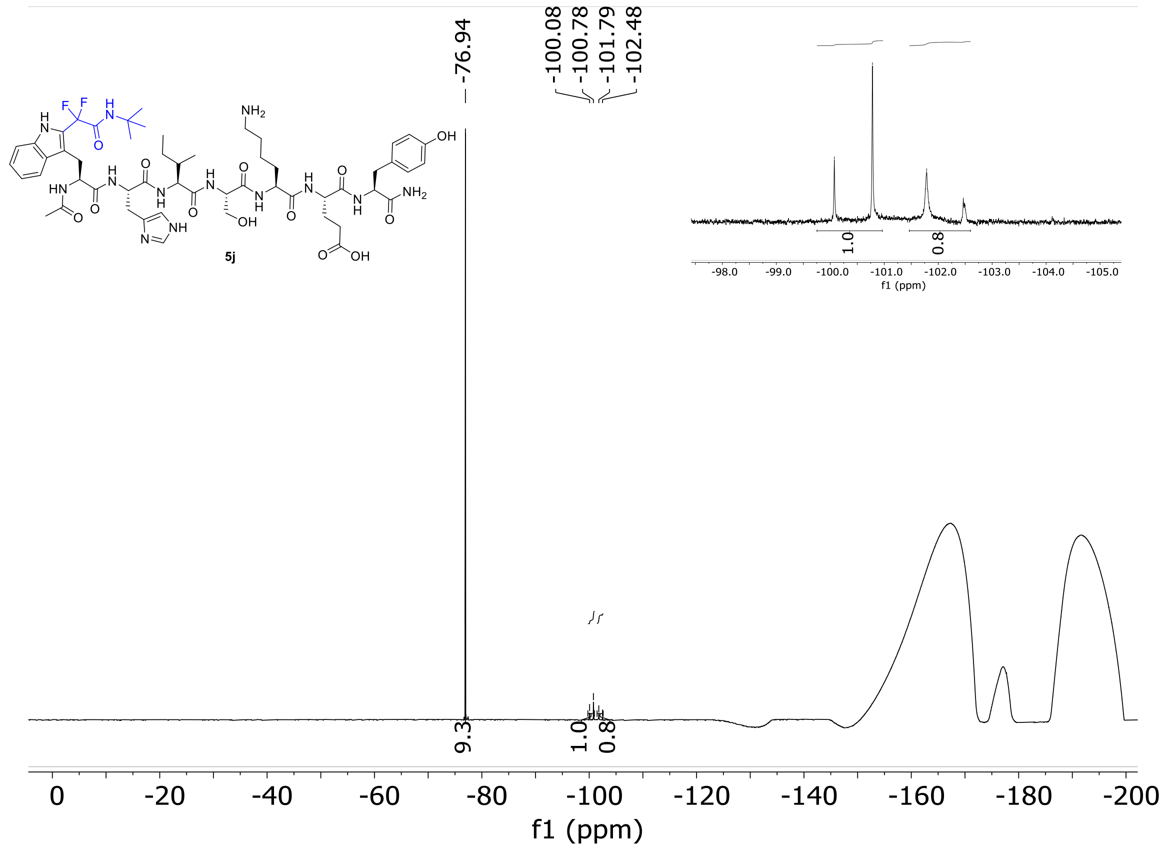

^{1}H NMR spectrum of product **5k** (500 MHz, CD_3OD)

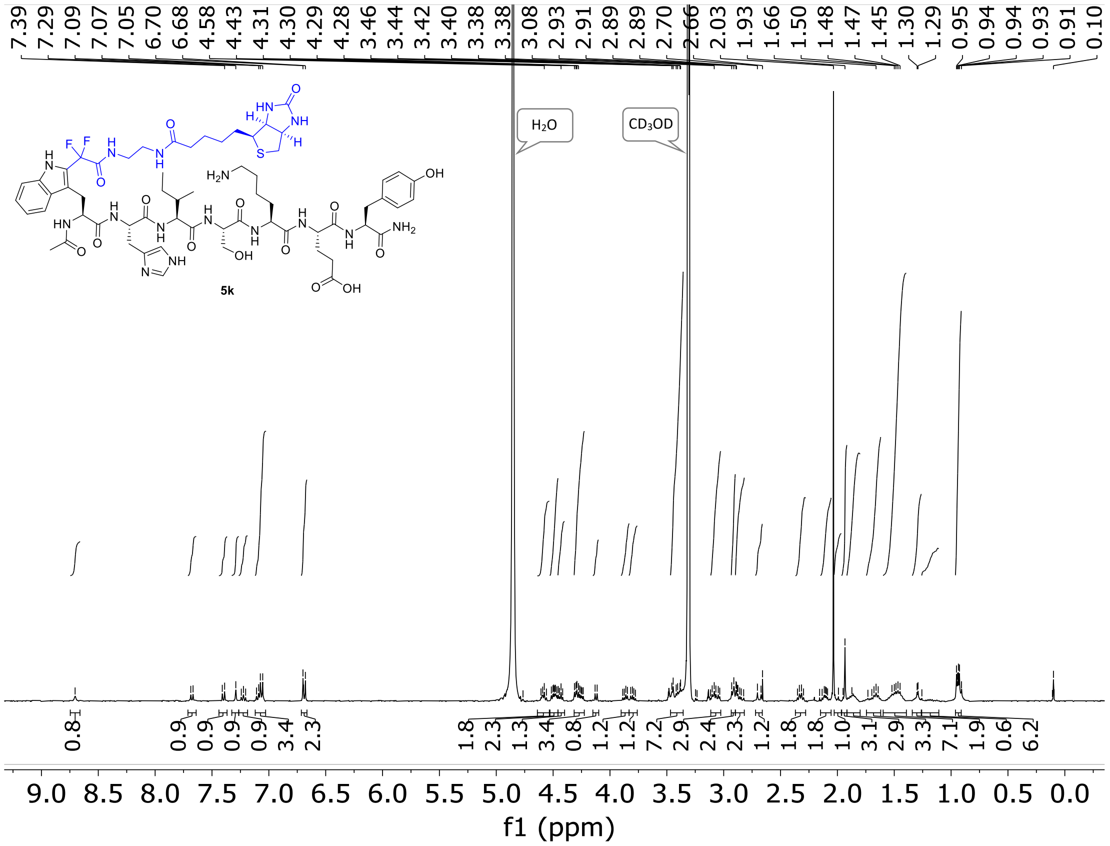

^{19}F NMR spectrum of product **5k** (376 MHz, CD_3OD)

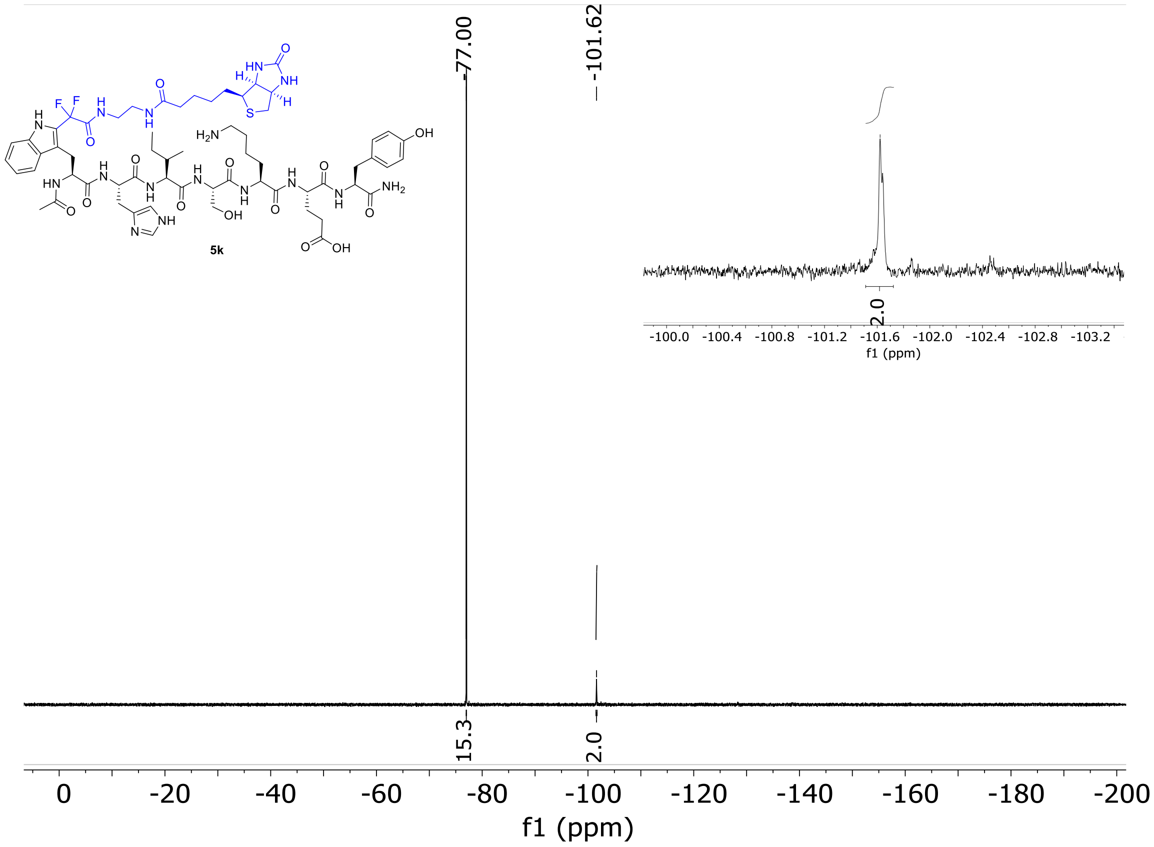

^{1}**H NMR** spectrum of product **5l** (500 MHz, CD_3OD)

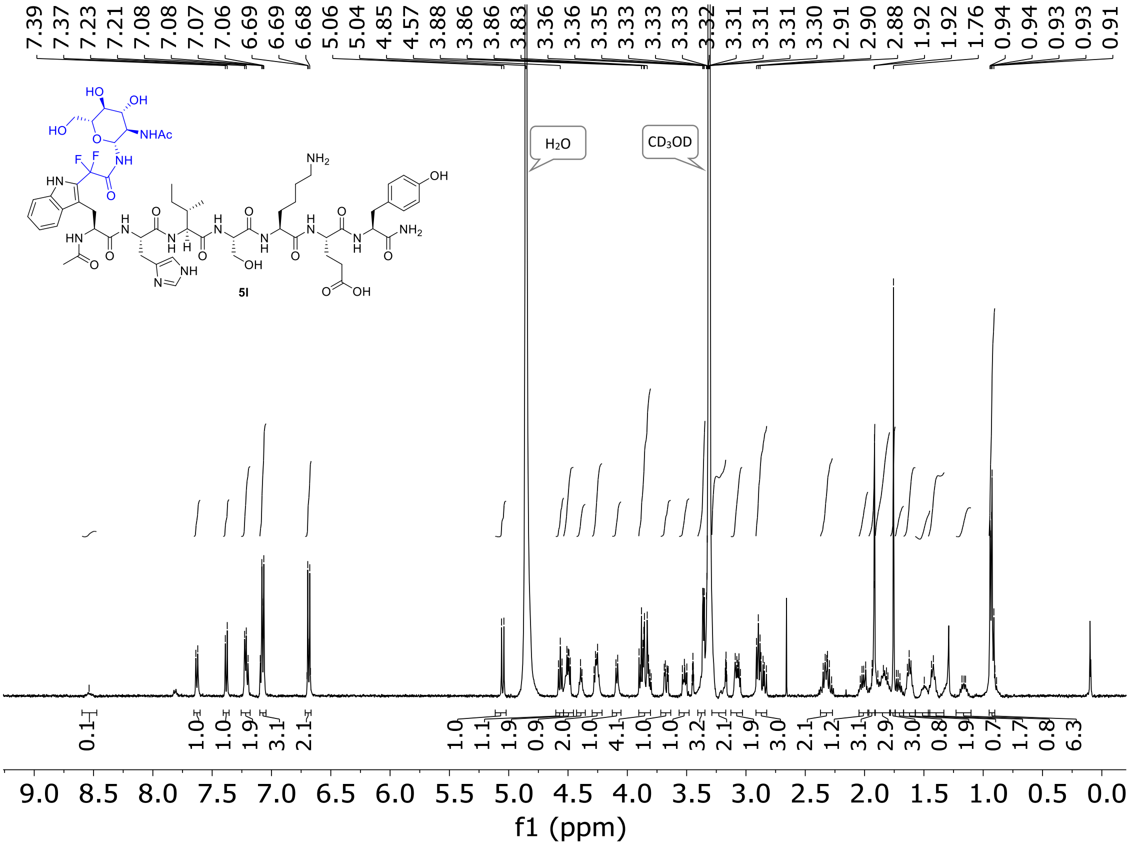

^{19}**F NMR** spectrum of product **5l** (376 MHz, CD_3OD)

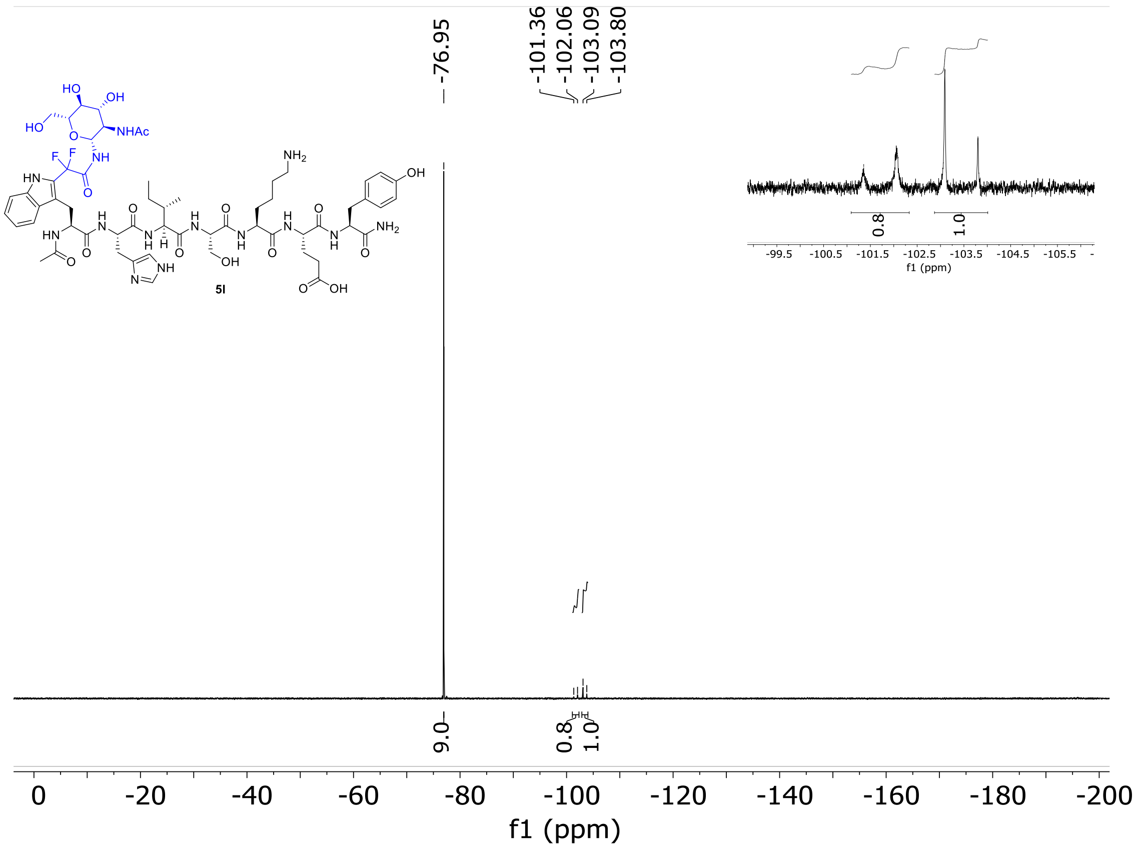

^{19}F NMR spectrum of the crude reaction mixture for peptide **6** using α,α,α TFT as the internal standard (376 MHz, DMSO)

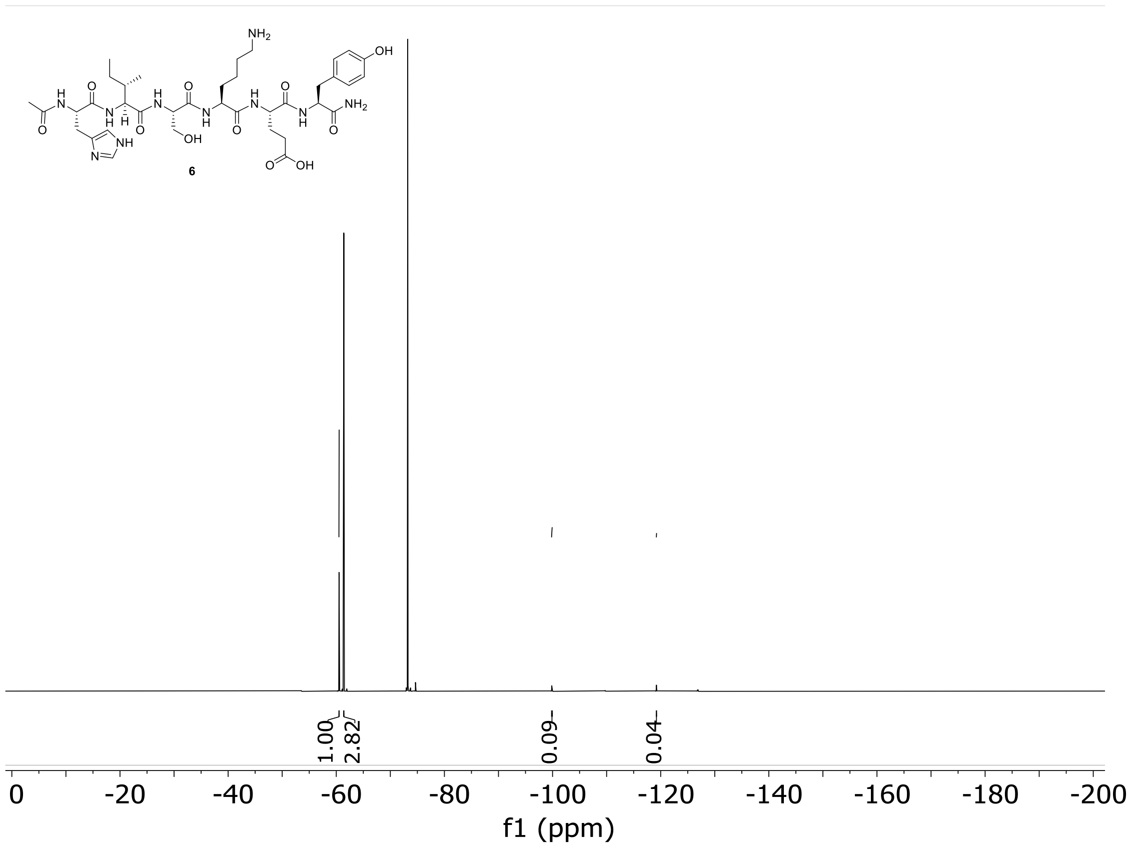

^{1}H NMR spectrum of product **13** (500 MHz, CD_3OD)

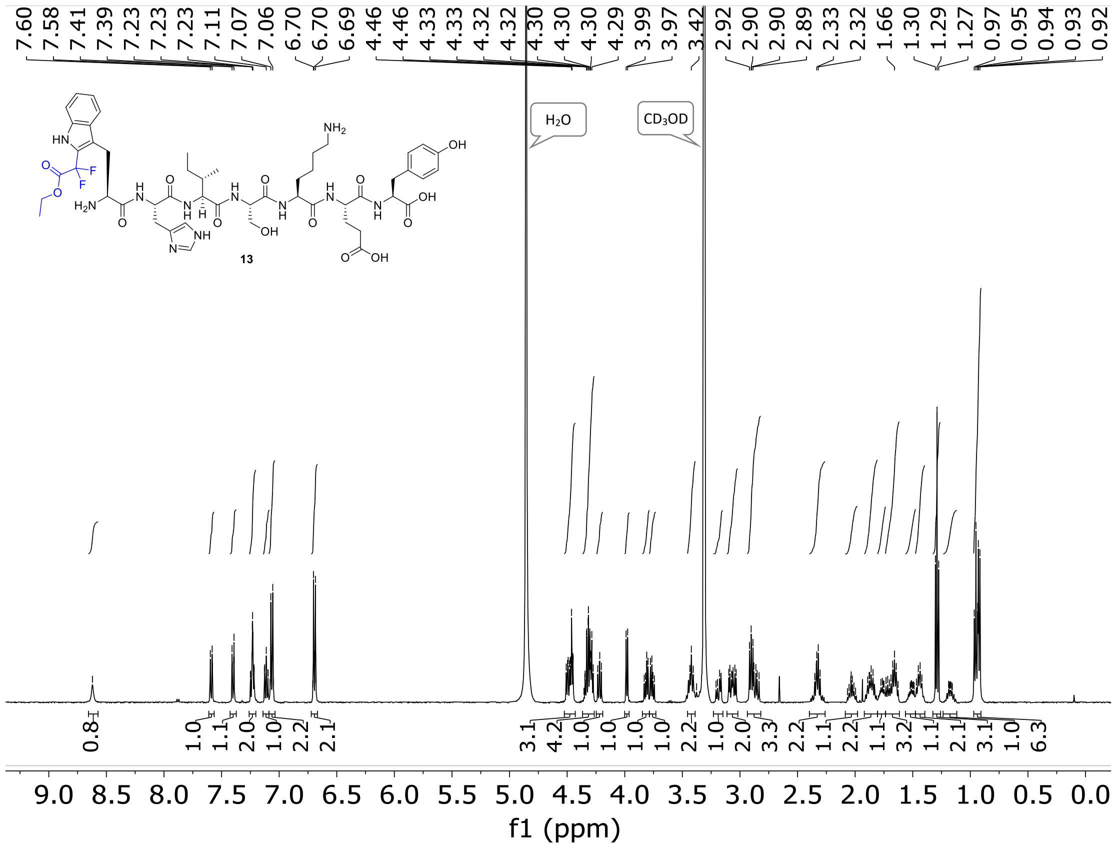

^{19}F NMR spectrum of product **13** (376 MHz, CD_3OD)

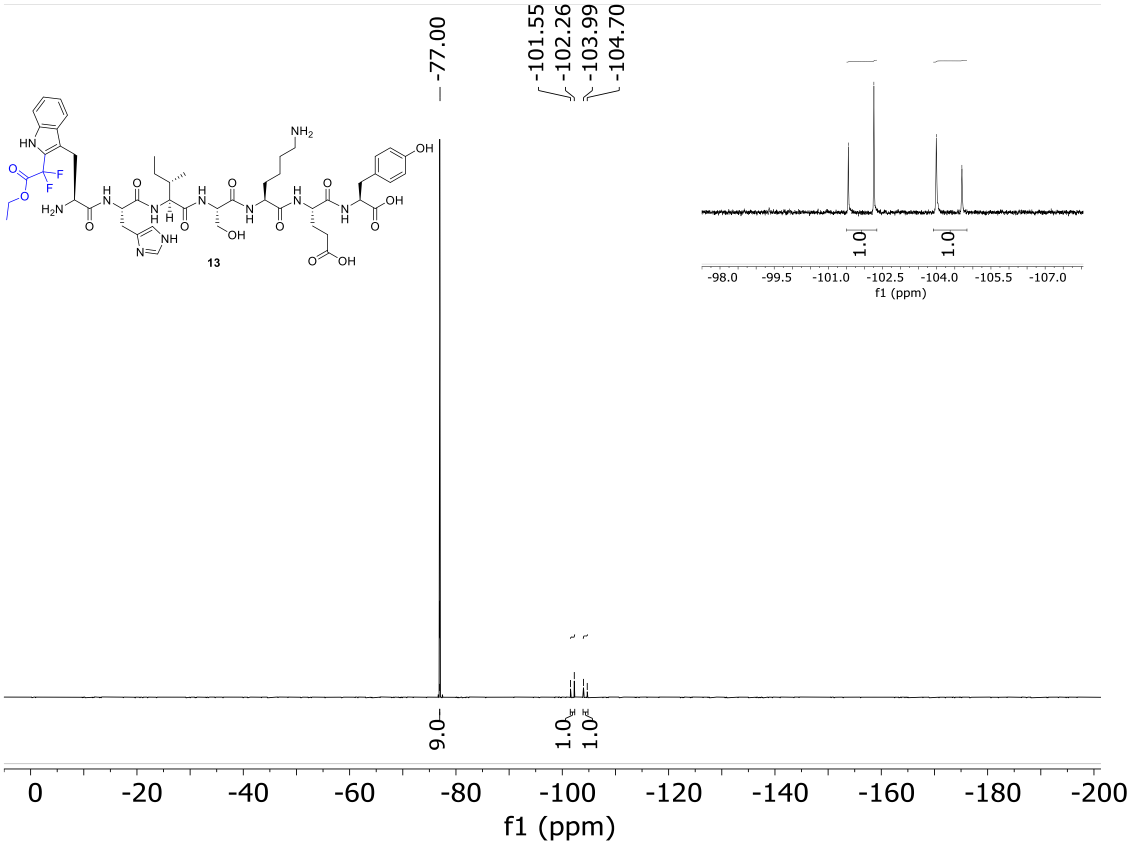

^{1}H NMR spectrum of product **14** (500 MHz, CD_3OD)

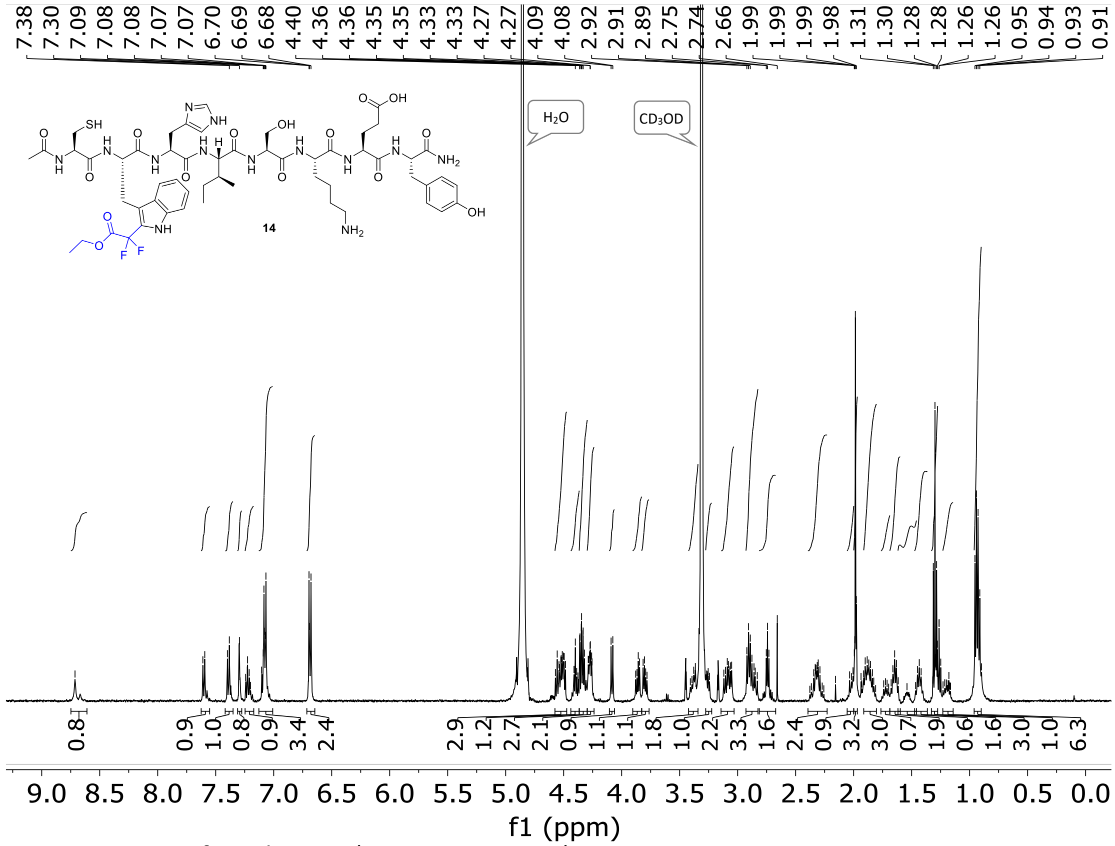

^{19}F NMR spectrum of product **14** (376 MHz, CD_3OD)

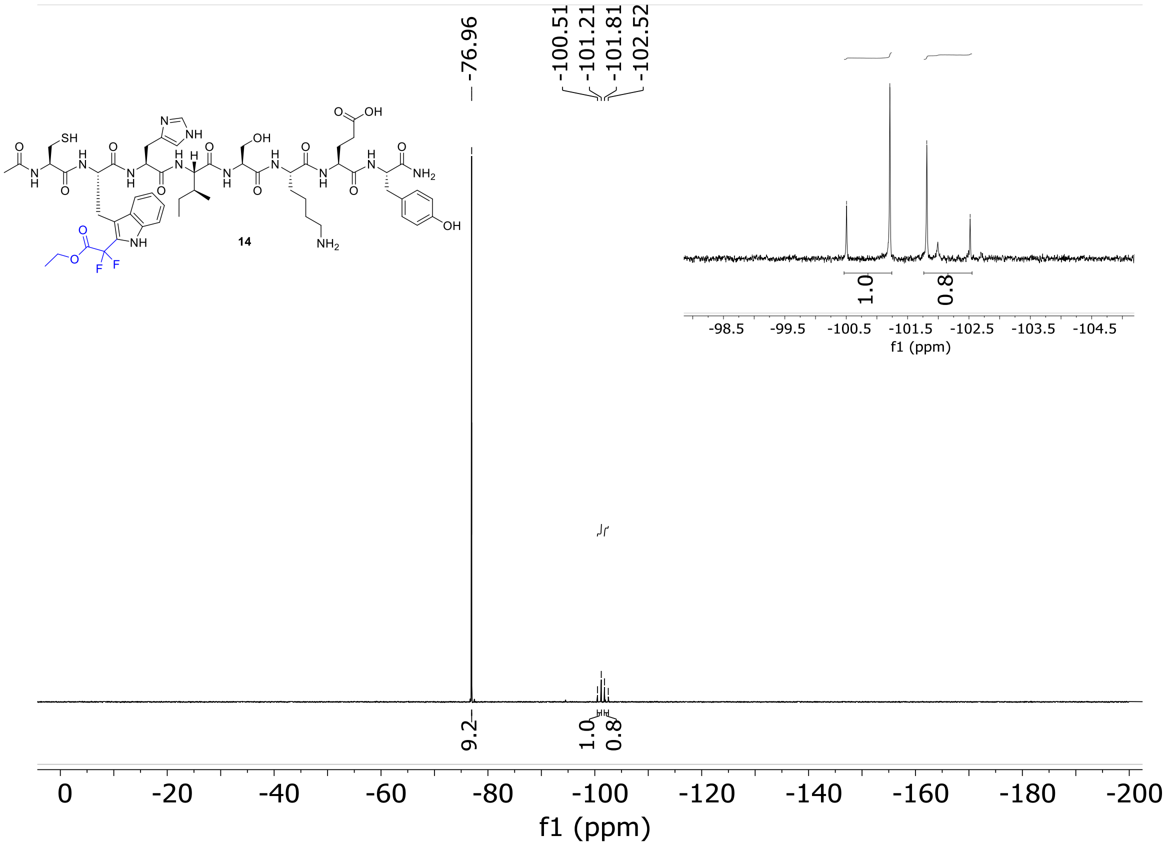

[1]**H NMR** spectrum of product **15** (400 MHz, CD_3OD)

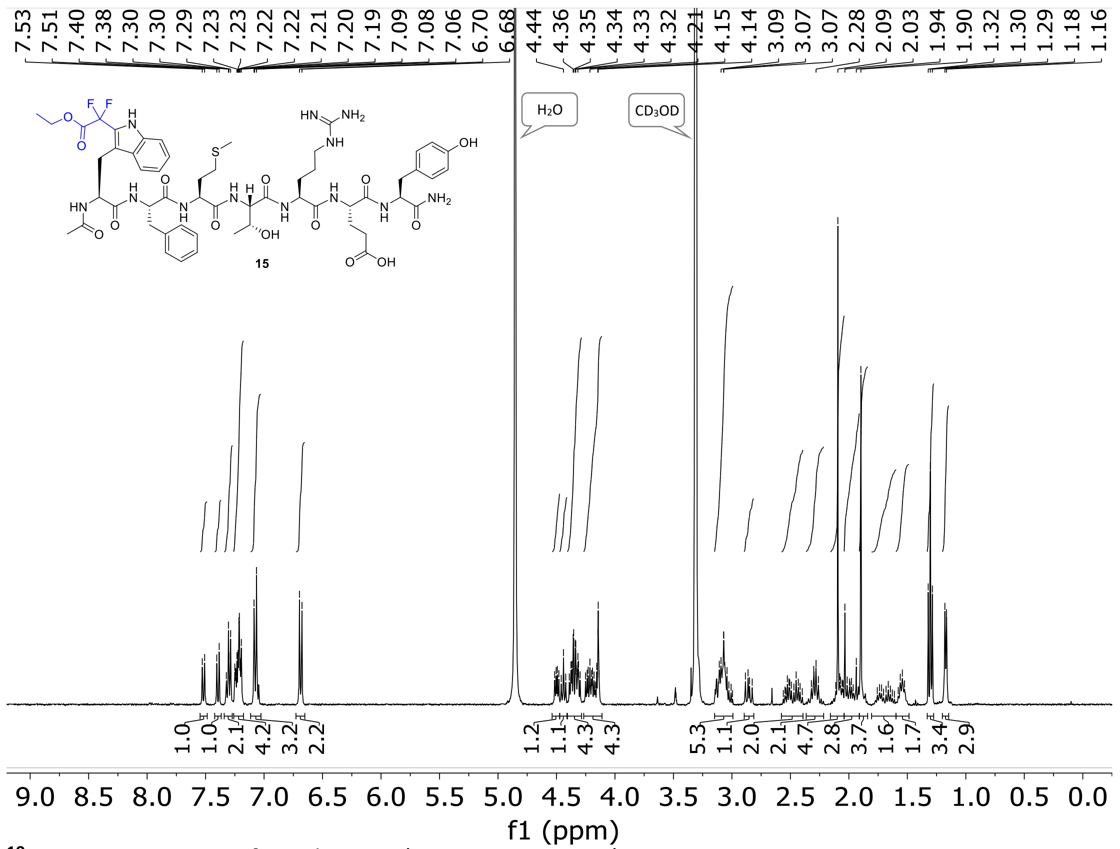

[19]**F NMR** spectrum of product **15** (400 MHz, CD_3OD)

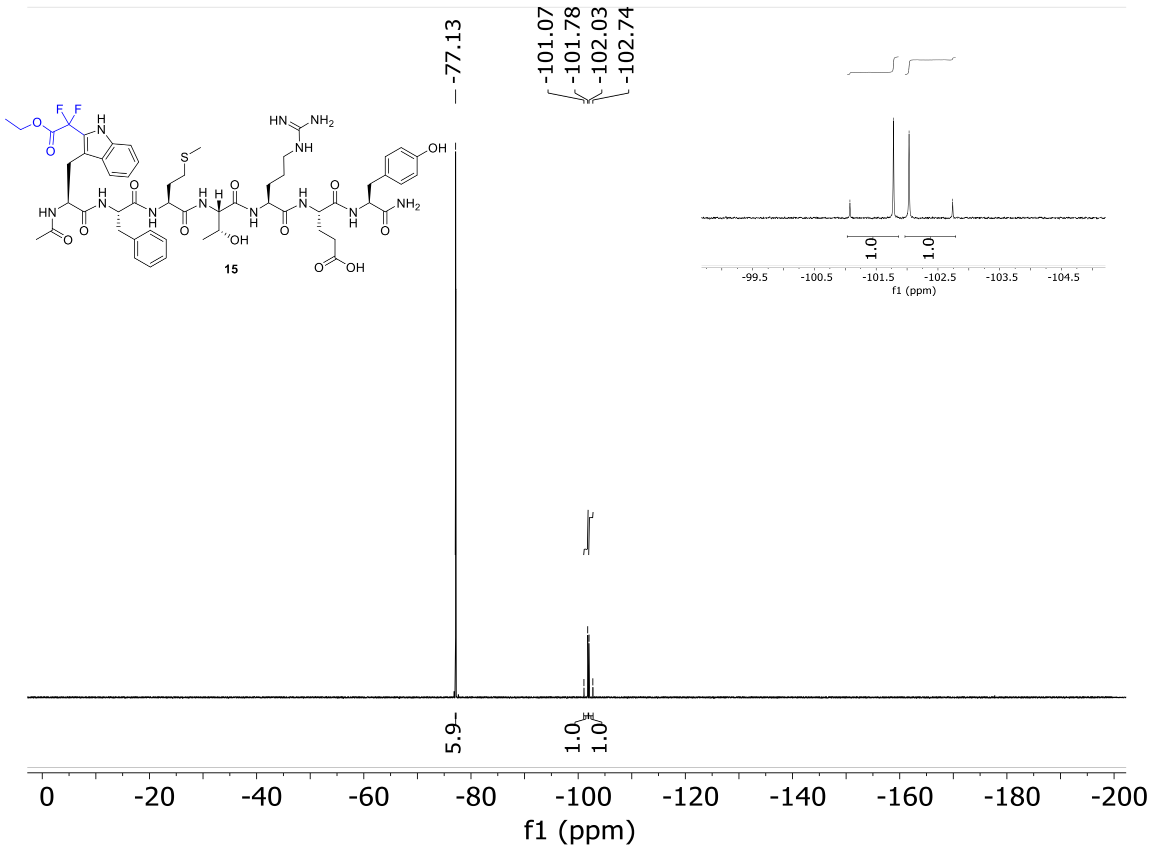

^{1}H NMR spectrum of product **16** (500 MHz, CD_3OD)

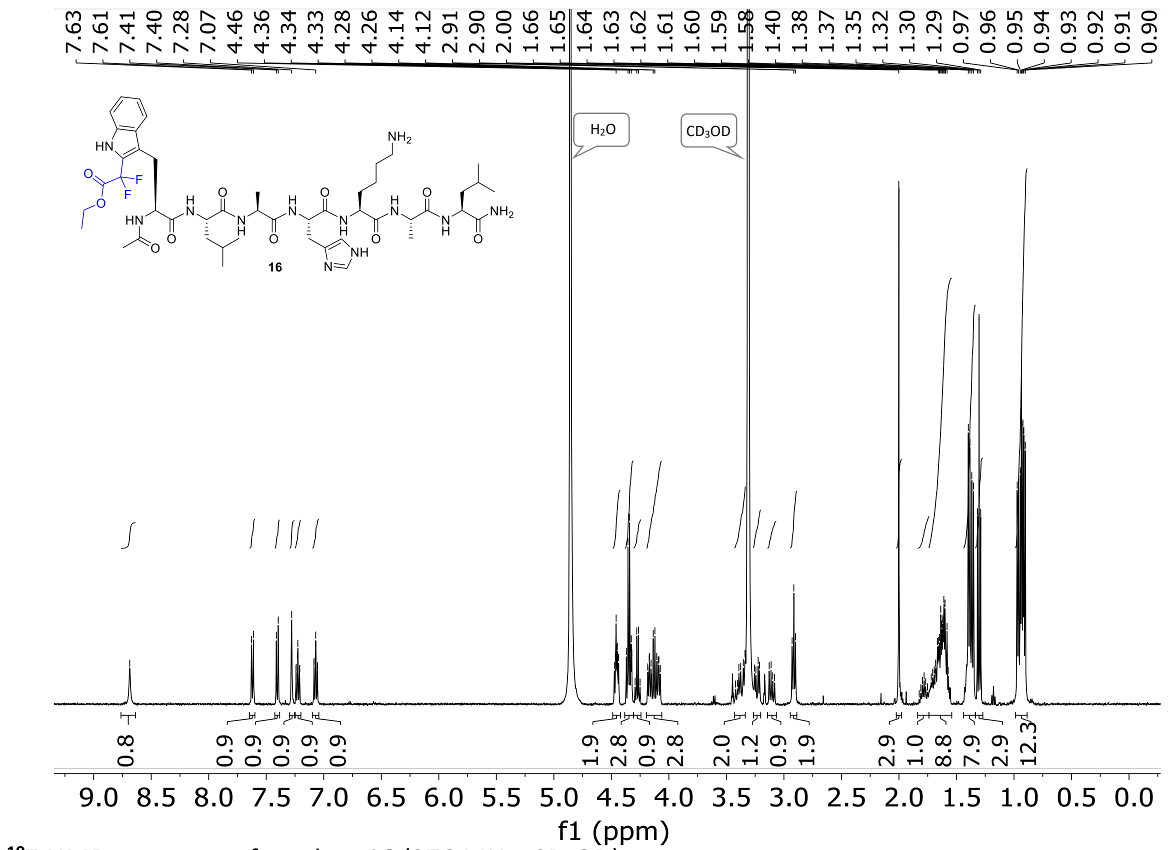

^{19}F NMR spectrum of product **16** (376 MHz, CD_3OD)

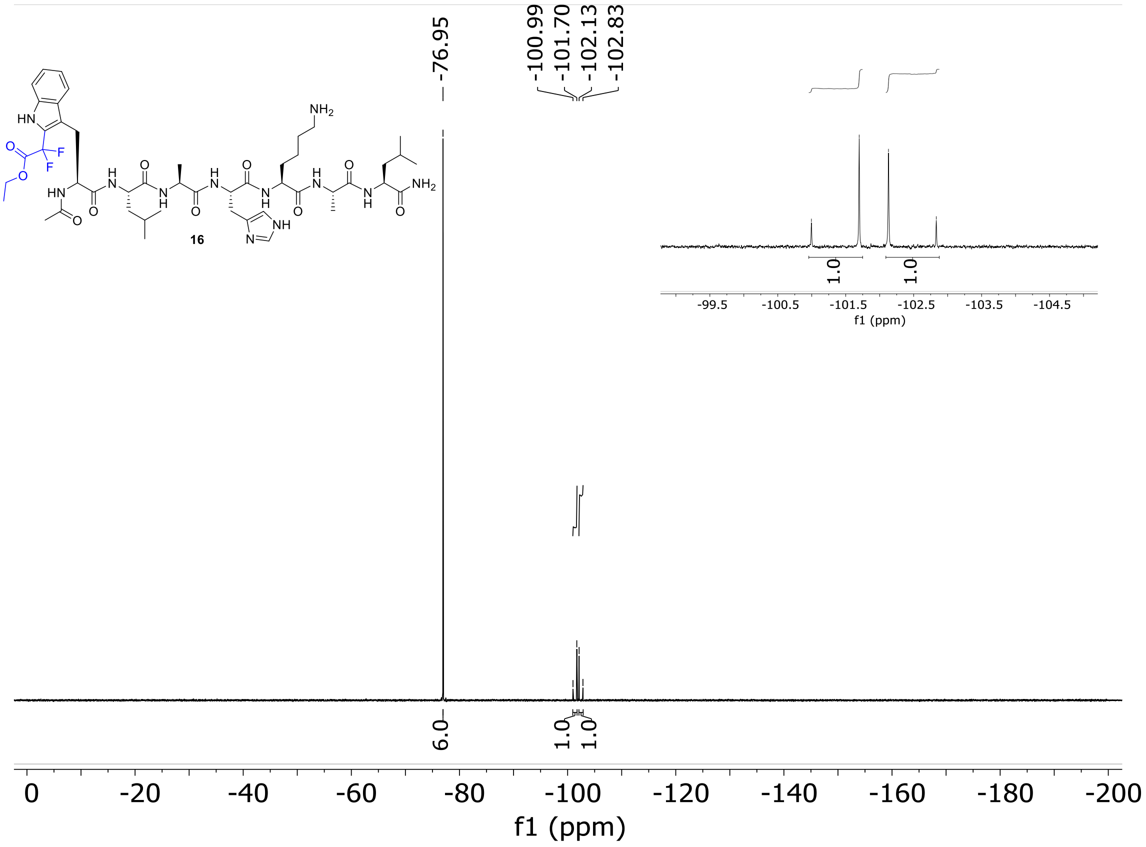

^{1}**H NMR** spectrum of product **17** (500 MHz, DMSO-d_6)

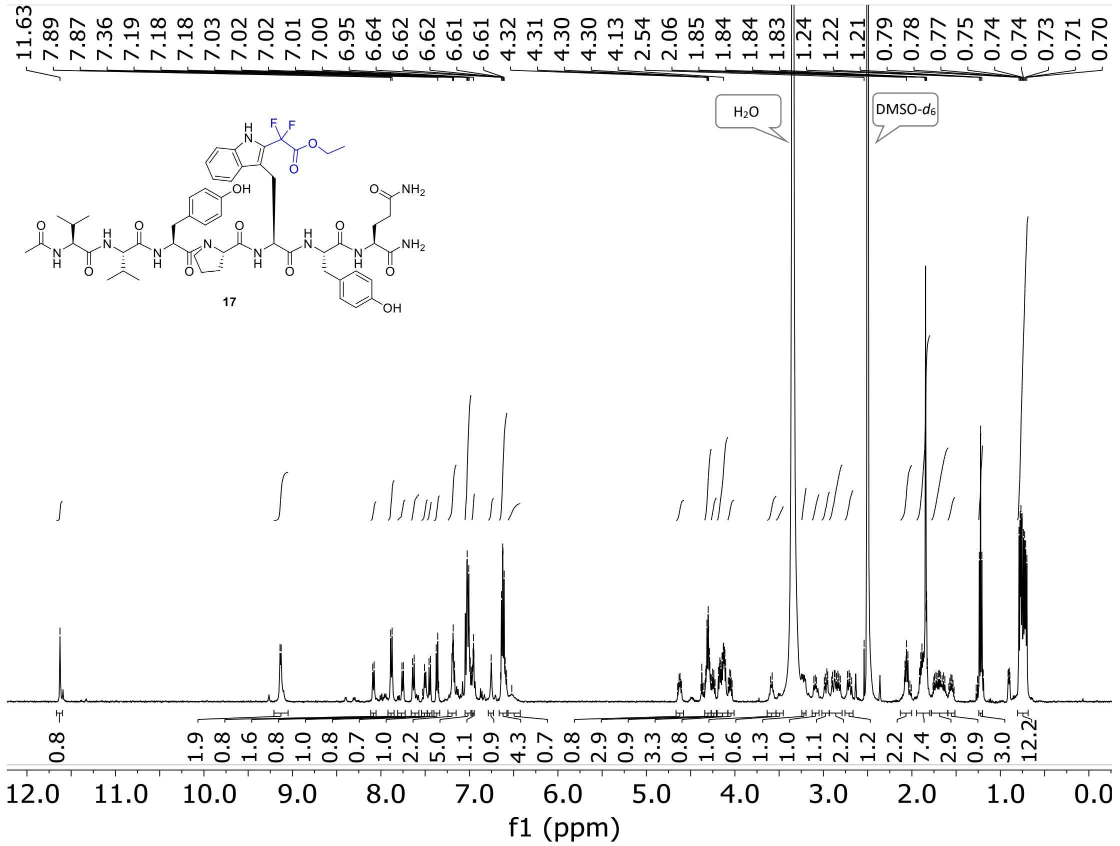

^{19}**F NMR** spectrum of product **17** (376 MHz, DMSO-d_6)

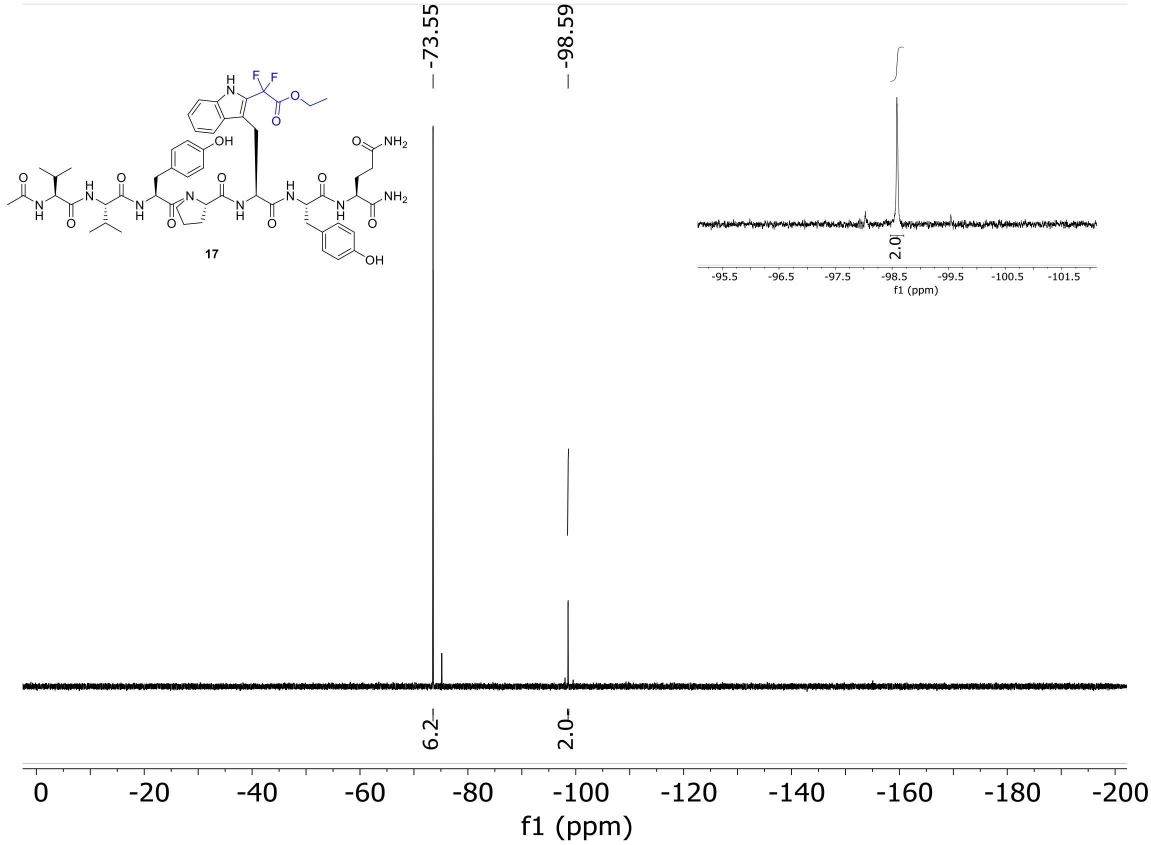

^{1}H NMR spectrum of product **18** (400 MHz, CD_3OD)

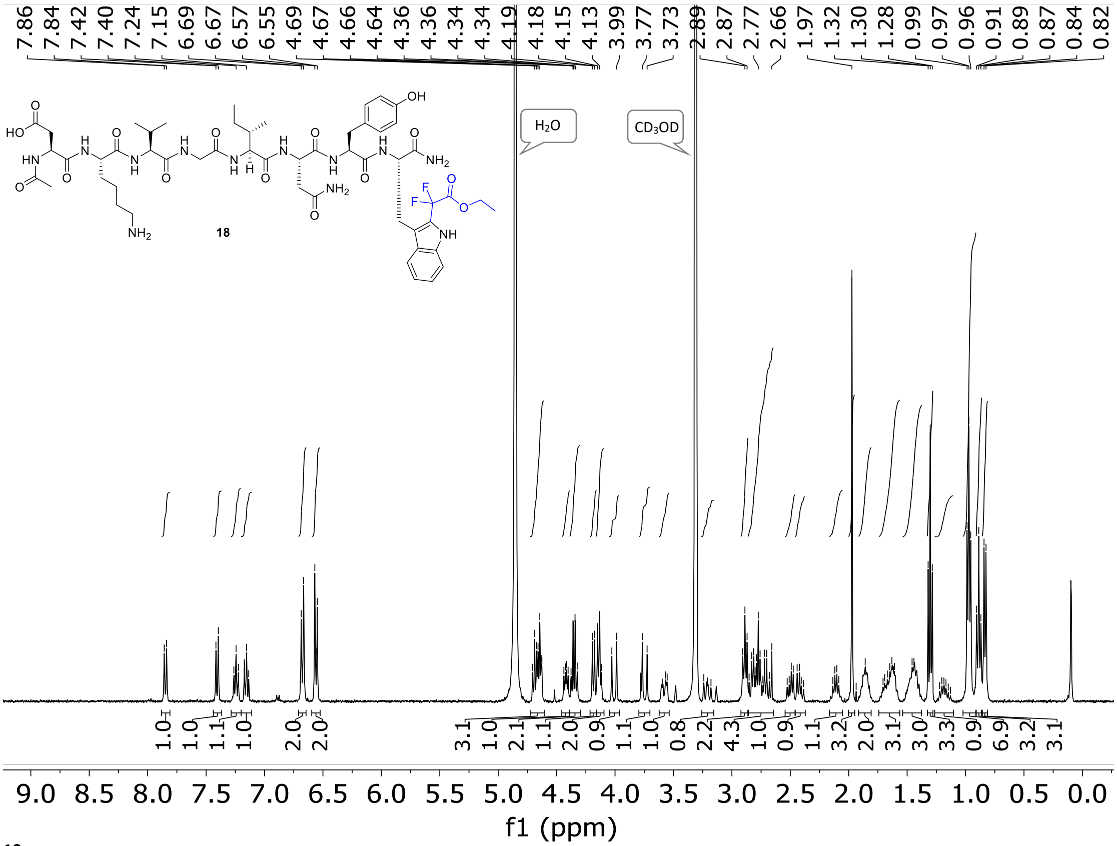

^{19}F NMR spectrum of product **18** (376 MHz, CD_3OD)

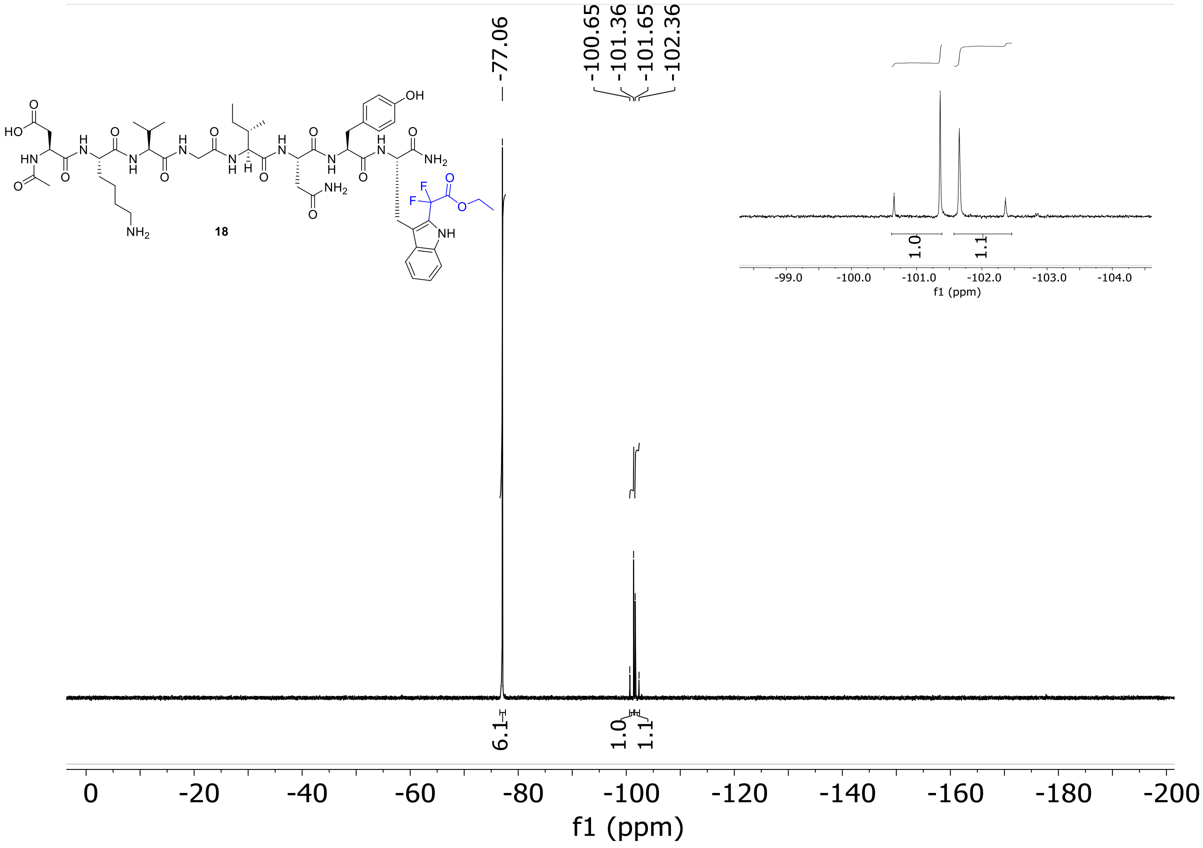

^{1}H NMR spectrum of product **19** (400 MHz, CD_3OD)

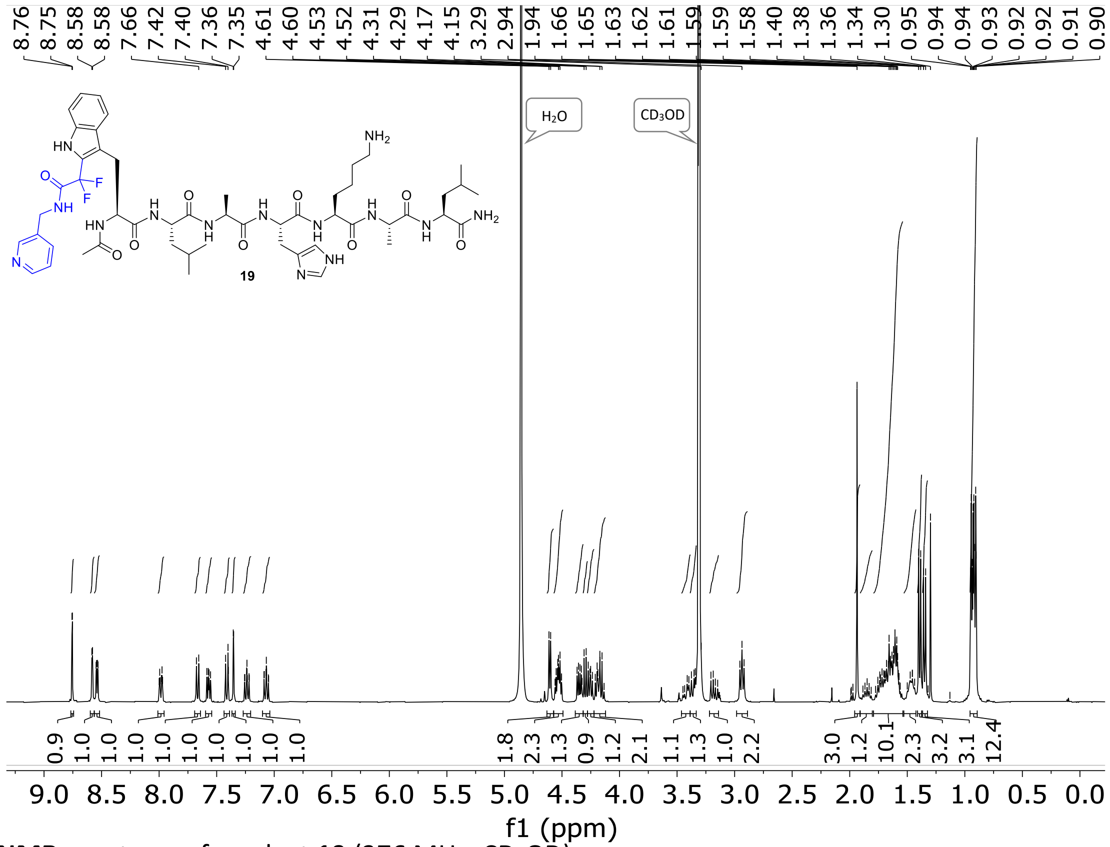

^{19}F NMR spectrum of product **19** (376 MHz, CD_3OD)

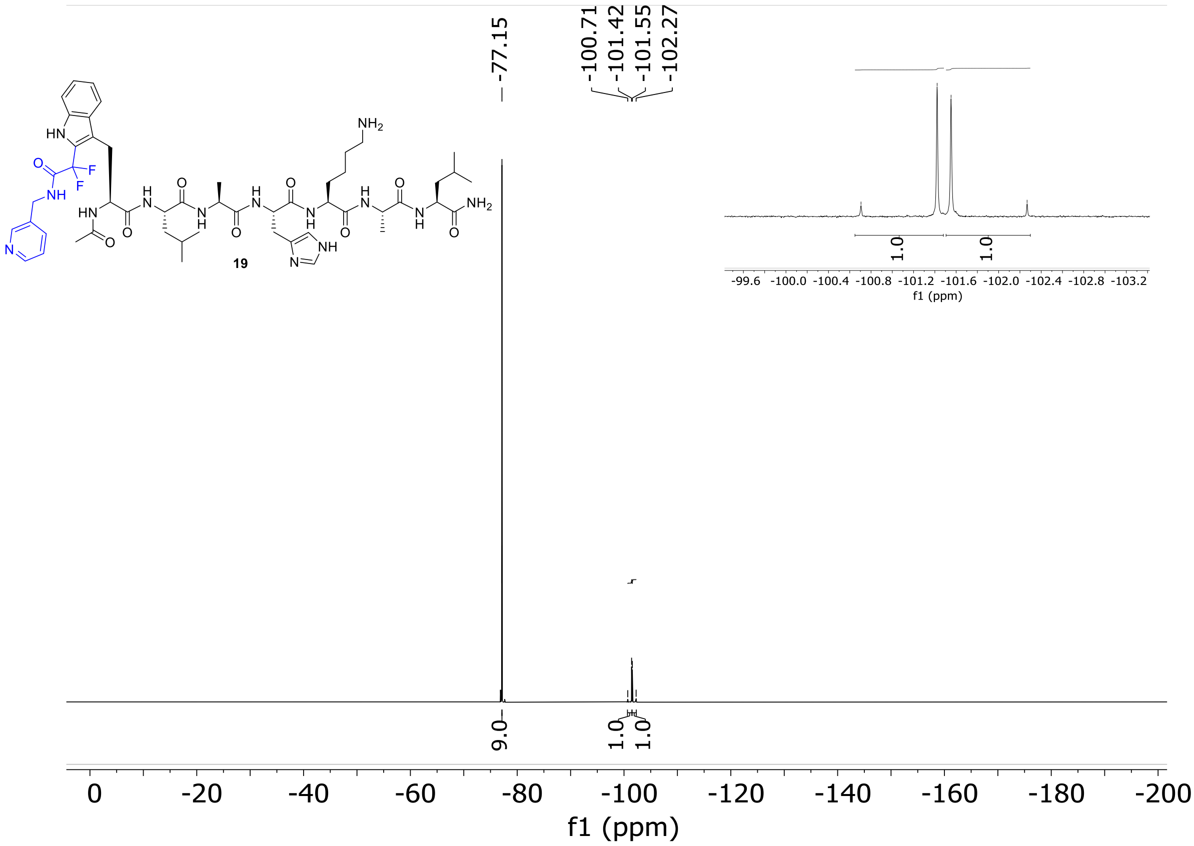

^{1}H NMR spectrum of product **20** (500 MHz, DMSO-d_6)

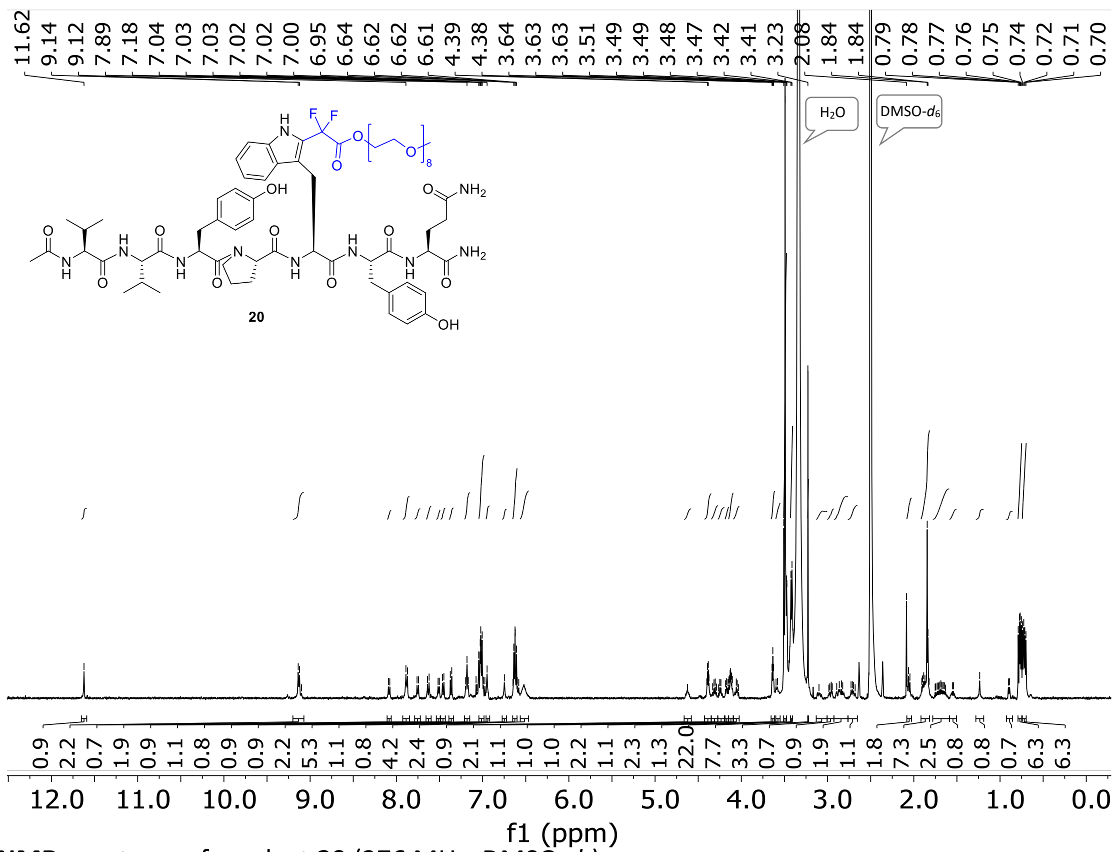

^{19}F NMR spectrum of product **20** (376 MHz, DMSO-d_6)

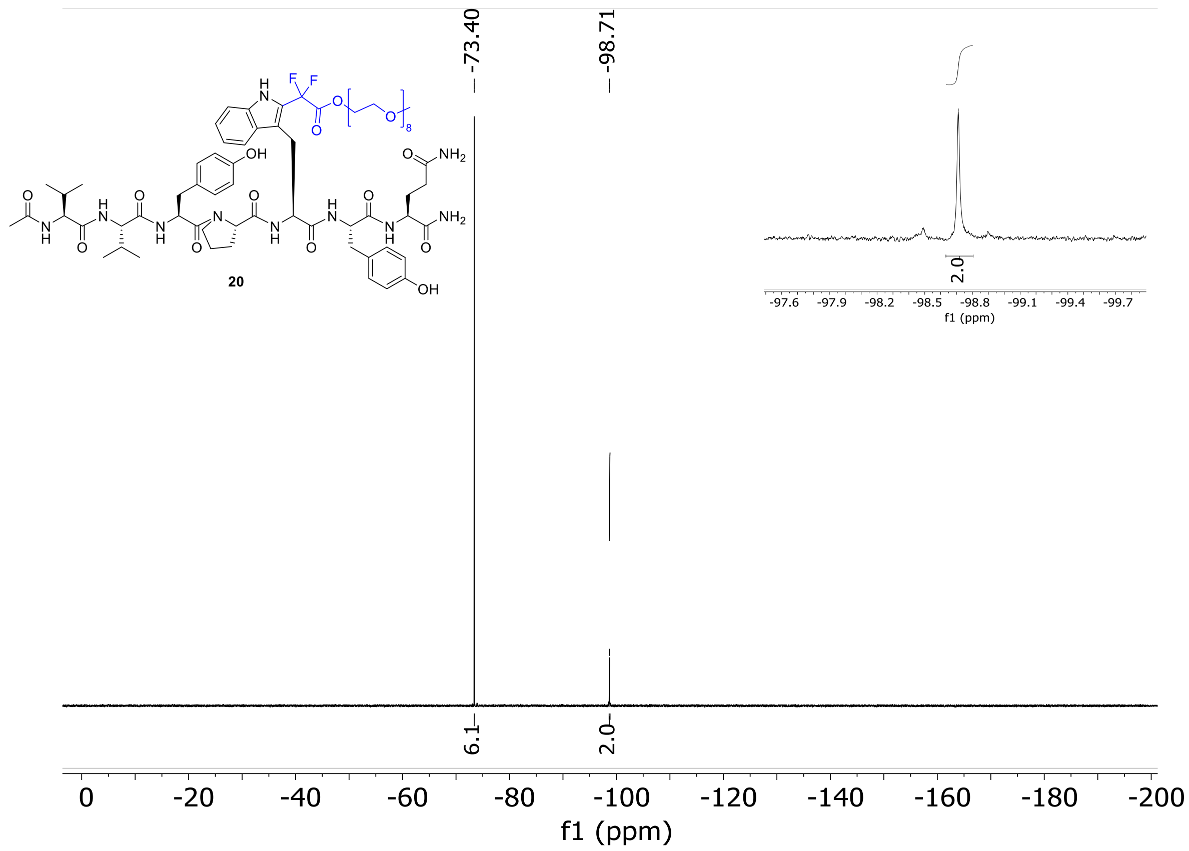

^{1}H NMR spectrum of product **21** (400 MHz, CD_3OD)

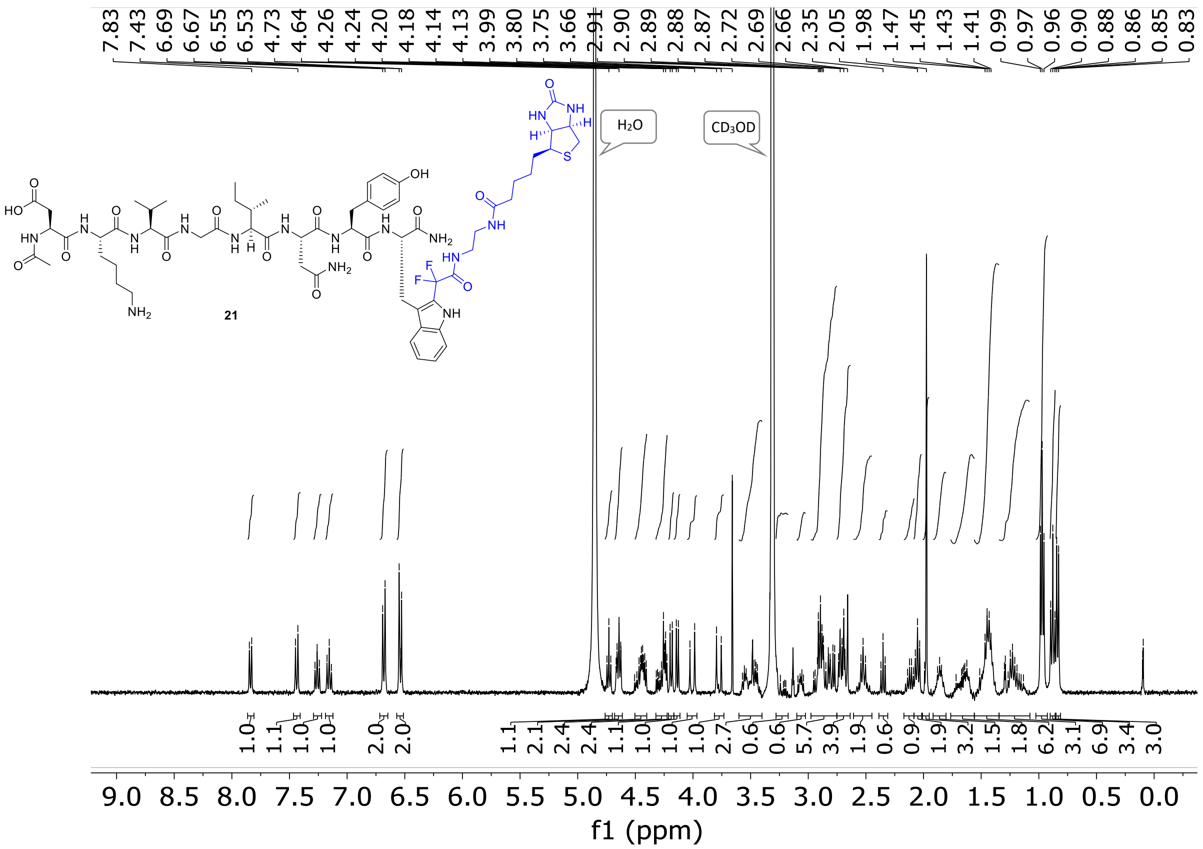

^{19}F NMR spectrum of product **21** (376 MHz, CD_3OD)

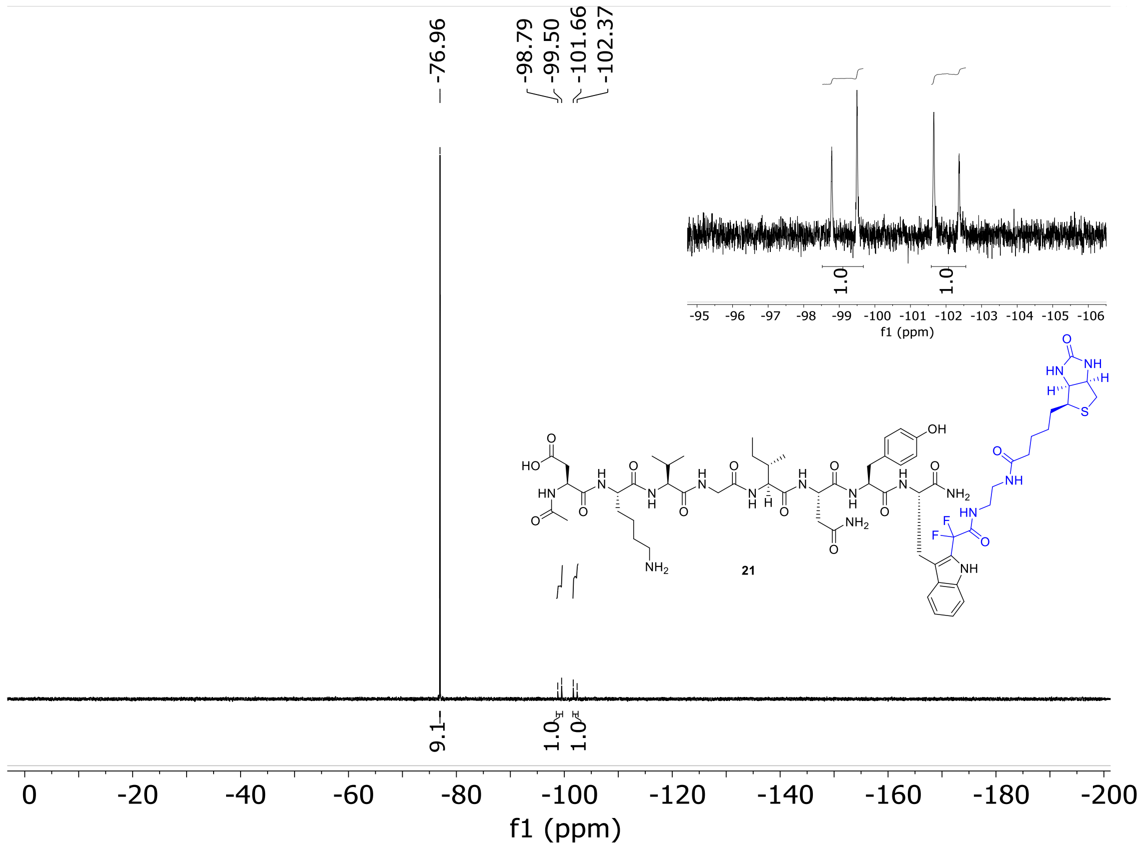

Supplement: Supplementary file 1 — ol3c01795_si_001.pdf [file ol3c01795_si_001.pdf]
